# Genetic association of hypertension and several other metabolic disorders with Bell’s palsy

**DOI:** 10.3389/fgene.2023.1077438

**Published:** 2023-07-18

**Authors:** Huawei Liu, Qingyan Sun, Wenting Bi, Xiaodan Mu, Yongfeng Li, Min Hu

**Affiliations:** ^1^ Department of Stomatology, The First Medical Center, Chinese PLA General Hospital, Beijing, China; ^2^ Department of Stomatology, Beijing Hospital of Integrated Chinese and Western Medicine, Beijing, China; ^3^ Department of Stomatology, Beijing Friendship Hospital, Capital Medical University, Beijing, China; ^4^ Department of Stomatology, Beijing Tsinghua Changgung Hospital, School of Clinical Medicine, Tsinghua University, Beijing, China

**Keywords:** Bell palsy, body mass index, diabetes mellitus, hypertension, obesity

## Abstract

**Background:** Effects of hypertension, type 2 diabetes and obesity on Bell’s palsy risk remains unclear. The aim of the study was to explore whether hypertension and these metabolic disorders promoted Bell’s palsy at the genetic level.

**Methods:** Genetic variants from genome-wide association studies for hypertension, type 2 diabetes, body mass index and several lipid metabolites were adopted as instrumental variables. Two-sample Mendelian randomization including IVW and MR-Egger was used to measure the genetic relationship between the exposures and Bell’s palsy. Sensitivity analyses (i.e., Cochran’s Q test, MR-Egger intercept test, “leave-one-SNP-out” analysis and funnel plot) were carried out to assess heterogeneity and horizontal pleiotropy. All statistical analyses were performed using R software.

**Results:** Hypertension was significantly associated with the increased risk of Bell’s palsy (IVW: OR = 2.291, 95%CI = 1.025–5.122, *p* = 0.043; MR-Egger: OR = 16.445, 95%CI = 1.377–196.414, *p* = 0.029). Increased level of LDL cholesterol might upexpectedly decrease the risk of the disease (IVW: OR = 0.805, 95%CI = 0.649–0.998, *p* = 0.048; MR-Egger: OR = 0.784, 95%CI = 0.573–1.074, *p* = 0.132). In addition, type 2 diabetes, body mass index and other lipid metabolites were not related to the risk of Bell’s palsy. No heterogeneity and horizontal pleiotropy had been found.

**Conclusion:** Hypertension might be a risk factor for Bell’s palsy at the genetic level, and LDL cholesterol might reduce the risk of the disease. These findings (especially for LDL cholesterol) need to be validated by further studies.

## 1 Introduction

Bell’s palsy is one type of peripheral facial nerve palsy, and is more common in young adults than in other age groups (Holland and Bernstein, 2014; Vakharia and Vakharia, 2016). Patients usually do not experience any prodromal symptoms, but find a series of facial paralysis symptoms directly when they wake up in the morning (Holland and Bernstein, 2014; Vakharia and Vakharia, 2016). At present, the causes and risk factors for Bell’s palsy are not well understood. Previous studies reported that viral infections, blood vessel disorders, immunodeficiency, genetic defects and physical stress were involved in the pathogenesis of Bell’s palsy (Amit, 1987; Browning, 2010; Tseng et al., 2017; Grewal, 2018; Yamamoto et al., 2022). However, a firmed conclusion can not be drawn, and more research about this topic are ongoing.

An increasing number of evidence had reported that several chronic diseases might be associated with the development and progression of Bell’s palsy. Stamatiou et al. concluded recently that the risk of Bell’s palsy was significantly increased in patients with type 2 diabetes mellitus (T2DM), while T2DM may contribute to severe facial nerve degeneration (Stamatiou et al., 2022). Savadi-Oskouei et al. in their study reported that hypertension was related to the increased risk of Bell’s palsy among those patients aged above 40 years (Savadi-Oskouei et al., 2008). Psillas et al. suggested that the mean severity of Bell’s palsy was more significant in patients with hypercholesterolemia, T2DM or hypertension, in comparison to control group (Psillas et al., 2021). Meanwhile, patients suffering from Bell’s palsy and concomitant comorbidities had a poorer prognosis compared to patients without comorbidities (Psillas et al., 2021). Kim et al. using a national health screening cohort revealed that obesity was associated with the risk of Bell’s palsy in the population over 40 years old (Kim et al., 2020). However, all these findings were obtained from previous observational studies, which can not avoid the effects of confounding factors and can not distinguish the chronological order of research factors.

Mendelian randomization studies adopt genetic variants to replace traits, and the latters usually refer to exposures or outcomes in observational studies. Thus, Mendelian randomization studies explain the potential effect of one exposure on one outcome by studing the relationship between the corresponding two genetic variants. The characteristics of this genetic method determine that it may make up for the above shortcomings of observational studies and provide more reliable findings (Emdin et al., 2017; Sanderson, 2021). However, there was no Mendelian randomization studies focusing on the association of T2DM, hypertension and obesity with Bell’s palsy at present.

Taken together, the study adopted a well-designed Mendelian randomization study to explore the genetic effects of several chronic diseases, such as T2DM, hypertension and obesity, on the risk of Bell’s palsy, and also to reveal the potential effects of several blood lipids, lipoproteins and apolipoproteins on the risk of the disease.

## 2 Methods

### 2.1 Data retrieval for Mendelian randomization analyses

As mentioned above, Mendelian randomisation studies require specific genetic variants that must be associated with a certain traits to be studied. Because genome-wide association studies (GWASs) are a type of studies that explain the correlation between genetic variants and traits, GWAS can provide the genetic variants required for Mendelian randomisation studies (Dehghan, 2018).

The traits required for this study were described here. Bell’s palsy was the outcome. T2DM, hypertension and obesity were defined as the primary exposures. Of these, obesity was expressed as body mass index (BMI). As lipid metabolism was associated with all three of the primary exposures, several lipid markers (i.e., triglycerides, LDL cholesterol, HDL cholesterol, lipoprotein A-I, lipoprotein B and lipoprotein A) were considered as secondary exposures and were included in the study.

The summary data of Bell’s palsy was collected from a FinnGen study including 1,740 cases and 195,047 controls of European individuals (both sexes, mean age: 51 years) from the IEU Open GWAS Project (https://gwas.mrcieu.ac.uk), and its ID was finn-b-G6_BELLPA.

The summary data of T2DM was obtained from a meta-analysis of GWASs in a very large sample of T2DM (62,892 cases and 596,424 controls, both sexes, adults), by combining three GWAS data sets of European ancestry: DIAbetes Genetics Replication and Meta-analysis (DIAGRAM), Genetic Epidemiology Research on Aging (GERA), and the full cohort release of the UK Biobank (UKB) (Xue et al., 2018). The summary data of hypertension was included from a Neale Lab UKB GWAS including 87,690 cases and 249,469 controls of European individuals (both sexes, adults) from the IEU Open GWAS Project, and its ID was ukb-a-61. The summary data of BMI was provided by a meta-analysis of GWASs for height and BMI in up to 700,000 individuals of European ancestry (both sexes, any age) (Yengo et al., 2018).

The summary data for triglycerides, LDL cholesterol, HDL cholesterol, apolipoprotein A-I, apolipoprotein B were collected from a GWAS of circulating non-fasted lipoprotein lipid traits in UKB including 393,193 to 441,016 European individuals (both sexes, mean age: 57 years) (Richardson et al., 2020). The summary data for lipoprotein A was obtained from a Neale Lab UKB GWAS using 361,194 samples of white-British ancestry (both sexes, any age) from the IEU Open GWAS Project, and its ID was ukb-d-30790_raw.

The characteristics of the GWAS in the present study were showed in Table 1.

**TABLE 1:** Characteristics of the GWAS in the study.

Traits	GWAS ID	Year	Population	Sex	Consortium^a^	Sample size	No. of suitable SNPs
Exposures							
Type 2 diabetes	ebi-a-GCST006867	2018	European	Both sexes	EBI	655,666	103
Hypertension	ukb-a-61	2017	European	Both sexes	Neale Lab	337,159	124
Body mass index	ieu-b-40	2018	European	Both sexes	GIANT	681,275	453
Triglycerides	ieu-b-111	2020	European	Both sexes	United Kingdom Biobank	441,016	233
LDL cholesterol	ieu-b-110	2020	European	Both sexes	United Kingdom Biobank	440,546	126
HDL cholesterol	ieu-b-109	2020	European	Both sexes	United Kingdom Biobank	403,943	256
Apolipoprotein A-I	ieu-b-107	2020	European	Both sexes	United Kingdom Biobank	393,193	222
Apolipoprotein B	ieu-b-108	2020	European	Both sexes	United Kingdom Biobank	439,214	137
Lipoprotein A	ukb-d-30790_raw	2018	European	Both sexes	Neale lab	361,194	13
Outcome							
Bell’s palsy	finn-b-G6_BELLPA	2021	European	Both sexes	FinnGen	196,787	—

^a^ EBI, european bioinformatics institute; GIANT, genetic investigation of anthropometric traits consortium.

### 2.2 Selection of genetic instrumental variables

Single nucleotide polymorphisms (SNPs) are one of the most common genetic variants in humans. In Mendelian randomisation studies, SNPs are extracted from summary data of GWAS and used as instrumental variables. The criteria for extracting SNPs are: 1) SNPs must be associated with the exposures to be studied. 2) SNPs are not associated with the confounding factors affecting the relationship between exposures and outcomes. 3) SNPs can only have an effect on outcomes through exposures (Burgess et al., 2017).

In this study, each exposure-related SNP was genome-wide significant (*p* < 5 × 10^−8^, F-statistic >10) and independent (linkage disequilibrium *r* ^2^ < 0.001, distance = 10,000 kb) (Birney, 2022). These exposure-related SNPs were then collected from the outcome dataset. To correct for allelic orientation, SNP harmonization was performed so that the effect of one SNP on exposure and the effect of that SNP on outcome must correspond to the same allele, respectively (Birney, 2022). After that, all SNPs related to Bell’s palsy or confounding factors were manually removing using the PhenoScanner Pheno Scanner V2, and the confounding factors were viral infections, blood vessel disorders, immunodeficiency, genetic defects, physical stress and their synonyms (Amit, 1987; Browning, 2010; Tseng et al., 2017; Grewal, 2018; Yamamoto et al., 2022).

### 2.3 Statistic analysis

In the study, two-sample Mendelian randomization analysis was performed using random-effect inverse variance weighted (IVW) and MR-Egger methods. The IVW is a method used under more ideal conditions, which assumes that all genetic variants are valid instrumental variables with strong causality detection (Emdin et al., 2017; Birney, 2022). The MR-Egger, on the other hand, considers the presence of intercept terms, that is, the presence of genetic pleiotropy. This method tolerates the fact that all instrumental variables are pleiotropic, but these pleiotropies cannot influence the correlation between genetic variants and exposures (Bowden et al., 2016). Thus, the MR-Egger method can be adapted to a wider range of conditions, yielding more conservative results, but with less detectability than IVW. Both methods can report odds ratios (ORs), 95%CIs and *p* values. In the present study, the correlation was statistically significant when the *p*-value for IVW was less than 0.05 and the MR-Egger result was in the same direction as IVW. Furthermore, these results were visualized using forest plots and scatter plots. A forest plot showed the result from each SNP and the pooled result from all the SNPs. A scatter plot showed the results from the two methods at the same time.

A series of sensitivity analyses were also carried out in this study, such as Cochran’s Q test, MR-Egger intercept test, “leave-one-SNP-out” analysis and funnel plot (Zheng et al., 2017; Bowden et al., 2018). The Cochran’s Q test was adopted to assess heterogeneity. The MR-Egger intercept test was used to detect horizontal pleiotropy. In a “Leave-one-SNP-out” analysis, each SNP was sequentially removed from the analyses to assess its contribution to the final results. In a funnel plot, possible bias among the SNPs were detected, and if the plot was relatively symmetrical, this indicated that the study was not affected by bias.

All analyses was done using TwoSampleMR R package (version 3.5.2).

## 3 Results

As shown in Table 1, a total of 13–453 suitable SNPs were selected to predict T2DM, hypertension, BMI, several blood lipids, apolipoproteins and lipoproteins. All SNPs (including their F-statistics) were listed in Supplementary Table S1.

### 3.1 Effect of hypertension, T2DM and BMI on Bell’s palsy risk

**FIGURE 1:**
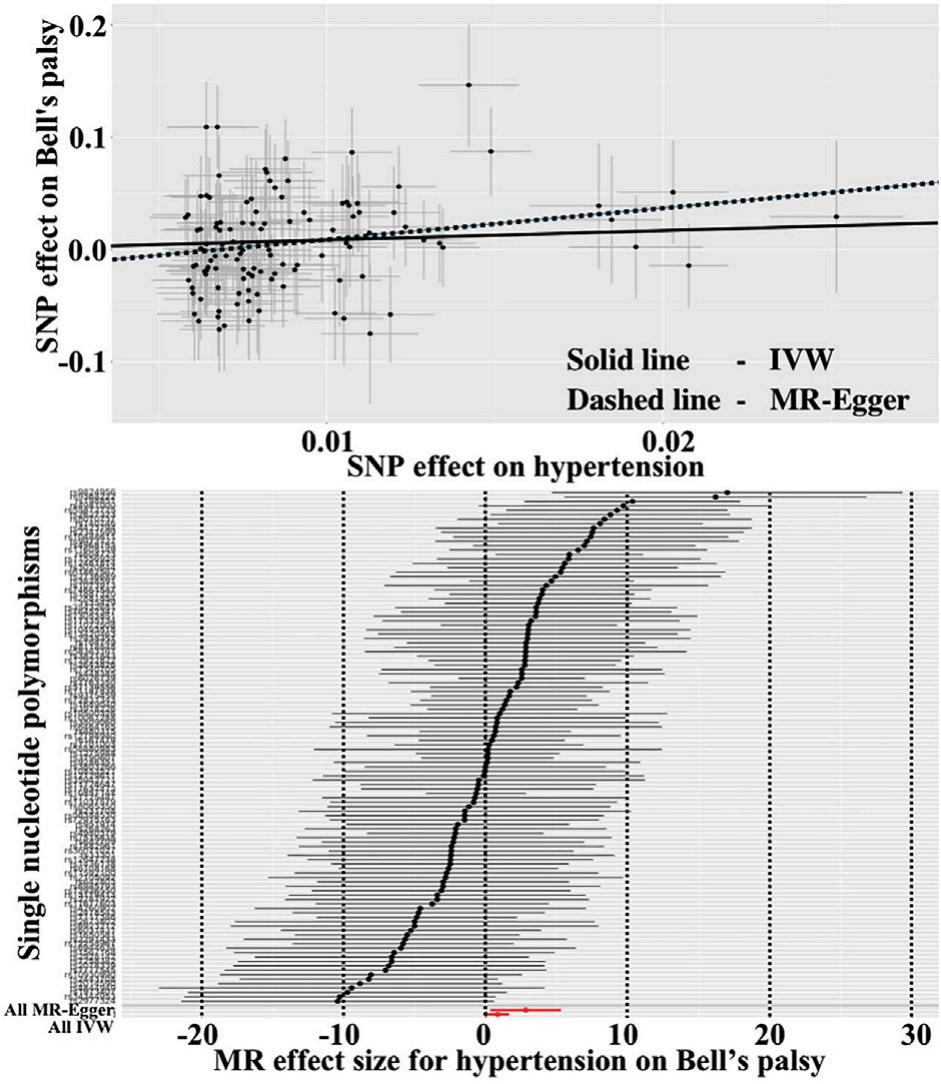
Scatter and forest plots for hypertension affecting Bell’s palsy risk.

As shown in Table 2, the IVW initially did not find a correlation between hypertension and Bell’s palsy. However, the “leave-one-SNP-out” analysis found an outlier (rs8027450). After removing the outlier, the IVW was conducted again and reported that hypertension significantly increased the risk of Bell’s palsy (OR = 2.291, 95%CI = 1.025–5.122, *p* = 0.043). The MR-Egger also provided a consistent result, although it had a relatively wide 95%CI (OR = 16.445, 95%CI = 1.377–196.414, *p* = 0.029). Cochran’s Q test did not report any heterogeneity (*p* = 0.227), and MR-Egger intercept test did not find any horizontal pleiotropy (*p* = 0.103). Scatter and forest plots for hypertension affecting Bell’s palsy risk was showed in Figure 1. In addition, potential horizontal pleiotropy was also not reported by funnel plot and “leave-one-SNP-out” analysis in Supplementary Figure S2.

**TABLE 2:** Effects of type 2 diabetes, hypertension and obesity on Bell’s palsy risk using IVW and MR-Egger in the study.

Exposures	Methods	No. of SNP	OR (95%CI)	*p*-Value for MR	*p*-Value for heterogeneity^b^	*p*-Value for horizontal pleiotropy^b^
Type 2 diabetes	IVW	99	1.069 (0.947–1.206)	0.280	0.024	0.578
	MR-Egger	99	1.150 (0.866–1.525)	0.336		
Hypertension	IVW	117	1.976 (0.869–4.491)	0.104	0.113	0.190
	MR-Egger	117	9.968 (0.785–126.525)	0.079		
Removing rs8027450	IVW	116	2.291 (1.025–5.122)	0.043	0.227	0.103
	MR-Egger	116	16.445 (1.377–196.414)	0.029		
Body mass index	IVW	432	1.168 (0.934–1.459)	0.173	0.507	0.624
	MR-Egger	432	1.338 (0.742–2.412)	0.333		

^a^ Heterogeneity was assessed using the Cochran’s Q test, and horizontal pleiotropy was assessed using the MR-Egger intercept test.

As shown in Table 2, the IVW reported that T2DM and BMI did not associated with the risk of Bell’s palsy (OR = 1.069, 95%CI = 0.947–1.206, *p* = 0.280; OR = 1.168, 95%CI = 0.934–1.459, *p* = 0.173). Visualised results and sensitivity analyses were listed in Table 2, Supplementary Figure S1, 3.

### 3.2 Effect of several lipids, apolipoproteins and lipoproteins on Bell’s palsy risk

**FIGURE 2:**
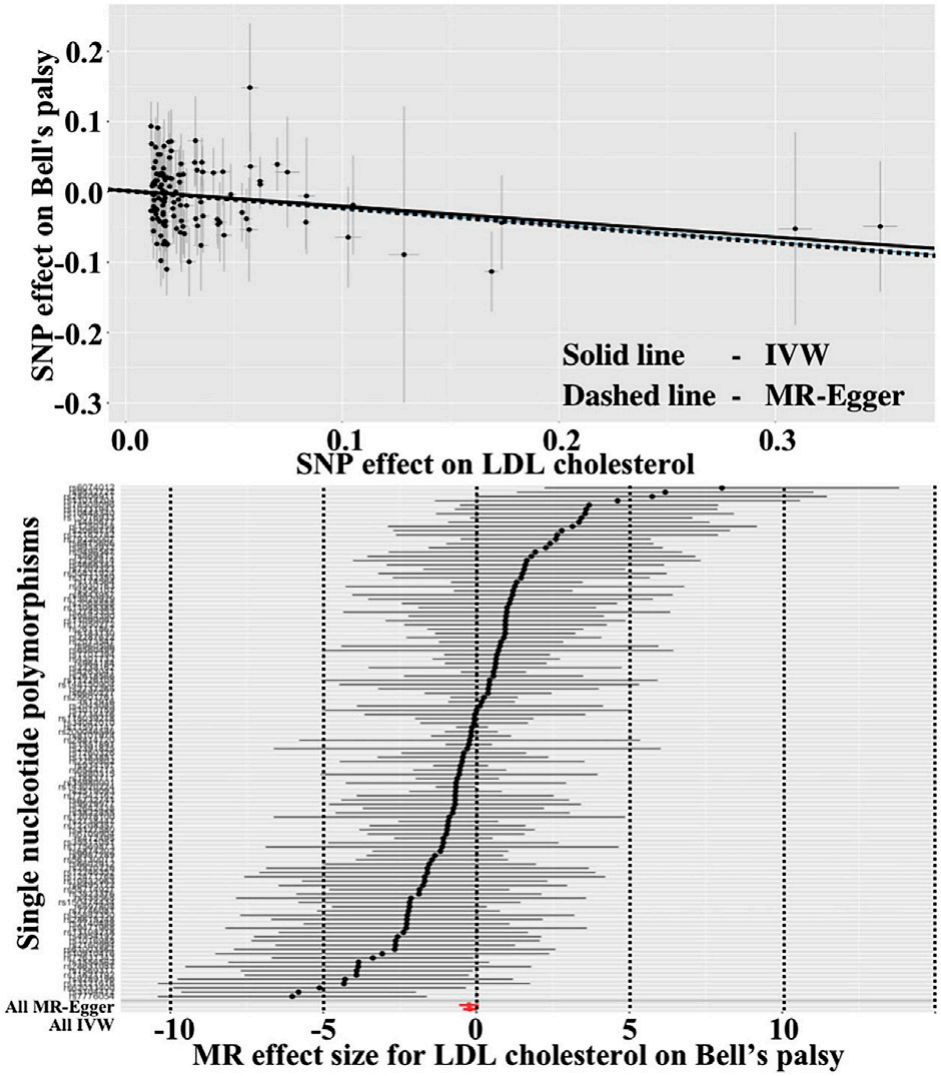
Scatter and forest plots for LDL cholesterol affecting Bell’s palsy risk.

As show in Table 3, the IVW reported that LDL cholesterol was not associated with the risk of Bell’s palsy (OR = 0.836, 95%CI = 0.678–1.031, *p* = 0.094). However, the “leave-one-SNP-out” analysis found an outlier (rs497083). After removing the outlier, the IVW revealed that increased level of LDL cholesterol signficantly decreased the risk of the disease (OR = 0.805, 95%CI = 0.649–0.998, *p* = 0.048), and the MR-Egger reported a result in the same direction (OR = 0.784, 95%CI = 0.573–1.074, *p* = 0.132). Heterogeneity and horizontal pleiotropy were not found separately by Cochran’s Q test and MR-Egger intercept test (*p* = 0.129, *p* = 0.823). Visualised results for this estimate were showed in Figure 2. Funnel plot and “leave-one-SNP-out” analysis also did not find any horizontal pleiotropy in Supplementary Figure S5.

**TABLE 3:** Effects of several blood lipids, lipoproteins and apolipoproteins on Bell’s palsy risk using IVW and MR-Egger in the study.

Exposures	Methods	No. of SNP	OR (95%CI)	*p*-Value for MR	*p*-Value for heterogeneity^a^	*p*-Value for horizontal pleiotropy^a^
Triglycerides	IVW	220	1.034 (0.872–1.228)	0.698	0.511	0.479
	MR-Egger	220	0.968 (0.753–1.244)	0.799		
LDL cholesterol	IVW	121	0.836 (0.678–1.031)	0.094	0.114	0.975
	MR-Egger	121	0.833 (0.614–1.130)	0.242		
Removing rs497083	IVW	120	0.805 (0.649–0.998)	0.048	0.129	0.823
	MR-Egger	120	0.784 (0.573–1.074)	0.132		
HDL cholesterol	IVW	250	0.900 (0.763–1.062)	0.213	0.575	0.649
	MR-Egger	250	0.940 (0.733–1.204)	0.624		
Apolipoprotein A-I	IVW	216	0.987 (0.818–1.191)	0.891	0.096	0.250
	MR-Egger	216	1.130 (0.839–1.522)	0.421		
Apolipoprotein B	IVW	134	0.817 (0.687–0.972)	0.023	0.366	0.601
	MR-Egger	134	0.851 (0.675–1.071)	0.172		
Removing rs143020224	IVW	133	0.844 (0.706–1.010)	0.063	0.391	0.463
	MR-Egger	133	0.896 (0.705–1.138)	0.370		
Lipoprotein A	IVW	12	1.000 (0.998–1.001)	0.775	0.388	0.320
	MR-Egger	12	1.000 (0.998–1.002)	0.692		

^a^ Heterogeneity was assessed using the Cochran’s Q test, and horizontal pleiotropy was assessed using the MR-Egger intercept test.

**FIGURE 3:**
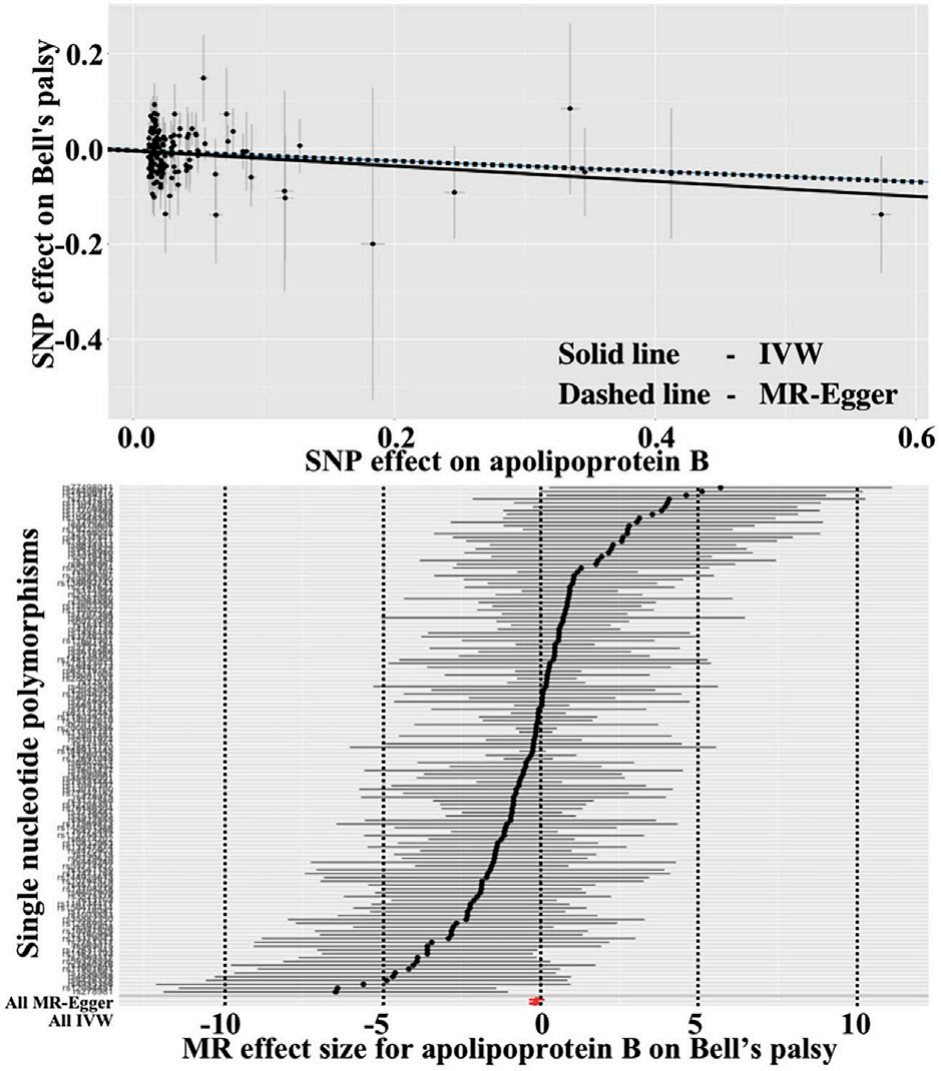
Scatter and forest plots for alipoprotein B affecting Bell’s palsy risk.

As show in Table 3, the IVW reported that increased level of apolipoprotein B was associated with the decreased risk of Bell’s palsy (OR = 0.817, 95%CI = 0.687–0.972, *p* = 0.023). However, after removing one outlier (rs143020224) which was detected by “leave-one-SNP-out” analysis, the relationship between apolipoprotein B and Bell’s palsy disappearred in the IVW (OR = 0.844, 95%CI = 0.706–1.010, *p* = 0.063). Scatter and forest plots for alipoprotein B affecting Bell’s palsy risk were showed in Figure 3. Funnel plot and “leave-one-SNP-out” analysis was listed in Supplementary Figure S8.

In addition, the IVW did not find any association of triglycerides, HDL cholesterol, apolipoprotein A-I and lipoprotein A with the risk of Bell’s palsy (*p* > 0.05). The results and sensitivity analyses were listed in Table 3 and Supplementary Figure S4, 6, 7, 9.

## 4 Discussion

To our knowledge, this was the first Mendelian randomization study focusing on the association of hypertension with Bell’s palsy. Based on the results from the study, we reported that hypertension might increase the risk of Bell’s palsy by about 130% at the genetic level. As mentioned above, a great number of traits, such as viral infections, blood vessel disorders, immunodeficiency, genetic defects and physical stress, contributed to the occurrence of Bell’s palsy (Amit, 1987; Browning, 2010; Tseng et al., 2017; Grewal, 2018; Yamamoto et al., 2022), and should be regarded as confounding factors in the study. So, all the instrumental variables related to these confounding factors were manually excluded using PhenoScanner Pheno Scanner V2 prior to conducting Mendelian randomisation analyses. All the instrumental variables were also consistent with the principles of relevance and independence. Therefore, we believed that a causal correlation between hypertension and Bell’s palsy was relatively well established.

A number of observational studies had explored the relationship between hypertension and Bell’s palsy. Savadi-Oskouei et al. and Psillas et al. separately reported that hypertension may be related to the increased risk of Bell’s palsy in different populations (Savadi-Oskouei et al., 2008; Psillas et al., 2021). Two earlier studies also showed that hypertension might be an potential predisposing and prognostic factor in Bell’s palsy, though a firm conclusion can not be drawn (Abraham-Inpijn et al., 1982; Abraham-Inpijn et al., 1987). These were consistent with the results of the present study.

The mechanisms by which hypertension promoted the development of Bell’s palsy were unclear and no previous studies had been published. As hypertension was an important vasculogenic factor and nerve ischaemia was a potential cause of Bell’s palsy (Grewal, 2018; Humphrey, 2021), we speculated that hypertension may cause nerve damage by affecting the blood supply to the associated facial nerve, which in turn induced Bell’s palsy.

This study also found, for the first time, an unexpected result of an inverse causal relationship between LDL cholesterol and Bell’s palsy. For each standard deviation increase in LDL cholesterol levels, the risk of the disease was reduced by approximately 20%. This result seemed to contradict the current understanding of LDL cholesterol, which was now widely regarded as a risk factor for atherosclerosis and coronary heart disease (Navarese et al., 2018). However, some scholars still had opposite views on the role of LDL cholesterol (Ravnskov et al., 2018). Perhaps the role of this cholesterol was more complex than one might think. In addition, this result did not exist in isolation. The present study also showed that increased level of apolipoprotein B was almost associated with a reduced risk of Bell’s palsy, although this correlation was not statistically significant after excluding one outlier. Whereas apolipoprotein B was the main structural protein of LDL cholesterol, measurement of apolipoprotein B gave a direct indication of LDL cholesterol level (Sniderman et al., 2021). Therefore, the causal relationship between LDL cholesterol and Bell’s palsy must be revalidated and the underlying mechanisms should be further explored.

In addition, the present study did not find any association of T2DM and obesity with Bell’s palsy risk. However, a number of published observational studies suggested that T2DM and obesity contributed to the development of Bell’s palsy, which was inconsistent with the present study (Kim et al., 2020; Psillas et al., 2021; Stamatiou et al., 2022). One reasonable explanation was that although there was no genetic relationship, some uncertain traits may act as an intermediary and link these two diseases (i.e., T2DM and obesity) and Bell’s palsy. For example, the trait that most readily came to mind was the inflammatory response. Both obesity and T2DM were accompanied by systemic metabolic inflammation, while inflammation was one of the important pathophysiological changes in Bell’s palsy (Kınar et al., 2021). It was possible that obesity and T2DM promoted the development of Bell’s palsy through inflammatory pathways, rather than genetic variants.

As we all know, observational studies had a range of congenital deficiencies, which made them difficult to avoid the interference of reverse causality and confounding factors (Bowden and Holmes, 2019). Mendelian randomization studies adopted specific genetic variants (i.e., SNPs or instrumental variables) to replace traits (i.e., exposures or outcomes). The design overcame the deficiencies and led to more reliable conclusions. This was the main advantage of this study.

As a two-sample Mendelian randomisation study, the summary data for the outcome were all from FinnGen, while the summary data for the exposures were mainly from UKB, whose population included only a very small proportion of Finnish individuals. According to previous studies, the European population can be divided into two subgroups, namely, Finns and non-Finnish Europeans (Liu and Fu, 2015; Lek et al., 2016; Gilbert et al., 2022). In this sense, the exposures and outcomes in this study can be considered to be from two different populations, which would affect the estimation of causal effects. Although most previously published two-sample Mendelian randomisation studies had not distinguished between these two European subgroups, this did represent a potential limitation of the study. For this reason, this study initially compared allele frequencies of the exposures and outcome for all instrumental variables and did not find substantial differences between them. In addition, we also provided these allele frequencies in Supplementary Table S1 to make these information public. We hoped that future research may overcome this limitation and validate the reliability of our findings in different European subgroups.

In conclusion, this study confirmed that hypertension may be a risk factor for Bell’s palsy at the genetic level. Also, this study provided some insufficient evidence to prove that LDL cholesterol might reduce the risk of Bell’s palsy. In addition, no genetic association of other lipids, apolipoproteins and lipoproteins with Bell’s palsy was found. The results of this study (especially for LDL cholesterol) need to be validated by further studies.

## Data Availability

The original contributions presented in the study are included in the article/Supplementary Material, further inquiries can be directed to the corresponding author.

## References

[B1] Abraham-Inpijn L. Devriese P. P. Hart A. A. (1982). Predisposing factors in Bell's palsy: A clinical study with reference to diabetes mellitus, hypertension, clotting mechanism and lipid disturbance. Clin. Otolaryngol. Allied Sci. 7, 99–105. 10.1111/j.1365-2273.1982.tb01569.x 7094388

[B2] Abraham-Inpijn L. Oosting J. Hart A. A. (1987). Bell's palsy: Factors affecting the prognosis in 200 patients with reference to hypertension and diabetes mellitus. Clin. Otolaryngol. Allied Sci. 12, 349–355. 10.1111/j.1365-2273.1987.tb00215.x 2827919

[B3] Amit R. (1987). Familial juvenile onset of Bell's palsy. Eur. J. Pediatr. 146, 608–609. 10.1007/BF02467367 3428296

[B4] Birney E. (2022). Mendelian randomization. Cold Spring Harb. Perspect. Med. 12, a041302. 10.1101/cshperspect.a041302 34872952 PMC9121891

[B5] Bowden J. Del Greco M. F. Minelli C. Davey Smith G. Sheehan N. A. Thompson J. R. (2016). Assessing the suitability of summary data for two-sample mendelian randomization analyses using MR-egger regression: The role of the I2 statistic. Int. J. Epidemiol. 45, 1961–1974. 10.1093/ije/dyw220 27616674 PMC5446088

[B6] Bowden J. Hemani G. Davey Smith G. (2018). Invited commentary: Detecting individual and global horizontal pleiotropy in mendelian randomization-A job for the humble heterogeneity statistic? Am. J. Epidemiol. 187, 2681–2685. 10.1093/aje/kwy185 30188969 PMC6269239

[B7] Bowden J. Holmes M. V. (2019). Meta-analysis and mendelian randomization: A review. Res. Synth. Methods 10, 486–496. 10.1002/jrsm.1346 30861319 PMC6973275

[B8] Browning G. G. (2010). Bell's palsy: A review of three systematic reviews of steroid and anti-viral therapy. Clin. Otolaryngol. 35, 56–58. 10.1111/j.1749-4486.2010.02084.x 20447166

[B9] Burgess S. Small D. S. Thompson S. G. (2017). A review of instrumental variable estimators for Mendelian randomization. Stat. Methods Med. Res. 26, 2333–2355. 10.1177/0962280215597579 26282889 PMC5642006

[B10] Dehghan A. (2018). Genome-wide association studies. Methods Mol. Biol. 1793, 37–49. 10.1007/978-1-4939-7868-7_4 29876890

[B11] Emdin C. A. Khera A. V. Kathiresan S. (2017). Mendelian randomization. JAMA 318, 1925–1926. 10.1001/jama.2017.17219 29164242

[B12] Gilbert E. Shanmugam A. Cavalleri G. L. (2022). Revealing the recent demographic history of Europe via haplotype sharing in the UK Biobank. Proc. Natl. Acad. Sci. U. S. A. 119, e2119281119. 10.1073/pnas.2119281119 35696575 PMC9233301

[B13] Grewal D. S. (2018). Bell's palsy-tertiary ischemia: An etiological factor in residual facial palsy. Indian J. Otolaryngol. Head. Neck Surg. 70, 374–379. 10.1007/s12070-018-1381-9 30211092 PMC6127045

[B14] Holland N. J. Bernstein J. M. (2014). Bell's palsy. BMJ Clin. Evid. 2014, 1204. PMC3980711 24717284

[B15] Humphrey J. D. (2021). Mechanisms of vascular remodeling in hypertension. Am. J. Hypertens. 34, 432–441. 10.1093/ajh/hpaa195 33245319 PMC8140657

[B16] Kim S. Y. Oh D. J. Park B. Choi H. G. (2020). Bell's palsy and obesity, alcohol consumption and smoking: A nested case-control study using a national health screening cohort. Sci. Rep. 10, 4248. 10.1038/s41598-020-61240-7 32144385 PMC7060281

[B17] Kınar A. Ulu Ş. Bucak A. Kazan E. (2021). Can Systemic Immune-Inflammation Index (SII) be a prognostic factor of Bell's palsy patients? Neurol. Sci. 42, 3197–3201. 10.1007/s10072-020-04921-5 33237492

[B18] Lek M. Karczewski K. J. Minikel E. V. Samocha K. E. Banks E. Fennell T. (2016). Analysis of protein-coding genetic variation in 60,706 humans. Nature 536, 285–291. 10.1038/nature19057 27535533 PMC5018207

[B19] Liu X. Fu Y. X. (2015). Exploring population size changes using SNP frequency spectra. Nat. Genet. 47, 555–559. 10.1038/ng.3254 25848749 PMC4414822

[B20] Navarese E. P. Robinson J. G. Kowalewski M. Kolodziejczak M. Andreotti F. Bliden K. (2018). Association between baseline LDL-C level and total and cardiovascular mortality after LDL-C lowering: A systematic review and meta-analysis. JAMA 319, 1566–1579. 10.1001/jama.2018.2525 29677301 PMC5933331

[B21] Psillas G. Dimas G. G. Sarafidou A. Didangelos T. Perifanis V. Kaiafa G. (2021). Evaluation of effects of diabetes mellitus, hypercholesterolemia and hypertension on Bell's palsy. J. Clin. Med. 10, 2357. 10.3390/jcm10112357 34072018 PMC8198958

[B22] Ravnskov U. de Lorgeril M. Diamond D. M. Hama R. Hamazaki T. Hammarskjöld B. (2018). LDL-C does not cause cardiovascular disease: A comprehensive review of the current literature. Expert Rev. Clin. Pharmacol. 11, 959–970. 10.1080/17512433.2018.1519391 30198808

[B23] Richardson T. G. Sanderson E. Palmer T. M. Ala-Korpela M. Ference B. A. Davey Smith G. (2020). Evaluating the relationship between circulating lipoprotein lipids and apolipoproteins with risk of coronary heart disease: A multivariable mendelian randomisation analysis. PLoS Med. 17, e1003062. 10.1371/journal.pmed.1003062 32203549 PMC7089422

[B24] Sanderson E. (2021). Multivariable mendelian randomization and mediation. Cold Spring Harb. Perspect. Med. 11, a038984. 10.1101/cshperspect.a038984 32341063 PMC7849347

[B25] Savadi-Oskouei D. Abedi A. Sadeghi-Bazargani H. (2008). Independent role of hypertension in Bell's palsy: A case-control study. Eur. Neurol. 60, 253–257. 10.1159/000151701 18756090

[B26] Sniderman A. Langlois M. Cobbaert C. (2021). Update on apolipoprotein B. Curr. Opin. Lipidol. 32, 226–230. 10.1097/MOL.0000000000000754 33870931

[B27] Stamatiou I. Papachristou S. Papanas N. (2022). Carpal tunnel syndrome in diabetes mellitus. Curr. Diabetes Rev. 18, e010921196025. 10.2174/1573399817666210901114610 34468300

[B28] Tseng C. C. Hu L. Y. Liu M. E. Yang A. C. Shen C. C. Tsai S. J. (2017). Bidirectional association between Bell's palsy and anxiety disorders: A nationwide population-based retrospective cohort study. J. Affect Disord. 215, 269–273. 10.1016/j.jad.2017.03.051 28359982

[B29] Vakharia K. Vakharia K. (2016). Bell's palsy. Facial Plast. Surg. Clin. North Am. 24, 1–10. 10.1016/j.fsc.2015.08.001 26611696

[B30] Xue A. Wu Y. Zhu Z. Zhang F. Kemper K. E. Zheng Z. (2018). Genome-wide association analyses identify 143 risk variants and putative regulatory mechanisms for type 2 diabetes. Nat. Commun. 9, 2941. 10.1038/s41467-018-04951-w 30054458 PMC6063971

[B31] Yamamoto K. Kurioka T. Ohki M. Ohashi K. Harada Y. Asako Y. (2022). Immune-nutritional status as a novel prognostic predictor of Bell's palsy. Audiol. Neurootol 27, 418–426. 10.1159/000524355 35512660

[B32] Yengo L. Sidorenko J. Kemper K. E. Zheng Z. Wood A. R. Weedon M. N. (2018). Meta-analysis of genome-wide association studies for height and body mass index in ∼700000 individuals of European ancestry. Hum. Mol. Genet. 27, 3641–3649. 10.1093/hmg/ddy271 30124842 PMC6488973

[B33] Zheng J. Baird D. Borges M. C. Bowden J. Hemani G. Haycock P. (2017). Recent developments in mendelian randomization studies. Curr. Epidemiol. Rep. 4, 330–345. 10.1007/s40471-017-0128-6 29226067 PMC5711966

