# Supplementary material for: Genetic association of hypertension and several other metabolic disorders with Bell’s palsy
Source: Front Genet. 2023 Jul 18;14:1077438. doi: 10.3389/fgene.2023.1077438 (PMC10391645; doi:10.3389/fgene.2023.1077438)
Supplement: Supplementary file 1 [file DataSheet2.docx]

A


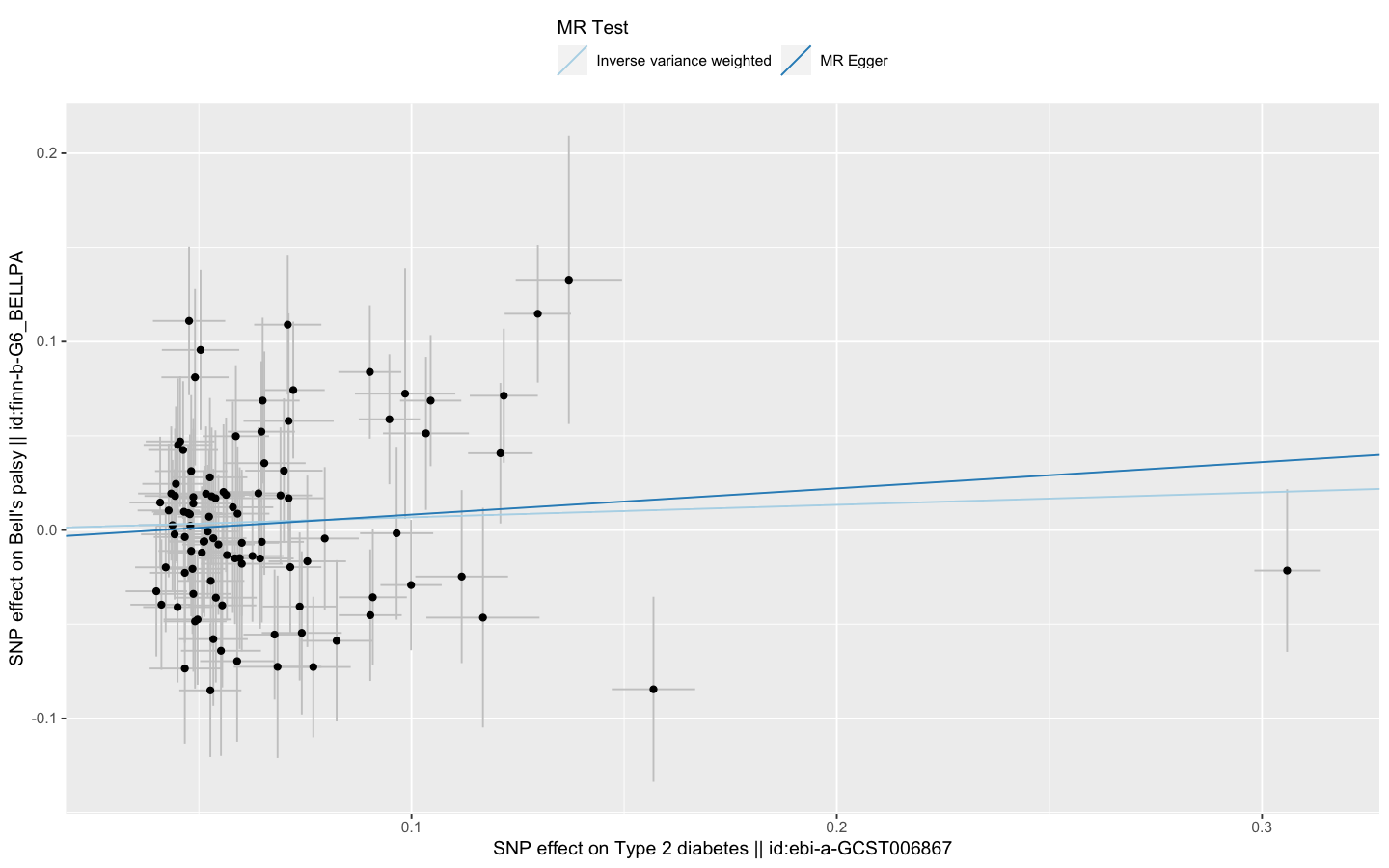


B


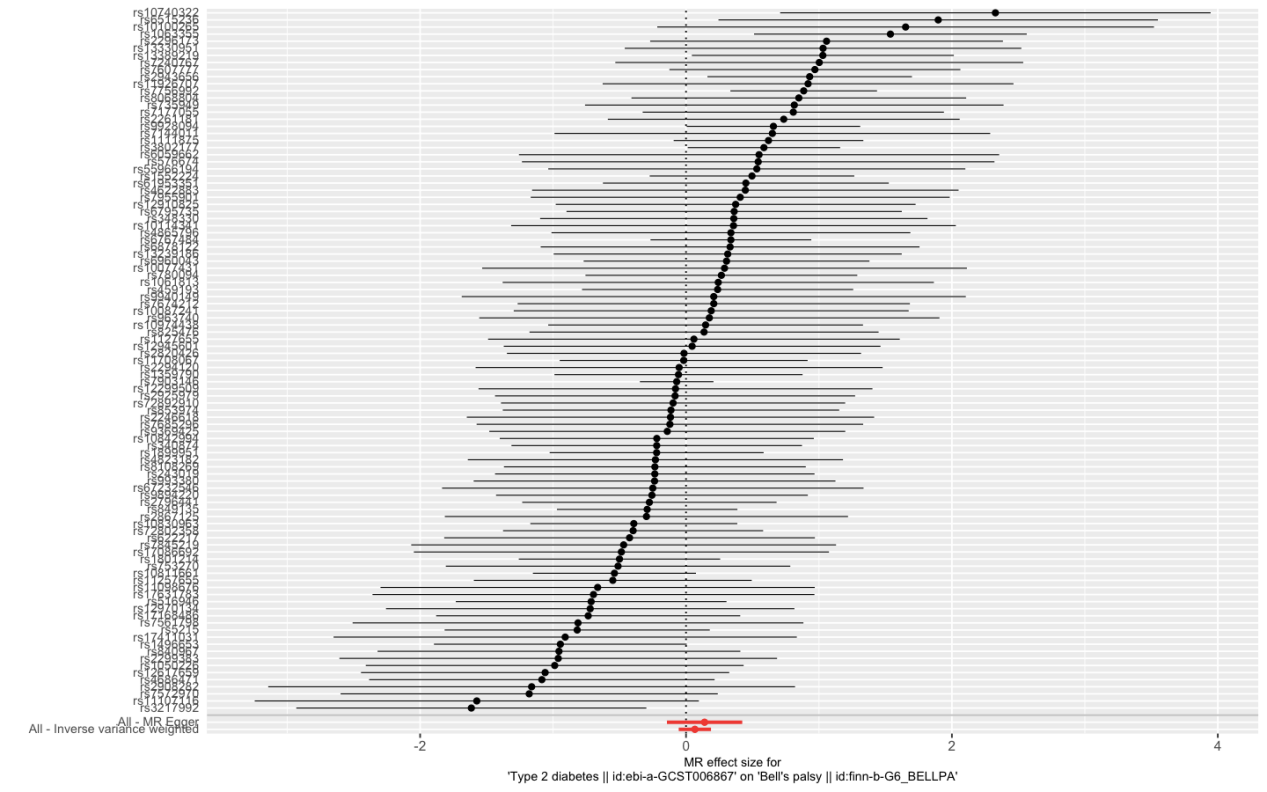


C


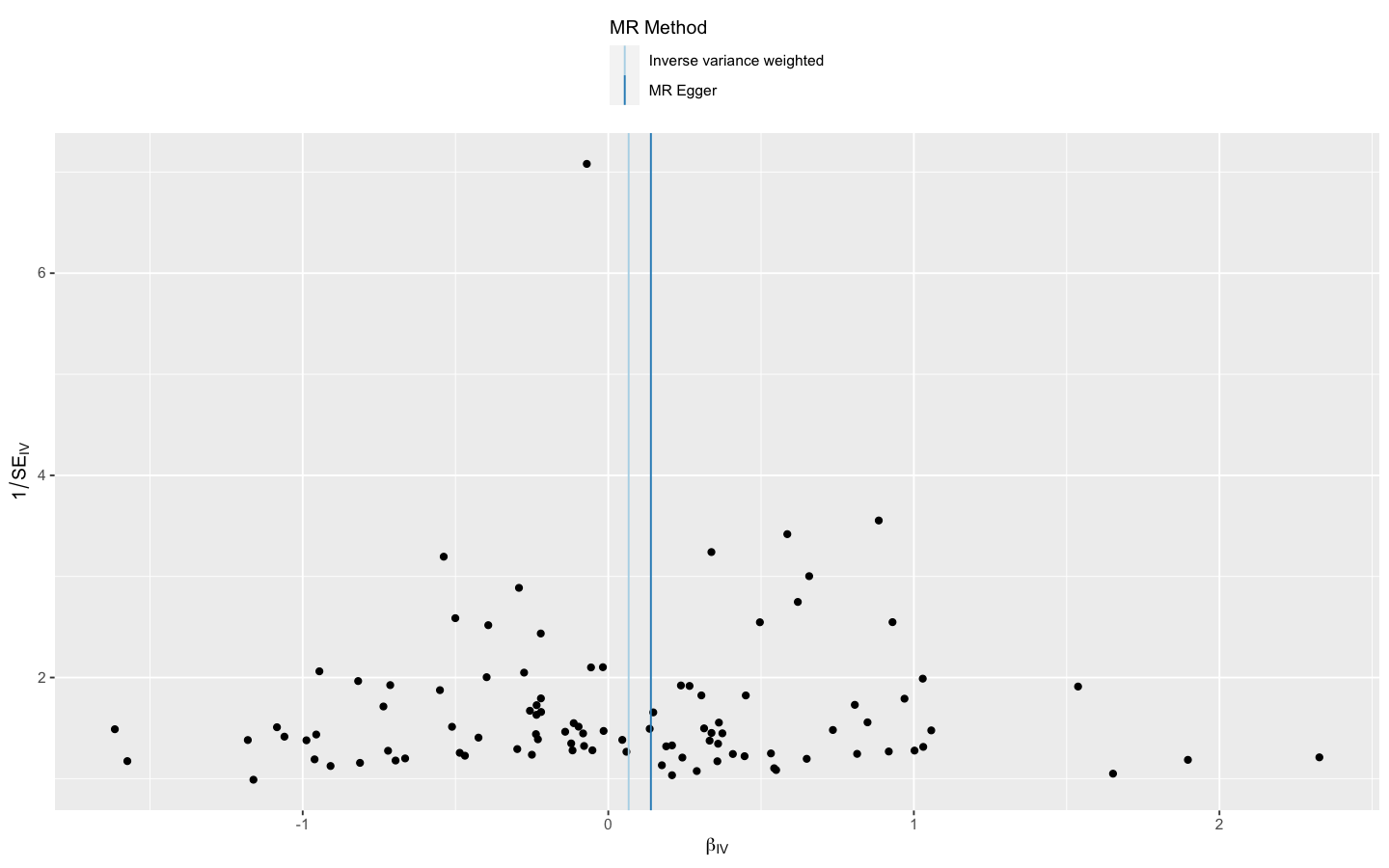


D


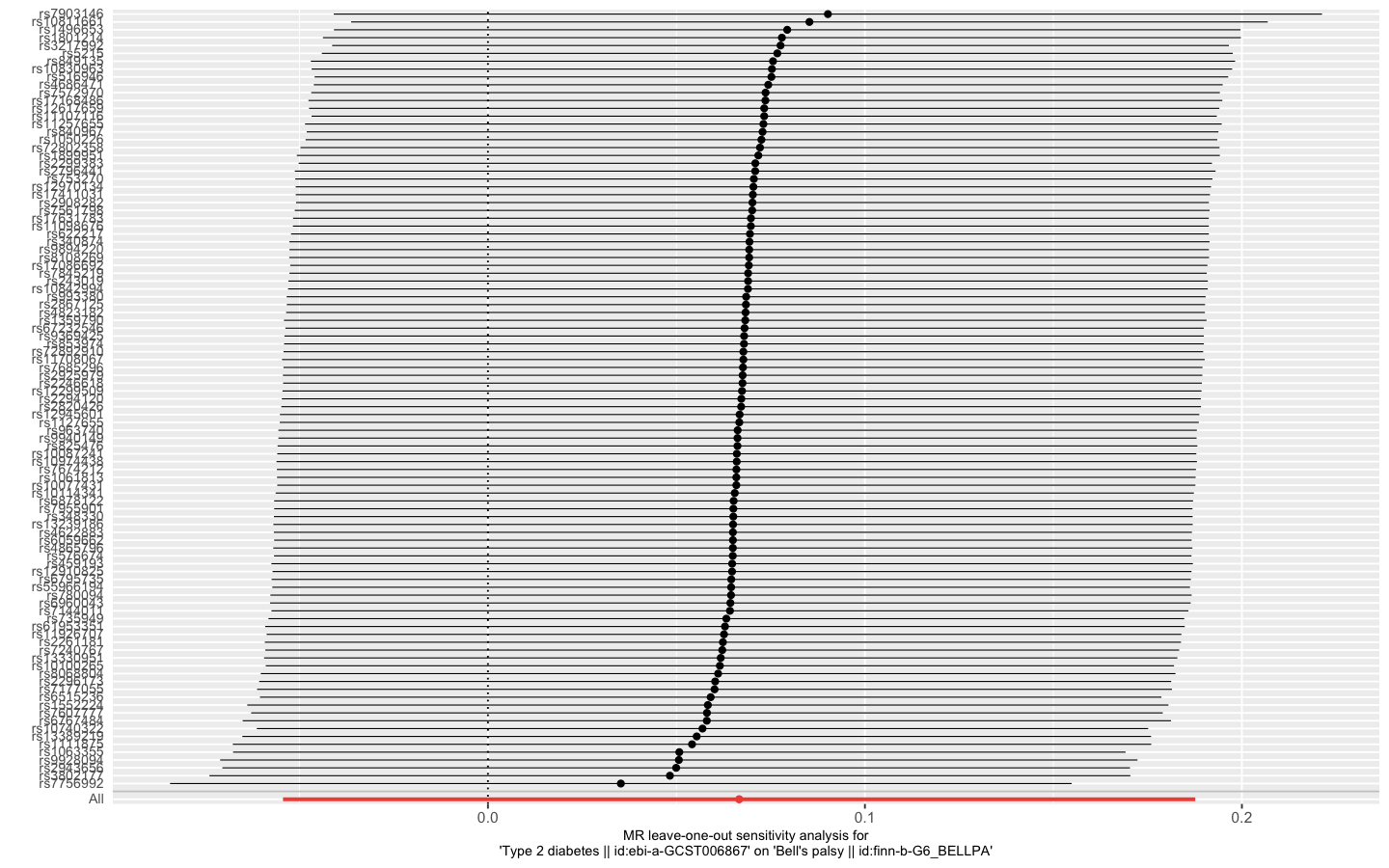


**Supplementary Figure 1 Effect of type 2 diabetes on Bell's palsy risk**

Figure 1A: scatter plot; Figure 1B: forest plot; Figure 1C: funnel plot;

Figure 1D: “leave-one-SNP-out” analysis.

A


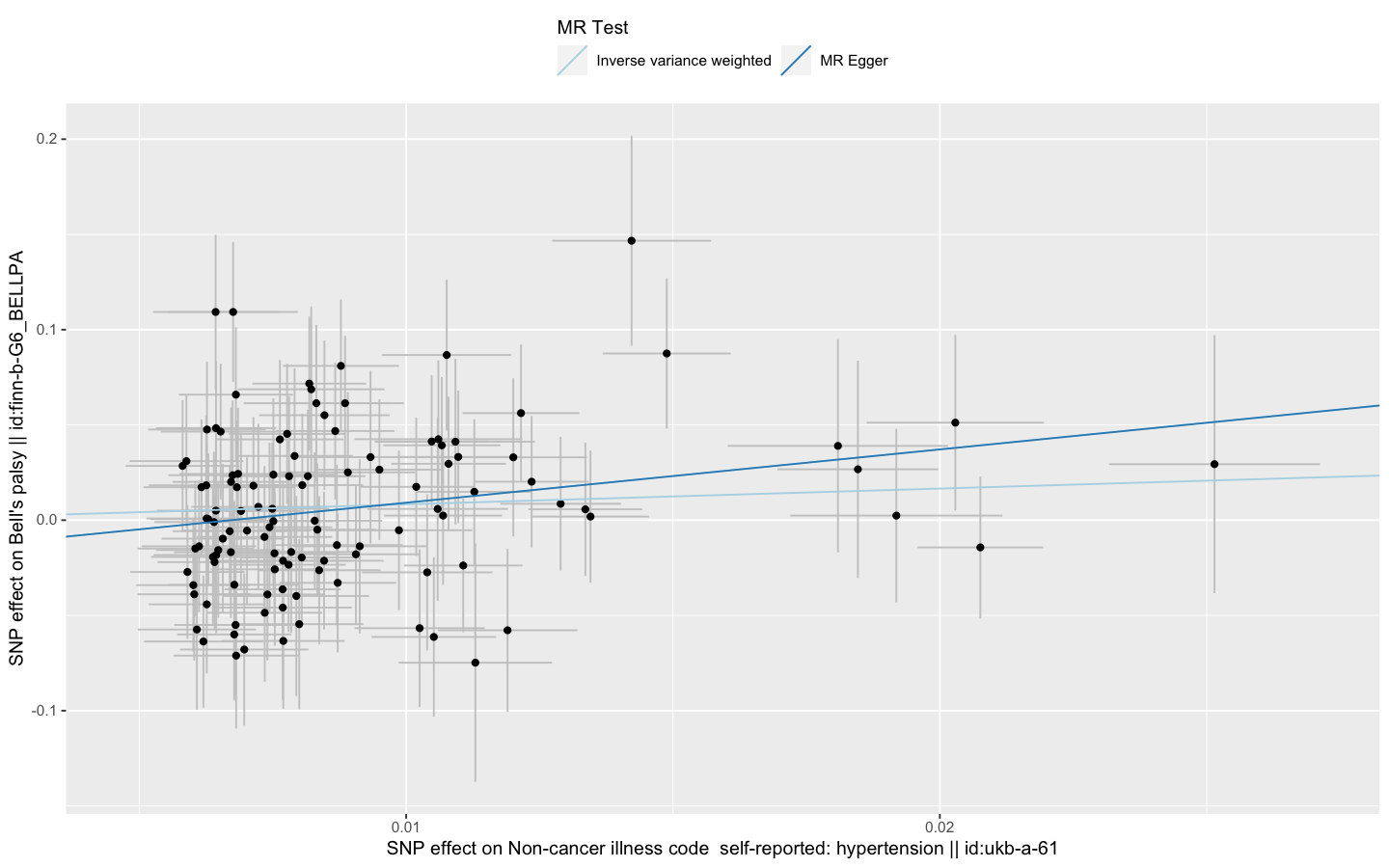


B


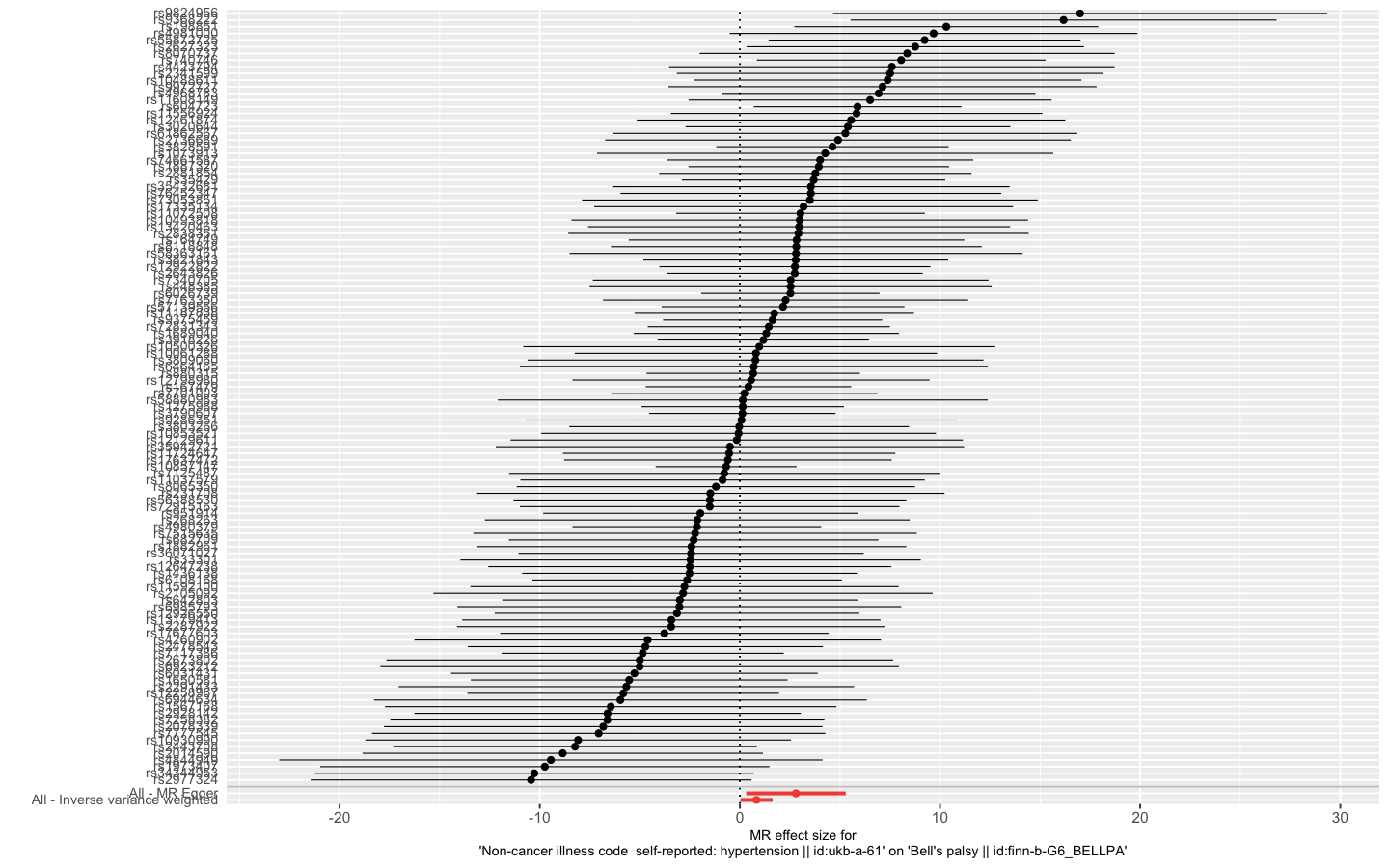


C


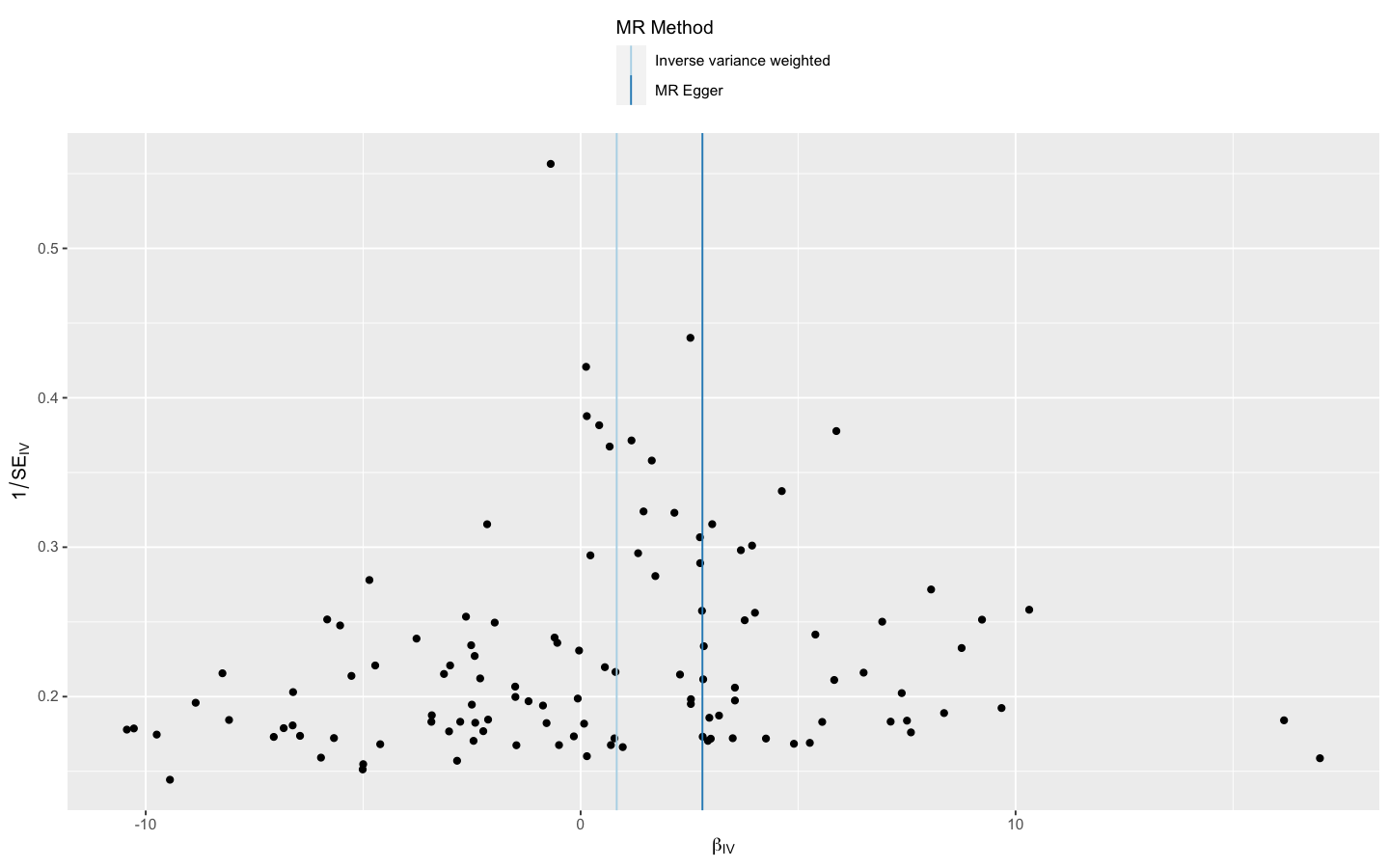


D


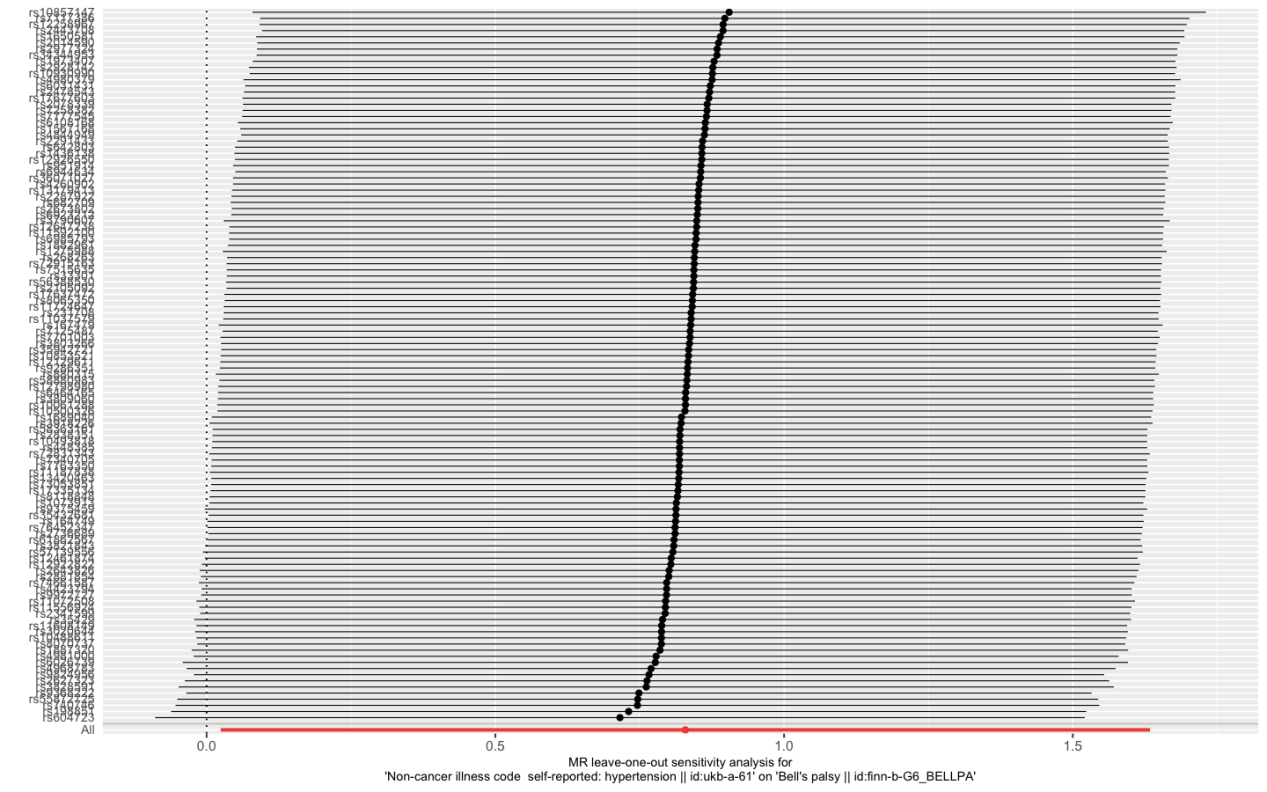


**Supplementary Figure 2 Effect of hypertension on Bell's palsy risk (removing rs8027450）**

Figure 1A: scatter plot; Figure 1B: forest plot; Figure 1C: funnel plot;

Figure 1D: “leave-one-SNP-out” analysis.

A


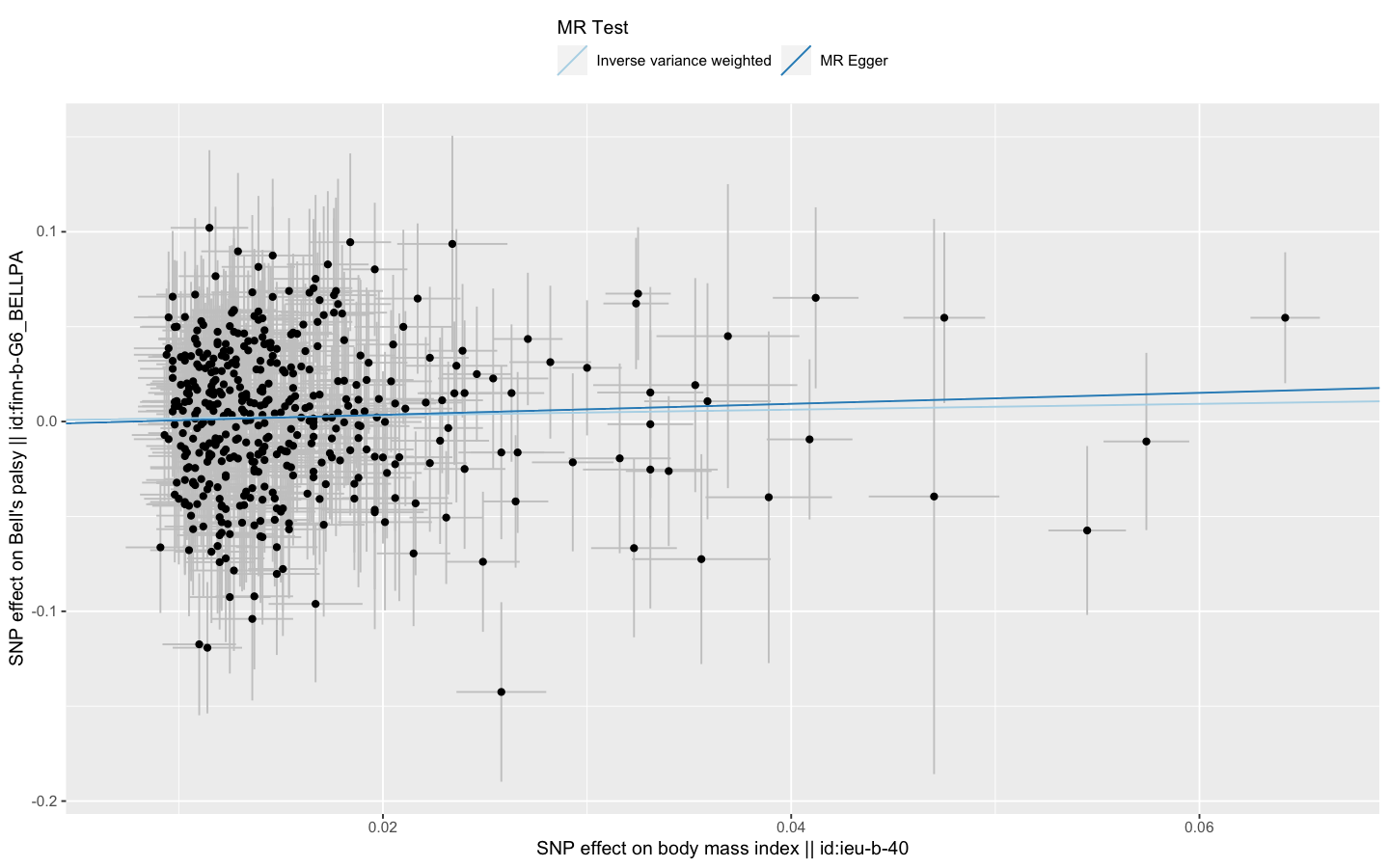


B


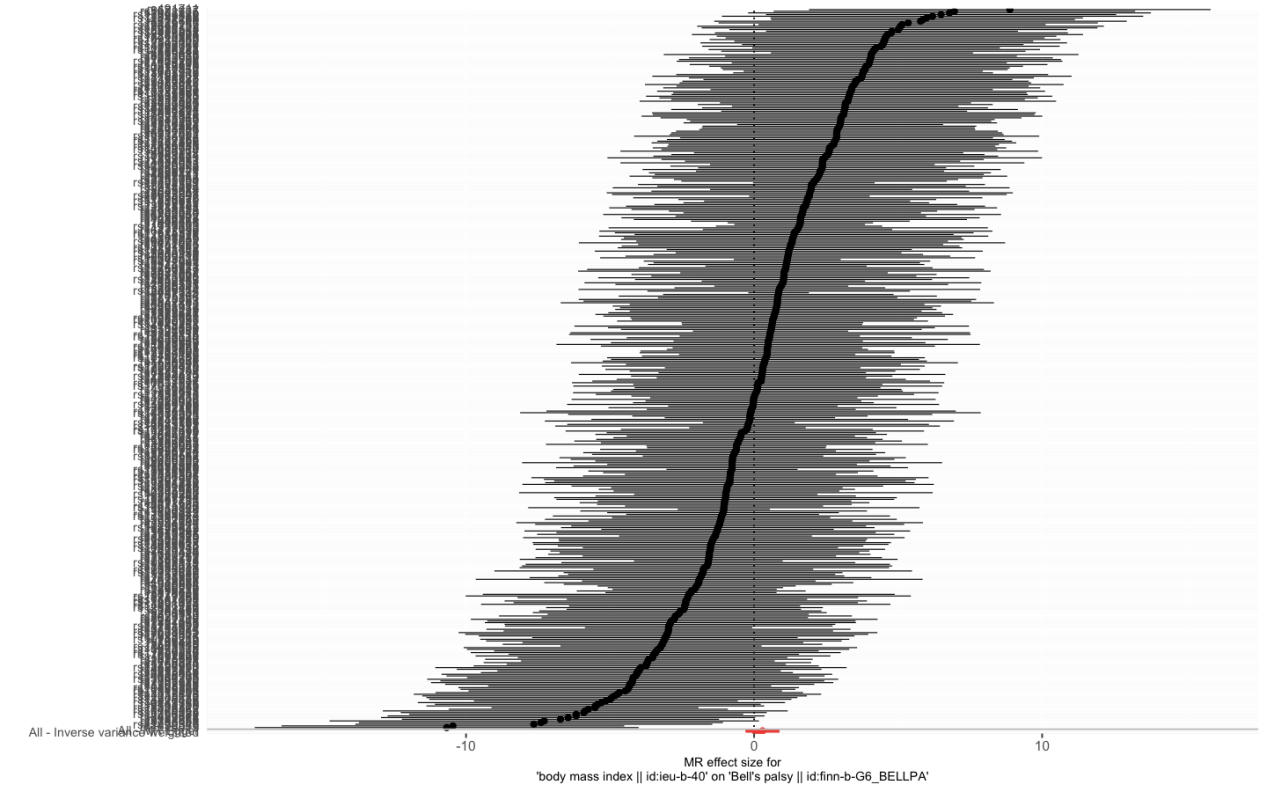


C


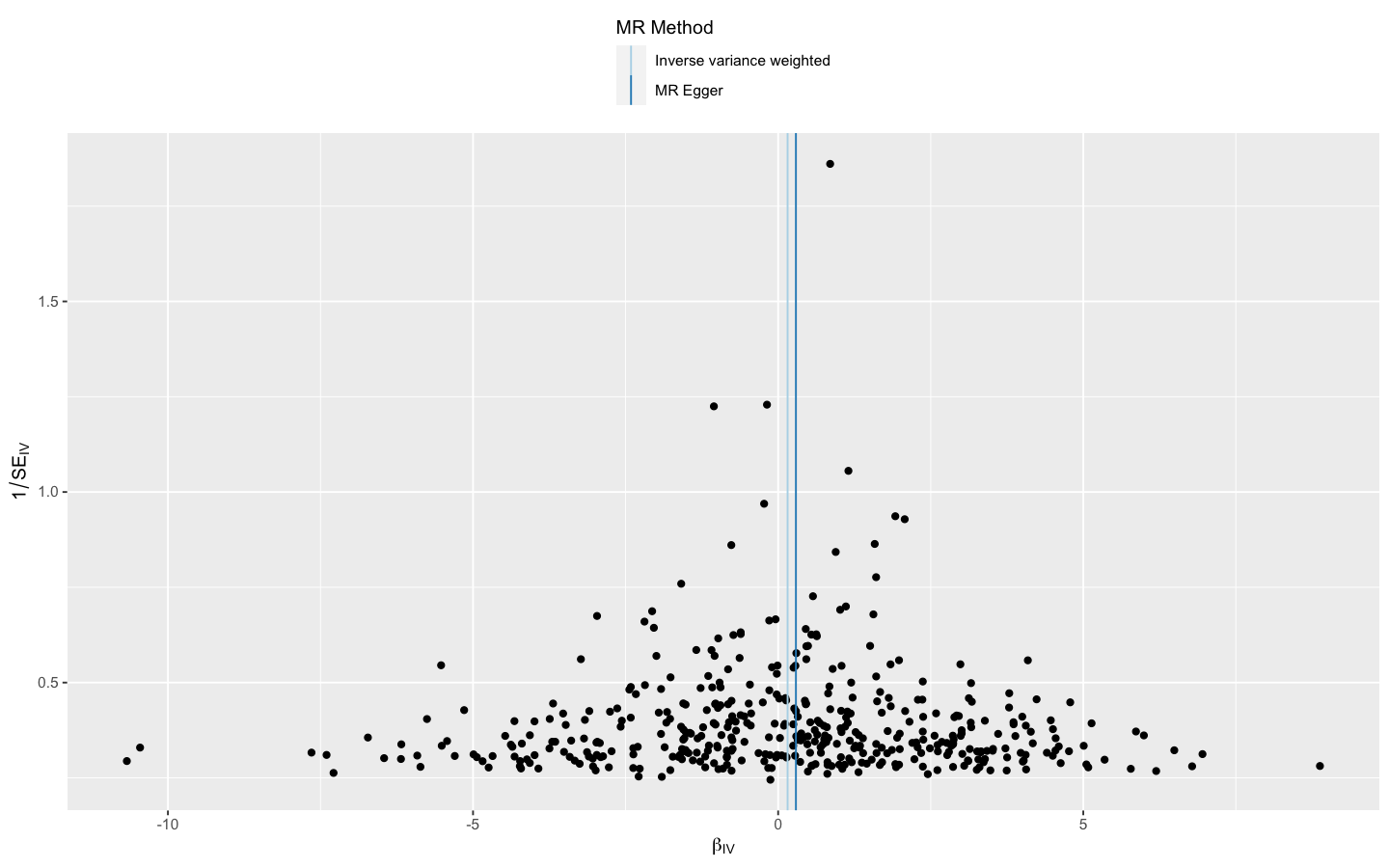


D


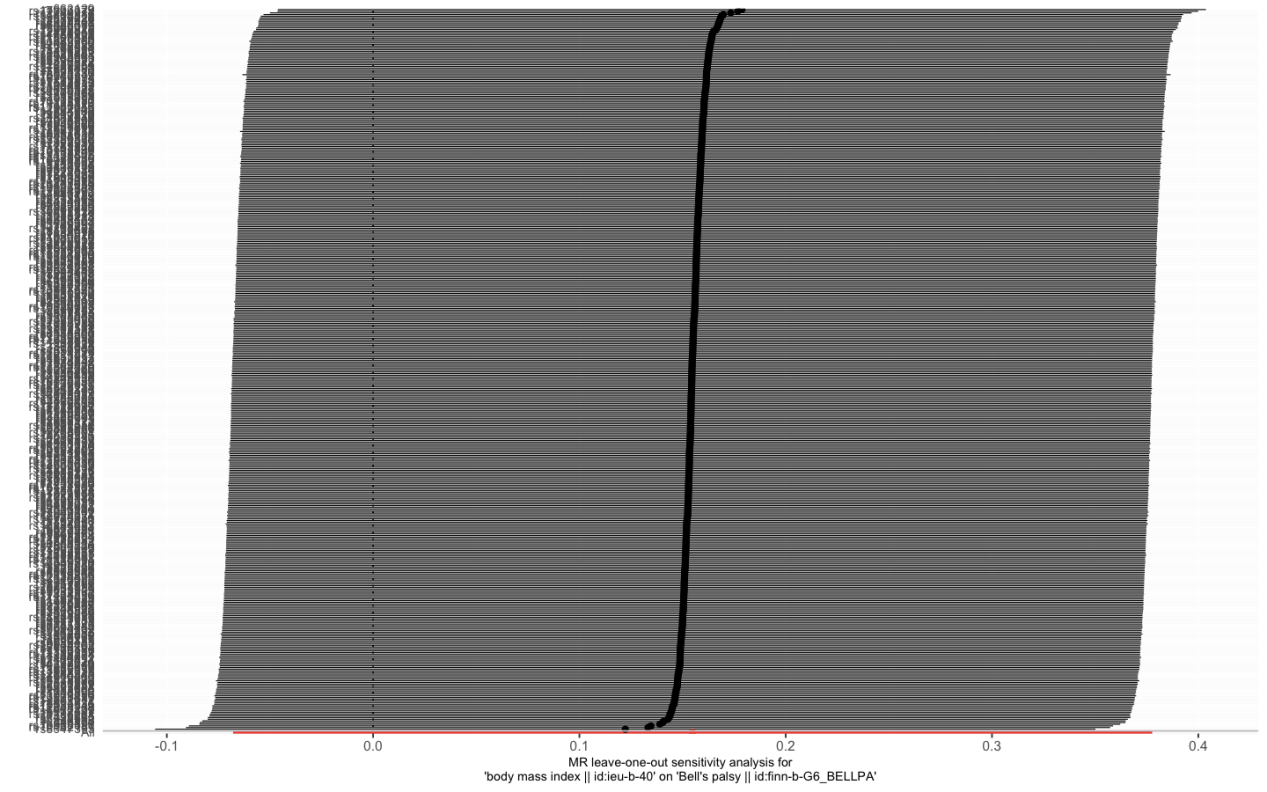


**Supplementary Figure 3 Effect of body mass index on Bell's palsy risk**

Figure 1A: scatter plot; Figure 1B: forest plot; Figure 1C: funnel plot;

Figure 1D: “leave-one-SNP-out” analysis.

A


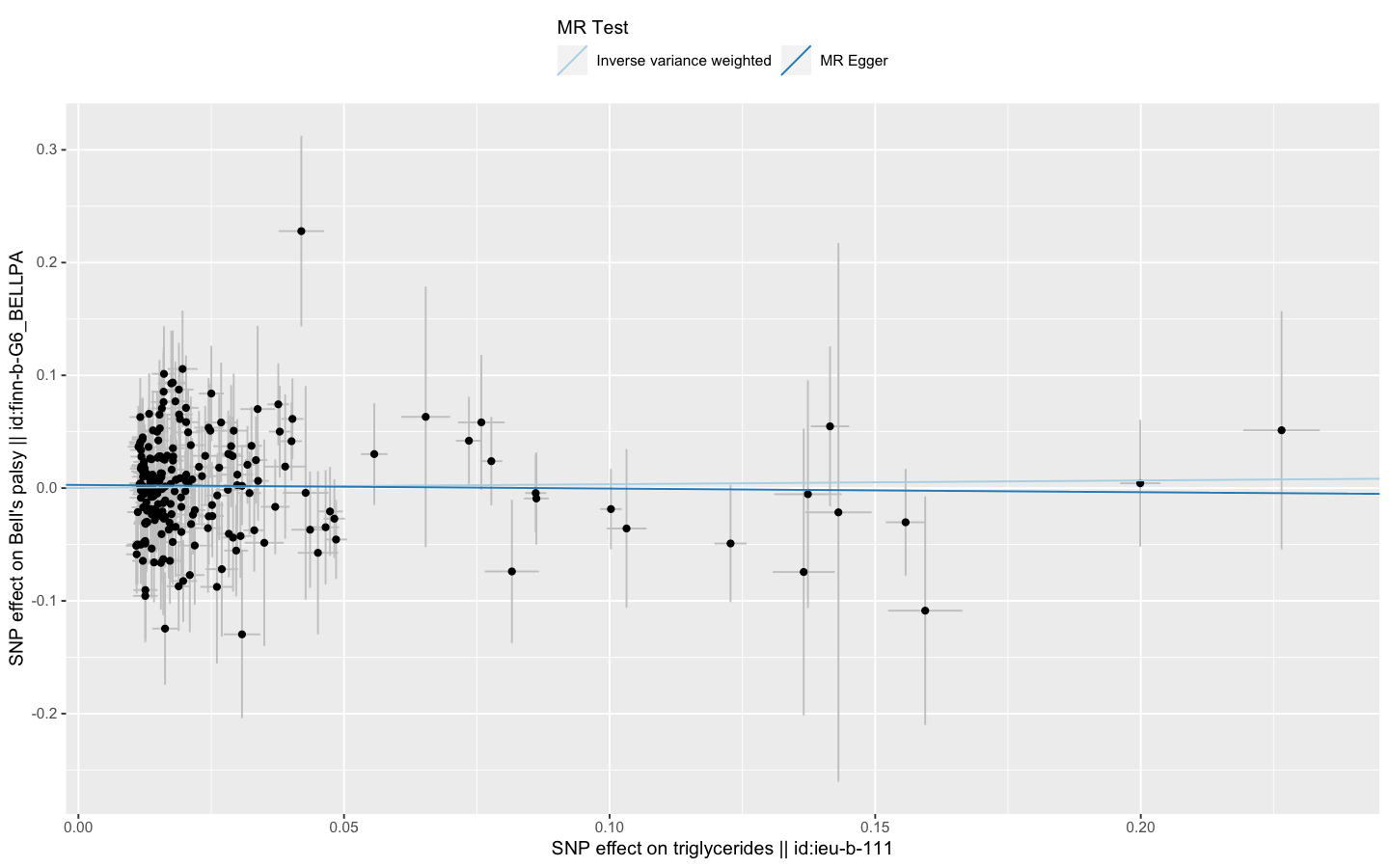


B


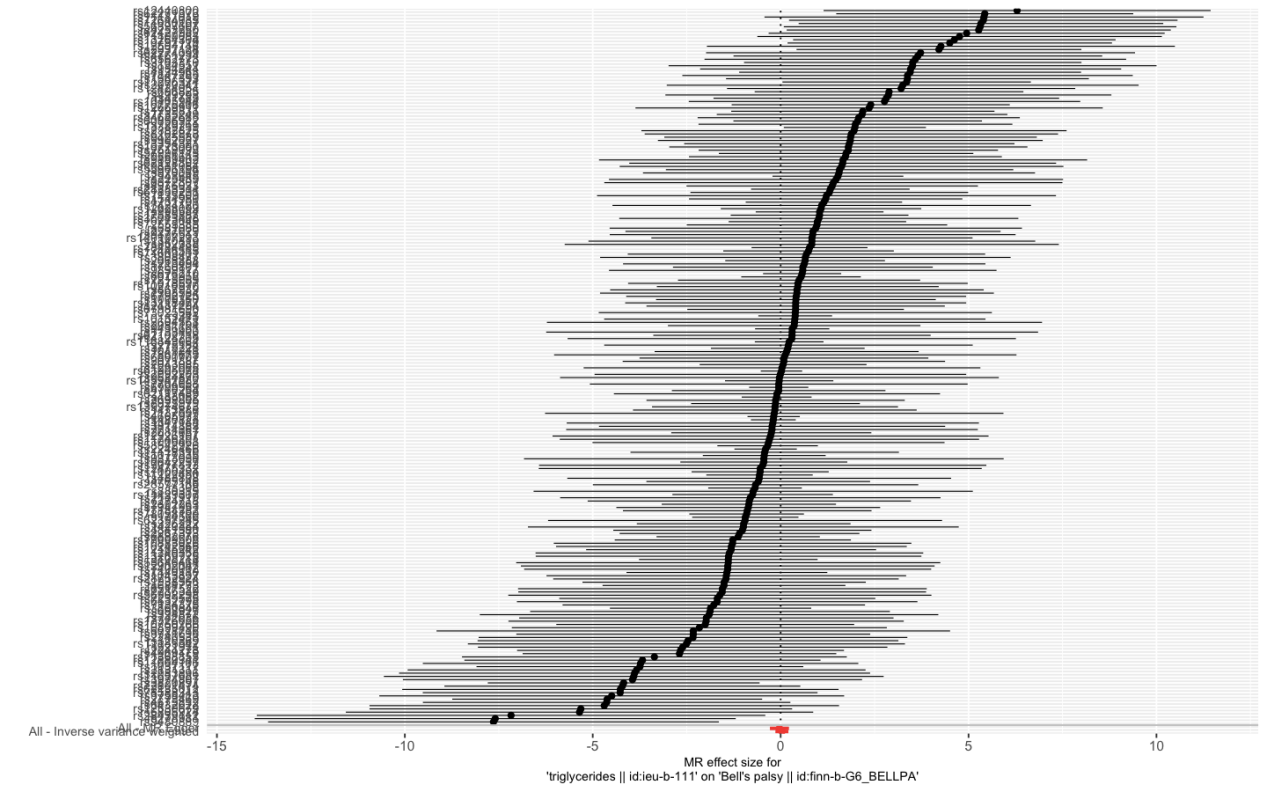


C


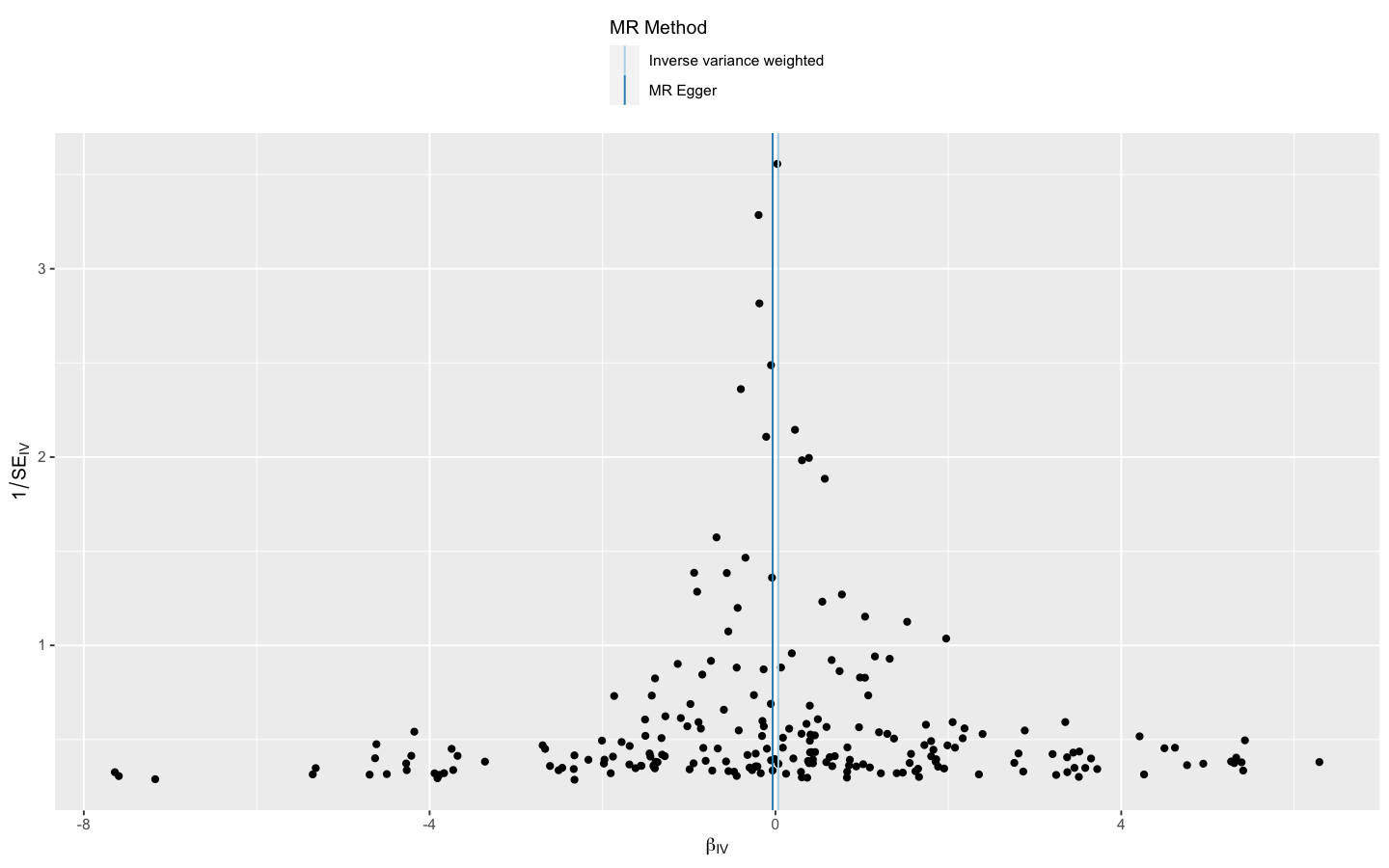


D


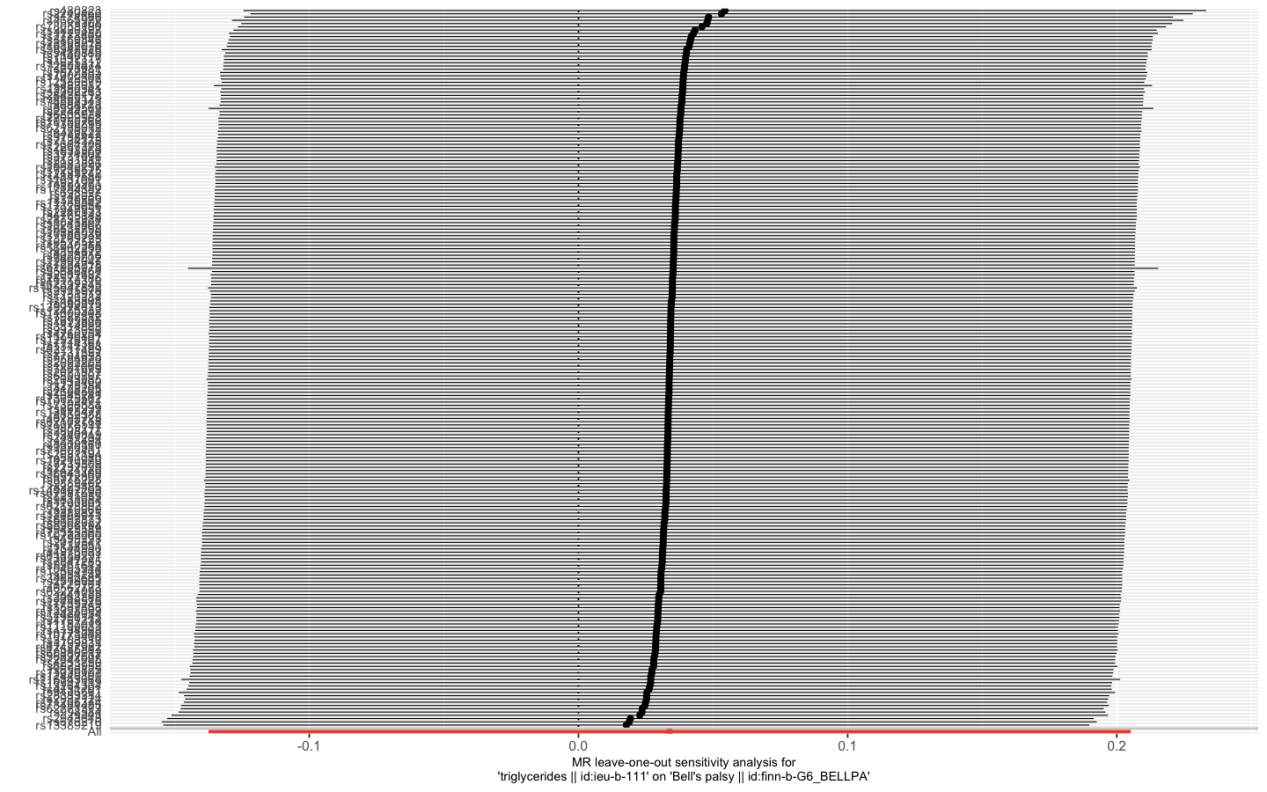


**Supplementary Figure 4 Effect of triglycerides on Bell's palsy risk**

Figure 1A: scatter plot; Figure 1B: forest plot; Figure 1C: funnel plot;

Figure 1D: “leave-one-SNP-out” analysis.

A


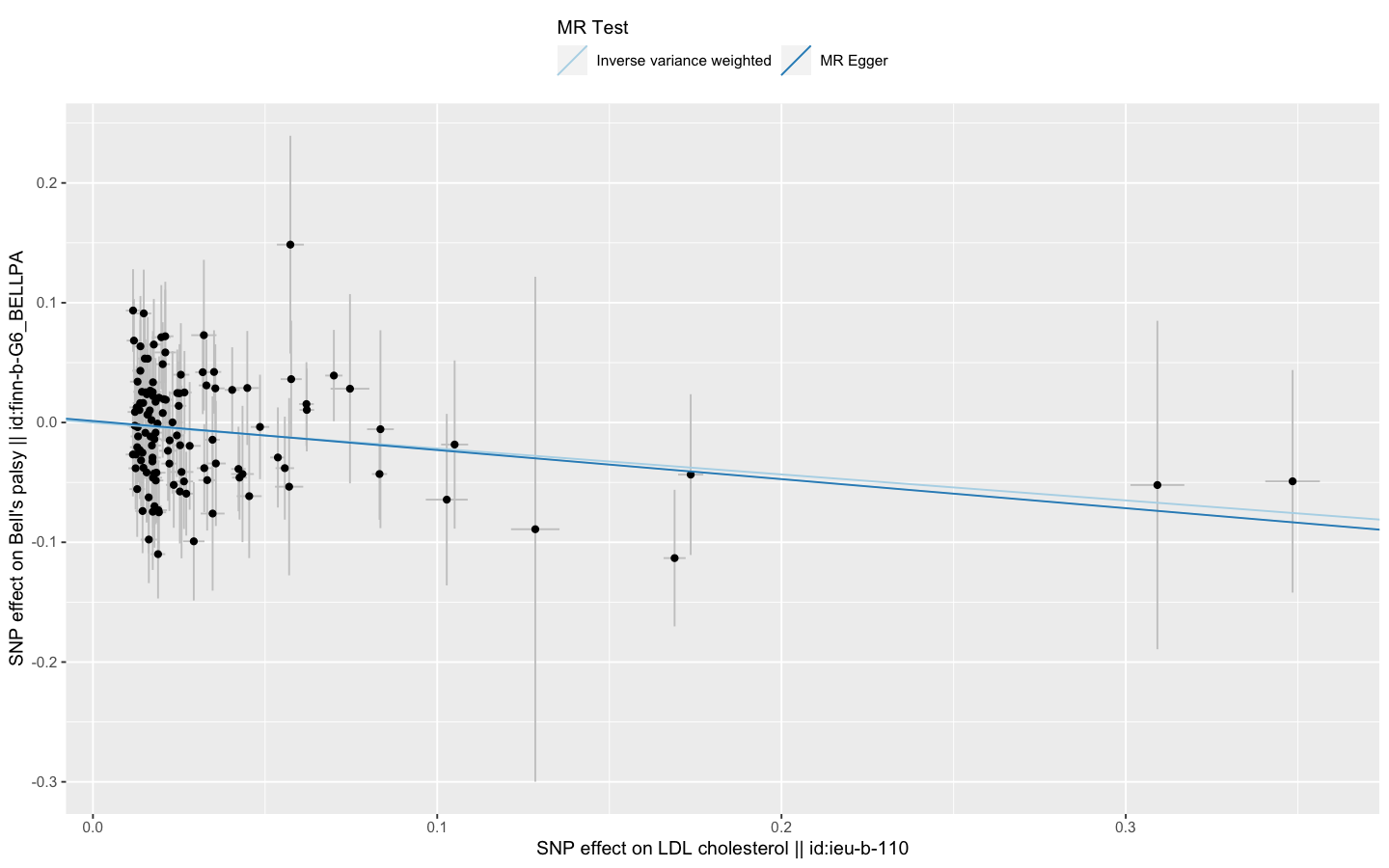


B


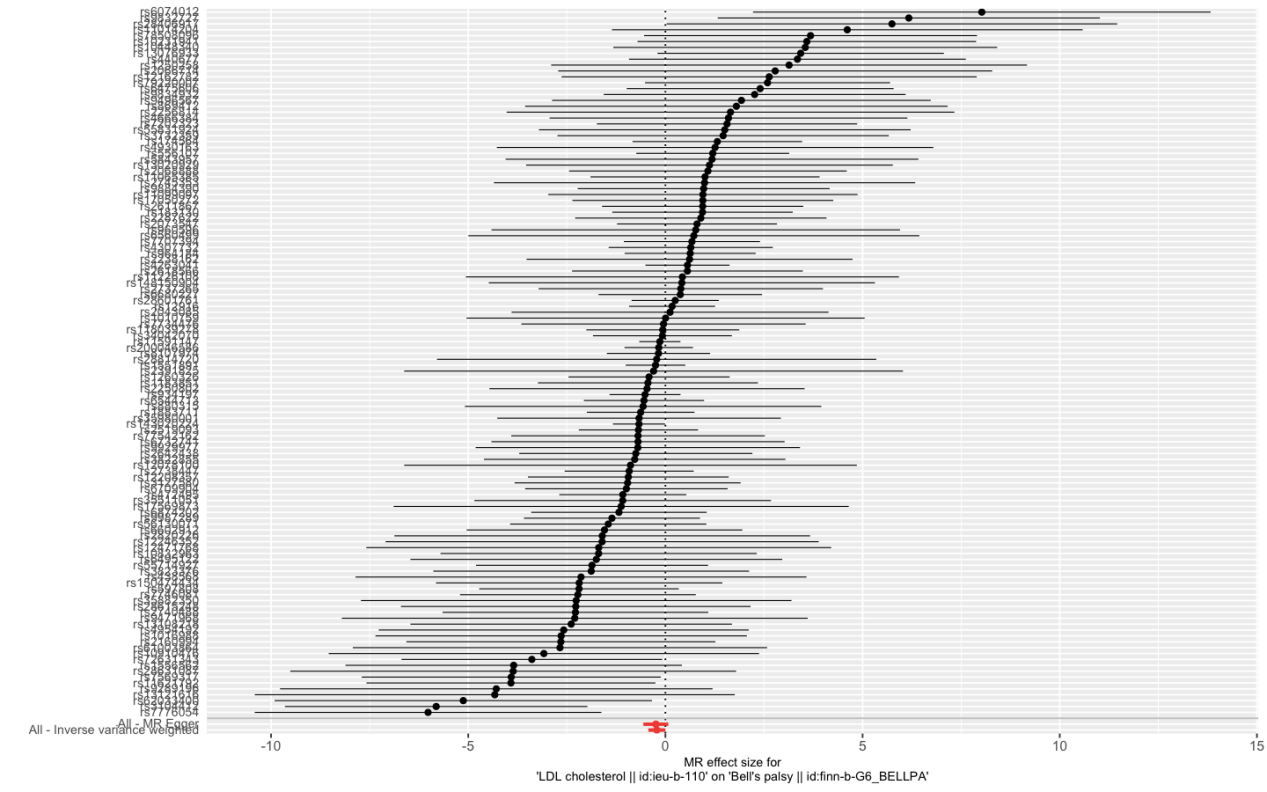


C


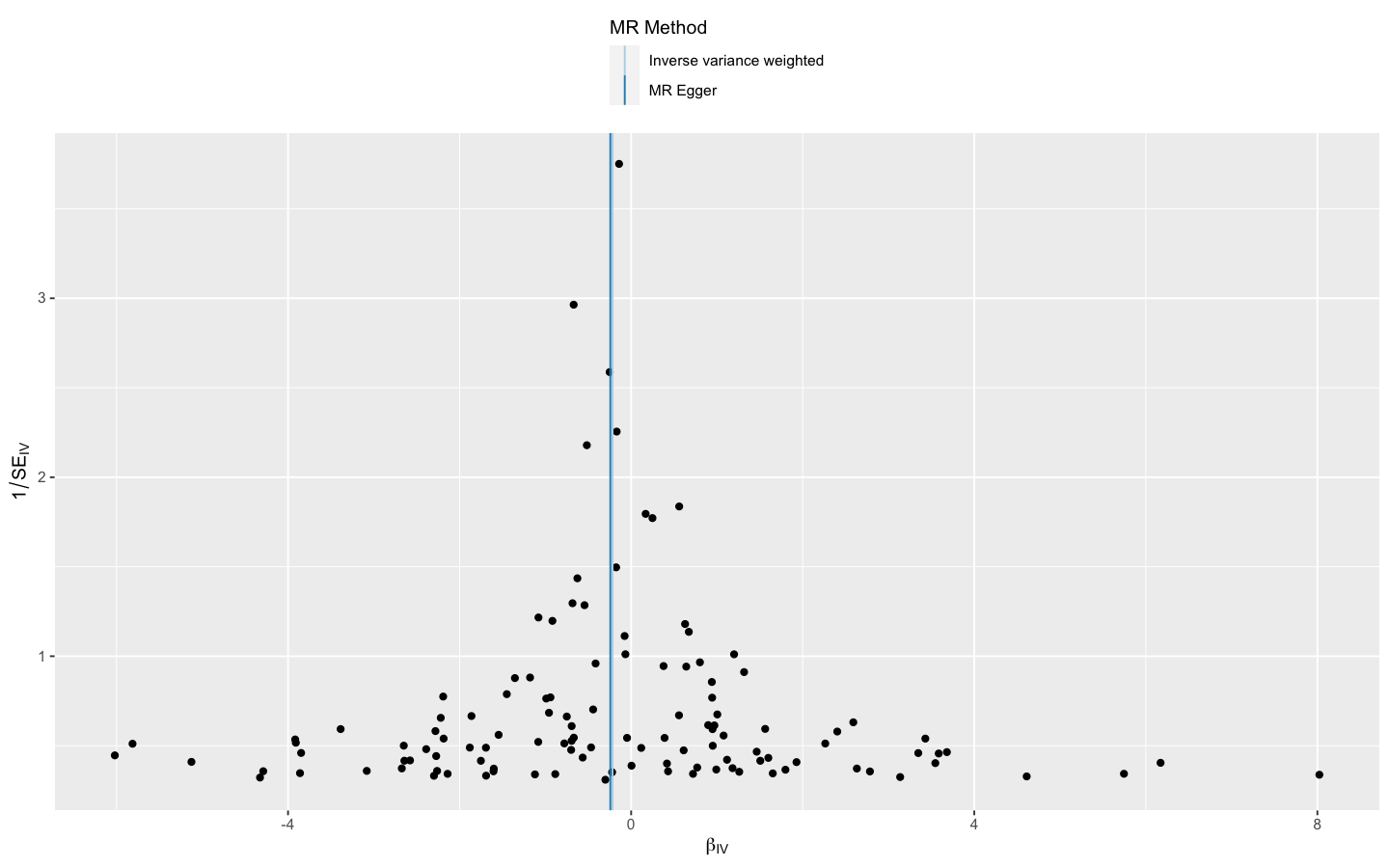


D


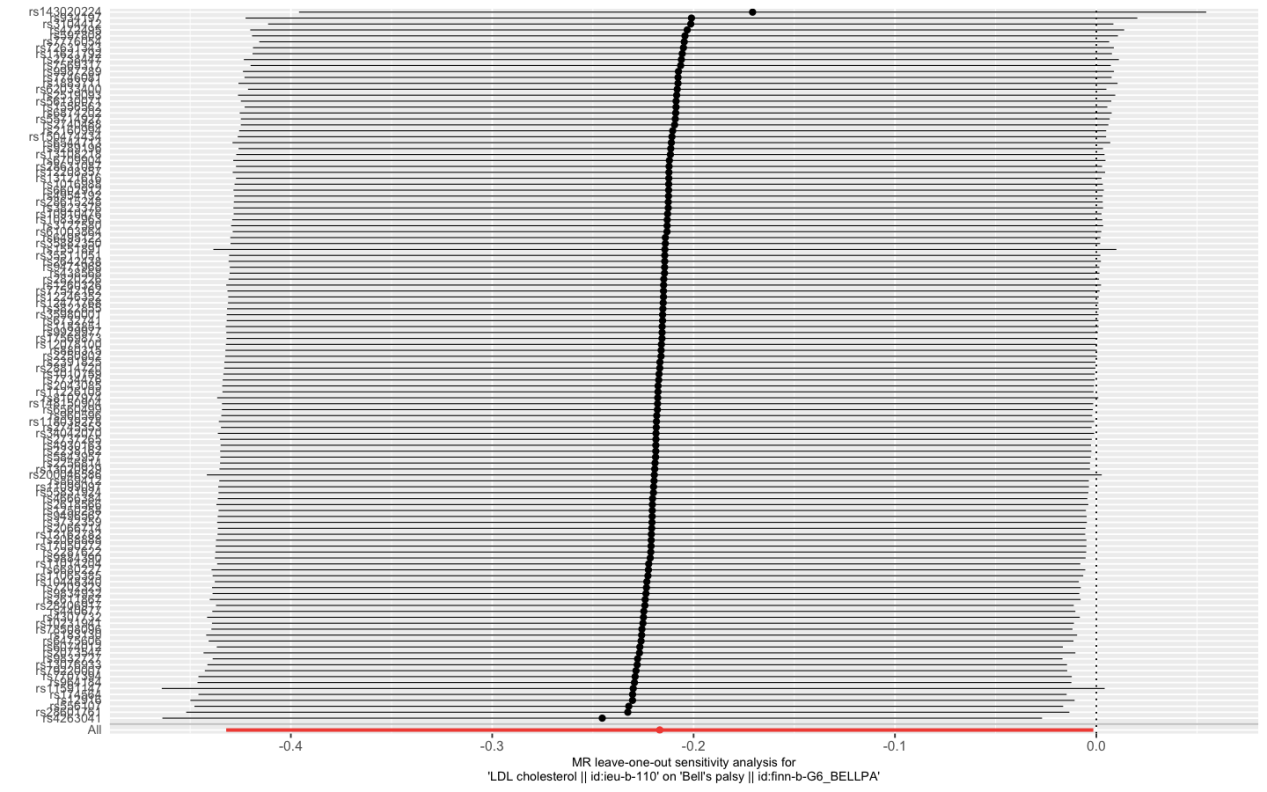


**Supplementary Figure 5 Effect of LDL cholesterol on Bell's palsy risk (removing rs497083)**

Figure 1A: scatter plot; Figure 1B: forest plot; Figure 1C: funnel plot;

Figure 1D: “leave-one-SNP-out” analysis.

A


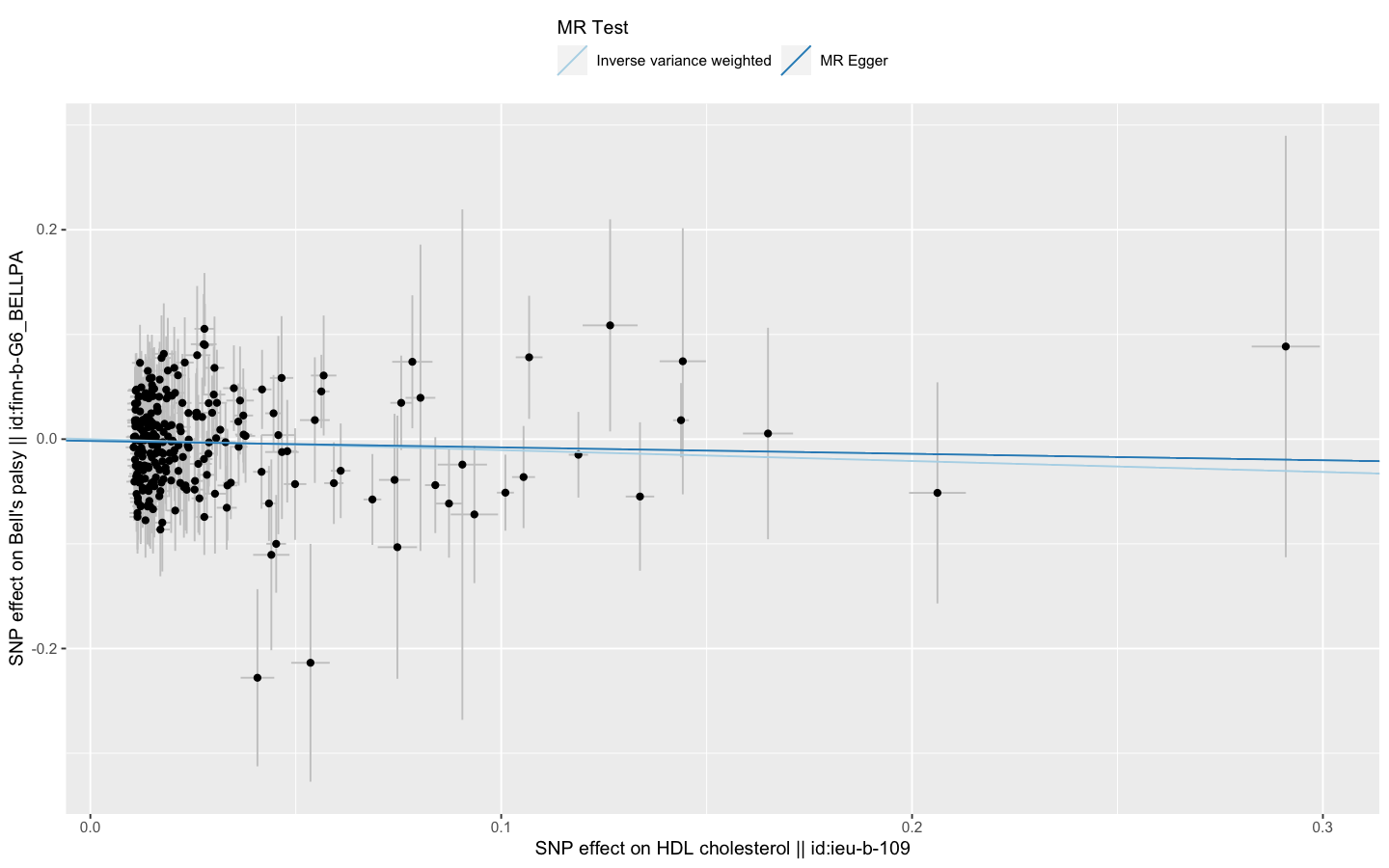


B


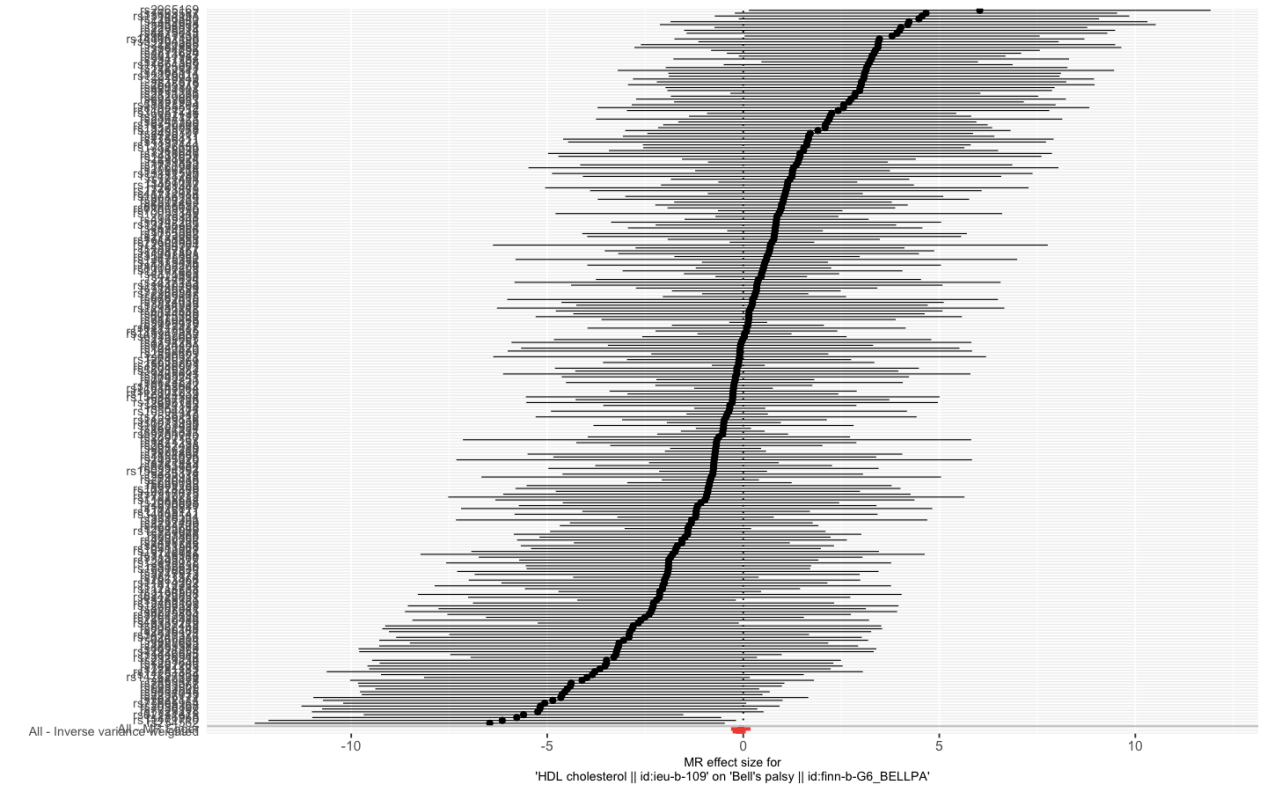


C


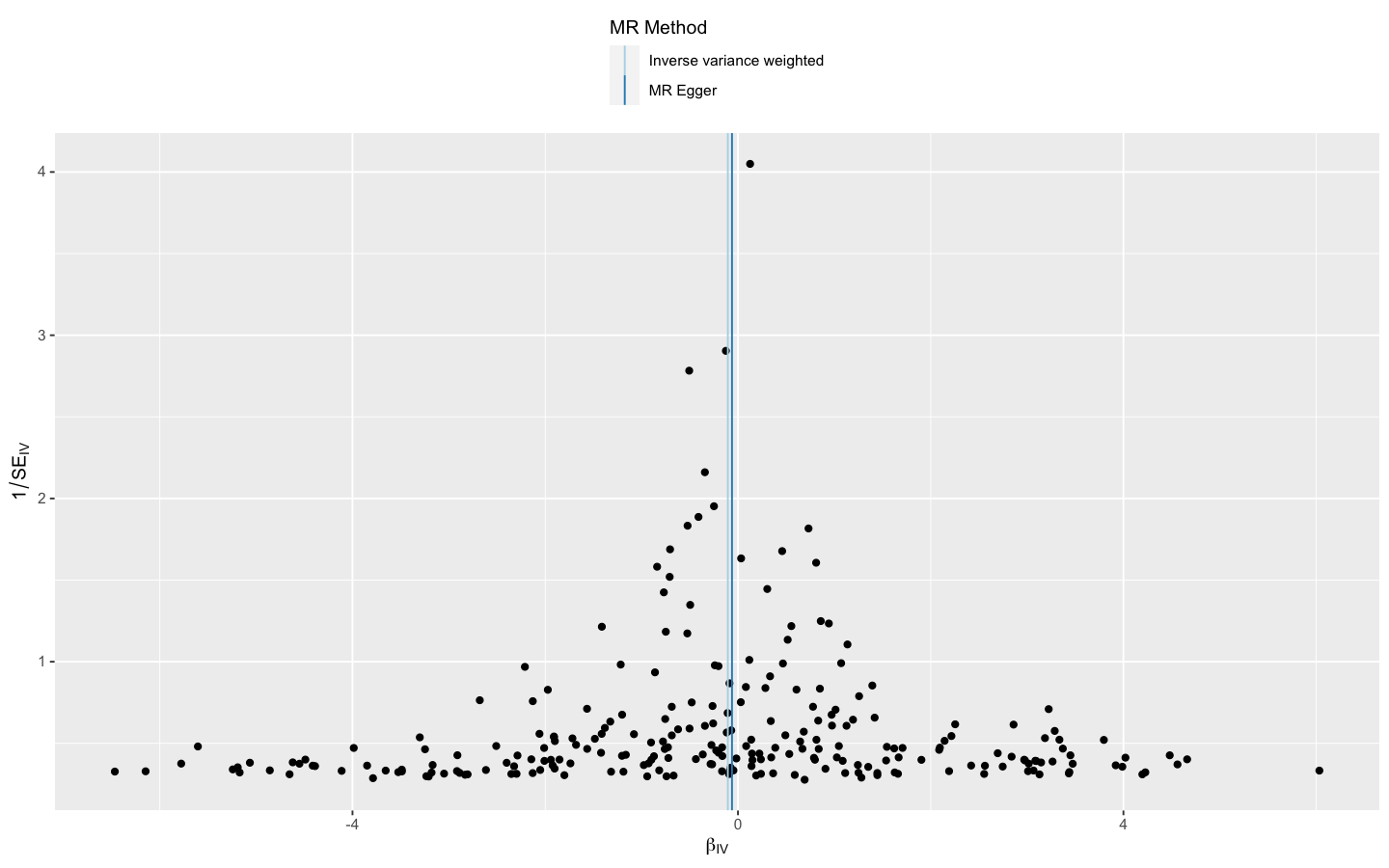


D


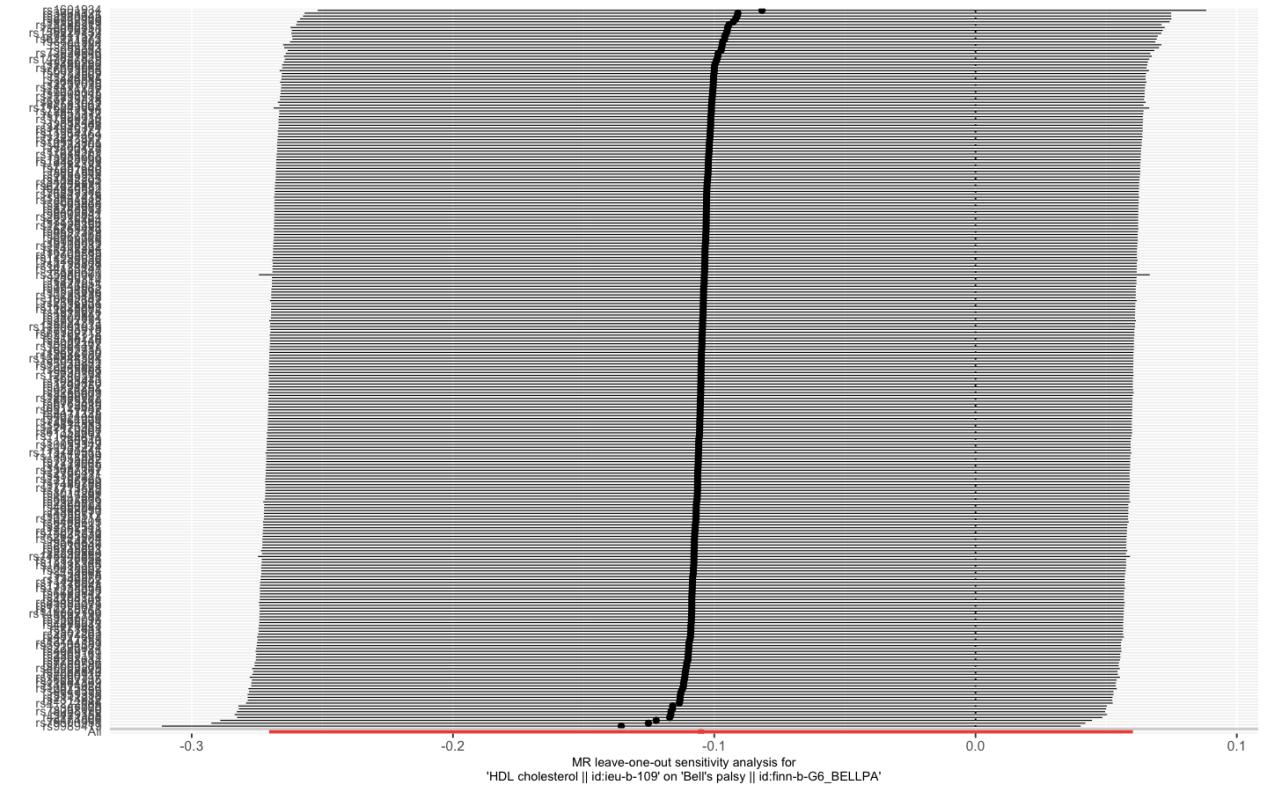


**Supplementary Figure 6 Effect of HDL cholesterol on Bell's palsy risk**

Figure 1A: scatter plot; Figure 1B: forest plot; Figure 1C: funnel plot;

Figure 1D: “leave-one-SNP-out” analysis.

A


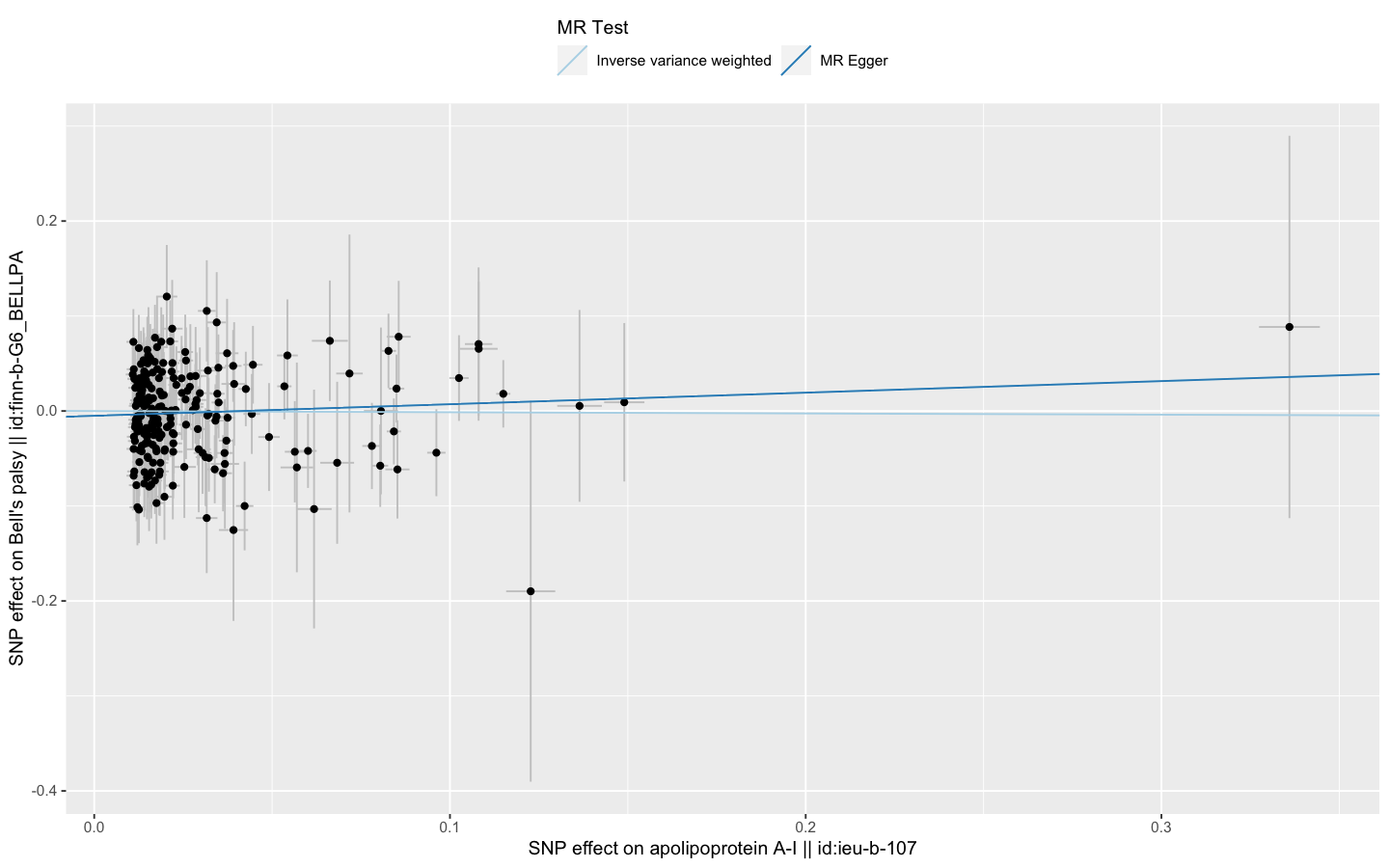


B


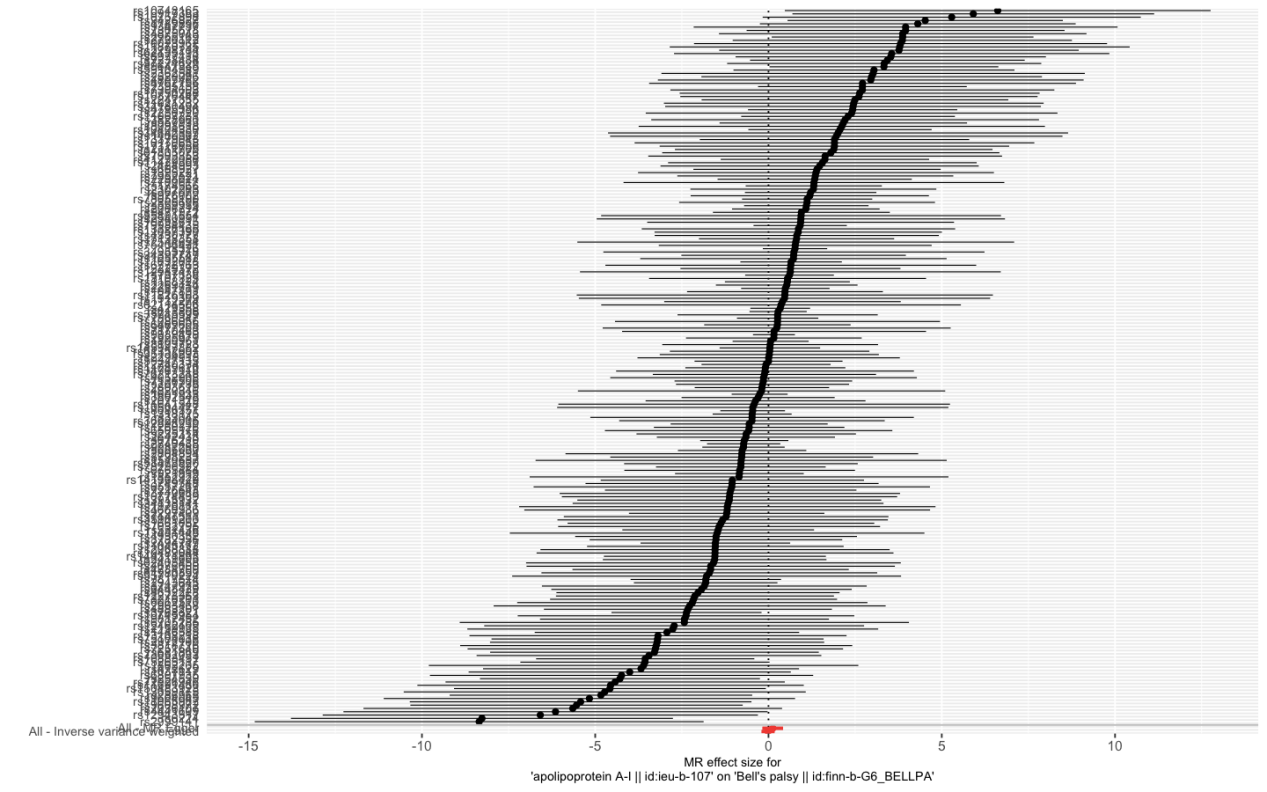


C


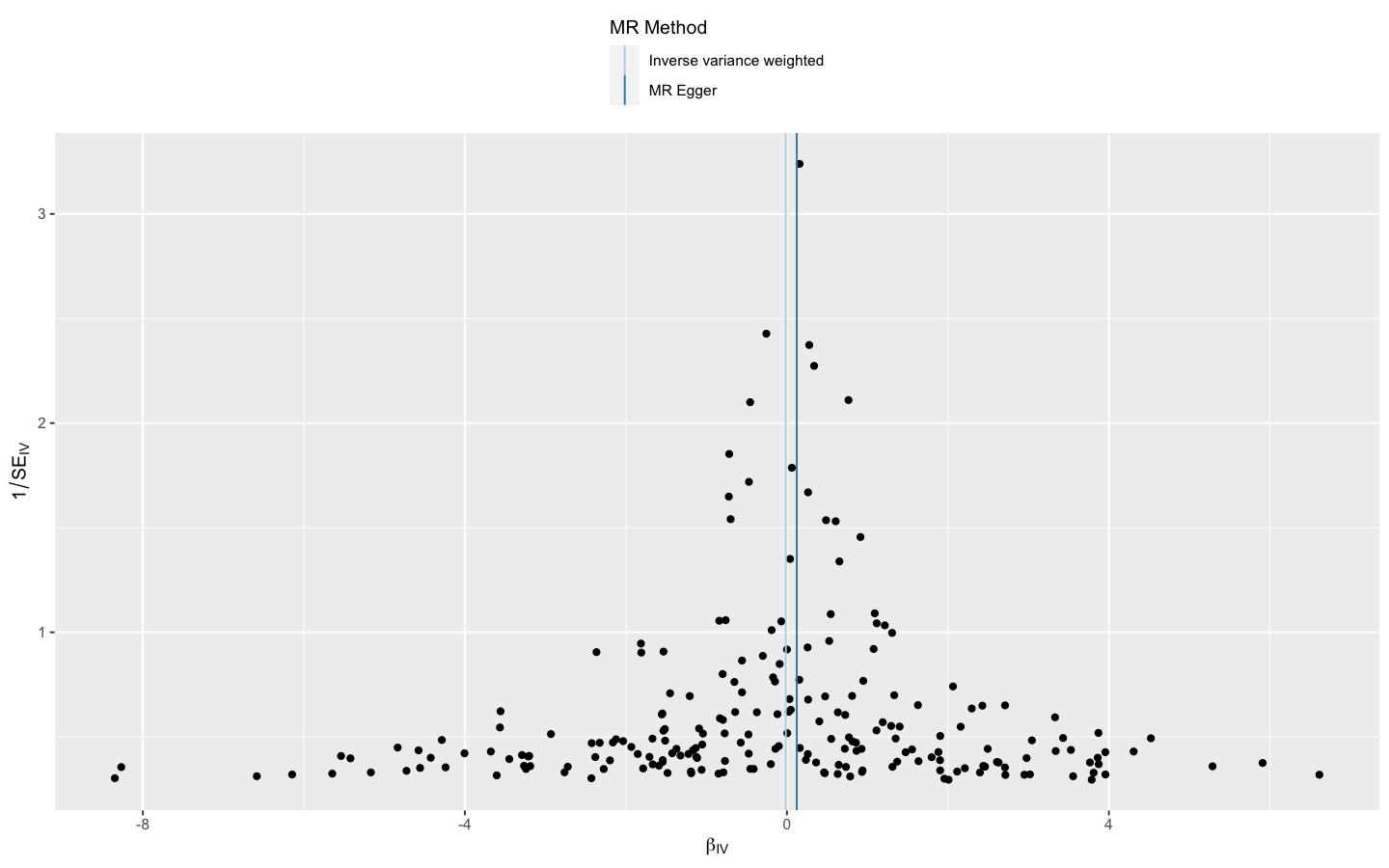


D


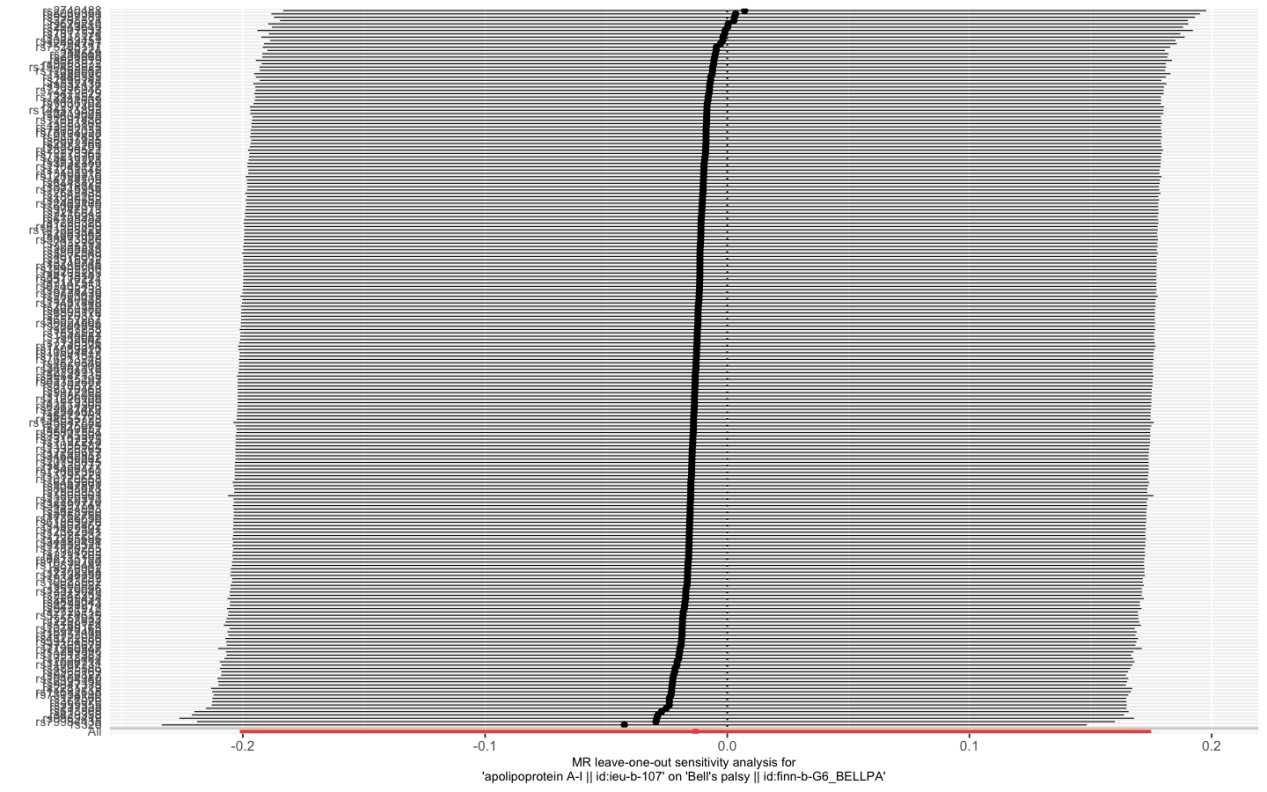


**Supplementary Figure 7 Effect of apolipoprotein A-I on Bell's palsy risk**

Figure 1A: scatter plot; Figure 1B: forest plot; Figure 1C: funnel plot;

Figure 1D: “leave-one-SNP-out” analysis.

A


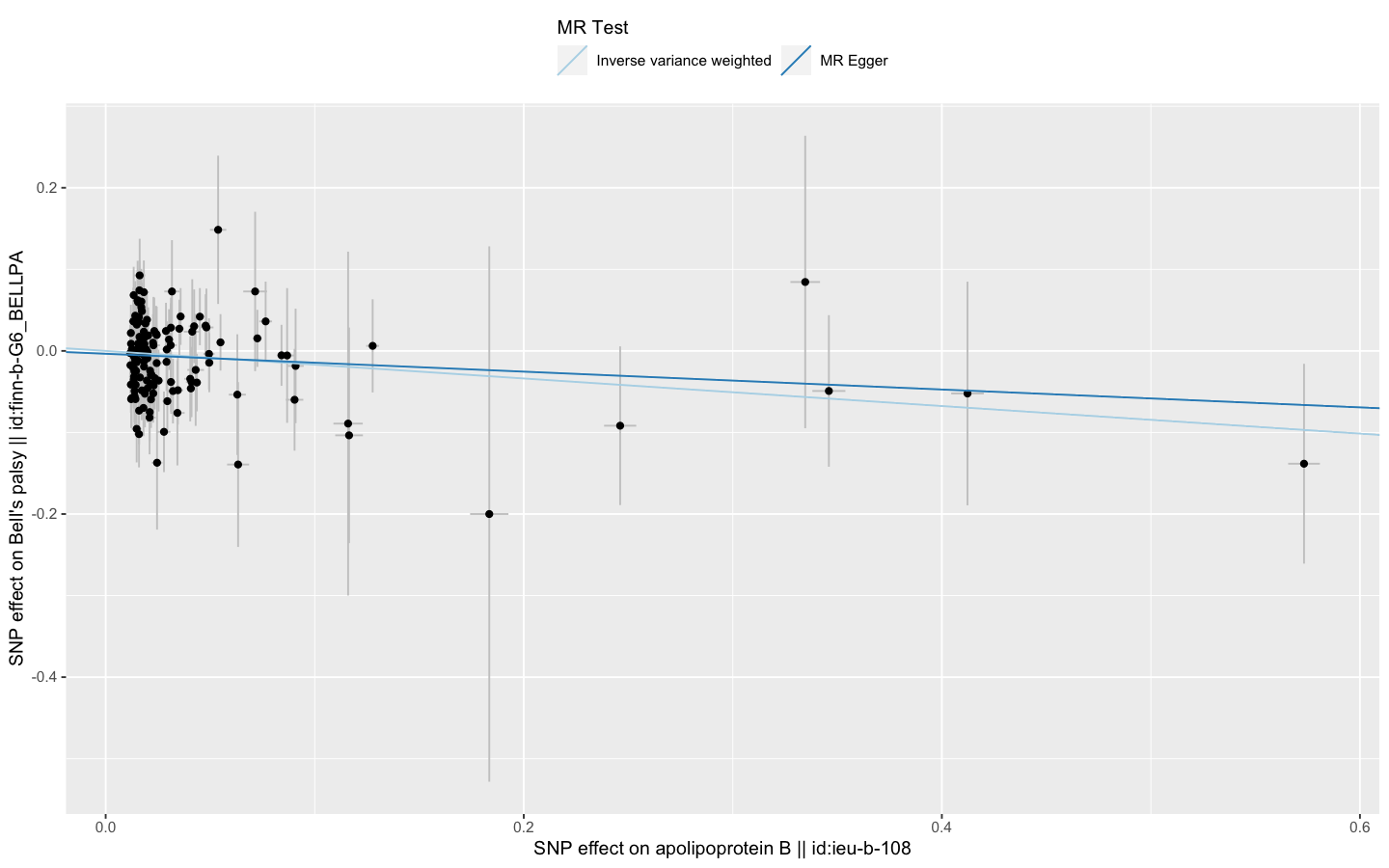


B


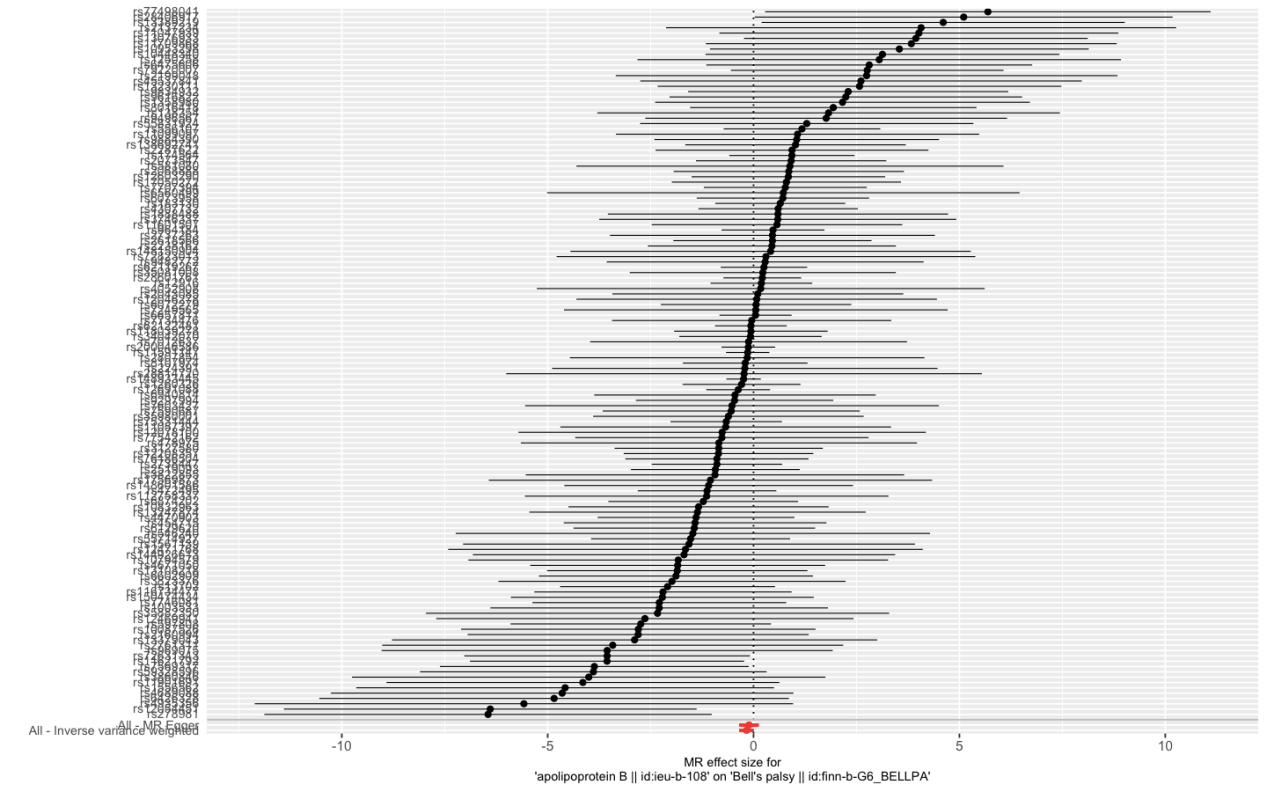


C


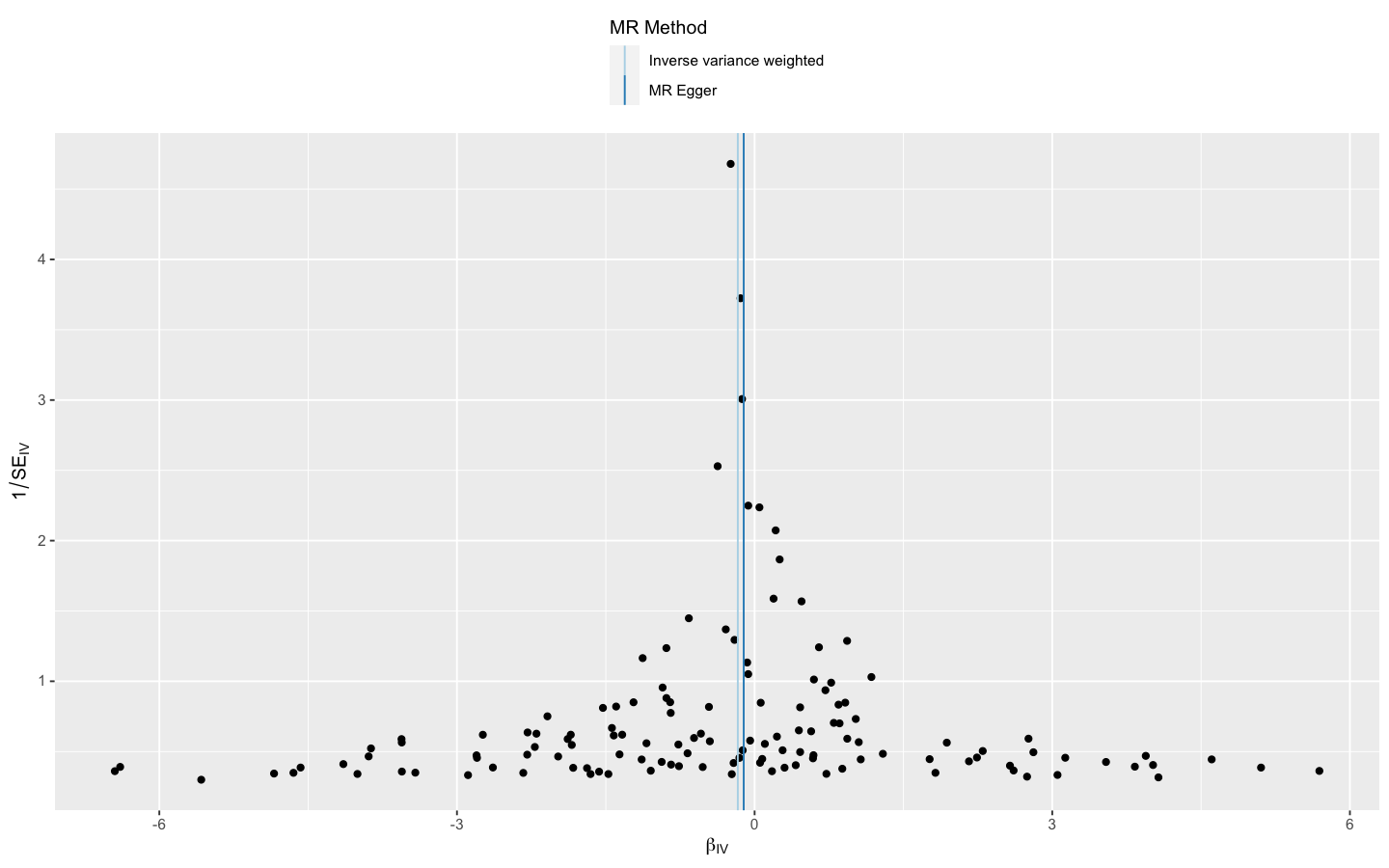


D


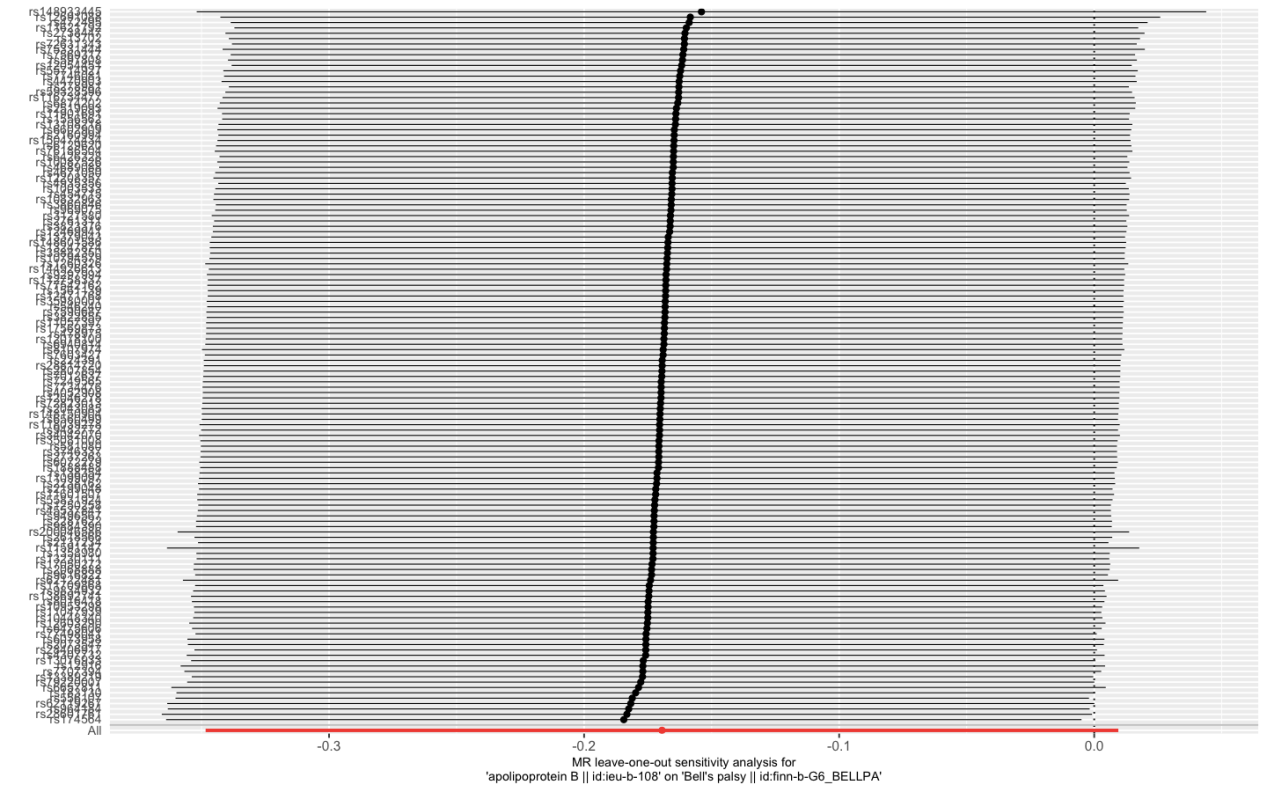


**Supplementary Figure 8 Effect of apolipoprotein B on Bell's palsy risk (removing rs143020224)**

Figure 1A: scatter plot; Figure 1B: forest plot; Figure 1C: funnel plot;

Figure 1D: “leave-one-SNP-out” analysis.

A


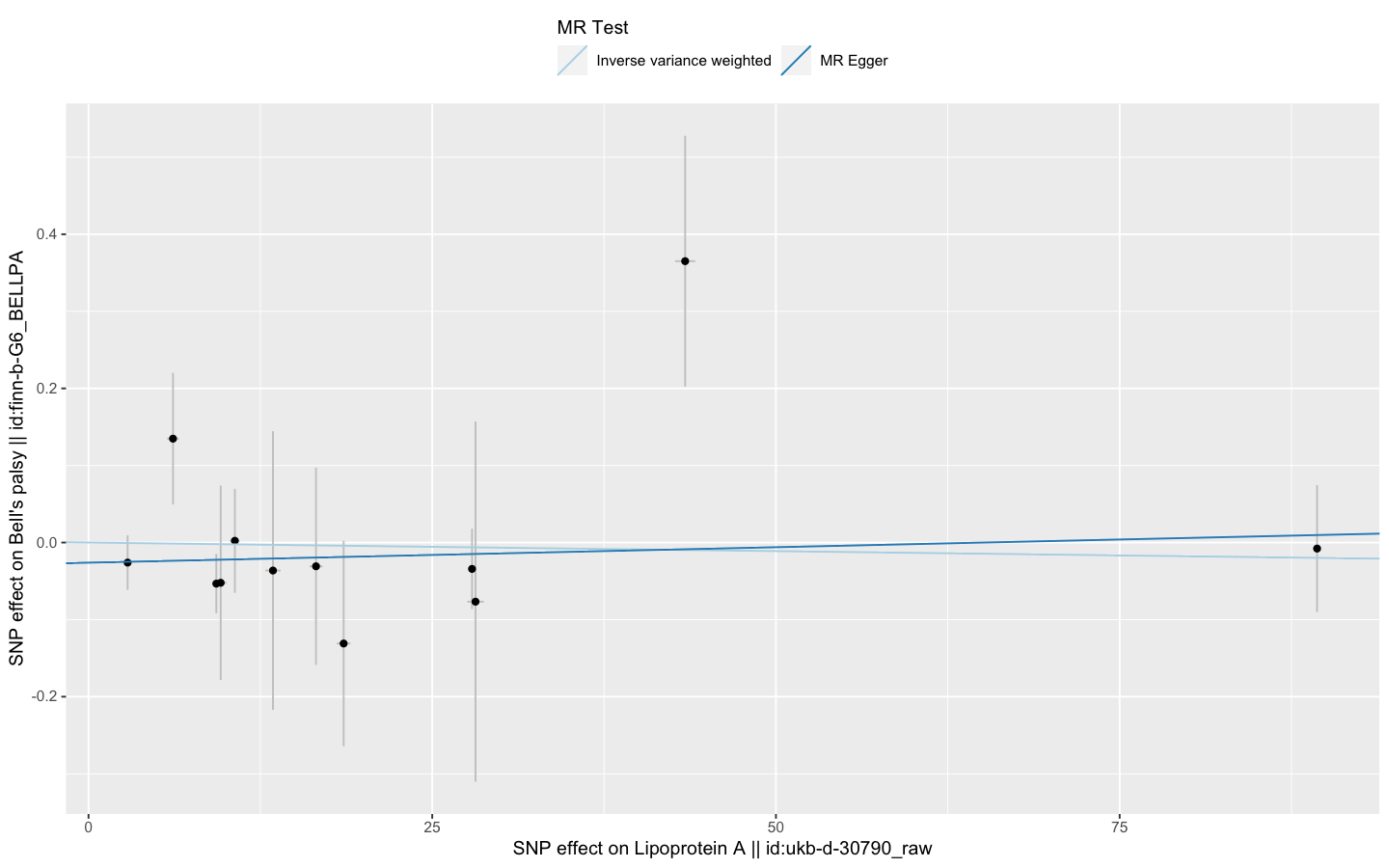


B


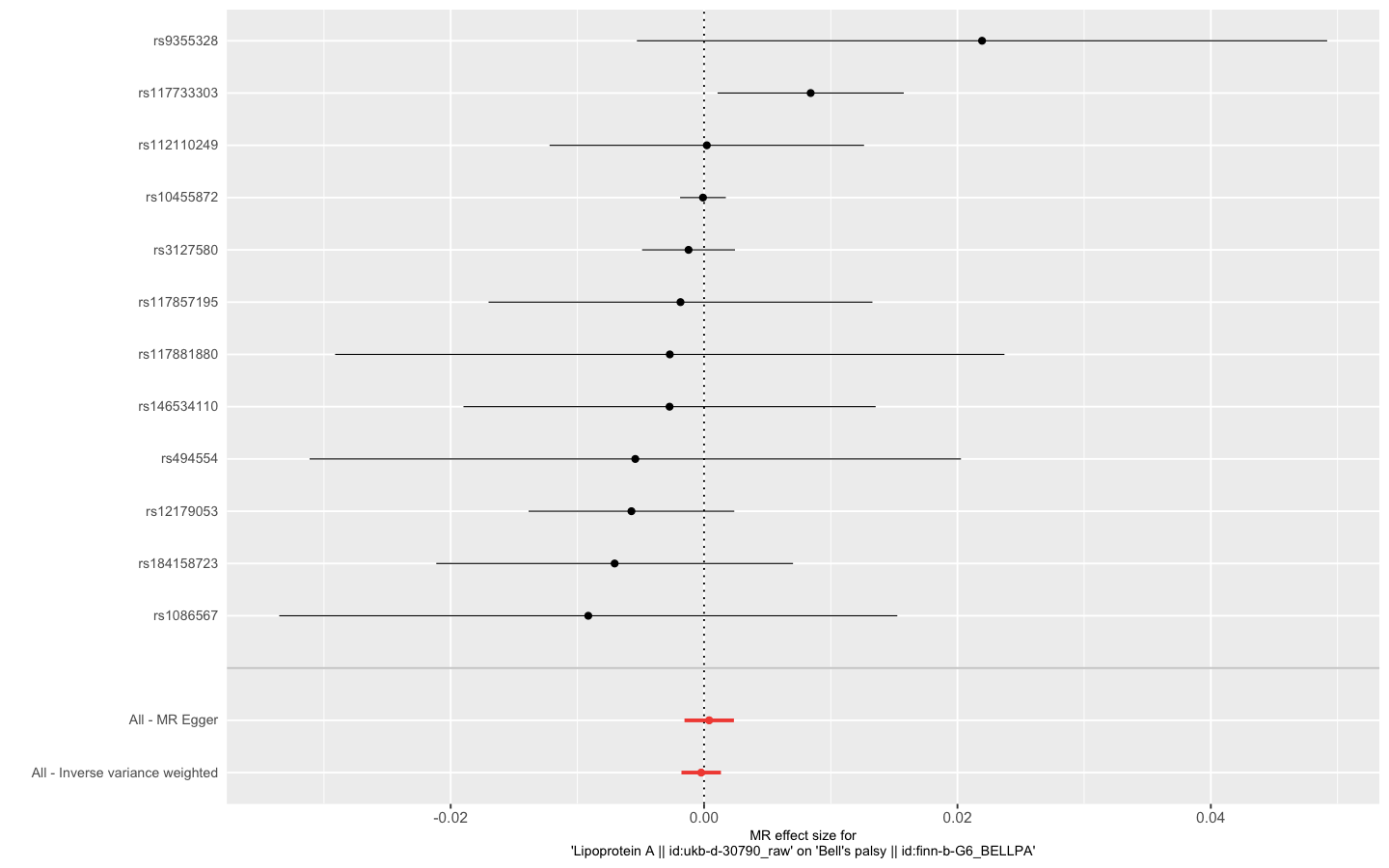


C


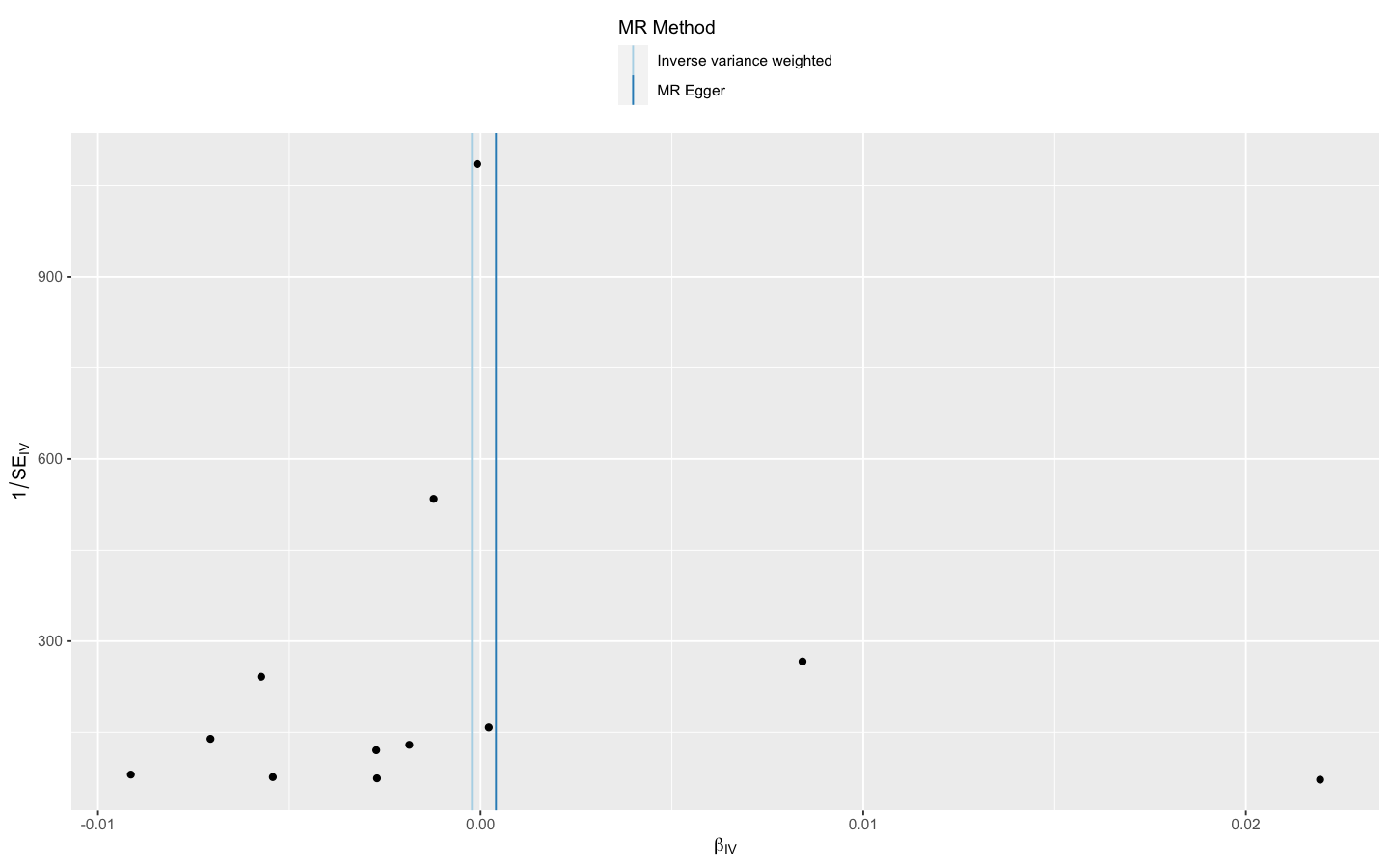


D


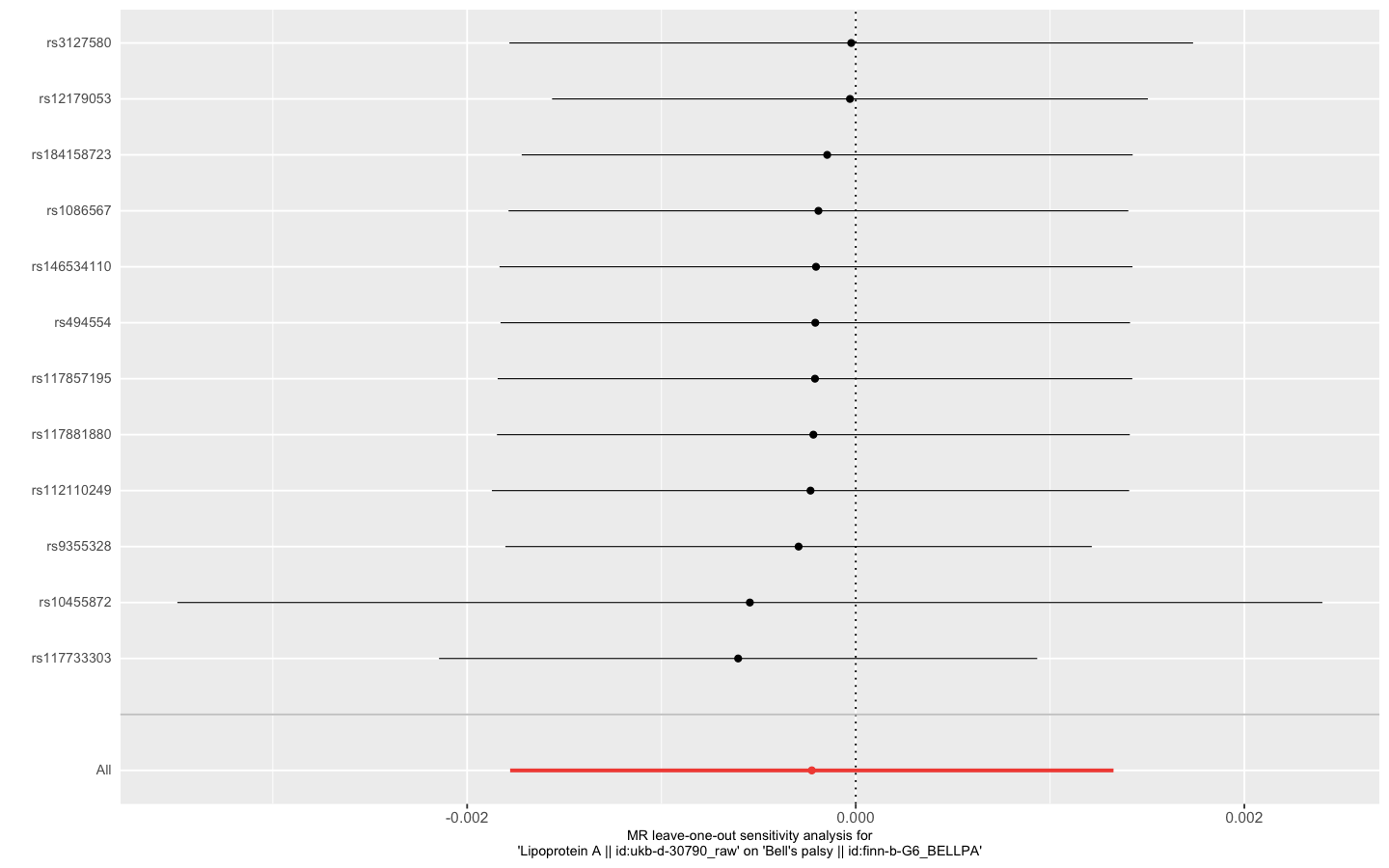


**Supplementary Figure 9 Effect of lipoprotein A on Bell's palsy risk**

Figure 1A: scatter plot; Figure 1B: forest plot; Figure 1C: funnel plot;

Figure 1D: “leave-one-SNP-out” analysis.
