# Supplementary material for: Genetic association of hypertension and several other metabolic disorders with Bell’s palsy
Source: Front Genet. 2023 Jul 18;14:1077438. doi: 10.3389/fgene.2023.1077438 (PMC10391645; doi:10.3389/fgene.2023.1077438)
Supplement: Supplementary file 2 [file DataSheet1.docx]

**Supplementary Table 1 Suitable SNPs for exposures in the study**

| **Exposures** | **SNP** | **Effect**  **allele** | **Other**  **allele** | **Eaf**  **Outcomes** | **Eaf**  **Exposures** | **Beta**  **Exposures** | **Se**  **Exposures** | **P**  **Exposures** | **F statistic** |
| --- | --- | --- | --- | --- | --- | --- | --- | --- | --- |
| **T2DM** |  |  |  |  |  |  |  |  |  |
| 1 | rs10077431 | A | C | 0.1751 | 0.214668 | -0.0487 | 0.0089 | 4.75E-08 | 10.09681542 |
| 2 | rs10087241 | A | G | 0.6393 | 0.594893 | -0.0475 | 0.008 | 2.80E-09 | 16.99610436 |
| 3 | rs10100265 | C | A | 0.5427 | 0.61049 | -0.0491 | 0.0079 | 6.29E-10 | 18.37591745 |
| 4 | rs10114341 | C | T | 0.4237 | 0.440754 | -0.0409 | 0.0072 | 1.15E-08 | 15.91134236 |
| 5 | rs1050226 | G | A | 0.3764 | 0.406792 | -0.0491 | 0.0074 | 3.34E-11 | 21.2540589 |
| 6 | rs1061813 | A | G | 0.6198 | 0.537119 | -0.0429 | 0.0073 | 3.37E-09 | 17.17684985 |
| 7 | rs1063355 | G | T | 0.5253 | 0.602436 | 0.0709 | 0.0079 | 3.72E-19 | 38.60459024 |
| 8 | rs10740322 | A | G | 0.7412 | 0.687104 | 0.0477 | 0.0085 | 2.11E-08 | 13.54350572 |
| 9 | rs10811661 | C | T | 0.1436 | 0.173605 | -0.1569 | 0.0098 | 4.13E-58 | 73.63223945 |
| 10 | rs10830963 | G | C | 0.356 | 0.275768 | 0.0909 | 0.008 | 5.85E-30 | 51.61094559 |
| 11 | rs10842994 | T | C | 0.1733 | 0.197025 | -0.0755 | 0.0091 | 1.02E-16 | 21.78718351 |
| 12 | rs10974438 | C | A | 0.3758 | 0.351215 | 0.0591 | 0.0075 | 3.01E-15 | 28.30987352 |
| 13 | rs11098676 | C | T | 0.8207 | 0.787639 | 0.054 | 0.0096 | 2.03E-08 | 10.58610774 |
| 14 | rs11107116 | T | G | 0.2507 | 0.219714 | 0.0467 | 0.0085 | 3.75E-08 | 10.35130526 |
| 15 | rs1111875 | T | C | 0.4772 | 0.408262 | -0.0948 | 0.0072 | 3.61E-39 | 83.87163022 |
| 16 | rs11257655 | T | C | 0.2646 | 0.206773 | 0.0737 | 0.0087 | 1.97E-17 | 23.54867956 |
| 17 | rs1127655 | T | C | 0.5363 | 0.529064 | -0.0438 | 0.0079 | 2.47E-08 | 15.32096473 |
| 18 | rs11708067 | G | A | 0.1698 | 0.23899 | -0.0965 | 0.0086 | 5.93E-29 | 45.83111032 |
| 19 | rs11926707 | C | T | 0.6516 | 0.625556 | 0.0463 | 0.0082 | 1.69E-08 | 14.93848926 |
| 20 | rs12299509 | G | A | 0.5063 | 0.478622 | 0.0467 | 0.0073 | 2.09E-10 | 20.43104831 |
| 21 | rs12617659 | T | C | 0.1487 | 0.147238 | -0.0685 | 0.0103 | 2.83E-11 | 11.10829309 |
| 22 | rs12910825 | G | A | 0.3686 | 0.360391 | 0.0517 | 0.0074 | 2.16E-12 | 22.51011703 |
| 23 | rs12945601 | C | T | 0.5639 | 0.613603 | -0.048 | 0.008 | 1.72E-09 | 17.07488558 |
| 24 | rs12970134 | A | G | 0.1953 | 0.26512 | 0.0555 | 0.008 | 5.31E-12 | 18.75904044 |
| 25 | rs13234269 | A | T | 0.4643 | 0.492515 | -0.0583 | 0.0078 | 6.98E-14 | 27.93827894 |
| 26 | rs13239186 | T | C | 0.3669 | 0.302029 | 0.0539 | 0.0085 | 2.70E-10 | 16.95738859 |
| 27 | rs13330951 | G | A | 0.5001 | 0.488314 | -0.0456 | 0.0081 | 1.54E-08 | 15.84119434 |
| 28 | rs13389219 | T | C | 0.3486 | 0.394368 | -0.0722 | 0.0074 | 2.11E-22 | 45.50422726 |
| 29 | rs1359790 | A | G | 0.2936 | 0.2867 | -0.0796 | 0.008 | 2.80E-23 | 40.51743752 |
| 30 | rs1496653 | G | A | 0.3117 | 0.204782 | -0.0769 | 0.0088 | 2.57E-18 | 24.88016643 |
| 31 | rs1552224 | C | A | 0.2361 | 0.15421 | -0.1034 | 0.0101 | 8.64E-25 | 27.35132907 |
| 32 | rs17086692 | T | G | 0.3125 | 0.313426 | -0.0467 | 0.0084 | 2.48E-08 | 13.30471618 |
| 33 | rs17168486 | T | C | 0.1981 | 0.173603 | 0.0742 | 0.0094 | 2.18E-15 | 17.88289064 |
| 34 | rs17411031 | G | C | 0.2466 | 0.261729 | -0.045 | 0.0081 | 3.04E-08 | 11.92947145 |
| 35 | rs1758632 | G | C | 0.55 | 0.623407 | 0.0491 | 0.0081 | 1.36E-09 | 17.2572744 |
| 36 | rs17631783 | T | C | 0.2278 | 0.26346 | -0.0487 | 0.0089 | 3.95E-08 | 11.62212037 |
| 37 | rs1801214 | T | C | 0.5764 | 0.599585 | 0.0903 | 0.0074 | 5.52E-34 | 71.57862595 |
| 38 | rs1899951 | T | C | 0.1699 | 0.123288 | -0.1118 | 0.0109 | 1.64E-24 | 22.75001194 |
| 39 | rs2058913 | T | A | 0.5677 | 0.565008 | -0.0491 | 0.0078 | 3.26E-10 | 19.48320289 |
| 40 | rs2246618 | T | C | 0.2949 | 0.307253 | 0.0513 | 0.0084 | 1.20E-09 | 15.88081481 |
| 41 | rs2261181 | T | C | 0.07326 | 0.09647 | 0.0985 | 0.0118 | 9.18E-17 | 12.14907954 |
| 42 | rs2294120 | G | A | 0.4685 | 0.455879 | -0.0443 | 0.0079 | 1.62E-08 | 15.6035087 |
| 43 | rs2296173 | G | A | 0.1904 | 0.212011 | 0.065 | 0.0087 | 7.66E-14 | 18.65570872 |
| 44 | rs2299383 | T | C | 0.4659 | 0.423455 | 0.0412 | 0.0073 | 1.49E-08 | 15.55653198 |
| 45 | rs243019 | C | T | 0.4486 | 0.455831 | 0.0566 | 0.0071 | 2.29E-15 | 31.54189386 |
| 46 | rs2796441 | A | G | 0.417 | 0.416458 | -0.0715 | 0.0073 | 1.96E-22 | 46.66034825 |
| 47 | rs2820426 | G | A | 0.6126 | 0.610058 | 0.0521 | 0.0073 | 1.30E-12 | 24.24289232 |
| 48 | rs2867125 | C | T | 0.8351 | 0.827825 | 0.0601 | 0.0096 | 4.33E-10 | 11.17399438 |
| 49 | rs2908282 | A | G | 0.1069 | 0.177395 | 0.0552 | 0.0094 | 4.25E-09 | 10.06562755 |
| 50 | rs2925979 | C | T | 0.6776 | 0.70085 | -0.0534 | 0.0078 | 9.06E-12 | 19.65890454 |
| 51 | rs2943656 | G | A | 0.617 | 0.635133 | 0.0902 | 0.0074 | 6.70E-34 | 68.93517204 |
| 52 | rs3217992 | T | C | 0.3841 | 0.369901 | 0.0527 | 0.0073 | 7.23E-13 | 24.30265844 |
| 53 | rs340874 | C | T | 0.4313 | 0.563904 | 0.0626 | 0.0073 | 8.41E-18 | 36.18730436 |
| 54 | rs348330 | A | G | 0.6443 | 0.633451 | -0.0487 | 0.0081 | 1.86E-09 | 16.79055564 |
| 55 | rs3802177 | A | G | 0.3762 | 0.311281 | -0.1217 | 0.008 | 2.32E-52 | 99.37972287 |
| 56 | rs459193 | G | A | 0.6826 | 0.745338 | 0.0711 | 0.0083 | 8.81E-18 | 27.86820619 |
| 57 | rs4622883 | G | A | 0.3877 | 0.508544 | -0.0435 | 0.0078 | 3.02E-08 | 15.54984404 |
| 58 | rs4686471 | C | T | 0.609 | 0.609775 | 0.0534 | 0.0081 | 4.28E-11 | 20.68979642 |
| 59 | rs4823182 | G | A | 0.4427 | 0.335748 | 0.0482 | 0.0077 | 3.36E-10 | 17.48218954 |
| 60 | rs4865796 | A | G | 0.6672 | 0.69309 | 0.053 | 0.0078 | 1.33E-11 | 19.64785073 |
| 61 | rs516946 | C | T | 0.7959 | 0.760633 | 0.0824 | 0.0085 | 3.16E-22 | 34.23801415 |
| 62 | rs5215 | T | C | 0.5283 | 0.639941 | -0.0678 | 0.0073 | 2.09E-20 | 39.77571983 |
| 63 | rs55966194 | G | C | 0.2151 | 0.281312 | -0.0526 | 0.0088 | 2.25E-09 | 14.44943119 |
| 64 | rs576674 | A | G | 0.906 | 0.832515 | -0.0654 | 0.0097 | 1.79E-11 | 12.6789633 |
| 65 | rs6059662 | G | A | 0.769 | 0.663176 | 0.0446 | 0.0079 | 1.51E-08 | 14.24170174 |
| 66 | rs61953351 | T | G | 0.2844 | 0.249902 | -0.07 | 0.0091 | 1.98E-14 | 22.1906709 |
| 67 | rs622217 | C | T | 0.5025 | 0.483932 | -0.0485 | 0.0077 | 3.13E-10 | 19.82194853 |
| 68 | rs6494307 | G | C | 0.447 | 0.426225 | -0.0443 | 0.0078 | 1.67E-08 | 15.78061535 |
| 69 | rs6515236 | C | A | 0.2084 | 0.24933 | -0.0504 | 0.0091 | 3.34E-08 | 11.48411063 |
| 70 | rs67232546 | T | C | 0.1518 | 0.209222 | 0.0596 | 0.0096 | 4.66E-10 | 12.75605095 |
| 71 | rs6767484 | G | A | 0.3073 | 0.312261 | 0.1209 | 0.0076 | 2.70E-56 | 108.8765149 |
| 72 | rs6795735 | T | C | 0.3607 | 0.410912 | -0.0558 | 0.0073 | 1.63E-14 | 28.29848837 |
| 73 | rs6878122 | A | G | 0.7721 | 0.681791 | -0.0564 | 0.0079 | 1.19E-12 | 22.12262595 |
| 74 | rs6960043 | C | T | 0.4118 | 0.521837 | 0.064 | 0.0071 | 3.61E-19 | 40.57423792 |
| 75 | rs7144011 | T | G | 0.24 | 0.221063 | 0.0482 | 0.0085 | 1.64E-08 | 11.07560044 |
| 76 | rs7177055 | A | G | 0.6932 | 0.718289 | 0.0647 | 0.0079 | 2.75E-16 | 27.15570429 |
| 77 | rs7240767 | C | T | 0.3952 | 0.383677 | 0.0451 | 0.0081 | 2.16E-08 | 14.66474882 |
| 78 | rs72802358 | C | G | 0.09695 | 0.101594 | -0.1168 | 0.0133 | 1.97E-18 | 14.08109796 |
| 79 | rs72892910 | T | G | 0.2027 | 0.172394 | 0.0648 | 0.0099 | 6.43E-11 | 12.22717174 |
| 80 | rs735949 | C | T | 0.1021 | 0.14115 | -0.0711 | 0.0106 | 1.95E-11 | 10.90981008 |
| 81 | rs753270 | C | T | 0.5779 | 0.583535 | 0.0528 | 0.0079 | 2.70E-11 | 21.71826951 |
| 82 | rs7561798 | G | A | 0.4509 | 0.482171 | 0.04 | 0.0072 | 2.79E-08 | 15.41576466 |
| 83 | rs7572970 | G | A | 0.796 | 0.722047 | 0.059 | 0.0087 | 1.39E-11 | 18.46486042 |
| 84 | rs7607777 | T | G | 0.05385 | 0.105906 | -0.137 | 0.0125 | 9.40E-28 | 22.75611686 |
| 85 | rs7674212 | T | G | 0.4255 | 0.408864 | -0.0465 | 0.0075 | 6.18E-10 | 18.58635281 |
| 86 | rs7685296 | T | C | 0.2939 | 0.279365 | -0.0511 | 0.0081 | 2.32E-10 | 16.02824471 |
| 87 | rs7756992 | G | A | 0.3326 | 0.266896 | 0.1297 | 0.0078 | 6.00E-62 | 108.3833063 |
| 88 | rs780094 | C | T | 0.6432 | 0.612845 | 0.0692 | 0.0074 | 5.16E-21 | 41.5228448 |
| 89 | rs7845219 | C | T | 0.5102 | 0.492786 | -0.0422 | 0.0072 | 4.54E-09 | 17.17688025 |
| 90 | rs7903146 | T | C | 0.1993 | 0.291585 | 0.3059 | 0.0077 | 1.00E-200 | 658.828794 |
| 91 | rs7955901 | T | C | 0.6297 | 0.556668 | -0.0444 | 0.0072 | 7.16E-10 | 18.77466083 |
| 92 | rs8068804 | A | G | 0.2987 | 0.325097 | 0.0587 | 0.0078 | 4.41E-14 | 24.86160448 |
| 93 | rs8108269 | G | T | 0.3131 | 0.281015 | 0.0644 | 0.0079 | 3.11E-16 | 26.86386776 |
| 94 | rs825476 | T | C | 0.595 | 0.580549 | 0.0524 | 0.0073 | 6.80E-13 | 25.10304886 |
| 95 | rs840967 | A | C | 0.4816 | 0.605922 | -0.0497 | 0.008 | 5.44E-10 | 18.43636067 |
| 96 | rs849135 | A | G | 0.464 | 0.499052 | -0.0999 | 0.0072 | 1.04E-43 | 96.40195055 |
| 97 | rs853974 | C | T | 0.7313 | 0.737586 | -0.0601 | 0.0088 | 7.86E-12 | 18.06026631 |
| 98 | rs9369425 | A | G | 0.6891 | 0.708185 | -0.0546 | 0.0085 | 1.13E-10 | 17.05829955 |
| 99 | rs963740 | T | A | 0.2114 | 0.294299 | -0.0479 | 0.0086 | 2.23E-08 | 12.88809769 |
| 100 | rs9894220 | G | A | 0.4107 | 0.43374 | -0.0585 | 0.0079 | 1.52E-13 | 26.94667209 |
| 101 | rs9928094 | G | A | 0.428 | 0.42602 | 0.1045 | 0.0072 | 3.59E-47 | 103.18641 |
| 102 | rs993380 | G | A | 0.5963 | 0.66555 | -0.0507 | 0.0081 | 4.59E-10 | 17.4459415 |
| 103 | rs9940149 | A | G | 0.106 | 0.178556 | -0.058 | 0.0095 | 9.29E-10 | 10.93585854 |
| **Exposures** | **SNP** | **Effect**  **allele** | **Other**  **allele** | **Eaf**  **Outcomes** | **Eaf**  **Exposures** | **Beta**  **Exposures** | **Se**  **Exposures** | **P**  **Exposures** | **F statistic** |
| **Hypertension** |  |  |  |  |  |  |  |  |  |
| 1 | rs10061288 | G | A | 0.4563 | 0.515823 | -0.00749137 | 0.00106635 | 2.14E-12 | 24.65397673 |
| 2 | rs10488611 | A | G | 0.2294 | 0.192156 | -0.00831702 | 0.00136158 | 1.01E-09 | 11.58438438 |
| 3 | rs10493818 | T | C | 0.2668 | 0.385781 | 0.0067188 | 0.00109621 | 8.85E-10 | 17.80370301 |
| 4 | rs10500326 | T | G | 0.1965 | 0.234551 | -0.00723077 | 0.00125751 | 8.93E-09 | 11.87250774 |
| 5 | rs1073913 | C | A | 0.551 | 0.601007 | -0.00790945 | 0.00109119 | 4.22E-13 | 25.19976555 |
| 6 | rs10853521 | G | A | 0.296 | 0.324913 | -0.00751144 | 0.00113766 | 4.05E-11 | 19.12495028 |
| 7 | rs10857147 | T | A | 0.3134 | 0.292191 | 0.0207589 | 0.00117598 | 1.05E-69 | 128.9392658 |
| 8 | rs10930990 | G | C | 0.3256 | 0.323377 | -0.00680353 | 0.00114873 | 3.17E-09 | 15.35096773 |
| 9 | rs10995311 | G | C | 0.3874 | 0.445151 | -0.00680503 | 0.00107639 | 2.58E-10 | 19.74493884 |
| 10 | rs11037579 | T | C | 0.5299 | 0.533606 | 0.00669415 | 0.00106908 | 3.82E-10 | 19.51626408 |
| 11 | rs11072508 | T | C | 0.564 | 0.673798 | -0.0109765 | 0.00114167 | 6.99E-22 | 40.63896941 |
| 12 | rs11187838 | A | G | 0.3453 | 0.431994 | -0.0101888 | 0.00107543 | 2.71E-21 | 44.05513961 |
| 13 | rs11556924 | T | C | 0.3262 | 0.390062 | -0.00776973 | 0.00109211 | 1.13E-12 | 24.08556645 |
| 14 | rs11592100 | A | C | 0.2151 | 0.219836 | 0.00769353 | 0.00128944 | 2.43E-09 | 12.21172161 |
| 15 | rs11608149 | A | G | 0.2618 | 0.261857 | 0.00846907 | 0.0012217 | 4.15E-12 | 18.57797354 |
| 16 | rs11724647 | A | T | 0.2175 | 0.184256 | -0.00986477 | 0.00137489 | 7.25E-13 | 15.47607773 |
| 17 | rs12129611 | A | T | 0.6668 | 0.593776 | -0.00639871 | 0.00111504 | 9.56E-09 | 15.88693293 |
| 18 | rs12258967 | G | C | 0.2207 | 0.297673 | -0.0105188 | 0.00116672 | 1.97E-19 | 33.9898399 |
| 19 | rs12461874 | A | C | 0.2181 | 0.276091 | 0.00763416 | 0.00119387 | 1.61E-10 | 16.34530905 |
| 20 | rs12647238 | G | T | 0.5287 | 0.484063 | -0.0067152 | 0.00106654 | 3.05E-10 | 19.80227539 |
| 21 | rs1275988 | T | C | 0.5422 | 0.616496 | -0.0134538 | 0.00109698 | 1.43E-34 | 71.13964961 |
| 22 | rs12798980 | C | G | 0.1512 | 0.205377 | 0.0105906 | 0.00131775 | 9.25E-16 | 21.08349447 |
| 23 | rs12922822 | T | C | 0.00114 | 0.182238 | -0.0120076 | 0.00138135 | 3.56E-18 | 22.52305292 |
| 24 | rs12922822 | T | C | 0.2249 | 0.182238 | -0.0120076 | 0.00138135 | 3.56E-18 | 22.52305292 |
| 25 | rs12926550 | A | G | 0.2723 | 0.315784 | -0.00836988 | 0.00115 | 3.39E-13 | 22.891963 |
| 26 | rs13179413 | T | C | 0.2539 | 0.284424 | 0.00753875 | 0.00119874 | 3.20E-10 | 16.09973959 |
| 27 | rs13420463 | G | A | 0.215 | 0.227978 | -0.00780625 | 0.0012703 | 8.00E-10 | 13.29350409 |
| 28 | rs1436138 | G | A | 0.357 | 0.358412 | -0.0084618 | 0.00111461 | 3.17E-14 | 26.50818423 |
| 29 | rs1567168 | A | C | 0.4368 | 0.484626 | 0.00602924 | 0.00106738 | 1.62E-08 | 15.93911438 |
| 30 | rs164749 | G | T | 0.4184 | 0.427 | -0.008158 | 0.00107777 | 3.76E-14 | 28.03891813 |
| 31 | rs1650581 | G | C | 0.2249 | 0.262035 | 0.0102524 | 0.0012169 | 3.62E-17 | 27.4535905 |
| 32 | rs167479 | T | G | 0.4249 | 0.470348 | -0.0133572 | 0.00106605 | 5.24E-36 | 78.23729248 |
| 33 | rs16885627 | G | C | 0.4669 | 0.401635 | 0.00656368 | 0.00109342 | 1.94E-09 | 17.32081156 |
| 34 | rs1689040 | T | C | 0.2884 | 0.405735 | -0.011277 | 0.00108609 | 2.98E-25 | 51.99631279 |
| 35 | rs17335134 | G | A | 0.2469 | 0.275143 | -0.00751195 | 0.00119687 | 3.47E-10 | 15.71338963 |
| 36 | rs17637472 | A | G | 0.4422 | 0.401043 | 0.00833543 | 0.00109495 | 2.69E-14 | 27.84308221 |
| 37 | rs17677603 | G | A | 0.3384 | 0.394587 | 0.00871735 | 0.00109576 | 1.79E-15 | 30.24119633 |
| 38 | rs1882961 | T | C | 0.3934 | 0.306476 | 0.00647928 | 0.00116059 | 2.37E-08 | 13.24946369 |
| 39 | rs1887320 | A | G | 0.4352 | 0.476784 | 0.0104778 | 0.00106809 | 1.03E-22 | 48.01937123 |
| 40 | rs1973407 | A | G | 0.2509 | 0.272759 | -0.00696639 | 0.00120412 | 7.24E-09 | 13.27938607 |
| 41 | rs198851 | G | T | 0.8901 | 0.848911 | -0.014224 | 0.00148812 | 1.20E-21 | 23.43798096 |
| 42 | rs2014590 | T | C | 0.5327 | 0.487316 | -0.00677839 | 0.00106726 | 2.14E-10 | 20.15702608 |
| 43 | rs2078339 | G | A | 0.1814 | 0.271239 | -0.00799702 | 0.00119925 | 2.59E-11 | 17.58021701 |
| 44 | rs2105092 | A | G | 0.2276 | 0.2908 | -0.00644064 | 0.00117474 | 4.19E-08 | 12.39884 |
| 45 | rs2287922 | A | G | 0.4196 | 0.536146 | 0.00641003 | 0.00107017 | 2.10E-09 | 17.84552634 |
| 46 | rs2291433 | G | T | 0.4395 | 0.540015 | -0.00601224 | 0.00106888 | 1.86E-08 | 15.71852806 |
| 47 | rs231708 | C | G | 0.738 | 0.686651 | -0.00656473 | 0.0011478 | 1.07E-08 | 14.07703591 |
| 48 | rs2341599 | A | G | 0.5777 | 0.659223 | -0.00643731 | 0.00112626 | 1.09E-08 | 14.67845956 |
| 49 | rs2443708 | C | T | 0.6285 | 0.68549 | 0.00769786 | 0.00115017 | 2.19E-11 | 19.31540536 |
| 50 | rs2478543 | C | T | 0.4292 | 0.401346 | 0.00768545 | 0.00108899 | 1.70E-12 | 23.93557677 |
| 51 | rs2627323 | T | C | 0.4055 | 0.466748 | 0.00818512 | 0.00106714 | 1.72E-14 | 29.28784423 |
| 52 | rs2643826 | T | C | 0.4048 | 0.4525 | 0.0107941 | 0.00107303 | 8.40E-24 | 50.1468736 |
| 53 | rs2673802 | G | A | 0.8085 | 0.743531 | 0.00678052 | 0.00122111 | 2.81E-08 | 11.75961091 |
| 54 | rs268263 | A | T | 0.7917 | 0.75424 | 0.00784677 | 0.00124822 | 3.25E-10 | 14.65097821 |
| 55 | rs2736689 | A | C | 0.5087 | 0.497214 | -0.00581131 | 0.00106575 | 4.96E-08 | 14.86656032 |
| 56 | rs2760061 | A | T | 0.4653 | 0.477559 | 0.00667934 | 0.00107799 | 5.79E-10 | 19.15816991 |
| 57 | rs2838351 | G | A | 0.6635 | 0.555638 | 0.00625391 | 0.00107319 | 5.64E-09 | 16.76980034 |
| 58 | rs28667801 | T | A | 0.478 | 0.406256 | 0.00809012 | 0.00109011 | 1.16E-13 | 26.57234901 |
| 59 | rs2881854 | A | C | 0.8063 | 0.849681 | -0.0109233 | 0.0014895 | 2.25E-13 | 13.73857245 |
| 60 | rs2928142 | C | T | 0.6533 | 0.571579 | -0.00735 | 0.00107697 | 8.82E-12 | 22.81241611 |
| 61 | rs2977324 | G | T | 0.7174 | 0.701089 | 0.00681436 | 0.00116859 | 5.51E-09 | 14.25235542 |
| 62 | rs3020644 | G | A | 0.3617 | 0.359599 | 0.00867111 | 0.00110985 | 5.61E-15 | 28.11609032 |
| 63 | rs33301 | A | C | 0.6202 | 0.602367 | 0.00604958 | 0.00108797 | 2.69E-08 | 14.81176106 |
| 64 | rs34344953 | C | T | 0.4467 | 0.361129 | 0.00620089 | 0.00111056 | 2.36E-08 | 14.38618002 |
| 65 | rs35429 | G | A | 0.3644 | 0.382709 | -0.0106679 | 0.00109716 | 2.42E-22 | 44.6746874 |
| 66 | rs35432681 | C | T | 0.4421 | 0.38064 | 0.00684977 | 0.00109898 | 4.59E-10 | 18.31812396 |
| 67 | rs35942721 | T | C | 0.1869 | 0.290533 | -0.00744067 | 0.00117524 | 2.44E-10 | 16.52522311 |
| 68 | rs36071027 | T | C | 0.387 | 0.361625 | -0.00804318 | 0.00111102 | 4.51E-13 | 24.19940731 |
| 69 | rs3790607 | C | A | 0.1744 | 0.0784299 | 0.0191848 | 0.00198668 | 4.63E-22 | 13.48073382 |
| 70 | rs3803266 | C | G | 0.6399 | 0.768714 | -0.00828718 | 0.00126353 | 5.43E-11 | 15.29690914 |
| 71 | rs3809060 | T | G | 0.3086 | 0.37435 | 0.00643525 | 0.00110354 | 5.50E-09 | 15.92985827 |
| 72 | rs3821843 | A | G | 0.6594 | 0.679566 | 0.00949922 | 0.0011588 | 2.46E-16 | 29.26808249 |
| 73 | rs3828591 | C | G | 0.3634 | 0.392998 | -0.012152 | 0.00109254 | 9.85E-29 | 59.03434469 |
| 74 | rs3918226 | T | C | 0.06973 | 0.0815898 | 0.0251463 | 0.00197493 | 3.97E-37 | 24.29830925 |
| 75 | rs4260902 | G | A | 0.4305 | 0.46633 | 0.00590231 | 0.0010705 | 3.52E-08 | 15.13156137 |
| 76 | rs4423794 | A | G | 0.3751 | 0.376746 | 0.00626795 | 0.00110186 | 1.28E-08 | 15.19703735 |
| 77 | rs448385 | A | G | 0.4093 | 0.440915 | 0.00682794 | 0.00107376 | 2.03E-10 | 19.93658473 |
| 78 | rs4844949 | A | G | 0.2156 | 0.369872 | 0.0060787 | 0.00110728 | 4.03E-08 | 14.04857448 |
| 79 | rs4968783 | A | C | 0.614 | 0.619406 | -0.00885509 | 0.00110138 | 9.01E-16 | 30.48004557 |
| 80 | rs4980379 | T | C | 0.4058 | 0.364915 | 0.0110694 | 0.00111004 | 2.03E-23 | 46.09791368 |
| 81 | rs4981000 | C | T | 0.6033 | 0.49117 | -0.00680968 | 0.00106829 | 1.84E-10 | 20.31108881 |
| 82 | rs55872725 | T | C | 0.4182 | 0.402363 | 0.00877746 | 0.00108655 | 6.59E-16 | 31.38785581 |
| 83 | rs56388530 | T | C | 0.8285 | 0.75956 | 0.00913047 | 0.00124652 | 2.40E-13 | 19.5978863 |
| 84 | rs57139556 | G | A | 0.1059 | 0.0716654 | -0.0180907 | 0.00206548 | 1.99E-18 | 10.20759404 |
| 85 | rs58363161 | A | T | 0.3781 | 0.403019 | 0.00616523 | 0.00108566 | 1.36E-08 | 15.51830669 |
| 86 | rs58880983 | G | A | 0.2666 | 0.339252 | -0.00626108 | 0.00112592 | 2.69E-08 | 13.8639502 |
| 87 | rs6026739 | T | A | 0.1692 | 0.11777 | 0.0202897 | 0.00166013 | 2.42E-34 | 31.04209016 |
| 88 | rs6031431 | G | A | 0.494 | 0.460907 | 0.00740028 | 0.00107419 | 5.62E-12 | 23.58679278 |
| 89 | rs604723 | C | T | 0.7409 | 0.724339 | 0.0148829 | 0.00119978 | 2.51E-35 | 61.46044035 |
| 90 | rs6108168 | A | C | 0.2295 | 0.255081 | -0.010394 | 0.00122166 | 1.78E-17 | 27.51152944 |
| 91 | rs61862567 | A | G | 0.4281 | 0.507674 | -0.00588315 | 0.00106669 | 3.48E-08 | 15.20645902 |
| 92 | rs642803 | T | C | 0.3971 | 0.464339 | -0.00779692 | 0.00106859 | 2.96E-13 | 26.48565203 |
| 93 | rs6464165 | C | T | 0.2296 | 0.282572 | 0.00690473 | 0.00118478 | 5.62E-09 | 13.77116173 |
| 94 | rs682709 | G | C | 0.6128 | 0.666099 | 0.00753247 | 0.0011431 | 4.42E-11 | 19.31590999 |
| 95 | rs6923212 | C | T | 0.1228 | 0.190219 | 0.00794242 | 0.00135887 | 5.07E-09 | 10.52476312 |
| 96 | rs6944634 | G | C | 0.1516 | 0.217855 | 0.00768813 | 0.00129316 | 2.76E-09 | 12.04577237 |
| 97 | rs6985793 | C | T | 0.3541 | 0.310168 | -0.00637999 | 0.00115774 | 3.58E-08 | 12.99578161 |
| 98 | rs7117386 | A | G | 0.7597 | 0.789332 | 0.0118999 | 0.00130555 | 7.92E-20 | 27.63254584 |
| 99 | rs7125487 | G | C | 0.7236 | 0.726357 | 0.00701821 | 0.00119914 | 4.84E-09 | 13.61734269 |
| 100 | rs7258382 | C | T | 0.08555 | 0.164358 | -0.0112961 | 0.00143543 | 3.57E-15 | 17.011925 |
| 101 | rs72831343 | G | T | 0.1023 | 0.145717 | -0.0184644 | 0.0015074 | 1.73E-34 | 37.35948692 |
| 102 | rs72915163 | T | C | 0.2189 | 0.258623 | 0.00870236 | 0.00123116 | 1.57E-12 | 19.16035052 |
| 103 | rs72915163 | T | C | 0.000477 | 0.258623 | 0.00870236 | 0.00123116 | 1.57E-12 | 19.16035052 |
| 104 | rs73053851 | G | C | 0.2618 | 0.268745 | -0.00674877 | 0.00120427 | 2.10E-08 | 12.34394792 |
| 105 | rs7310615 | G | C | 0.5863 | 0.516763 | -0.0117391 | 0.00107351 | 7.90E-28 | 59.73286713 |
| 106 | rs7340705 | C | T | 0.3611 | 0.32143 | 0.00713831 | 0.00114734 | 4.93E-10 | 16.88638837 |
| 107 | rs740746 | A | G | 0.7475 | 0.734879 | 0.0107596 | 0.00121059 | 6.26E-19 | 30.78400531 |
| 108 | rs74661587 | G | A | 0.2227 | 0.132036 | 0.0106037 | 0.00157235 | 1.54E-11 | 10.42440637 |
| 109 | rs7515635 | C | T | 0.4741 | 0.543067 | -0.00611983 | 0.00107211 | 1.14E-08 | 16.1716454 |
| 110 | rs76452347 | T | C | 0.179 | 0.204343 | -0.00933201 | 0.00136953 | 9.51E-12 | 15.09870889 |
| 111 | rs7701003 | G | A | 0.3447 | 0.368047 | -0.0106918 | 0.00110455 | 3.70E-22 | 43.59157101 |
| 112 | rs7763350 | C | A | 0.304 | 0.319377 | 0.00805425 | 0.00114179 | 1.74E-12 | 21.6343218 |
| 113 | rs7777545 | C | T | 0.3553 | 0.410909 | -0.0062642 | 0.00108555 | 7.91E-09 | 16.12158709 |
| 114 | rs8027450 | T | C | 0.2672 | 0.324065 | 0.0129055 | 0.00114296 | 1.47E-29 | 55.86297252 |
| 115 | rs8065350 | A | G | 0.3186 | 0.243668 | 0.00734544 | 0.00126755 | 6.84E-09 | 12.37826483 |
| 116 | rs8070737 | T | G | 0.1953 | 0.184287 | 0.00822151 | 0.00137321 | 2.14E-09 | 10.77711828 |
| 117 | rs8118848 | A | G | 0.2163 | 0.23658 | -0.00890795 | 0.00125568 | 1.30E-12 | 18.17983698 |
| 118 | rs880315 | C | T | 0.4134 | 0.340442 | 0.0128945 | 0.00112999 | 3.72E-30 | 58.48691155 |
| 119 | rs9286351 | G | A | 0.4678 | 0.41763 | 0.00629367 | 0.00108242 | 6.09E-09 | 16.44582273 |
| 120 | rs9368222 | A | C | 0.3291 | 0.262035 | 0.00675834 | 0.00121057 | 2.37E-08 | 12.05419669 |
| 121 | rs9375459 | T | C | 0.4951 | 0.435415 | 0.0123507 | 0.00107271 | 1.14E-30 | 65.18723924 |
| 122 | rs951914 | C | G | 0.6553 | 0.713884 | 0.00905843 | 0.00118571 | 2.18E-14 | 23.84387438 |
| 123 | rs9824956 | T | C | 0.2384 | 0.296404 | 0.00643011 | 0.00117181 | 4.08E-08 | 12.55953106 |
| 124 | rs9972727 | G | A | 0.6189 | 0.645202 | 0.0065241 | 0.00111974 | 5.67E-09 | 15.54286737 |
| **Exposures** | **SNP** | **Effect**  **allele** | **Other**  **allele** | **Eaf**  **Outcomes** | **Eaf**  **Exposures** | **Beta**  **Exposures** | **Se**  **Exposures** | **P**  **Exposures** | **F statistic** |
| **BMI** |  |  |  |  |  |  |  |  |  |
| 1 | rs10009336 | T | C | 0.1652 | 0.1638 | -0.014 | 0.0022 | 2.20E-10 | 11.09352931 |
| 2 | rs1006896 | C | A | 0.1019 | 0.1061 | -0.0234 | 0.0027 | 5.50E-18 | 14.24774688 |
| 3 | rs10132280 | A | C | 0.3485 | 0.3017 | -0.0223 | 0.0018 | 5.60E-35 | 64.67652357 |
| 4 | rs10169594 | C | T | 0.3607 | 0.3596 | 0.0121 | 0.0018 | 2.00E-11 | 20.81318994 |
| 5 | rs10182181 | G | A | 0.418 | 0.4753 | 0.0325 | 0.0016 | 6.70E-90 | 205.8483458 |
| 6 | rs10192119 | G | T | 0.1978 | 0.1673 | 0.0166 | 0.0022 | 3.00E-14 | 15.86327618 |
| 7 | rs10197031 | C | T | 0.3434 | 0.2834 | 0.0166 | 0.0019 | 1.90E-18 | 31.00514997 |
| 8 | rs10243319 | C | T | 0.4414 | 0.3939 | -0.0107 | 0.0018 | 1.20E-09 | 16.87299408 |
| 9 | rs10248136 | T | C | 0.4828 | 0.5142 | -0.0097 | 0.0017 | 2.00E-08 | 16.2657549 |
| 10 | rs10269783 | A | G | 0.4581 | 0.3896 | 0.0133 | 0.0017 | 1.40E-15 | 29.1127883 |
| 11 | rs10408324 | T | C | 0.3056 | 0.2744 | -0.0124 | 0.0019 | 9.50E-11 | 16.96121471 |
| 12 | rs10478110 | C | A | 0.4941 | 0.4348 | 0.01 | 0.0017 | 9.60E-09 | 17.00722353 |
| 13 | rs1048932 | A | C | 0.429 | 0.4162 | -0.016 | 0.0017 | 3.80E-22 | 43.04876569 |
| 14 | rs10492229 | T | C | 0.2714 | 0.2268 | 0.0142 | 0.0019 | 7.70E-14 | 19.59043093 |
| 15 | rs10510419 | T | G | 0.1432 | 0.1416 | -0.0177 | 0.0023 | 2.20E-14 | 14.39728626 |
| 16 | rs1064213 | A | G | 0.3981 | 0.492 | 0.012 | 0.0017 | 2.40E-12 | 24.90794079 |
| 17 | rs10733051 | G | A | 0.5671 | 0.4802 | -0.0097 | 0.0016 | 2.90E-09 | 18.34851874 |
| 18 | rs10742752 | C | T | 0.5727 | 0.6159 | 0.0124 | 0.0017 | 1.10E-13 | 25.17344947 |
| 19 | rs10747488 | A | C | 0.6828 | 0.7601 | -0.0123 | 0.002 | 1.20E-09 | 13.79394972 |
| 20 | rs10750215 | T | G | 0.3938 | 0.3883 | 0.0108 | 0.0017 | 1.30E-10 | 19.17321706 |
| 21 | rs1075901 | C | T | 0.595 | 0.5639 | 0.0121 | 0.0016 | 1.20E-13 | 28.12957884 |
| 22 | rs10768994 | C | T | 0.4215 | 0.4337 | -0.0114 | 0.0017 | 6.40E-12 | 22.08965127 |
| 23 | rs10795422 | G | A | 0.7221 | 0.6905 | 0.0139 | 0.0019 | 9.30E-14 | 22.87651318 |
| 24 | rs10811871 | G | A | 0.3752 | 0.3829 | -0.0108 | 0.0018 | 1.60E-09 | 17.0130786 |
| 25 | rs10832778 | G | C | 0.5626 | 0.6222 | 0.0125 | 0.0017 | 1.30E-13 | 25.41892196 |
| 26 | rs10867256 | T | C | 0.6036 | 0.553 | -0.0118 | 0.0017 | 8.70E-12 | 23.82004434 |
| 27 | rs10878946 | T | C | 0.6479 | 0.714 | -0.0141 | 0.0019 | 3.60E-13 | 22.4925266 |
| 28 | rs10887578 | C | G | 0.4862 | 0.4896 | 0.0128 | 0.0017 | 1.60E-13 | 28.33485578 |
| 29 | rs10914462 | G | A | 0.4315 | 0.4255 | -0.0112 | 0.0017 | 1.50E-10 | 21.22119797 |
| 30 | rs10915840 | A | G | 0.2329 | 0.283 | -0.0118 | 0.0019 | 1.30E-09 | 15.65312515 |
| 31 | rs10920678 | G | A | 0.474 | 0.5709 | -0.0155 | 0.0016 | 1.50E-21 | 45.98288385 |
| 32 | rs10938397 | G | A | 0.4726 | 0.4317 | 0.0324 | 0.0016 | 3.40E-86 | 201.2559807 |
| 33 | rs10942267 | G | A | 0.2205 | 0.3088 | -0.0156 | 0.0019 | 3.90E-17 | 28.77861495 |
| 34 | rs10953740 | G | A | 0.5196 | 0.5534 | -0.0153 | 0.0017 | 1.00E-18 | 40.04027261 |
| 35 | rs10962550 | C | G | 0.1758 | 0.1801 | 0.0182 | 0.0022 | 6.20E-16 | 20.21219421 |
| 36 | rs10968114 | C | A | 0.5111 | 0.4681 | -0.0113 | 0.0017 | 6.10E-11 | 22.00241409 |
| 37 | rs10971709 | T | C | 0.1825 | 0.2062 | 0.0132 | 0.0021 | 6.20E-10 | 12.93438915 |
| 38 | rs11030618 | T | C | 0.548 | 0.5679 | 0.011 | 0.0017 | 2.40E-10 | 20.54874642 |
| 39 | rs11066188 | A | G | 0.379 | 0.4181 | -0.012 | 0.0017 | 8.10E-13 | 24.24573513 |
| 40 | rs11084553 | G | A | 0.1306 | 0.1518 | -0.021 | 0.0024 | 1.80E-18 | 19.71638429 |
| 41 | rs11105839 | A | T | 0.3605 | 0.3799 | -0.0109 | 0.0017 | 1.10E-10 | 19.36983073 |
| 42 | rs11115176 | C | T | 0.226 | 0.2399 | -0.0121 | 0.0019 | 2.00E-10 | 14.79113584 |
| 43 | rs11118308 | G | A | 0.4519 | 0.4703 | -0.0101 | 0.0016 | 4.80E-10 | 19.85397576 |
| 44 | rs11150911 | C | A | 0.6943 | 0.7191 | -0.0133 | 0.0018 | 4.70E-13 | 22.05669501 |
| 45 | rs11165643 | T | C | 0.5989 | 0.5828 | 0.0206 | 0.0017 | 1.40E-35 | 71.41155092 |
| 46 | rs11170468 | C | A | 0.1898 | 0.2326 | -0.0123 | 0.0019 | 1.90E-10 | 14.96137815 |
| 47 | rs11173522 | A | C | 0.1931 | 0.2078 | 0.0128 | 0.0021 | 1.10E-09 | 12.23201914 |
| 48 | rs11251352 | G | A | 0.5418 | 0.5988 | 0.0109 | 0.0018 | 7.00E-10 | 17.61937573 |
| 49 | rs1144387 | C | G | 0.5496 | 0.5714 | 0.0098 | 0.0017 | 1.60E-08 | 16.27742583 |
| 50 | rs11496125 | T | C | 0.4663 | 0.4212 | 0.0169 | 0.0017 | 3.00E-22 | 48.18942549 |
| 51 | rs11538 | G | A | 0.1928 | 0.1805 | 0.0135 | 0.0023 | 3.30E-09 | 10.19232266 |
| 52 | rs1158805 | A | C | 0.4463 | 0.3766 | -0.0137 | 0.0018 | 1.20E-14 | 27.20126205 |
| 53 | rs11609659 | C | T | 0.254 | 0.2371 | -0.0154 | 0.002 | 2.20E-14 | 21.44979035 |
| 54 | rs11611246 | T | G | 0.2165 | 0.21 | 0.024 | 0.002 | 5.00E-32 | 47.78200503 |
| 55 | rs11615578 | T | C | 0.2315 | 0.2474 | 0.013 | 0.002 | 8.10E-11 | 15.73365156 |
| 56 | rs11656076 | A | G | 0.2636 | 0.2254 | -0.0142 | 0.0021 | 5.60E-12 | 15.96644663 |
| 57 | rs11672660 | T | C | 0.2575 | 0.2049 | -0.034 | 0.0021 | 1.70E-60 | 85.42010412 |
| 58 | rs11713193 | A | G | 0.3976 | 0.5073 | 0.0239 | 0.0017 | 2.40E-44 | 98.81801448 |
| 59 | rs11736228 | T | A | 0.2344 | 0.2587 | -0.0139 | 0.002 | 4.10E-12 | 18.52679994 |
| 60 | rs11738695 | A | C | 0.5783 | 0.586 | 0.0097 | 0.0017 | 2.00E-08 | 15.79727743 |
| 61 | rs11739877 | T | C | 0.534 | 0.6118 | 0.0117 | 0.0018 | 6.60E-11 | 20.06933784 |
| 62 | rs11781699 | C | T | 0.2831 | 0.1896 | 0.0132 | 0.0021 | 3.10E-10 | 12.14178805 |
| 63 | rs11855853 | T | C | 0.2156 | 0.2649 | -0.0145 | 0.002 | 2.40E-13 | 20.47133392 |
| 64 | rs1187352 | C | T | 0.6861 | 0.6518 | 0.0119 | 0.0018 | 6.00E-11 | 19.83961698 |
| 65 | rs11880870 | G | A | 0.5281 | 0.4801 | -0.0189 | 0.0017 | 1.00E-28 | 61.70827858 |
| 66 | rs11889536 | G | A | 0.05339 | 0.1493 | -0.0189 | 0.0024 | 6.40E-15 | 15.75346275 |
| 67 | rs11908637 | A | G | 0.2779 | 0.236 | -0.012 | 0.0021 | 4.90E-09 | 11.77512157 |
| 68 | rs11945861 | A | G | 0.2289 | 0.2369 | -0.0148 | 0.002 | 5.00E-13 | 19.79936566 |
| 69 | rs11951673 | T | C | 0.3013 | 0.3941 | -0.0123 | 0.0017 | 1.10E-13 | 25.00128733 |
| 70 | rs12041258 | C | T | 0.2374 | 0.2287 | -0.0146 | 0.002 | 9.50E-13 | 18.80077742 |
| 71 | rs12044597 | G | A | 0.5156 | 0.5029 | 0.0143 | 0.0016 | 1.70E-18 | 39.94002973 |
| 72 | rs12049202 | T | C | 0.284 | 0.203 | 0.024 | 0.0022 | 1.00E-28 | 38.5109653 |
| 73 | rs12098284 | T | C | 0.07331 | 0.1241 | 0.0178 | 0.0026 | 1.80E-11 | 10.18954509 |
| 74 | rs12150665 | C | T | 0.3693 | 0.4058 | -0.0162 | 0.0017 | 1.60E-22 | 43.79552021 |
| 75 | rs1218822 | A | G | 0.667 | 0.6663 | 0.0168 | 0.0017 | 1.90E-22 | 43.43095478 |
| 76 | rs12299814 | A | C | 0.342 | 0.2525 | -0.0157 | 0.002 | 5.20E-15 | 23.26244241 |
| 77 | rs12328930 | C | T | 0.3762 | 0.4235 | 0.0098 | 0.0017 | 1.80E-08 | 16.22728929 |
| 78 | rs12334877 | A | G | 0.2385 | 0.198 | -0.0144 | 0.0022 | 7.70E-11 | 13.60681713 |
| 79 | rs12364470 | G | T | 0.2256 | 0.1626 | 0.0178 | 0.0022 | 1.10E-15 | 17.82735308 |
| 80 | rs12369179 | T | C | 0.08408 | 0.08782 | -0.0359 | 0.0031 | 2.50E-31 | 21.48730931 |
| 81 | rs12416812 | A | G | 0.5643 | 0.5088 | 0.0111 | 0.0016 | 6.10E-12 | 24.05766779 |
| 82 | rs12422552 | C | G | 0.2122 | 0.2663 | -0.0134 | 0.002 | 1.60E-11 | 17.54199873 |
| 83 | rs12429545 | A | G | 0.1392 | 0.1248 | 0.0316 | 0.0025 | 9.60E-38 | 34.90313066 |
| 84 | rs12448257 | A | G | 0.1624 | 0.218 | 0.0184 | 0.002 | 8.10E-20 | 28.85917148 |
| 85 | rs12546578 | A | T | 0.7357 | 0.7246 | 0.0146 | 0.002 | 1.00E-13 | 21.2691495 |
| 86 | rs12564992 | G | A | 0.0823 | 0.1144 | 0.0196 | 0.0026 | 5.30E-14 | 11.51500874 |
| 87 | rs12593036 | G | A | 0.2814 | 0.2993 | -0.0154 | 0.0019 | 3.80E-16 | 27.55619487 |
| 88 | rs12602912 | T | C | 0.2378 | 0.2048 | 0.0176 | 0.0021 | 9.90E-18 | 22.87887828 |
| 89 | rs1260326 | C | T | 0.6493 | 0.5973 | 0.0105 | 0.0017 | 3.90E-10 | 18.35244572 |
| 90 | rs12629015 | G | A | 0.2564 | 0.1852 | -0.0135 | 0.0023 | 2.10E-09 | 10.39774448 |
| 91 | rs1266874 | G | A | 0.483 | 0.3558 | 0.014 | 0.0018 | 9.80E-15 | 27.7321725 |
| 92 | rs1268065 | A | G | 0.4839 | 0.4794 | -0.0102 | 0.0017 | 1.00E-09 | 17.96982386 |
| 93 | rs12680842 | G | A | 0.4154 | 0.3205 | -0.0133 | 0.0018 | 4.40E-14 | 23.7803285 |
| 94 | rs12718572 | T | C | 0.3173 | 0.4024 | -0.0117 | 0.0018 | 3.00E-11 | 20.32061554 |
| 95 | rs12779328 | T | C | 0.2248 | 0.2833 | 0.0105 | 0.0019 | 4.50E-08 | 12.40200525 |
| 96 | rs1285997 | G | C | 0.7365 | 0.7153 | 0.0142 | 0.0019 | 1.20E-13 | 22.75036376 |
| 97 | rs12888545 | G | A | 0.2352 | 0.2519 | 0.0136 | 0.002 | 9.10E-12 | 17.4279126 |
| 98 | rs12888955 | A | G | 0.626 | 0.6513 | -0.0178 | 0.0018 | 1.40E-22 | 44.42062282 |
| 99 | rs12905439 | G | C | 0.3414 | 0.3393 | -0.0118 | 0.0018 | 1.40E-10 | 19.26851625 |
| 100 | rs12922346 | C | G | 0.2047 | 0.2657 | 0.0136 | 0.002 | 1.00E-11 | 18.04359855 |
| 101 | rs12936083 | G | A | 0.4132 | 0.3267 | 0.0139 | 0.0019 | 4.10E-13 | 23.54642136 |
| 102 | rs12939549 | G | A | 0.3589 | 0.4335 | -0.018 | 0.0016 | 2.70E-28 | 62.16657868 |
| 103 | rs1296328 | C | A | 0.482 | 0.5657 | -0.0179 | 0.0018 | 4.90E-24 | 48.5955679 |
| 104 | rs12981256 | A | G | 0.4378 | 0.5325 | 0.0142 | 0.0018 | 1.10E-15 | 30.98713755 |
| 105 | rs13021737 | G | A | 0.8368 | 0.8319 | 0.0574 | 0.0021 | 7.50E-157 | 209.0103935 |
| 106 | rs13047416 | G | C | 0.4892 | 0.3769 | -0.0154 | 0.0018 | 2.20E-17 | 34.38198124 |
| 107 | rs13107325 | T | C | 0.01403 | 0.07373 | 0.047 | 0.0032 | 1.10E-47 | 29.46609924 |
| 108 | rs13110266 | A | G | 0.4133 | 0.4065 | -0.0117 | 0.0017 | 1.90E-12 | 22.85580905 |
| 109 | rs13132853 | G | A | 0.3787 | 0.3452 | -0.0142 | 0.0018 | 4.70E-15 | 28.13570239 |
| 110 | rs13147390 | C | T | 0.3055 | 0.3569 | 0.0103 | 0.0018 | 1.00E-08 | 15.03117054 |
| 111 | rs13174863 | G | A | 0.15 | 0.1548 | 0.0192 | 0.0023 | 2.90E-16 | 18.23544384 |
| 112 | rs13184896 | T | G | 0.437 | 0.4346 | -0.0133 | 0.0016 | 3.30E-16 | 33.95911007 |
| 113 | rs13191362 | G | A | 0.06089 | 0.1198 | -0.0236 | 0.0025 | 5.90E-21 | 18.79409282 |
| 114 | rs1320903 | A | G | 0.2947 | 0.3174 | 0.0216 | 0.0018 | 9.20E-32 | 62.40273539 |
| 115 | rs1321432 | C | A | 0.6753 | 0.6321 | 0.0201 | 0.0018 | 3.50E-29 | 58.00001485 |
| 116 | rs13250058 | T | G | 0.6749 | 0.6771 | 0.0112 | 0.0018 | 2.90E-10 | 16.9297334 |
| 117 | rs13263601 | C | A | 0.2681 | 0.3478 | 0.0154 | 0.0018 | 2.20E-17 | 33.20905757 |
| 118 | rs1327259 | G | A | 0.4602 | 0.3872 | -0.0155 | 0.0018 | 1.70E-18 | 35.1903431 |
| 119 | rs13287131 | C | T | 0.2807 | 0.2491 | 0.0123 | 0.002 | 6.80E-10 | 14.14958738 |
| 120 | rs1330052 | G | C | 0.4396 | 0.3504 | 0.0132 | 0.0018 | 1.50E-13 | 24.48257419 |
| 121 | rs13329567 | T | C | 0.1613 | 0.2308 | -0.0293 | 0.002 | 1.00E-50 | 76.21157153 |
| 122 | rs1365466 | T | C | 0.8021 | 0.7406 | -0.0137 | 0.0019 | 3.30E-13 | 19.97687107 |
| 123 | rs1371108 | A | C | 0.3007 | 0.3247 | 0.0119 | 0.0018 | 9.00E-11 | 19.16764851 |
| 124 | rs138289 | T | A | 0.3732 | 0.4829 | -0.0103 | 0.0017 | 3.30E-09 | 18.33363864 |
| 125 | rs1412235 | C | G | 0.3758 | 0.3175 | 0.0246 | 0.0017 | 6.00E-45 | 90.76063712 |
| 126 | rs1421334 | C | A | 0.5905 | 0.5431 | -0.0125 | 0.0018 | 1.00E-12 | 23.93425794 |
| 127 | rs1430387 | C | T | 0.5086 | 0.4295 | -0.0114 | 0.0017 | 5.80E-11 | 22.03805674 |
| 128 | rs1431659 | G | A | 0.7588 | 0.7344 | -0.0196 | 0.0019 | 6.00E-24 | 41.51648303 |
| 129 | rs1436344 | C | G | 0.6336 | 0.5922 | 0.0141 | 0.0017 | 4.10E-16 | 33.228107 |
| 130 | rs1452075 | T | C | 0.7074 | 0.7277 | 0.0141 | 0.0018 | 1.30E-14 | 24.31843341 |
| 131 | rs1454687 | G | C | 0.5557 | 0.5227 | -0.0202 | 0.0017 | 5.20E-32 | 70.45661382 |
| 132 | rs1465900 | C | A | 0.2089 | 0.2188 | -0.0125 | 0.002 | 4.80E-10 | 13.35383194 |
| 133 | rs1472169 | T | C | 0.3083 | 0.3772 | -0.0139 | 0.0018 | 2.80E-15 | 28.01891099 |
| 134 | rs1476322 | A | G | 0.4948 | 0.569 | 0.0101 | 0.0017 | 5.00E-09 | 17.31306821 |
| 135 | rs1477199 | G | A | 0.1145 | 0.1451 | 0.0228 | 0.0024 | 9.40E-22 | 22.39087588 |
| 136 | rs1492767 | T | C | 0.4211 | 0.4957 | 0.0094 | 0.0016 | 1.00E-08 | 17.25686763 |
| 137 | rs1503526 | C | T | 0.5754 | 0.4838 | 0.014 | 0.0017 | 5.50E-17 | 33.87588202 |
| 138 | rs1521527 | C | G | 0.5677 | 0.5324 | -0.0121 | 0.0017 | 3.10E-12 | 25.22495009 |
| 139 | rs1522569 | G | T | 0.08679 | 0.1819 | -0.0164 | 0.0022 | 2.90E-13 | 16.53943154 |
| 140 | rs1528435 | T | C | 0.6393 | 0.6331 | 0.0164 | 0.0017 | 9.10E-23 | 43.23768407 |
| 141 | rs1538247 | C | T | 0.3222 | 0.3181 | 0.0108 | 0.0019 | 1.00E-08 | 14.01723352 |
| 142 | rs1552893 | G | A | 0.2316 | 0.2777 | -0.0126 | 0.0019 | 8.10E-11 | 17.64278759 |
| 143 | rs156201 | C | G | 0.6265 | 0.7606 | 0.0123 | 0.002 | 5.80E-10 | 13.77425395 |
| 144 | rs1624134 | C | G | 0.4782 | 0.4068 | 0.0101 | 0.0018 | 1.10E-08 | 15.19560928 |
| 145 | rs1656377 | C | T | 0.5593 | 0.5885 | 0.0099 | 0.0017 | 1.60E-08 | 16.4258519 |
| 146 | rs1681740 | C | A | 0.3642 | 0.3933 | -0.0115 | 0.0018 | 1.10E-10 | 19.48004159 |
| 147 | rs16849710 | G | A | 0.5273 | 0.515 | -0.0116 | 0.0018 | 6.00E-11 | 20.74732701 |
| 148 | rs16851483 | T | G | 0.0485 | 0.06928 | 0.0369 | 0.0035 | 3.20E-26 | 14.33445885 |
| 149 | rs16871902 | A | G | 0.5061 | 0.4877 | 0.0125 | 0.0017 | 4.60E-13 | 27.01749113 |
| 150 | rs16903285 | C | T | 0.1079 | 0.1407 | 0.0331 | 0.0026 | 7.60E-38 | 39.19238342 |
| 151 | rs17001561 | A | G | 0.1162 | 0.1573 | 0.0151 | 0.0023 | 3.80E-11 | 11.42707529 |
| 152 | rs17014375 | G | T | 0.1046 | 0.1348 | 0.0172 | 0.0025 | 1.10E-11 | 11.04126818 |
| 153 | rs17033117 | T | C | 0.166 | 0.1872 | 0.0137 | 0.0022 | 8.90E-10 | 11.801072 |
| 154 | rs17056301 | C | T | 0.2228 | 0.2636 | 0.0118 | 0.002 | 2.40E-09 | 13.51451523 |
| 155 | rs17113297 | T | C | 0.2304 | 0.2082 | 0.0166 | 0.0021 | 2.10E-15 | 20.60229376 |
| 156 | rs17203016 | G | A | 0.2038 | 0.196 | 0.015 | 0.002 | 2.10E-13 | 17.72855463 |
| 157 | rs17207196 | T | C | 0.5155 | 0.4118 | -0.0221 | 0.0018 | 2.10E-35 | 73.03432394 |
| 158 | rs17238110 | G | A | 0.1049 | 0.1634 | -0.0353 | 0.005 | 2.00E-12 | 13.62748842 |
| 159 | rs17311369 | T | C | 0.296 | 0.3278 | -0.0104 | 0.0019 | 3.10E-08 | 13.20395874 |
| 160 | rs17399237 | C | T | 0.6141 | 0.5497 | -0.0129 | 0.0017 | 6.70E-14 | 28.50728898 |
| 161 | rs17405819 | C | T | 0.2831 | 0.301 | -0.0215 | 0.0018 | 4.30E-33 | 60.03952695 |
| 162 | rs17424296 | A | G | 0.326 | 0.3659 | -0.0108 | 0.0018 | 2.40E-09 | 16.70559664 |
| 163 | rs17513613 | C | T | 0.3531 | 0.3236 | 0.0186 | 0.0018 | 3.60E-26 | 46.74633824 |
| 164 | rs175165 | G | T | 0.4478 | 0.3941 | -0.0103 | 0.0018 | 5.20E-09 | 15.63779081 |
| 165 | rs17551974 | A | C | 0.2135 | 0.1782 | -0.0141 | 0.0022 | 1.90E-10 | 12.03103525 |
| 166 | rs17710386 | C | T | 0.3938 | 0.3319 | 0.0126 | 0.0018 | 1.00E-12 | 21.73130126 |
| 167 | rs17724992 | G | A | 0.2244 | 0.2596 | -0.0183 | 0.0019 | 1.00E-22 | 35.6627373 |
| 168 | rs17789218 | C | T | 0.2084 | 0.2392 | 0.013 | 0.0019 | 7.40E-12 | 17.039208 |
| 169 | rs17806379 | T | C | 0.1567 | 0.1789 | -0.0258 | 0.0022 | 1.50E-30 | 40.40681416 |
| 170 | rs1784460 | A | T | 0.296 | 0.4035 | 0.0132 | 0.0018 | 9.00E-14 | 25.88821402 |
| 171 | rs1804528 | A | G | 0.4003 | 0.3507 | 0.0109 | 0.002 | 3.00E-08 | 13.52738397 |
| 172 | rs1830074 | C | T | 0.3159 | 0.288 | 0.0115 | 0.0019 | 1.40E-09 | 15.02446978 |
| 173 | rs1836303 | G | A | 0.3231 | 0.3873 | 0.0116 | 0.0018 | 5.30E-11 | 19.71094767 |
| 174 | rs1843328 | A | C | 0.5003 | 0.5085 | -0.0099 | 0.0017 | 7.90E-09 | 16.95221595 |
| 175 | rs1863652 | A | G | 0.2969 | 0.3449 | -0.0115 | 0.0018 | 1.40E-10 | 18.44555695 |
| 176 | rs1884389 | T | C | 0.4338 | 0.4289 | -0.0103 | 0.0017 | 4.00E-09 | 17.98394486 |
| 177 | rs1885728 | A | G | 0.6835 | 0.6787 | 0.0108 | 0.0019 | 1.00E-08 | 14.09180341 |
| 178 | rs1891216 | G | T | 0.4197 | 0.3759 | 0.0107 | 0.0018 | 2.40E-09 | 16.58014376 |
| 179 | rs1896767 | A | G | 0.4936 | 0.5376 | -0.0109 | 0.0017 | 2.40E-10 | 20.43967111 |
| 180 | rs189843 | C | G | 0.61 | 0.5557 | -0.0098 | 0.0017 | 1.70E-08 | 16.41005914 |
| 181 | rs1927790 | C | T | 0.4849 | 0.4109 | 0.0148 | 0.0016 | 1.80E-19 | 41.42477707 |
| 182 | rs1928295 | C | T | 0.5131 | 0.4461 | -0.0141 | 0.0016 | 5.40E-18 | 38.38059927 |
| 183 | rs1937683 | T | C | 0.6631 | 0.6699 | 0.0109 | 0.0018 | 3.20E-09 | 16.21819059 |
| 184 | rs1948080 | G | T | 0.3026 | 0.3749 | -0.0137 | 0.0018 | 1.10E-14 | 27.15231576 |
| 185 | rs1982441 | T | G | 0.1843 | 0.1381 | 0.0175 | 0.0026 | 7.00E-12 | 10.78488464 |
| 186 | rs1982725 | T | C | 0.462 | 0.4778 | 0.0097 | 0.0017 | 3.30E-08 | 16.24679465 |
| 187 | rs1993709 | G | A | 0.8598 | 0.8177 | 0.0331 | 0.0021 | 1.90E-57 | 74.0743556 |
| 188 | rs2007231 | T | C | 0.6918 | 0.6387 | -0.0104 | 0.0018 | 5.20E-09 | 15.40724386 |
| 189 | rs200810 | C | T | 0.3907 | 0.3716 | -0.0136 | 0.0017 | 5.50E-16 | 29.89077065 |
| 190 | rs2009416 | T | C | 0.4304 | 0.361 | -0.0121 | 0.0018 | 1.10E-11 | 20.84853868 |
| 191 | rs2033529 | G | A | 0.3292 | 0.2936 | 0.0205 | 0.0018 | 1.90E-30 | 53.80564627 |
| 192 | rs2051559 | C | T | 0.1104 | 0.1308 | 0.0176 | 0.0026 | 5.00E-12 | 10.41936859 |
| 193 | rs2065418 | G | T | 0.3085 | 0.3623 | -0.0166 | 0.0018 | 3.60E-20 | 39.30151863 |
| 194 | rs208015 | C | T | 0.8924 | 0.9216 | -0.0356 | 0.0034 | 1.40E-25 | 15.8430714 |
| 195 | rs2124499 | C | G | 0.3562 | 0.3718 | -0.0123 | 0.0017 | 3.40E-13 | 24.45468657 |
| 196 | rs2143253 | A | G | 0.09932 | 0.1189 | -0.0188 | 0.0026 | 1.10E-12 | 10.95498365 |
| 197 | rs215634 | G | A | 0.7146 | 0.6212 | -0.0152 | 0.0018 | 2.60E-17 | 33.56090758 |
| 198 | rs2162524 | C | T | 0.3033 | 0.3321 | 0.0155 | 0.0018 | 4.10E-17 | 32.89638005 |
| 199 | rs2163188 | C | G | 0.4841 | 0.474 | 0.0131 | 0.0017 | 2.00E-14 | 29.61121974 |
| 200 | rs2174307 | C | G | 0.4921 | 0.4067 | 0.0121 | 0.0017 | 4.90E-12 | 24.44925413 |
| 201 | rs217671 | G | A | 0.2114 | 0.2719 | 0.0144 | 0.0019 | 1.30E-13 | 22.74369618 |
| 202 | rs2228213 | A | G | 0.346 | 0.3481 | -0.0139 | 0.0017 | 4.60E-16 | 30.34325936 |
| 203 | rs2235564 | T | C | 0.347 | 0.3466 | 0.0131 | 0.0018 | 3.70E-13 | 23.99104004 |
| 204 | rs2246012 | C | T | 0.2311 | 0.1628 | 0.0158 | 0.0022 | 3.10E-13 | 14.06011948 |
| 205 | rs2283093 | T | C | 0.1874 | 0.2066 | 0.0127 | 0.0021 | 3.10E-09 | 11.99023329 |
| 206 | rs2284746 | G | C | 0.5055 | 0.5238 | -0.0104 | 0.0017 | 1.40E-09 | 18.6708537 |
| 207 | rs2285178 | C | T | 0.3228 | 0.3109 | 0.0112 | 0.0019 | 9.40E-09 | 14.88917317 |
| 208 | rs2306537 | G | A | 0.2633 | 0.3092 | 0.0133 | 0.0019 | 8.70E-13 | 20.93291805 |
| 209 | rs2307111 | C | T | 0.4272 | 0.3962 | -0.0265 | 0.0016 | 1.60E-58 | 131.2683212 |
| 210 | rs2317299 | C | T | 0.5072 | 0.5597 | -0.0106 | 0.0017 | 1.30E-09 | 19.16279571 |
| 211 | rs2325036 | C | A | 0.4579 | 0.3845 | -0.0181 | 0.0017 | 3.60E-27 | 53.65893773 |
| 212 | rs2357760 | A | G | 0.6652 | 0.6754 | 0.0145 | 0.0017 | 6.80E-17 | 31.90025424 |
| 213 | rs2361988 | C | T | 0.2033 | 0.2539 | -0.0155 | 0.002 | 5.20E-15 | 22.75653844 |
| 214 | rs2365389 | T | C | 0.4379 | 0.4143 | -0.0174 | 0.0017 | 1.30E-25 | 50.84495607 |
| 215 | rs2367112 | G | T | 0.5298 | 0.4919 | -0.0119 | 0.0016 | 2.30E-13 | 27.65183748 |
| 216 | rs2423668 | C | T | 0.562 | 0.5505 | -0.0105 | 0.0019 | 2.80E-08 | 15.11468886 |
| 217 | rs2425840 | C | A | 0.3355 | 0.4059 | 0.0119 | 0.0018 | 1.60E-11 | 21.07995442 |
| 218 | rs2429150 | C | A | 0.4553 | 0.4164 | 0.0111 | 0.0018 | 2.70E-10 | 18.48278356 |
| 219 | rs2479958 | G | A | 0.5083 | 0.5075 | -0.0154 | 0.0018 | 1.50E-17 | 36.5924362 |
| 220 | rs2481665 | C | T | 0.3672 | 0.4408 | -0.0161 | 0.0016 | 7.20E-23 | 49.92024407 |
| 221 | rs2543132 | C | G | 0.8422 | 0.8134 | 0.0146 | 0.0022 | 5.00E-11 | 13.36944338 |
| 222 | rs2600226 | T | C | 0.6344 | 0.6697 | -0.0116 | 0.0019 | 3.70E-10 | 16.49061407 |
| 223 | rs2605603 | A | G | 0.517 | 0.4887 | -0.0103 | 0.0016 | 2.50E-10 | 20.71060979 |
| 224 | rs2608703 | A | C | 0.4411 | 0.4546 | 0.0142 | 0.0017 | 1.90E-16 | 34.59983466 |
| 225 | rs2643452 | A | T | 0.5925 | 0.5448 | 0.0136 | 0.0017 | 4.70E-15 | 31.74446612 |
| 226 | rs2693826 | A | G | 0.5091 | 0.4421 | -0.0137 | 0.0017 | 2.00E-15 | 32.03826929 |
| 227 | rs2694047 | G | A | 0.7075 | 0.747 | 0.0188 | 0.002 | 3.90E-21 | 33.40000874 |
| 228 | rs273504 | G | A | 0.4898 | 0.4266 | 0.0153 | 0.0018 | 4.40E-18 | 35.34820424 |
| 229 | rs2744974 | T | C | 0.3332 | 0.338 | 0.0249 | 0.0018 | 1.40E-45 | 85.64546482 |
| 230 | rs2791653 | G | A | 0.7722 | 0.7577 | -0.0141 | 0.0019 | 1.30E-13 | 20.22188663 |
| 231 | rs2820311 | G | A | 0.2943 | 0.3369 | 0.0235 | 0.0018 | 4.10E-38 | 76.1635712 |
| 232 | rs2832283 | A | G | 0.258 | 0.2208 | 0.0115 | 0.002 | 5.80E-09 | 11.37676632 |
| 233 | rs2836964 | C | T | 0.2352 | 0.3576 | -0.011 | 0.0018 | 1.30E-09 | 17.15864174 |
| 234 | rs2861683 | C | A | 0.4126 | 0.407 | -0.0144 | 0.0017 | 1.30E-16 | 34.63592145 |
| 235 | rs2868975 | A | G | 0.2329 | 0.178 | -0.0143 | 0.0023 | 2.20E-10 | 11.31212184 |
| 236 | rs287104 | A | G | 0.5618 | 0.6604 | 0.0115 | 0.0017 | 4.40E-11 | 20.52640054 |
| 237 | rs2875762 | C | G | 0.111 | 0.2473 | 0.0139 | 0.002 | 1.20E-11 | 17.98273595 |
| 238 | rs2907948 | A | G | 0.1691 | 0.2427 | -0.0141 | 0.0019 | 1.30E-13 | 20.24457797 |
| 239 | rs2931434 | T | C | 0.2969 | 0.3168 | -0.0104 | 0.0018 | 1.40E-08 | 14.45082107 |
| 240 | rs294704 | T | G | 0.7639 | 0.7239 | -0.0113 | 0.0019 | 4.00E-09 | 14.13944309 |
| 241 | rs3007105 | T | C | 0.3519 | 0.4697 | 0.0142 | 0.0017 | 1.10E-17 | 34.75914947 |
| 242 | rs326896 | T | C | 0.3296 | 0.3925 | -0.0128 | 0.0018 | 2.80E-13 | 24.11597258 |
| 243 | rs331966 | C | A | 0.3658 | 0.3792 | 0.0112 | 0.0018 | 3.20E-10 | 18.22851642 |
| 244 | rs33500 | T | C | 0.8128 | 0.8082 | -0.0167 | 0.0022 | 4.30E-14 | 17.8646708 |
| 245 | rs339991 | G | A | 0.6164 | 0.5631 | 0.0124 | 0.0018 | 1.20E-12 | 23.35121246 |
| 246 | rs349088 | A | C | 0.6104 | 0.4976 | -0.0128 | 0.0017 | 1.80E-13 | 28.34645884 |
| 247 | rs355777 | C | G | 0.4327 | 0.4106 | 0.0153 | 0.0017 | 1.40E-18 | 39.20735184 |
| 248 | rs3731695 | C | T | 0.451 | 0.5582 | 0.0116 | 0.0016 | 7.90E-13 | 25.92594802 |
| 249 | rs3732084 | C | T | 0.5194 | 0.6139 | 0.0107 | 0.0018 | 1.10E-09 | 16.75171351 |
| 250 | rs3736485 | G | A | 0.5874 | 0.5443 | -0.0134 | 0.0016 | 2.50E-16 | 34.79645571 |
| 251 | rs3749897 | T | C | 0.4497 | 0.4172 | 0.0122 | 0.0018 | 8.40E-12 | 22.33995687 |
| 252 | rs3754963 | T | A | 0.3021 | 0.2574 | -0.0123 | 0.002 | 3.30E-10 | 14.45944219 |
| 253 | rs3772882 | A | C | 0.4509 | 0.3661 | 0.0127 | 0.0018 | 6.60E-13 | 23.10607377 |
| 254 | rs3800229 | T | G | 0.639 | 0.7123 | 0.0175 | 0.0018 | 1.40E-22 | 38.74217729 |
| 255 | rs3800637 | C | T | 0.3377 | 0.336 | 0.0115 | 0.0018 | 5.10E-10 | 18.21370707 |
| 256 | rs3806114 | A | G | 0.7524 | 0.6773 | -0.0113 | 0.0018 | 3.40E-10 | 17.22782313 |
| 257 | rs3806572 | A | G | 0.2438 | 0.2788 | -0.0145 | 0.0019 | 1.60E-14 | 23.42182948 |
| 258 | rs3807645 | A | G | 0.2032 | 0.221 | -0.0166 | 0.0021 | 2.40E-15 | 21.51541468 |
| 259 | rs3814883 | T | C | 0.4115 | 0.4764 | 0.0232 | 0.0017 | 1.10E-40 | 92.92597223 |
| 260 | rs3828783 | A | G | 0.2285 | 0.1809 | -0.0165 | 0.0021 | 5.60E-15 | 18.29547606 |
| 261 | rs3829849 | T | C | 0.3708 | 0.3589 | 0.0098 | 0.0017 | 5.90E-09 | 15.29293391 |
| 262 | rs38314 | A | G | 0.5295 | 0.4912 | -0.012 | 0.0017 | 4.70E-12 | 24.90660469 |
| 263 | rs3844598 | G | A | 0.5649 | 0.521 | 0.0095 | 0.0017 | 3.80E-08 | 15.58695003 |
| 264 | rs391300 | C | T | 0.6282 | 0.6275 | -0.0119 | 0.0017 | 3.10E-12 | 22.90749288 |
| 265 | rs3935648 | G | C | 0.1785 | 0.2328 | -0.0125 | 0.0022 | 6.80E-09 | 11.53195153 |
| 266 | rs3977755 | T | C | 0.2057 | 0.2804 | -0.0135 | 0.0019 | 5.90E-13 | 20.37372326 |
| 267 | rs40067 | A | G | 0.2093 | 0.1713 | -0.0266 | 0.0023 | 7.10E-30 | 37.9765246 |
| 268 | rs4012234 | G | T | 0.6477 | 0.5924 | 0.0141 | 0.0018 | 9.90E-16 | 29.63397004 |
| 269 | rs4072917 | A | G | 0.4393 | 0.4694 | 0.0115 | 0.0018 | 6.90E-11 | 20.33305451 |
| 270 | rs4148155 | G | A | 0.07481 | 0.1127 | -0.0188 | 0.0026 | 5.00E-13 | 10.45678109 |
| 271 | rs4148866 | T | C | 0.401 | 0.4068 | 0.0098 | 0.0018 | 4.00E-08 | 14.30629331 |
| 272 | rs4237643 | G | T | 0.6941 | 0.6938 | -0.0223 | 0.0019 | 4.30E-33 | 58.53389921 |
| 273 | rs427943 | C | A | 0.582 | 0.5669 | 0.017 | 0.0017 | 7.30E-23 | 49.1081265 |
| 274 | rs429343 | G | A | 0.5313 | 0.5813 | -0.015 | 0.0017 | 6.80E-18 | 37.90011884 |
| 275 | rs4307239 | G | A | 0.453 | 0.4578 | 0.0115 | 0.0017 | 3.90E-11 | 22.71832075 |
| 276 | rs4358081 | C | A | 0.4486 | 0.4631 | 0.0097 | 0.0017 | 1.50E-08 | 16.19021952 |
| 277 | rs4414033 | A | G | 0.5718 | 0.627 | 0.0129 | 0.0018 | 1.40E-12 | 24.02453539 |
| 278 | rs4430672 | C | T | 0.7904 | 0.8004 | -0.0127 | 0.0022 | 3.90E-09 | 10.64792847 |
| 279 | rs4482463 | A | C | 0.9404 | 0.9213 | -0.0331 | 0.0033 | 2.80E-23 | 14.58957066 |
| 280 | rs4516268 | A | C | 0.2549 | 0.1925 | -0.0217 | 0.0021 | 5.20E-25 | 33.19719294 |
| 281 | rs4518345 | A | G | 0.2435 | 0.2842 | -0.0117 | 0.0019 | 1.00E-09 | 15.42832411 |
| 282 | rs4556997 | A | C | 0.1382 | 0.1349 | 0.0197 | 0.0024 | 6.90E-17 | 15.72627058 |
| 283 | rs4639527 | G | A | 0.3754 | 0.3012 | 0.0172 | 0.0019 | 3.30E-20 | 34.49911512 |
| 284 | rs4653017 | T | C | 0.6656 | 0.6818 | 0.0122 | 0.0018 | 4.50E-11 | 19.9330229 |
| 285 | rs4660443 | T | C | 0.1892 | 0.2218 | 0.0164 | 0.0021 | 6.80E-15 | 21.05445053 |
| 286 | rs4740619 | C | T | 0.5114 | 0.4521 | -0.0186 | 0.0016 | 2.30E-30 | 66.95565018 |
| 287 | rs4757144 | A | G | 0.5086 | 0.5878 | 0.0169 | 0.0018 | 5.60E-22 | 42.71905029 |
| 288 | rs4783830 | A | G | 0.319 | 0.3074 | -0.0105 | 0.0019 | 2.40E-08 | 13.00453468 |
| 289 | rs4786903 | G | A | 0.7578 | 0.7368 | 0.0125 | 0.002 | 3.50E-10 | 15.15074294 |
| 290 | rs4800191 | C | G | 0.6433 | 0.6369 | 0.0103 | 0.0017 | 2.50E-09 | 16.97901075 |
| 291 | rs4813619 | T | G | 0.5576 | 0.5101 | -0.0108 | 0.0018 | 2.30E-09 | 17.99311735 |
| 292 | rs4818225 | G | A | 0.5858 | 0.6606 | 0.0117 | 0.0018 | 2.30E-10 | 18.94601204 |
| 293 | rs4820408 | G | T | 0.6014 | 0.592 | -0.0151 | 0.0017 | 2.10E-19 | 38.11427522 |
| 294 | rs4842491 | T | C | 0.6948 | 0.7138 | 0.0098 | 0.0018 | 4.00E-08 | 12.11124616 |
| 295 | rs4851029 | G | T | 0.4981 | 0.5247 | 0.0121 | 0.0017 | 1.70E-12 | 25.26948687 |
| 296 | rs4858193 | C | T | 0.2628 | 0.2779 | -0.0129 | 0.0019 | 1.60E-11 | 18.50114214 |
| 297 | rs486359 | C | G | 0.4966 | 0.4853 | 0.0112 | 0.0017 | 1.60E-11 | 21.68421704 |
| 298 | rs4864201 | C | T | 0.6175 | 0.6469 | -0.0141 | 0.0017 | 1.50E-16 | 31.42833093 |
| 299 | rs4880341 | T | C | 0.5116 | 0.5606 | -0.0118 | 0.0017 | 1.10E-11 | 23.73684612 |
| 300 | rs4906908 | G | T | 0.5129 | 0.5253 | 0.0103 | 0.0017 | 2.50E-09 | 18.30810857 |
| 301 | rs491711 | C | A | 0.2317 | 0.316 | -0.0115 | 0.0019 | 1.10E-09 | 15.83690932 |
| 302 | rs4929923 | C | T | 0.6472 | 0.6376 | 0.0181 | 0.0017 | 7.20E-27 | 52.39059479 |
| 303 | rs4936175 | C | T | 0.3583 | 0.4445 | 0.0122 | 0.0017 | 1.40E-12 | 25.43444924 |
| 304 | rs4937870 | G | A | 0.4145 | 0.3172 | -0.0109 | 0.0019 | 8.80E-09 | 14.25641314 |
| 305 | rs4952843 | G | A | 0.3389 | 0.3807 | -0.0131 | 0.0018 | 6.80E-14 | 24.97617582 |
| 306 | rs4954638 | C | A | 0.3432 | 0.2492 | -0.0118 | 0.002 | 2.90E-09 | 13.0260656 |
| 307 | rs4968656 | G | A | 0.3079 | 0.3216 | 0.0116 | 0.0019 | 8.20E-10 | 16.2648412 |
| 308 | rs4981693 | A | G | 0.6705 | 0.771 | 0.0206 | 0.002 | 6.90E-24 | 37.46421654 |
| 309 | rs4986044 | T | C | 0.4992 | 0.4687 | -0.0164 | 0.0016 | 3.30E-23 | 52.32873792 |
| 310 | rs538579 | C | G | 0.2647 | 0.3228 | 0.0137 | 0.0019 | 1.30E-13 | 22.73146789 |
| 311 | rs543874 | G | A | 0.1782 | 0.1952 | 0.0475 | 0.002 | 1.20E-122 | 177.2640539 |
| 312 | rs559231 | T | G | 0.4233 | 0.3956 | 0.0135 | 0.0018 | 2.40E-14 | 26.89979955 |
| 313 | rs577525 | C | T | 0.511 | 0.5676 | 0.0166 | 0.0017 | 9.70E-22 | 46.80632854 |
| 314 | rs592483 | T | C | 0.4447 | 0.5716 | -0.0147 | 0.0017 | 2.00E-18 | 36.62079217 |
| 315 | rs6011457 | A | T | 0.3978 | 0.4975 | -0.0116 | 0.0017 | 2.70E-11 | 23.28041206 |
| 316 | rs6235 | G | C | 0.2855 | 0.2702 | 0.0175 | 0.0019 | 1.50E-19 | 33.45860624 |
| 317 | rs6265 | T | C | 0.1542 | 0.1951 | -0.0412 | 0.0021 | 1.00E-86 | 120.9065835 |
| 318 | rs6443750 | C | T | 0.7894 | 0.8068 | 0.0148 | 0.0021 | 3.20E-12 | 15.48443585 |
| 319 | rs6448587 | C | A | 0.2252 | 0.1891 | -0.0167 | 0.0023 | 2.30E-13 | 16.16869612 |
| 320 | rs645040 | T | G | 0.8509 | 0.7762 | 0.0171 | 0.002 | 2.50E-18 | 25.39853801 |
| 321 | rs6461115 | G | A | 0.3025 | 0.2285 | -0.0144 | 0.0019 | 1.20E-13 | 20.25255988 |
| 322 | rs6471941 | A | G | 0.3267 | 0.1684 | 0.0156 | 0.0021 | 3.10E-13 | 15.45626414 |
| 323 | rs6500208 | A | G | 0.3117 | 0.2006 | 0.014 | 0.002 | 4.10E-12 | 15.71552037 |
| 324 | rs6512302 | C | G | 0.7851 | 0.7511 | 0.0142 | 0.002 | 2.10E-12 | 18.8486399 |
| 325 | rs6545714 | A | G | 0.5995 | 0.6139 | -0.0191 | 0.0017 | 9.10E-31 | 59.8450158 |
| 326 | rs6556301 | T | G | 0.3707 | 0.3596 | -0.0111 | 0.0018 | 4.10E-10 | 17.51503945 |
| 327 | rs6561943 | T | C | 0.2634 | 0.2595 | 0.0119 | 0.0019 | 4.20E-10 | 15.07600574 |
| 328 | rs657452 | G | A | 0.5737 | 0.6216 | -0.0188 | 0.0017 | 7.20E-29 | 57.53622912 |
| 329 | rs6587552 | G | A | 0.7277 | 0.7591 | -0.0173 | 0.002 | 1.60E-17 | 27.36616706 |
| 330 | rs6593688 | G | A | 0.384 | 0.3733 | 0.0137 | 0.0018 | 8.60E-15 | 27.10563371 |
| 331 | rs6595205 | G | C | 0.5411 | 0.5305 | -0.0114 | 0.0016 | 2.00E-12 | 25.28910431 |
| 332 | rs663129 | A | G | 0.1841 | 0.2301 | 0.0545 | 0.0019 | 1.60E-178 | 291.6259312 |
| 333 | rs6673081 | C | T | 0.5956 | 0.5534 | -0.01 | 0.0018 | 1.80E-08 | 15.25637491 |
| 334 | rs6692586 | G | A | 0.8848 | 0.832 | -0.0192 | 0.0023 | 1.10E-16 | 19.48140959 |
| 335 | rs6764533 | A | G | 0.3033 | 0.359 | 0.0116 | 0.0018 | 1.40E-10 | 19.11455541 |
| 336 | rs6772756 | G | A | 0.3606 | 0.3372 | -0.0104 | 0.0019 | 4.00E-08 | 13.39265853 |
| 337 | rs6785245 | C | T | 0.3505 | 0.3969 | 0.0132 | 0.0017 | 4.00E-14 | 28.8647165 |
| 338 | rs6804842 | G | A | 0.5345 | 0.572 | 0.0156 | 0.0017 | 3.60E-21 | 41.23279142 |
| 339 | rs6815910 | A | T | 0.5166 | 0.5435 | -0.0128 | 0.0017 | 1.40E-13 | 28.1325363 |
| 340 | rs6841761 | T | G | 0.6101 | 0.5252 | -0.0131 | 0.0016 | 6.40E-16 | 33.43376255 |
| 341 | rs685870 | C | T | 0.6893 | 0.7035 | 0.012 | 0.0019 | 2.40E-10 | 16.64114948 |
| 342 | rs6985109 | A | G | 0.4264 | 0.5338 | -0.0177 | 0.0017 | 1.50E-26 | 53.95826084 |
| 343 | rs7024334 | G | T | 0.7922 | 0.7742 | -0.0138 | 0.002 | 3.10E-12 | 16.64613495 |
| 344 | rs7025938 | G | C | 0.3884 | 0.3187 | 0.0166 | 0.0019 | 3.70E-19 | 33.14965271 |
| 345 | rs7037266 | A | C | 0.4026 | 0.3739 | -0.0112 | 0.0018 | 3.50E-10 | 18.12718332 |
| 346 | rs705217 | G | T | 0.3613 | 0.3652 | -0.0102 | 0.0018 | 9.30E-09 | 14.88884966 |
| 347 | rs705217 | G | T | 0.002553 | 0.3652 | -0.0102 | 0.0018 | 9.30E-09 | 14.88884966 |
| 348 | rs705704 | A | G | 0.3199 | 0.3304 | -0.0131 | 0.0018 | 1.90E-13 | 23.43665247 |
| 349 | rs7084454 | A | G | 0.3057 | 0.335 | 0.0193 | 0.0019 | 4.00E-25 | 45.9760873 |
| 350 | rs709400 | G | A | 0.3088 | 0.3818 | -0.015 | 0.0017 | 4.60E-19 | 36.75348889 |
| 351 | rs7102454 | C | T | 0.285 | 0.3435 | 0.0158 | 0.0018 | 2.40E-18 | 34.75211266 |
| 352 | rs7124681 | A | C | 0.3972 | 0.4133 | 0.0263 | 0.0016 | 3.20E-58 | 131.0549634 |
| 353 | rs7138803 | A | G | 0.3839 | 0.3772 | 0.03 | 0.0017 | 2.30E-71 | 146.343601 |
| 354 | rs7144011 | T | G | 0.24 | 0.2136 | 0.0282 | 0.002 | 5.20E-47 | 66.79568513 |
| 355 | rs7172627 | G | A | 0.4502 | 0.4719 | 0.0117 | 0.0017 | 1.10E-11 | 23.60932731 |
| 356 | rs7181498 | C | T | 0.5541 | 0.6309 | -0.0163 | 0.0018 | 1.00E-19 | 38.19332905 |
| 357 | rs7196720 | C | T | 0.5207 | 0.5068 | -0.0129 | 0.0017 | 7.30E-14 | 28.78645002 |
| 358 | rs7206608 | G | C | 0.3521 | 0.3146 | 0.0132 | 0.0019 | 1.30E-12 | 20.81542743 |
| 359 | rs7222349 | A | G | 0.2996 | 0.3441 | 0.0115 | 0.0018 | 3.30E-10 | 18.42524514 |
| 360 | rs7239575 | C | T | 0.4941 | 0.4832 | -0.0202 | 0.0017 | 7.40E-32 | 70.52243595 |
| 361 | rs7318817 | T | C | 0.6102 | 0.6071 | -0.0155 | 0.0018 | 2.70E-18 | 35.37623353 |
| 362 | rs7334078 | C | T | 0.2538 | 0.2882 | -0.0121 | 0.0019 | 2.20E-10 | 16.64005398 |
| 363 | rs7358465 | T | C | 0.7105 | 0.6781 | 0.0103 | 0.0019 | 3.00E-08 | 12.82976823 |
| 364 | rs7488867 | T | C | 0.304 | 0.2639 | -0.0204 | 0.002 | 8.40E-24 | 40.42339584 |
| 365 | rs7498665 | G | A | 0.4129 | 0.4038 | 0.0271 | 0.0017 | 5.60E-60 | 122.3756936 |
| 366 | rs7519259 | A | G | 0.5658 | 0.5356 | 0.0125 | 0.0017 | 3.80E-13 | 26.89681322 |
| 367 | rs7535528 | A | G | 0.3535 | 0.3741 | -0.0152 | 0.0018 | 1.40E-16 | 33.39537812 |
| 368 | rs754635 | G | C | 0.8988 | 0.8873 | 0.0198 | 0.0027 | 2.20E-13 | 10.75555322 |
| 369 | rs7551507 | T | C | 0.4917 | 0.5633 | -0.0184 | 0.0016 | 9.30E-30 | 65.0703422 |
| 370 | rs7557796 | C | T | 0.6071 | 0.6524 | -0.016 | 0.0018 | 2.30E-19 | 35.83768056 |
| 371 | rs756717 | A | G | 0.4081 | 0.3973 | -0.0148 | 0.0017 | 5.40E-18 | 36.29899789 |
| 372 | rs7599312 | A | G | 0.3026 | 0.2652 | -0.0186 | 0.0019 | 6.90E-24 | 37.35175496 |
| 373 | rs7626079 | T | C | 0.3047 | 0.3434 | 0.011 | 0.0018 | 1.60E-09 | 16.8415024 |
| 374 | rs7637852 | G | A | 0.5871 | 0.6951 | -0.0139 | 0.0019 | 1.70E-13 | 22.68663549 |
| 375 | rs7640424 | T | C | 0.3544 | 0.2969 | -0.0136 | 0.0018 | 2.30E-14 | 23.83428299 |
| 376 | rs765875 | T | C | 0.5794 | 0.4808 | -0.0121 | 0.0017 | 3.00E-12 | 25.29395125 |
| 377 | rs7683836 | A | G | 0.4764 | 0.5405 | -0.0114 | 0.0017 | 6.30E-11 | 22.33757001 |
| 378 | rs7685048 | T | C | 0.3873 | 0.4654 | -0.0101 | 0.0017 | 4.10E-09 | 17.56467006 |
| 379 | rs768840 | A | G | 0.3678 | 0.4183 | 0.0114 | 0.0018 | 2.00E-10 | 19.52058586 |
| 380 | rs769449 | A | G | 0.1611 | 0.1161 | -0.0254 | 0.0027 | 2.30E-20 | 18.16425701 |
| 381 | rs7694732 | G | A | 0.4557 | 0.4378 | -0.0099 | 0.0017 | 8.70E-09 | 16.69469085 |
| 382 | rs7703576 | C | T | 0.3292 | 0.2885 | 0.0103 | 0.0019 | 4.80E-08 | 12.06491572 |
| 383 | rs7715256 | T | G | 0.5905 | 0.5781 | -0.0166 | 0.0016 | 2.20E-24 | 52.51051562 |
| 384 | rs7724675 | A | G | 0.2648 | 0.2238 | -0.0119 | 0.0021 | 9.50E-09 | 11.15641848 |
| 385 | rs7730004 | T | C | 0.5558 | 0.6693 | 0.0148 | 0.0018 | 9.10E-16 | 29.92822843 |
| 386 | rs7730898 | A | G | 0.6773 | 0.729 | 0.0168 | 0.0018 | 4.50E-20 | 34.42057522 |
| 387 | rs7761673 | A | T | 0.1516 | 0.2058 | -0.0126 | 0.0021 | 1.90E-09 | 11.76830411 |
| 388 | rs7780752 | C | T | 0.2657 | 0.36 | 0.0139 | 0.0018 | 1.00E-14 | 27.47976905 |
| 389 | rs7788008 | A | G | 0.4026 | 0.4445 | -0.0157 | 0.0017 | 1.10E-19 | 42.12234337 |
| 390 | rs7819514 | A | G | 0.3823 | 0.3216 | -0.0107 | 0.0018 | 5.70E-09 | 15.41923859 |
| 391 | rs7826312 | C | T | 0.5765 | 0.5879 | 0.0104 | 0.0017 | 4.90E-10 | 18.13484419 |
| 392 | rs7844647 | C | T | 0.2431 | 0.2681 | -0.0123 | 0.0018 | 2.80E-11 | 18.3253679 |
| 393 | rs7869771 | C | A | 0.2096 | 0.2647 | -0.014 | 0.0019 | 4.90E-13 | 21.13535776 |
| 394 | rs7871866 | C | G | 0.1928 | 0.1531 | 0.0187 | 0.0024 | 2.30E-14 | 15.74369913 |
| 395 | rs7903146 | T | C | 0.1993 | 0.2912 | -0.0181 | 0.0018 | 1.30E-23 | 41.74254344 |
| 396 | rs7925214 | T | C | 0.5268 | 0.5133 | 0.0147 | 0.0018 | 4.40E-17 | 33.32516759 |
| 397 | rs7970953 | A | G | 0.3037 | 0.29 | 0.0135 | 0.0018 | 9.80E-14 | 23.16437181 |
| 398 | rs7983065 | T | C | 0.4485 | 0.4503 | -0.0148 | 0.0017 | 8.90E-18 | 37.52369491 |
| 399 | rs7998796 | G | A | 0.3715 | 0.3373 | 0.0105 | 0.0018 | 1.10E-08 | 15.21266208 |
| 400 | rs8027205 | G | C | 0.4495 | 0.3967 | -0.0108 | 0.0018 | 1.40E-09 | 17.23207869 |
| 401 | rs8036040 | A | C | 0.4868 | 0.4932 | 0.0109 | 0.0017 | 2.70E-10 | 20.55211312 |
| 402 | rs8047395 | A | G | 0.5088 | 0.5061 | 0.0642 | 0.0017 | 1.00E-200 | 713.6235459 |
| 403 | rs806600 | G | A | 0.4722 | 0.475 | -0.0095 | 0.0017 | 3.30E-08 | 15.57545703 |
| 404 | rs8071182 | A | G | 0.1541 | 0.1735 | 0.0133 | 0.0022 | 2.10E-09 | 10.48177971 |
| 405 | rs8090983 | G | A | 0.3457 | 0.3314 | 0.0118 | 0.0018 | 2.00E-10 | 19.04490115 |
| 406 | rs8097672 | T | A | 0.1731 | 0.1528 | 0.02 | 0.0025 | 8.40E-16 | 16.57022838 |
| 407 | rs8097783 | A | G | 0.04104 | 0.07554 | -0.0389 | 0.0031 | 7.20E-36 | 21.99286618 |
| 408 | rs8123881 | G | A | 0.2353 | 0.1299 | 0.0196 | 0.0024 | 4.40E-16 | 15.07665983 |
| 409 | rs8181823 | C | A | 0.7295 | 0.7614 | 0.0127 | 0.002 | 4.10E-10 | 14.65104847 |
| 410 | rs818524 | C | T | 0.7314 | 0.6939 | 0.0106 | 0.0019 | 3.40E-08 | 13.22214802 |
| 411 | rs8192675 | C | T | 0.2642 | 0.2888 | 0.0152 | 0.0018 | 1.40E-17 | 29.29381931 |
| 412 | rs825688 | T | C | 0.4326 | 0.456 | -0.0095 | 0.0017 | 4.70E-08 | 15.49357528 |
| 413 | rs845084 | A | G | 0.26 | 0.2678 | 0.014 | 0.002 | 1.30E-12 | 19.21663236 |
| 414 | rs852056 | C | T | 0.7654 | 0.7584 | -0.0128 | 0.002 | 1.80E-10 | 15.01043799 |
| 415 | rs865809 | G | A | 0.7178 | 0.7678 | -0.0127 | 0.002 | 5.40E-10 | 14.37790366 |
| 416 | rs872281 | T | C | 0.2399 | 0.1728 | -0.0151 | 0.0023 | 4.70E-11 | 12.32222185 |
| 417 | rs876605 | G | A | 0.7409 | 0.7352 | -0.0108 | 0.002 | 3.40E-08 | 11.35394693 |
| 418 | rs879620 | T | C | 0.5787 | 0.6179 | 0.0231 | 0.0018 | 5.30E-38 | 77.77714245 |
| 419 | rs889398 | T | C | 0.4268 | 0.4247 | -0.0196 | 0.0016 | 1.30E-32 | 73.33613838 |
| 420 | rs895330 | G | C | 0.1643 | 0.1924 | -0.0201 | 0.0023 | 5.50E-19 | 23.73458327 |
| 421 | rs901630 | T | C | 0.3395 | 0.3973 | -0.0146 | 0.0017 | 1.90E-18 | 35.32448463 |
| 422 | rs902695 | A | G | 0.4164 | 0.4798 | -0.0103 | 0.0017 | 2.20E-09 | 18.32515434 |
| 423 | rs9294260 | A | G | 0.5074 | 0.4731 | 0.0147 | 0.0016 | 1.80E-19 | 42.085071 |
| 424 | rs9300422 | G | A | 0.7126 | 0.6903 | -0.0103 | 0.0018 | 4.00E-09 | 14.00054911 |
| 425 | rs930295 | C | A | 0.8711 | 0.8417 | -0.0211 | 0.0023 | 1.00E-19 | 22.42798846 |
| 426 | rs9304665 | A | T | 0.7199 | 0.7633 | 0.0229 | 0.002 | 2.90E-29 | 47.37651062 |
| 427 | rs934224 | T | C | 0.7006 | 0.7399 | 0.0107 | 0.002 | 4.70E-08 | 11.01682862 |
| 428 | rs9362662 | G | A | 0.5757 | 0.5201 | -0.0112 | 0.0017 | 1.20E-10 | 21.66797324 |
| 429 | rs9367368 | C | T | 0.3837 | 0.3033 | -0.0121 | 0.0018 | 1.00E-11 | 19.09780195 |
| 430 | rs9379827 | A | C | 0.2326 | 0.2409 | -0.0132 | 0.0019 | 6.90E-12 | 17.65285678 |
| 431 | rs9408882 | A | G | 0.478 | 0.4594 | -0.0093 | 0.0016 | 1.30E-08 | 16.78151022 |
| 432 | rs946824 | C | T | 0.8539 | 0.859 | -0.0206 | 0.0026 | 1.10E-15 | 15.20681741 |
| 433 | rs947612 | A | G | 0.7289 | 0.7516 | -0.0116 | 0.002 | 5.60E-09 | 12.5611953 |
| 434 | rs9478671 | G | A | 0.1749 | 0.2087 | 0.012 | 0.0021 | 1.70E-08 | 10.78507223 |
| 435 | rs9522285 | A | G | 0.4754 | 0.4143 | 0.0127 | 0.0017 | 2.50E-13 | 27.08604064 |
| 436 | rs9538162 | C | T | 0.4012 | 0.4138 | -0.0156 | 0.0018 | 4.80E-19 | 36.4411563 |
| 437 | rs9547153 | G | A | 0.4017 | 0.3839 | 0.0098 | 0.0017 | 8.70E-09 | 15.72031738 |
| 438 | rs9571687 | A | C | 0.2256 | 0.329 | -0.0129 | 0.0018 | 2.80E-12 | 22.67753366 |
| 439 | rs9615905 | T | C | 0.4349 | 0.45 | 0.011 | 0.0017 | 2.70E-10 | 20.72547571 |
| 440 | rs962273 | C | T | 0.7234 | 0.7057 | 0.0137 | 0.0019 | 2.60E-13 | 21.59666022 |
| 441 | rs9650755 | G | A | 0.3112 | 0.2664 | 0.0154 | 0.002 | 2.80E-15 | 23.17492268 |
| 442 | rs977747 | G | T | 0.6348 | 0.5949 | -0.0169 | 0.0017 | 1.30E-24 | 47.63616045 |
| 443 | rs9783858 | T | C | 0.5247 | 0.5191 | 0.0091 | 0.0017 | 3.30E-08 | 14.30631149 |
| 444 | rs9806742 | A | G | 0.9454 | 0.8826 | 0.0208 | 0.0026 | 1.40E-15 | 13.26322243 |
| 445 | rs9816226 | T | A | 0.8382 | 0.8199 | 0.0323 | 0.0021 | 1.60E-52 | 69.8729244 |
| 446 | rs9845966 | G | T | 0.4885 | 0.5479 | -0.0105 | 0.0017 | 2.50E-10 | 18.89974703 |
| 447 | rs987237 | G | A | 0.2114 | 0.1803 | 0.0409 | 0.0021 | 9.30E-84 | 112.1369655 |
| 448 | rs9926784 | C | T | 0.1712 | 0.1822 | -0.0258 | 0.0021 | 9.90E-35 | 44.98315758 |
| 449 | rs9927848 | A | C | 0.8022 | 0.7326 | -0.0122 | 0.002 | 6.40E-10 | 14.57893951 |
| 450 | rs9951619 | G | T | 0.6731 | 0.7643 | 0.0156 | 0.002 | 1.40E-15 | 21.92067081 |
| 451 | rs998732 | G | A | 0.1004 | 0.1578 | -0.0171 | 0.0022 | 2.00E-14 | 16.05856677 |
| 452 | rs9989141 | T | C | 0.6261 | 0.6387 | 0.0162 | 0.0017 | 3.60E-21 | 41.91312921 |
| 453 | rs999889 | A | G | 0.2411 | 0.2818 | -0.0108 | 0.0019 | 1.40E-08 | 13.0786724 |
| **Exposures** | **SNP** | **Effect**  **allele** | **Other**  **allele** | **Eaf**  **Outcomes** | **Eaf**  **Exposures** | **Beta**  **Exposures** | **Se**  **Exposures** | **P**  **Exposures** | **F statistic** |
| **Triglycerides** |  |  |  |  |  |  |  |  |  |
| 1 | rs1009360 | C | T | 0.4091 | 0.41859 | -0.0184832 | 0.00200785 | 3.40E-20 | 41.25074424 |
| 2 | rs10152471 | A | G | 0.421 | 0.388656 | -0.0135209 | 0.00204778 | 4.00E-11 | 20.7178205 |
| 3 | rs10210970 | T | C | 0.1842 | 0.131448 | 0.0232557 | 0.00292788 | 2.00E-15 | 14.40602972 |
| 4 | rs10242866 | T | C | 0.2976 | 0.398698 | 0.015793 | 0.00202877 | 7.00E-15 | 29.05741578 |
| 5 | rs1037117 | A | G | 0.2854 | 0.254798 | 0.0172213 | 0.00229153 | 5.70E-14 | 21.4486491 |
| 6 | rs10405944 | C | T | 0.4134 | 0.483077 | -0.013209 | 0.00201995 | 6.20E-11 | 21.35747545 |
| 7 | rs1043897 | T | G | 0.3704 | 0.415585 | -0.014664 | 0.00201517 | 3.40E-13 | 25.72267319 |
| 8 | rs1045241 | T | C | 0.3132 | 0.270943 | -0.0206988 | 0.00224699 | 3.20E-20 | 33.52652312 |
| 9 | rs10642257 | A | AAGGCC | 0.7826 | 0.839273 | 0.0370518 | 0.00271209 | 1.70E-42 | 50.35935201 |
| 10 | rs10750766 | A | C | 0.7376 | 0.709959 | 0.0193855 | 0.00218742 | 7.80E-19 | 32.34761007 |
| 11 | rs10773000 | T | G | 0.353 | 0.332276 | -0.0149294 | 0.0021148 | 1.70E-12 | 22.11525079 |
| 12 | rs10773049 | C | T | 0.4249 | 0.39535 | -0.0290398 | 0.00203691 | 4.10E-46 | 97.19705212 |
| 13 | rs10775406 | G | A | 0.7359 | 0.759694 | 0.0206571 | 0.00232578 | 6.60E-19 | 28.80455316 |
| 14 | rs1077835 | G | A | 0.2574 | 0.220459 | 0.0473668 | 0.00240452 | 2.20E-86 | 133.4189417 |
| 15 | rs10797119 | C | T | 0.5091 | 0.536622 | 0.0157113 | 0.00200465 | 4.60E-15 | 30.54985265 |
| 16 | rs10822163 | G | C | 0.4718 | 0.473369 | -0.0321674 | 0.00199059 | 9.70E-59 | 130.2359179 |
| 17 | rs10883026 | T | C | 0.5852 | 0.521569 | -0.0144604 | 0.00200472 | 5.50E-13 | 25.96796082 |
| 18 | rs10899490 | T | C | 0.1978 | 0.161369 | -0.0170082 | 0.00269347 | 2.70E-10 | 10.79250098 |
| 19 | rs11000468 | T | C | 0.4188 | 0.255269 | -0.0148544 | 0.00231748 | 1.50E-10 | 15.62137139 |
| 20 | rs11030107 | G | A | 0.2505 | 0.260757 | 0.0160418 | 0.00225693 | 1.20E-12 | 19.47782321 |
| 21 | rs11078597 | C | T | 0.1891 | 0.186613 | 0.0191708 | 0.0025513 | 5.70E-14 | 17.14120869 |
| 22 | rs11100083 | C | T | 0.1876 | 0.226214 | -0.0160248 | 0.00237585 | 1.50E-11 | 15.92690015 |
| 23 | rs11118310 | T | A | 0.596 | 0.593476 | 0.0193178 | 0.00202011 | 1.10E-21 | 44.12920725 |
| 24 | rs11122450 | G | T | 0.5638 | 0.611733 | -0.0481745 | 0.0020373 | 1.30E-123 | 265.7703442 |
| 25 | rs11185542 | C | G | 0.6941 | 0.727914 | -0.0126395 | 0.00223848 | 1.60E-08 | 12.62932335 |
| 26 | rs11187019 | G | A | 0.555 | 0.550621 | -0.0116437 | 0.00200546 | 6.40E-09 | 16.6826103 |
| 27 | rs11206374 | A | G | 0.2124 | 0.224815 | 0.0250232 | 0.00237577 | 6.10E-26 | 38.66995409 |
| 28 | rs11240358 | A | G | 0.3735 | 0.394026 | 0.0135651 | 0.00202953 | 2.30E-11 | 21.33452384 |
| 29 | rs11274835 | CGAGTGTGGGAATCT | C | 0.1184 | 0.174385 | -0.0254256 | 0.0027693 | 4.30E-20 | 24.27398277 |
| 30 | rs1133400 | G | A | 0.1731 | 0.219923 | 0.0137462 | 0.00239759 | 9.80E-09 | 11.27879588 |
| 31 | rs11429307 | G | GT | 0.8652 | 0.80902 | -0.0465382 | 0.00253471 | 2.70E-75 | 104.1934393 |
| 32 | rs11637681 | G | A | 0.2063 | 0.276291 | 0.0125866 | 0.00224251 | 2.00E-08 | 12.59851127 |
| 33 | rs11664106 | T | A | 0.3314 | 0.374102 | -0.0125831 | 0.00210816 | 2.40E-09 | 16.68423153 |
| 34 | rs116843064 | A | G | 0.02767 | 0.019327 | -0.226505 | 0.00721009 | 1.00E-200 | 37.41344918 |
| 35 | rs11722924 | C | G | 0.5077 | 0.53531 | 0.0129095 | 0.00199093 | 8.90E-11 | 20.91819323 |
| 36 | rs11746801 | A | G | 0.5908 | 0.637069 | -0.0123657 | 0.00207937 | 2.70E-09 | 16.35417095 |
| 37 | rs12185242 | C | A | 0.4829 | 0.454836 | 0.0175265 | 0.00199898 | 1.80E-18 | 38.1259845 |
| 38 | rs12424054 | A | G | 0.1768 | 0.232437 | 0.0191212 | 0.00235346 | 4.50E-16 | 23.55521976 |
| 39 | rs12440800 | T | A | 0.2103 | 0.255149 | 0.0161 | 0.00229294 | 2.20E-12 | 18.74028223 |
| 40 | rs12446515 | T | C | 0.2798 | 0.322889 | -0.0334207 | 0.00213589 | 3.50E-55 | 107.0828454 |
| 41 | rs12475332 | G | T | 0.2139 | 0.260988 | -0.0140007 | 0.00225386 | 5.20E-10 | 14.88539426 |
| 42 | rs12504746 | T | C | 0.1512 | 0.192857 | -0.0152674 | 0.00252148 | 1.40E-09 | 11.41416826 |
| 43 | rs12530679 | G | A | 0.4901 | 0.48466 | -0.0121299 | 0.00201682 | 1.80E-09 | 18.06995262 |
| 44 | rs12669911 | C | A | 0.6979 | 0.614026 | -0.0118536 | 0.00205405 | 7.90E-09 | 15.78581092 |
| 45 | rs12880341 | C | T | 0.1344 | 0.159023 | 0.0209696 | 0.00273647 | 1.80E-14 | 15.7067548 |
| 46 | rs12902047 | C | A | 0.3408 | 0.313907 | -0.0129253 | 0.00214533 | 1.70E-09 | 15.63581288 |
| 47 | rs1292065 | G | C | 0.693 | 0.709244 | -0.0139237 | 0.00218669 | 1.90E-10 | 16.72259711 |
| 48 | rs12926107 | G | A | 0.3062 | 0.454903 | 0.0126364 | 0.0020014 | 2.70E-10 | 19.77056254 |
| 49 | rs12928099 | A | C | 0.2823 | 0.296442 | -0.0282116 | 0.00218133 | 2.90E-38 | 69.78290924 |
| 50 | rs13108218 | G | A | 0.6773 | 0.614859 | -0.0305169 | 0.00205811 | 9.70E-50 | 104.1524598 |
| 51 | rs13118477 | A | G | 0.4572 | 0.392654 | 0.0149897 | 0.00203847 | 1.90E-13 | 25.79151533 |
| 52 | rs1316753 | C | G | 0.4239 | 0.394062 | -0.0145732 | 0.00202974 | 7.00E-13 | 24.61920617 |
| 53 | rs13264304 | G | C | 0.2255 | 0.151023 | 0.0189102 | 0.00276507 | 8.00E-12 | 11.99385623 |
| 54 | rs13269725 | G | A | 0.03677 | 0.078416 | 0.0349878 | 0.0036804 | 2.00E-21 | 13.06242251 |
| 55 | rs13354321 | C | T | 0.4829 | 0.409787 | -0.0154305 | 0.00201429 | 1.90E-14 | 28.3882704 |
| 56 | rs13389219 | T | C | 0.3486 | 0.392551 | -0.0376242 | 0.00202704 | 6.60E-77 | 164.3636117 |
| 57 | rs1340819 | C | A | 0.3245 | 0.345011 | -0.0121655 | 0.00209472 | 6.30E-09 | 15.24466742 |
| 58 | rs134551 | T | C | 0.276 | 0.335438 | -0.0116536 | 0.00211111 | 3.40E-08 | 13.58587636 |
| 59 | rs1347188 | G | A | 0.2701 | 0.245797 | 0.0139021 | 0.00231385 | 1.90E-09 | 13.38432437 |
| 60 | rs139974673 | C | T | 0.005245 | 0.026131 | 0.143073 | 0.00623867 | 2.20E-116 | 26.76962567 |
| 61 | rs140107293 | G | A | 0.1415 | 0.154707 | -0.0227003 | 0.00275296 | 1.60E-16 | 17.78386557 |
| 62 | rs1420384 | T | G | 0.6895 | 0.66699 | -0.01279 | 0.00210534 | 1.20E-09 | 16.39522475 |
| 63 | rs145947882 | C | A | 0.0301 | 0.025952 | 0.137333 | 0.0063668 | 3.40E-103 | 23.52392476 |
| 64 | rs1473886 | T | G | 0.5804 | 0.477624 | -0.0181218 | 0.00198519 | 6.90E-20 | 41.58501294 |
| 65 | rs1544980 | C | T | 0.1875 | 0.198644 | 0.0238704 | 0.00248513 | 7.60E-22 | 29.37508057 |
| 66 | rs1567353 | G | C | 0.3352 | 0.307644 | 0.0148202 | 0.00216358 | 7.40E-12 | 19.98885107 |
| 67 | rs17184382 | C | A | 0.3223 | 0.425367 | -0.0219251 | 0.00201039 | 1.10E-27 | 58.15166853 |
| 68 | rs17326656 | T | G | 0.1631 | 0.238472 | 0.0174394 | 0.00233365 | 7.80E-14 | 20.2844346 |
| 69 | rs174566 | G | A | 0.4139 | 0.34974 | 0.0485019 | 0.00207982 | 2.80E-120 | 247.4970533 |
| 70 | rs17585887 | C | T | 0.5177 | 0.591074 | -0.0285849 | 0.00201656 | 1.30E-45 | 97.15414356 |
| 71 | rs1760801 | A | G | 0.3707 | 0.295768 | -0.0202981 | 0.00218442 | 1.50E-20 | 35.97235545 |
| 72 | rs1799831 | T | C | 0.1922 | 0.156029 | 0.024506 | 0.00273953 | 3.70E-19 | 21.07532914 |
| 73 | rs1938566 | T | C | 0.7691 | 0.834537 | -0.0212127 | 0.00267054 | 2.00E-15 | 17.42550615 |
| 74 | rs2043085 | C | T | 0.5792 | 0.612184 | -0.0308028 | 0.00204157 | 1.90E-51 | 108.1170087 |
| 75 | rs2068888 | A | G | 0.47 | 0.450732 | -0.0318216 | 0.00199589 | 3.20E-57 | 125.8999334 |
| 76 | rs2070341 | T | C | 0.6229 | 0.60309 | 0.0113012 | 0.00203413 | 2.80E-08 | 14.77775488 |
| 77 | rs2071887 | A | T | 0.3671 | 0.344804 | 0.016312 | 0.00209671 | 7.30E-15 | 27.34866821 |
| 78 | rs2081194 | C | G | 0.6584 | 0.600934 | -0.0214115 | 0.00207674 | 6.30E-25 | 50.98937289 |
| 79 | rs2081687 | C | T | 0.6151 | 0.663201 | -0.0261379 | 0.00209126 | 7.60E-36 | 69.79720679 |
| 80 | rs2092203 | T | C | 0.5341 | 0.481154 | 0.0136756 | 0.00199323 | 6.80E-12 | 23.50453666 |
| 81 | rs2131311 | G | A | 0.709 | 0.714636 | -0.0124181 | 0.00222753 | 2.50E-08 | 12.67616154 |
| 82 | rs2131919 | G | A | 0.1812 | 0.164266 | 0.0173511 | 0.00268396 | 1.00E-10 | 11.47511446 |
| 83 | rs213494 | T | C | 0.719 | 0.648345 | 0.0156106 | 0.00207578 | 5.50E-14 | 25.7900943 |
| 84 | rs2137557 | C | T | 0.6673 | 0.645612 | 0.0117624 | 0.00208003 | 1.60E-08 | 14.63341634 |
| 85 | rs2237029 | A | G | 0.6079 | 0.601353 | -0.0139399 | 0.00204019 | 8.30E-12 | 22.38443508 |
| 86 | rs2240466 | A | G | 0.1254 | 0.122916 | -0.122769 | 0.00301848 | 1.00E-200 | 356.9684654 |
| 87 | rs2240533 | C | T | 0.2967 | 0.309898 | -0.0129897 | 0.00215234 | 1.60E-09 | 15.57944261 |
| 88 | rs2244278 | A | C | 0.09219 | 0.120553 | -0.0269912 | 0.00306133 | 1.20E-18 | 16.48375095 |
| 89 | rs2267373 | T | C | 0.5973 | 0.580693 | 0.0215638 | 0.00202145 | 1.40E-26 | 55.42247132 |
| 90 | rs2302263 | T | C | 0.128 | 0.088863 | 0.0436112 | 0.00349051 | 8.00E-36 | 25.27993044 |
| 91 | rs2382825 | T | C | 0.7312 | 0.622954 | -0.0134774 | 0.00204919 | 4.80E-11 | 20.32104952 |
| 92 | rs2487294 | T | G | 0.6823 | 0.723477 | 0.0183191 | 0.00222084 | 1.60E-16 | 27.22604451 |
| 93 | rs2519093 | T | C | 0.2006 | 0.184548 | -0.021158 | 0.00256189 | 1.50E-16 | 20.52977816 |
| 94 | rs2604568 | A | T | 0.7377 | 0.663895 | 0.0116923 | 0.00210543 | 2.80E-08 | 13.76366955 |
| 95 | rs2699805 | A | G | 0.405 | 0.399672 | -0.0201313 | 0.00203748 | 5.10E-23 | 46.85149115 |
| 96 | rs275184 | G | T | 0.1977 | 0.162051 | -0.0173487 | 0.00275263 | 2.90E-10 | 10.78812944 |
| 97 | rs2773469 | G | A | 0.7476 | 0.733286 | -0.0188717 | 0.00225426 | 5.70E-17 | 27.41500106 |
| 98 | rs278981 | C | T | 0.7608 | 0.758155 | 0.0125871 | 0.00229058 | 3.90E-08 | 11.07374411 |
| 99 | rs28383314 | C | T | 0.6895 | 0.624109 | 0.0379005 | 0.00204671 | 1.50E-76 | 160.9481992 |
| 100 | rs28439112 | A | T | 0.1962 | 0.255528 | 0.0126014 | 0.00226754 | 2.70E-08 | 11.75044036 |
| 101 | rs28577186 | A | G | 0.6422 | 0.664571 | -0.0163428 | 0.00211672 | 1.20E-14 | 26.578 |
| 102 | rs28752924 | G | T | 0.444 | 0.243148 | 0.0243875 | 0.00276364 | 1.10E-18 | 28.6622012 |
| 103 | rs28752924 | G | T | 0.1016 | 0.243148 | 0.0243875 | 0.00276364 | 1.10E-18 | 28.6622012 |
| 104 | rs2925979 | C | T | 0.6776 | 0.699976 | -0.0322092 | 0.00217095 | 8.50E-50 | 92.47374836 |
| 105 | rs2937124 | T | C | 0.3952 | 0.362769 | -0.0182561 | 0.00212336 | 8.10E-18 | 34.17885379 |
| 106 | rs2943645 | T | C | 0.638 | 0.646594 | 0.0402918 | 0.00207151 | 2.90E-84 | 172.9669725 |
| 107 | rs2983896 | A | G | 0.1887 | 0.21474 | 0.0137912 | 0.00242177 | 1.20E-08 | 10.93713428 |
| 108 | rs308 | G | T | 0.02987 | 0.020597 | -0.159413 | 0.00698442 | 2.60E-115 | 21.01846026 |
| 109 | rs3103310 | G | A | 0.1697 | 0.241785 | 0.0202549 | 0.00236652 | 1.10E-17 | 26.86062115 |
| 110 | rs320369 | G | A | 0.6274 | 0.683292 | -0.0125327 | 0.00214567 | 5.20E-09 | 14.76628909 |
| 111 | rs325485 | G | A | 0.6489 | 0.602889 | -0.0117435 | 0.00204042 | 8.60E-09 | 15.86166903 |
| 112 | rs326222 | C | T | 0.5973 | 0.697957 | 0.0252203 | 0.00215698 | 1.40E-31 | 57.64882971 |
| 113 | rs343 | A | C | 0.06338 | 0.083129 | -0.141493 | 0.00361052 | 1.00E-200 | 234.2339392 |
| 114 | rs34682685 | A | G | 0.05763 | 0.104198 | 0.0337428 | 0.00326987 | 5.80E-25 | 19.88017833 |
| 115 | rs35140741 | CAAAAAAAA | C | 0.66 | 0.634615 | -0.0155759 | 0.00206772 | 5.00E-14 | 26.31708799 |
| 116 | rs36043408 | A | G | 0.48 | 0.502153 | -0.0128072 | 0.00198554 | 1.10E-10 | 20.80326829 |
| 117 | rs3731696 | G | A | 0.1223 | 0.121188 | 0.0218939 | 0.00303176 | 5.10E-13 | 11.10839168 |
| 118 | rs3758413 | C | T | 0.4164 | 0.417724 | 0.0111232 | 0.00201767 | 3.50E-08 | 14.78495893 |
| 119 | rs3775228 | T | C | 0.3967 | 0.399717 | 0.0338197 | 0.00203602 | 5.80E-62 | 132.4471206 |
| 120 | rs3808477 | T | C | 0.3085 | 0.279294 | -0.0133854 | 0.00220342 | 1.20E-09 | 14.85697983 |
| 121 | rs3814883 | T | C | 0.4115 | 0.482498 | 0.0148926 | 0.00199657 | 8.70E-14 | 27.7865678 |
| 122 | rs3820897 | C | T | 0.647 | 0.819861 | 0.0197269 | 0.00260304 | 3.50E-14 | 16.96479867 |
| 123 | rs3860846 | T | C | 0.2504 | 0.27531 | 0.0297027 | 0.00224011 | 4.00E-40 | 70.16564798 |
| 124 | rs394872 | T | C | 0.5655 | 0.536237 | 0.0111807 | 0.00200285 | 2.40E-08 | 15.50020004 |
| 125 | rs3974807 | T | C | 0.157 | 0.189267 | 0.0159776 | 0.00253125 | 2.80E-10 | 12.22772954 |
| 126 | rs4128205 | C | A | 0.5786 | 0.509173 | 0.0115011 | 0.00199472 | 8.10E-09 | 16.61701744 |
| 127 | rs4134963 | T | C | 0.1935 | 0.189878 | -0.0189544 | 0.00254235 | 9.00E-14 | 17.10091492 |
| 128 | rs41785 | A | C | 0.3969 | 0.417487 | -0.0150445 | 0.00201492 | 8.20E-14 | 27.11709795 |
| 129 | rs4253750 | C | T | 0.1668 | 0.214455 | 0.0177576 | 0.00243701 | 3.20E-13 | 17.8898543 |
| 130 | rs4665972 | C | T | 0.6231 | 0.604587 | -0.10025 | 0.00203577 | 1.00E-200 | 1162.498488 |
| 131 | rs4675812 | A | G | 0.6172 | 0.587882 | -0.0142305 | 0.00201176 | 1.50E-12 | 24.24664372 |
| 132 | rs4731701 | T | C | 0.4643 | 0.493088 | -0.0325803 | 0.00198921 | 2.70E-60 | 134.1423779 |
| 133 | rs4760254 | C | G | 0.2316 | 0.23908 | -0.0281414 | 0.00232488 | 1.00E-33 | 53.3154511 |
| 134 | rs4761234 | C | T | 0.4124 | 0.484365 | -0.0140236 | 0.00199376 | 2.00E-12 | 24.71387121 |
| 135 | rs4765148 | T | G | 0.2899 | 0.312686 | -0.0251537 | 0.0021456 | 9.70E-32 | 59.08211914 |
| 136 | rs480823 | C | T | 0.1601 | 0.078996 | 0.15572 | 0.00371974 | 1.00E-200 | 255.15816 |
| 137 | rs483082 | T | G | 0.2364 | 0.23535 | 0.0862119 | 0.00234474 | 1.00E-200 | 487.1126365 |
| 138 | rs483808 | T | C | 0.6232 | 0.701973 | -0.0136636 | 0.00218517 | 4.00E-10 | 16.35987481 |
| 139 | rs4841580 | C | T | 0.4847 | 0.434941 | -0.0245383 | 0.00200093 | 1.40E-34 | 73.93500829 |
| 140 | rs4969179 | G | T | 0.7052 | 0.60445 | -0.0177529 | 0.00203649 | 2.80E-18 | 36.34123087 |
| 141 | rs4976033 | G | A | 0.4032 | 0.402122 | 0.0177909 | 0.00205178 | 4.30E-18 | 36.15499357 |
| 142 | rs499293 | A | G | 0.6208 | 0.65807 | -0.0117912 | 0.00208902 | 1.70E-08 | 14.33779149 |
| 143 | rs5112 | G | C | 0.5831 | 0.533428 | 0.0684501 | 0.00213412 | 1.00E-200 | 512.66961 |
| 144 | rs551243 | C | G | 0.4424 | 0.469258 | 0.0143952 | 0.00199178 | 4.90E-13 | 26.01965072 |
| 145 | rs55646464 | T | G | 0.4135 | 0.300087 | 0.0121913 | 0.00217016 | 1.90E-08 | 13.25710973 |
| 146 | rs55966194 | G | C | 0.2151 | 0.281997 | -0.0178509 | 0.00221245 | 7.10E-16 | 26.3631948 |
| 147 | rs56397607 | G | A | 0.1636 | 0.182538 | 0.0174851 | 0.00257608 | 1.10E-11 | 13.74928063 |
| 148 | rs56902258 | A | T | 0.1963 | 0.196198 | -0.0151434 | 0.0025129 | 1.70E-09 | 11.45458449 |
| 149 | rs5755799 | G | C | 0.4841 | 0.453747 | 0.0118859 | 0.00200343 | 3.00E-09 | 17.44892232 |
| 150 | rs57996145 | G | GT | 0.8642 | 0.83326 | -0.0292341 | 0.00268763 | 1.50E-27 | 32.87919915 |
| 151 | rs581080 | C | G | 0.8548 | 0.819008 | 0.0175432 | 0.00258442 | 1.10E-11 | 13.66093561 |
| 152 | rs58542926 | T | C | 0.06432 | 0.074608 | -0.103184 | 0.00378074 | 5.30E-164 | 102.8754045 |
| 153 | rs6028716 | A | G | 0.1827 | 0.258428 | -0.0128217 | 0.00228302 | 2.00E-08 | 12.08939622 |
| 154 | rs6073958 | C | T | 0.176 | 0.198674 | 0.0556952 | 0.00249344 | 1.60E-110 | 158.9177676 |
| 155 | rs60856912 | T | G | 0.218 | 0.162906 | 0.0248329 | 0.00270829 | 4.80E-20 | 22.93118318 |
| 156 | rs61830291 | C | A | 0.1154 | 0.095791 | 0.0287484 | 0.00337913 | 1.80E-17 | 12.53866975 |
| 157 | rs61905078 | C | A | 0.1055 | 0.073841 | 0.199898 | 0.00379494 | 1.00E-200 | 379.832482 |
| 158 | rs62102718 | T | A | 0.2879 | 0.286196 | 0.0202348 | 0.00220142 | 3.90E-20 | 34.5220498 |
| 159 | rs62112763 | G | C | 0.4337 | 0.438929 | 0.01935 | 0.00200786 | 5.60E-22 | 45.74885784 |
| 160 | rs62117489 | A | C | 0.03475 | 0.056432 | -0.0427743 | 0.00431389 | 3.60E-23 | 10.47043371 |
| 161 | rs62128802 | T | C | 0.1705 | 0.183216 | -0.0158746 | 0.00257782 | 7.40E-10 | 11.35037543 |
| 162 | rs62135012 | A | G | 0.43 | 0.357929 | -0.0117482 | 0.00207507 | 1.50E-08 | 14.73328656 |
| 163 | rs62271373 | A | T | 0.04392 | 0.059967 | 0.0419639 | 0.00426588 | 7.80E-23 | 10.91009696 |
| 164 | rs62274099 | T | C | 0.411 | 0.424205 | 0.0120882 | 0.00202424 | 2.30E-09 | 17.42160341 |
| 165 | rs62397245 | G | C | 0.2464 | 0.22236 | 0.0149941 | 0.00239634 | 3.90E-10 | 13.54005874 |
| 166 | rs62427982 | T | C | 0.3758 | 0.32217 | -0.0133153 | 0.00213196 | 4.20E-10 | 17.03705376 |
| 167 | rs6432622 | G | A | 0.4512 | 0.490264 | -0.0108868 | 0.00198007 | 3.80E-08 | 15.1097618 |
| 168 | rs6517522 | C | T | 0.3644 | 0.498138 | -0.0129031 | 0.00199242 | 9.40E-11 | 20.97051053 |
| 169 | rs6532798 | T | C | 0.6974 | 0.697405 | 0.013792 | 0.00216353 | 1.80E-10 | 17.15222143 |
| 170 | rs6562773 | G | A | 0.51 | 0.547533 | -0.0120538 | 0.00201057 | 2.00E-09 | 17.80952513 |
| 171 | rs6572807 | G | A | 0.2698 | 0.267065 | 0.0124759 | 0.00224899 | 2.90E-08 | 12.04733669 |
| 172 | rs6752845 | C | G | 0.4569 | 0.431425 | -0.0137812 | 0.00200389 | 6.10E-12 | 23.2043955 |
| 173 | rs676210 | A | G | 0.2662 | 0.205293 | -0.0735169 | 0.00244968 | 7.10E-198 | 294.0724635 |
| 174 | rs6792725 | G | A | 0.5431 | 0.692404 | -0.0152543 | 0.00221886 | 6.20E-12 | 20.1332028 |
| 175 | rs67981690 | G | A | 0.1913 | 0.129887 | 0.0299225 | 0.00297454 | 8.30E-24 | 22.87431087 |
| 176 | rs6800707 | G | C | 0.9031 | 0.810543 | 0.0298848 | 0.00253832 | 5.30E-32 | 42.57597783 |
| 177 | rs6805924 | T | G | 0.4264 | 0.430548 | 0.0109684 | 0.00200571 | 4.50E-08 | 14.66464137 |
| 178 | rs684773 | C | A | 0.8476 | 0.766797 | 0.0291133 | 0.00234718 | 2.50E-35 | 55.02839299 |
| 179 | rs6882076 | C | T | 0.6672 | 0.634285 | 0.0331011 | 0.00206122 | 4.90E-58 | 119.6765979 |
| 180 | rs6916318 | T | A | 0.5022 | 0.530533 | 0.0267283 | 0.0019907 | 4.20E-41 | 89.81833113 |
| 181 | rs696825 | T | C | 0.3195 | 0.252926 | -0.0202838 | 0.00228763 | 7.50E-19 | 29.7126547 |
| 182 | rs698927 | C | A | 0.1811 | 0.183785 | -0.0183059 | 0.00256302 | 9.20E-13 | 15.30509089 |
| 183 | rs6999569 | G | A | 0.4583 | 0.470737 | -0.0860885 | 0.00198061 | 1.00E-200 | 943.4052331 |
| 184 | rs7000494 | C | G | 0.01897 | 0.030042 | 0.136538 | 0.00584159 | 8.00E-121 | 31.84095476 |
| 185 | rs7077812 | C | T | 0.1985 | 0.194911 | 0.0142284 | 0.00250611 | 1.40E-08 | 10.11649576 |
| 186 | rs7134375 | A | C | 0.4079 | 0.431174 | -0.0171435 | 0.00200504 | 1.20E-17 | 35.86313463 |
| 187 | rs7135509 | C | T | 0.298 | 0.293196 | -0.0121338 | 0.00221197 | 4.10E-08 | 12.4718979 |
| 188 | rs71368855 | T | C | 0.06849 | 0.115087 | 0.026073 | 0.00312826 | 7.80E-17 | 14.14963154 |
| 189 | rs7140110 | C | T | 0.2805 | 0.298338 | 0.0283172 | 0.00217811 | 1.20E-38 | 70.77424513 |
| 190 | rs71603401 | G | A | 0.07798 | 0.136804 | 0.0264991 | 0.00291753 | 1.10E-19 | 19.48439905 |
| 191 | rs7215055 | G | A | 0.07877 | 0.062892 | 0.0389233 | 0.00410062 | 2.30E-21 | 10.62047135 |
| 192 | rs7239575 | C | T | 0.4941 | 0.490494 | -0.0160767 | 0.00199133 | 6.80E-16 | 32.57995293 |
| 193 | rs72555385 | G | A | 0.02291 | 0.048975 | 0.0653776 | 0.0046178 | 1.70E-45 | 18.6724154 |
| 194 | rs72644085 | C | T | 0.1275 | 0.146036 | -0.0196036 | 0.00281241 | 3.20E-12 | 12.11863903 |
| 195 | rs7274718 | A | G | 0.592 | 0.598516 | 0.0159491 | 0.00202919 | 3.80E-15 | 29.69123118 |
| 196 | rs72801474 | A | G | 0.05753 | 0.091843 | -0.0307938 | 0.00344154 | 3.60E-19 | 13.3557752 |
| 197 | rs729761 | G | T | 0.6975 | 0.711859 | 0.0177868 | 0.00221435 | 9.50E-16 | 26.47014022 |
| 198 | rs73025562 | A | G | 0.3178 | 0.245879 | 0.0138633 | 0.00231292 | 2.00E-09 | 13.32341756 |
| 199 | rs7308584 | A | G | 0.2432 | 0.184436 | 0.0149992 | 0.00256911 | 5.30E-09 | 10.25445679 |
| 200 | rs7400002 | G | A | 0.2744 | 0.230609 | 0.0139701 | 0.00236663 | 3.60E-09 | 12.36522024 |
| 201 | rs742036 | A | G | 0.3401 | 0.375259 | -0.0143632 | 0.00205112 | 2.50E-12 | 22.99334051 |
| 202 | rs7424120 | T | C | 0.5972 | 0.601724 | -0.0123561 | 0.00203065 | 1.20E-09 | 17.74678348 |
| 203 | rs77009508 | G | A | 0.06095 | 0.073734 | 0.0450912 | 0.00379997 | 1.80E-32 | 19.23418261 |
| 204 | rs7704653 | G | A | 0.7544 | 0.723029 | 0.0157533 | 0.00224582 | 2.30E-12 | 19.70745964 |
| 205 | rs7714361 | C | A | 0.273 | 0.233667 | 0.0138858 | 0.00236622 | 4.40E-09 | 12.33350891 |
| 206 | rs7735249 | G | C | 0.1207 | 0.112767 | 0.0268635 | 0.00315997 | 1.90E-17 | 14.46176343 |
| 207 | rs7786339 | T | C | 0.1777 | 0.168936 | 0.016328 | 0.00265352 | 7.60E-10 | 10.63203569 |
| 208 | rs78058190 | A | G | 0.08195 | 0.05045 | 0.0816071 | 0.00510649 | 1.70E-57 | 24.47045531 |
| 209 | rs7847285 | C | T | 0.6227 | 0.589213 | -0.0116104 | 0.00202626 | 1.00E-08 | 15.89412819 |
| 210 | rs78484485 | A | G | 0.09161 | 0.054016 | -0.0758529 | 0.00439863 | 1.20E-66 | 30.3930078 |
| 211 | rs78588343 | A | G | 0.2198 | 0.176216 | -0.0155231 | 0.0026035 | 2.50E-09 | 10.32140045 |
| 212 | rs7861679 | T | C | 0.7177 | 0.696815 | 0.0122025 | 0.00216741 | 1.80E-08 | 13.39312444 |
| 213 | rs7947951 | G | A | 0.5377 | 0.689254 | 0.0193777 | 0.00214074 | 1.40E-19 | 35.10136792 |
| 214 | rs8102873 | T | C | 0.4766 | 0.584919 | 0.0123302 | 0.00201474 | 9.40E-10 | 18.18765652 |
| 215 | rs852388 | C | G | 0.1269 | 0.211278 | 0.0157124 | 0.00245601 | 1.60E-10 | 13.64094578 |
| 216 | rs867939 | A | G | 0.5221 | 0.57621 | -0.0136048 | 0.00202516 | 1.80E-11 | 22.04179044 |
| 217 | rs880315 | C | T | 0.4134 | 0.340211 | -0.011772 | 0.00210497 | 2.20E-08 | 14.04119145 |
| 218 | rs921971 | C | T | 0.195 | 0.265901 | 0.01565 | 0.00225602 | 4.00E-12 | 18.78728064 |
| 219 | rs9368503 | T | A | 0.6323 | 0.510113 | -0.0119616 | 0.00198801 | 1.80E-09 | 18.09462439 |
| 220 | rs9373056 | T | C | 0.2507 | 0.320263 | -0.0122839 | 0.00212321 | 7.20E-09 | 14.57393064 |
| 221 | rs9376511 | G | A | 0.2053 | 0.203288 | -0.0154993 | 0.00246511 | 3.20E-10 | 12.80575829 |
| 222 | rs9425589 | A | G | 0.5589 | 0.566781 | -0.0137618 | 0.00200182 | 6.20E-12 | 23.20994421 |
| 223 | rs9436661 | G | T | 0.2642 | 0.352919 | -0.0777322 | 0.00207793 | 1.00E-200 | 640.0761912 |
| 224 | rs9480889 | G | C | 0.8604 | 0.783137 | 0.0163067 | 0.0024101 | 1.30E-11 | 15.5499351 |
| 225 | rs954244 | G | C | 0.1924 | 0.254641 | 0.015323 | 0.00227424 | 1.60E-11 | 17.23272044 |
| 226 | rs9561643 | C | A | 0.3813 | 0.314726 | 0.0167149 | 0.00214481 | 6.50E-15 | 26.19875269 |
| 227 | rs9584870 | C | T | 0.3367 | 0.366179 | -0.0123562 | 0.00210377 | 4.30E-09 | 16.01316567 |
| 228 | rs970069 | T | C | 0.2002 | 0.212039 | 0.0162322 | 0.00243455 | 2.60E-11 | 14.85526824 |
| 229 | rs9831084 | C | T | 0.3745 | 0.461922 | -0.0118516 | 0.00199656 | 2.90E-09 | 17.51654289 |
| 230 | rs9859117 | C | G | 0.2691 | 0.202649 | 0.0147856 | 0.00247772 | 2.40E-09 | 11.50818461 |
| 231 | rs9889402 | A | G | 0.766 | 0.728192 | 0.0122827 | 0.00222926 | 3.60E-08 | 12.01750304 |
| 232 | rs9902027 | T | C | 0.7458 | 0.77437 | -0.0151609 | 0.00238645 | 2.10E-10 | 14.10368387 |
| 233 | rs998584 | A | C | 0.4822 | 0.482727 | 0.0401182 | 0.00198999 | 2.20E-90 | 203.0628326 |
| **Exposures** | **SNP** | **Effect**  **allele** | **Other**  **allele** | **Eaf**  **Outcomes** | **Eaf**  **Exposures** | **Beta**  **Exposures** | **Se**  **Exposures** | **P**  **Exposures** | **F statistic** |
| **LDL-C** |  |  |  |  |  |  |  |  |  |
| 1 | rs1010759 | A | G | 0.09187 | 0.140367 | -0.0231042 | 0.00300476 | 1.50E-14 | 14.26863667 |
| 2 | rs1016988 | C | T | 0.2171 | 0.190907 | -0.0174377 | 0.00265087 | 4.80E-11 | 13.3678955 |
| 3 | rs10231941 | C | T | 0.1981 | 0.177717 | 0.0198565 | 0.00272709 | 3.30E-13 | 15.49530193 |
| 4 | rs10448340 | G | T | 0.3038 | 0.319765 | -0.0150594 | 0.00223737 | 1.70E-11 | 19.70956641 |
| 5 | rs10832963 | G | T | 0.6174 | 0.744224 | 0.0173059 | 0.00239616 | 5.10E-13 | 19.85947738 |
| 6 | rs10910476 | T | C | 0.5106 | 0.555163 | 0.0123938 | 0.00210193 | 3.70E-09 | 17.17271015 |
| 7 | rs11014204 | T | C | 0.2159 | 0.280227 | 0.0137924 | 0.00233007 | 3.20E-09 | 14.13478601 |
| 8 | rs11065385 | G | A | 0.6481 | 0.690891 | -0.0244904 | 0.00225744 | 2.00E-27 | 50.27569627 |
| 9 | rs11099097 | T | C | 0.3451 | 0.291333 | -0.0181876 | 0.00230231 | 2.80E-15 | 25.76964922 |
| 10 | rs11226108 | C | G | 0.1823 | 0.191562 | -0.016026 | 0.00265376 | 1.60E-09 | 11.2959362 |
| 11 | rs11591147 | T | G | 0.03605 | 0.017468 | -0.348456 | 0.00793088 | 1.00E-200 | 66.27296109 |
| 12 | rs11621792 | T | C | 0.3686 | 0.452867 | 0.0191489 | 0.00211033 | 1.10E-19 | 40.80550439 |
| 13 | rs118039278 | A | G | 0.04597 | 0.078672 | 0.0835056 | 0.00388533 | 1.80E-102 | 66.97363675 |
| 14 | rs1183851 | C | T | 0.4592 | 0.395544 | 0.024377 | 0.00213943 | 4.50E-30 | 62.08877246 |
| 15 | rs12078100 | G | C | 0.7215 | 0.623087 | 0.0131127 | 0.00214876 | 1.00E-09 | 17.49216252 |
| 16 | rs12162782 | G | T | 0.4222 | 0.344365 | 0.0129592 | 0.00219352 | 3.50E-09 | 15.76151225 |
| 17 | rs12208357 | T | C | 0.05701 | 0.070102 | 0.0569843 | 0.00409617 | 5.40E-44 | 25.23324539 |
| 18 | rs12246352 | G | A | 0.06137 | 0.103895 | 0.0257335 | 0.00342791 | 6.00E-14 | 10.49375237 |
| 19 | rs12471768 | C | T | 0.7691 | 0.704119 | 0.0135999 | 0.00228112 | 2.50E-09 | 14.81088233 |
| 20 | rs1250258 | T | C | 0.7884 | 0.736649 | 0.0137745 | 0.00236771 | 6.00E-09 | 13.13201031 |
| 21 | rs1260326 | C | T | 0.6493 | 0.604443 | -0.0347299 | 0.00212546 | 5.10E-60 | 127.7084173 |
| 22 | rs12916 | C | T | 0.4542 | 0.400537 | 0.0621175 | 0.00212705 | 1.70E-187 | 409.9298218 |
| 23 | rs13020929 | A | G | 0.5105 | 0.4567 | 0.0145846 | 0.00209518 | 3.40E-12 | 24.0474379 |
| 24 | rs13076933 | G | T | 0.2708 | 0.259454 | -0.020994 | 0.00239027 | 1.60E-18 | 29.64600134 |
| 25 | rs13108218 | G | A | 0.6773 | 0.614867 | -0.0177871 | 0.0021598 | 1.80E-16 | 32.1243855 |
| 26 | rs13121616 | G | A | 0.7526 | 0.693553 | 0.0128507 | 0.00226135 | 1.30E-08 | 13.72759901 |
| 27 | rs1350559 | G | C | 0.4563 | 0.401347 | 0.0137824 | 0.00213685 | 1.10E-10 | 19.99146135 |
| 28 | rs143020224 | G | C | 0.1022 | 0.118603 | -0.168952 | 0.00320833 | 1.00E-200 | 580.5448929 |
| 29 | rs148150904 | TTAAAG | T | 0.2587 | 0.189702 | 0.0158136 | 0.00265248 | 2.50E-09 | 10.9272967 |
| 30 | rs150474434 | A | G | 0.0787 | 0.101353 | -0.0347623 | 0.00347063 | 1.30E-23 | 18.27562131 |
| 31 | rs1551891 | A | G | 0.07252 | 0.088107 | -0.173628 | 0.00366029 | 1.00E-200 | 361.8657173 |
| 32 | rs1556562 | T | G | 0.778 | 0.790033 | 0.0190249 | 0.00248903 | 2.10E-14 | 19.38330861 |
| 33 | rs17050272 | A | G | 0.5353 | 0.409275 | -0.0205597 | 0.00211549 | 2.50E-22 | 45.67569988 |
| 34 | rs174564 | G | A | 0.4151 | 0.348557 | -0.0318977 | 0.0021896 | 4.50E-48 | 96.39676138 |
| 35 | rs17569873 | T | C | 0.1377 | 0.201299 | 0.0171196 | 0.00260335 | 4.80E-11 | 13.90559848 |
| 36 | rs183130 | T | C | 0.2796 | 0.323808 | -0.0329541 | 0.00223034 | 2.10E-49 | 95.62174074 |
| 37 | rs1883711 | C | G | 0.06316 | 0.031412 | 0.10277 | 0.0060919 | 7.50E-64 | 17.31836749 |
| 38 | rs200046586 | C | CA | 0.98386 | 0.981093 | 0.309188 | 0.00782788 | 1.00E-200 | 57.88617162 |
| 39 | rs2043085 | C | T | 0.5792 | 0.612225 | -0.017018 | 0.00214177 | 1.90E-15 | 29.97918484 |
| 40 | rs2066714 | C | T | 0.09405 | 0.128775 | 0.0210151 | 0.00310964 | 1.40E-11 | 10.24808474 |
| 41 | rs2068888 | A | G | 0.47 | 0.450695 | -0.0192081 | 0.00209421 | 4.60E-20 | 41.65755141 |
| 42 | rs2073547 | G | A | 0.3265 | 0.184007 | 0.0355498 | 0.00267287 | 2.30E-40 | 53.12755422 |
| 43 | rs2160994 | C | T | 0.665 | 0.647494 | 0.0182581 | 0.00218417 | 6.30E-17 | 31.90068585 |
| 44 | rs2238162 | T | C | 0.5627 | 0.522955 | -0.0165008 | 0.00208847 | 2.80E-15 | 31.14841239 |
| 45 | rs2250802 | A | G | 0.6818 | 0.72434 | -0.0181939 | 0.00233224 | 6.10E-15 | 24.30375231 |
| 46 | rs2256814 | A | G | 0.186 | 0.197877 | 0.0152618 | 0.00262512 | 6.10E-09 | 10.72971244 |
| 47 | rs2287622 | G | A | 0.5098 | 0.602794 | -0.0211572 | 0.00212931 | 2.90E-23 | 47.28222982 |
| 48 | rs2391825 | A | G | 0.2178 | 0.279219 | -0.0129848 | 0.00233356 | 2.60E-08 | 12.46295977 |
| 49 | rs2519093 | T | C | 0.2006 | 0.18451 | 0.0557289 | 0.0026865 | 1.40E-95 | 129.5333214 |
| 50 | rs2611867 | G | A | 0.5337 | 0.513941 | -0.0265838 | 0.00209023 | 4.70E-37 | 80.82689953 |
| 51 | rs2618566 | T | G | 0.6771 | 0.659861 | -0.0249198 | 0.00219993 | 9.60E-30 | 57.60564993 |
| 52 | rs2642438 | G | A | 0.7157 | 0.702657 | 0.0253218 | 0.00227308 | 8.00E-29 | 51.86088805 |
| 53 | rs2737265 | G | A | 0.309 | 0.28046 | -0.0202743 | 0.002322 | 2.50E-18 | 30.77170327 |
| 54 | rs2738447 | C | A | 0.6063 | 0.592612 | 0.0422734 | 0.00211024 | 2.90E-89 | 193.851164 |
| 55 | rs2740488 | C | A | 0.1971 | 0.265466 | -0.0252452 | 0.00236503 | 1.30E-26 | 44.44029816 |
| 56 | rs2745353 | T | C | 0.5252 | 0.5181 | 0.0126963 | 0.00208336 | 1.10E-09 | 18.54567024 |
| 57 | rs2820226 | A | G | 0.5344 | 0.451903 | -0.012864 | 0.00209731 | 8.60E-10 | 18.63699348 |
| 58 | rs28406917 | T | C | 0.4491 | 0.427624 | 0.0119057 | 0.00211433 | 1.80E-08 | 15.52212365 |
| 59 | rs28601761 | G | C | 0.4126 | 0.418688 | -0.0620105 | 0.00213624 | 2.90E-185 | 410.546866 |
| 60 | rs28615248 | C | T | 0.2189 | 0.196095 | 0.0184347 | 0.00263568 | 2.70E-12 | 15.42415092 |
| 61 | rs28631087 | C | T | 0.1631 | 0.21274 | -0.0161901 | 0.0025459 | 2.00E-10 | 13.54643755 |
| 62 | rs28814720 | G | A | 0.4586 | 0.519766 | 0.0121747 | 0.00215073 | 1.50E-08 | 15.99739678 |
| 63 | rs3104412 | G | A | 0.4639 | 0.451531 | -0.0189061 | 0.00209423 | 1.80E-19 | 40.37036037 |
| 64 | rs3127580 | T | C | 0.124 | 0.155131 | 0.0357031 | 0.00287572 | 2.20E-35 | 40.40866306 |
| 65 | rs34042070 | G | C | 0.1947 | 0.187927 | 0.0485227 | 0.00268011 | 2.90E-73 | 100.0680859 |
| 66 | rs35511051 | A | C | 0.216 | 0.209985 | -0.0217862 | 0.00254546 | 1.10E-17 | 24.30558133 |
| 67 | rs35882350 | G | A | 0.272 | 0.261067 | 0.0139684 | 0.00237417 | 4.00E-09 | 13.35573923 |
| 68 | rs35980001 | G | GC | 0.7673 | 0.786561 | -0.0222891 | 0.00256639 | 3.80E-18 | 25.32788555 |
| 69 | rs3732359 | A | G | 0.6921 | 0.78012 | -0.0173556 | 0.00252038 | 5.70E-12 | 16.26815931 |
| 70 | rs3822855 | T | G | 0.4256 | 0.40166 | 0.0178418 | 0.00212447 | 4.50E-17 | 33.90347345 |
| 71 | rs3823376 | T | C | 0.4554 | 0.502179 | 0.0172774 | 0.0020812 | 1.00E-16 | 34.46060489 |
| 72 | rs4263041 | G | A | 0.3175 | 0.283209 | -0.0699797 | 0.00251019 | 4.90E-171 | 315.7691943 |
| 73 | rs4307732 | A | G | 0.157 | 0.105876 | 0.0448315 | 0.0033985 | 9.80E-40 | 32.94941998 |
| 74 | rs438568 | G | A | 0.664 | 0.609246 | 0.0124777 | 0.00213986 | 5.50E-09 | 16.18968731 |
| 75 | rs440677 | A | G | 0.5424 | 0.623063 | -0.0159238 | 0.00215434 | 1.50E-13 | 25.66368363 |
| 76 | rs4666384 | G | A | 0.7201 | 0.678914 | -0.016633 | 0.00224003 | 1.10E-13 | 24.03927477 |
| 77 | rs472495 | T | G | 0.5767 | 0.648959 | 0.0425743 | 0.00218093 | 7.30E-85 | 173.6944344 |
| 78 | rs4782568 | G | C | 0.4383 | 0.450902 | -0.0165059 | 0.00210805 | 4.90E-15 | 30.36033647 |
| 79 | rs4930163 | A | G | 0.1433 | 0.159456 | 0.0174597 | 0.00285203 | 9.20E-10 | 10.0462648 |
| 80 | rs4954192 | T | C | 0.4073 | 0.373474 | 0.0146629 | 0.00213654 | 6.70E-12 | 22.04280783 |
| 81 | rs4970834 | T | C | 0.196 | 0.186642 | -0.105303 | 0.00268179 | 1.00E-200 | 468.6113665 |
| 82 | rs5112 | G | C | 0.5831 | 0.533387 | 0.0277824 | 0.00222587 | 9.40E-36 | 77.56104758 |
| 83 | rs516316 | C | G | 0.6256 | 0.507935 | 0.0296183 | 0.00206761 | 1.50E-46 | 102.5989174 |
| 84 | rs556107 | T | C | 0.5675 | 0.523338 | 0.0351712 | 0.00208846 | 1.20E-63 | 141.5410409 |
| 85 | rs55714927 | T | C | 0.2643 | 0.190364 | -0.0264314 | 0.00265551 | 2.40E-23 | 30.54057536 |
| 86 | rs55831924 | T | C | 0.3327 | 0.361448 | 0.0156761 | 0.0021829 | 6.90E-13 | 23.80685116 |
| 87 | rs56130071 | C | G | 0.2146 | 0.216984 | 0.0331798 | 0.00253796 | 4.70E-39 | 58.0846812 |
| 88 | rs5843957 | TG | T | 0.6692 | 0.570884 | 0.0137065 | 0.00210922 | 8.10E-11 | 20.69094754 |
| 89 | rs597808 | G | A | 0.5853 | 0.515915 | 0.0271066 | 0.00209151 | 2.10E-38 | 83.91521761 |
| 90 | rs6074012 | C | T | 0.5102 | 0.523914 | 0.0116537 | 0.00208962 | 2.40E-08 | 15.5160714 |
| 91 | rs61003864 | C | T | 0.2195 | 0.187128 | 0.0155966 | 0.00267616 | 5.60E-09 | 10.33320079 |
| 92 | rs62033400 | G | A | 0.4019 | 0.394662 | -0.0144167 | 0.00213447 | 1.40E-11 | 21.79838781 |
| 93 | rs6475606 | T | C | 0.4136 | 0.48447 | -0.0202689 | 0.00208367 | 2.30E-22 | 47.27134254 |
| 94 | rs6495122 | C | A | 0.4753 | 0.591279 | 0.0143852 | 0.00211976 | 1.20E-11 | 22.26013522 |
| 95 | rs6544713 | C | T | 0.7816 | 0.676864 | -0.0537152 | 0.00222274 | 5.00E-129 | 255.613486 |
| 96 | rs6560499 | A | G | 0.6252 | 0.576007 | -0.0122028 | 0.00212189 | 8.90E-09 | 16.15487686 |
| 97 | rs6602912 | G | T | 0.2626 | 0.284854 | 0.0221997 | 0.00231001 | 7.20E-22 | 37.63125024 |
| 98 | rs6680227 | A | G | 0.05001 | 0.035237 | -0.0746581 | 0.00563322 | 4.30E-40 | 11.94262524 |
| 99 | rs6709904 | G | A | 0.1024 | 0.112585 | -0.043441 | 0.00329601 | 1.10E-39 | 34.71299834 |
| 100 | rs6732741 | A | T | 0.1192 | 0.119324 | -0.0280985 | 0.00322423 | 2.90E-18 | 15.96253887 |
| 101 | rs6874202 | C | T | 0.6678 | 0.634269 | 0.032331 | 0.00216292 | 1.60E-50 | 103.6866514 |
| 102 | rs7202323 | G | T | 0.2015 | 0.229741 | -0.0255365 | 0.00247732 | 6.50E-25 | 37.60961743 |
| 103 | rs72631343 | G | C | 0.1426 | 0.128894 | -0.0292893 | 0.00311032 | 4.60E-21 | 19.91403199 |
| 104 | rs7569317 | C | T | 0.5179 | 0.530638 | 0.017881 | 0.00208232 | 8.90E-18 | 36.73323468 |
| 105 | rs7707394 | A | G | 0.3732 | 0.357329 | 0.040448 | 0.00217281 | 2.40E-77 | 159.2181319 |
| 106 | rs7734476 | A | G | 0.5056 | 0.550077 | 0.0187595 | 0.00209401 | 3.30E-19 | 39.72957183 |
| 107 | rs7746081 | A | G | 0.3701 | 0.304113 | -0.0234725 | 0.00226701 | 4.00E-25 | 45.379352 |
| 108 | rs77542162 | G | A | 0.006795 | 0.022495 | 0.128484 | 0.0070401 | 2.10E-74 | 14.64832243 |
| 109 | rs7776054 | G | A | 0.3406 | 0.261313 | -0.0162316 | 0.00237493 | 8.20E-12 | 18.03384406 |
| 110 | rs78508096 | A | G | 0.2871 | 0.226194 | 0.0176869 | 0.00249323 | 1.30E-12 | 17.61721755 |
| 111 | rs79220007 | C | T | 0.03725 | 0.076135 | -0.057338 | 0.00392142 | 2.00E-48 | 30.07799636 |
| 112 | rs8107974 | T | A | 0.06479 | 0.075765 | -0.105058 | 0.00391919 | 2.70E-158 | 100.6569812 |
| 113 | rs869412 | C | T | 0.2675 | 0.225684 | -0.0142314 | 0.00250093 | 1.30E-08 | 11.31749636 |
| 114 | rs880315 | C | T | 0.4134 | 0.34024 | -0.0152225 | 0.00220678 | 5.30E-12 | 21.36359431 |
| 115 | rs9289196 | C | T | 0.1492 | 0.173786 | 0.0173702 | 0.00275673 | 3.00E-10 | 11.4016518 |
| 116 | rs934197 | A | G | 0.2807 | 0.33544 | 0.0832348 | 0.00220298 | 1.00E-200 | 637.3730849 |
| 117 | rs9471968 | G | A | 0.4374 | 0.545696 | -0.0116148 | 0.00209345 | 2.90E-08 | 15.26295725 |
| 118 | rs9496567 | A | G | 0.2077 | 0.243458 | -0.0174316 | 0.00243272 | 7.80E-13 | 18.91446297 |
| 119 | rs960596 | T | C | 0.3672 | 0.33932 | 0.0135068 | 0.00221758 | 1.10E-09 | 16.63379658 |
| 120 | rs964184 | C | G | 0.854 | 0.866409 | -0.0575823 | 0.00306356 | 8.20E-79 | 81.79637233 |
| 121 | rs9832727 | G | C | 0.3435 | 0.339636 | -0.0147604 | 0.00220388 | 2.10E-11 | 20.12173073 |
| 122 | rs9834932 | G | A | 0.08202 | 0.08883 | -0.0322172 | 0.00366408 | 1.50E-18 | 12.51541133 |
| 123 | rs9884390 | C | T | 0.2312 | 0.234121 | 0.0251489 | 0.00249163 | 5.90E-24 | 36.53721652 |
| 124 | rs990619 | G | C | 0.5758 | 0.523638 | -0.0118015 | 0.00208525 | 1.50E-08 | 15.97975822 |
| 125 | rs9929977 | A | T | 0.4069 | 0.369846 | 0.0167269 | 0.00216129 | 1.00E-14 | 27.92080942 |
| 126 | rs9987289 | G | A | 0.8574 | 0.908808 | 0.0453824 | 0.00362169 | 5.10E-36 | 26.02764859 |
| **Exposures** | **SNP** | **Effect**  **allele** | **Other**  **allele** | **Eaf**  **Outcomes** | **Eaf**  **Exposures** | **Beta**  **Exposures** | **Se**  **Exposures** | **P**  **Exposures** | **F statistic** |
| **HDL-C** |  |  |  |  |  |  |  |  |  |
| 1 | rs10053349 | C | T | 0.4351 | 0.390258 | 0.0120071 | 0.00195336 | 7.90E-10 | 17.98277785 |
| 2 | rs10108282 | A | T | 0.2277 | 0.205724 | 0.0168525 | 0.00234262 | 6.30E-13 | 16.9132632 |
| 3 | rs10119644 | A | T | 0.4736 | 0.493902 | 0.013468 | 0.00191042 | 1.80E-12 | 24.84725792 |
| 4 | rs10162642 | A | G | 0.1451 | 0.211684 | -0.0479223 | 0.0023419 | 4.60E-93 | 139.7993372 |
| 5 | rs10233430 | C | T | 0.4449 | 0.427019 | -0.0204944 | 0.00192772 | 2.10E-26 | 55.31682503 |
| 6 | rs1045241 | T | C | 0.3132 | 0.271252 | 0.0164171 | 0.00215576 | 2.60E-14 | 22.92951615 |
| 7 | rs1047891 | A | C | 0.3233 | 0.315582 | -0.0189073 | 0.00204367 | 2.20E-20 | 36.97760148 |
| 8 | rs10504477 | C | T | 0.4313 | 0.412192 | -0.0150661 | 0.00193097 | 6.10E-15 | 29.50157838 |
| 9 | rs10513801 | G | T | 0.1008 | 0.137151 | -0.0303325 | 0.00277034 | 6.70E-28 | 28.37542648 |
| 10 | rs1055582 | T | C | 0.4091 | 0.505231 | 0.0140251 | 0.00190973 | 2.10E-13 | 26.96604779 |
| 11 | rs10750766 | A | C | 0.7376 | 0.709866 | -0.0185663 | 0.00209928 | 9.20E-19 | 32.22162795 |
| 12 | rs10774439 | A | G | 0.7612 | 0.814891 | 0.020513 | 0.00248318 | 1.40E-16 | 20.58822912 |
| 13 | rs10786114 | T | C | 0.8577 | 0.874851 | 0.0238735 | 0.00288231 | 1.20E-16 | 15.02298674 |
| 14 | rs1083470 | A | G | 0.5822 | 0.615717 | 0.0115829 | 0.00195974 | 3.40E-09 | 16.53162844 |
| 15 | rs11021232 | C | T | 0.1711 | 0.18096 | -0.0167378 | 0.00248678 | 1.70E-11 | 13.42925746 |
| 16 | rs11045171 | G | A | 0.2176 | 0.197605 | 0.0283661 | 0.00240558 | 4.30E-32 | 44.09821883 |
| 17 | rs11171710 | A | G | 0.4482 | 0.44814 | -0.0114694 | 0.00192933 | 2.80E-09 | 17.48065209 |
| 18 | rs112001035 | A | G | 0.08051 | 0.060492 | -0.0466304 | 0.00408312 | 3.30E-30 | 14.82505129 |
| 19 | rs11218738 | A | G | 0.2154 | 0.249489 | 0.0234498 | 0.00219817 | 1.40E-26 | 42.62239657 |
| 20 | rs11239536 | A | T | 0.2815 | 0.240967 | 0.0287501 | 0.00223129 | 5.50E-38 | 60.74045697 |
| 21 | rs11254464 | C | T | 0.4165 | 0.423107 | 0.0127581 | 0.00193081 | 3.90E-11 | 21.31518794 |
| 22 | rs1125873 | T | A | 0.4329 | 0.492135 | 0.0159741 | 0.00192237 | 9.60E-17 | 34.51885091 |
| 23 | rs1132274 | A | C | 0.1362 | 0.154029 | -0.0217809 | 0.00263979 | 1.60E-16 | 17.74262101 |
| 24 | rs113740515 | A | G | 0.1857 | 0.209452 | 0.0377752 | 0.00233951 | 1.20E-58 | 86.35692519 |
| 25 | rs11429307 | G | GT | 0.8652 | 0.808907 | 0.0308032 | 0.00243282 | 9.70E-37 | 49.56740956 |
| 26 | rs11614202 | G | A | 0.85 | 0.841919 | 0.0227709 | 0.00261356 | 3.00E-18 | 20.20668157 |
| 27 | rs11640494 | A | G | 0.3125 | 0.456655 | -0.015349 | 0.00189969 | 6.50E-16 | 32.39822878 |
| 28 | rs11664369 | T | C | 0.1998 | 0.267061 | -0.0229777 | 0.00215861 | 1.80E-26 | 44.36276247 |
| 29 | rs1168114 | G | A | 0.7482 | 0.65237 | 0.0155365 | 0.00199819 | 7.50E-15 | 27.42214186 |
| 30 | rs116843064 | A | G | 0.02767 | 0.019327 | 0.206178 | 0.0069176 | 3.40E-195 | 33.67646279 |
| 31 | rs11688682 | C | G | 0.251 | 0.270836 | 0.0147369 | 0.0022133 | 2.80E-11 | 17.51096123 |
| 32 | rs12046972 | C | T | 0.5847 | 0.564083 | -0.0148213 | 0.00191599 | 1.00E-14 | 29.43014645 |
| 33 | rs12205778 | A | G | 0.2424 | 0.258895 | 0.0162321 | 0.00217549 | 8.60E-14 | 21.36433884 |
| 34 | rs12229011 | T | C | 0.0749 | 0.097467 | -0.0259933 | 0.00322614 | 7.80E-16 | 11.42134453 |
| 35 | rs1225053 | C | T | 0.2608 | 0.264492 | -0.0150376 | 0.00216684 | 3.90E-12 | 18.73921105 |
| 36 | rs1240820 | A | G | 0.302 | 0.293164 | 0.0131835 | 0.00210043 | 3.50E-10 | 16.32750106 |
| 37 | rs12462109 | T | C | 0.3024 | 0.287067 | -0.0159539 | 0.00210812 | 3.80E-14 | 23.44380566 |
| 38 | rs12475332 | G | T | 0.2139 | 0.261038 | 0.012897 | 0.0021638 | 2.50E-09 | 13.70602713 |
| 39 | rs12575456 | A | G | 0.4052 | 0.321829 | 0.0445866 | 0.00203342 | 1.40E-106 | 209.9773562 |
| 40 | rs12686780 | T | C | 0.1988 | 0.174998 | -0.0161688 | 0.00251034 | 1.20E-10 | 11.97896502 |
| 41 | rs12705595 | A | G | 0.3903 | 0.37337 | 0.0110554 | 0.00198306 | 2.50E-08 | 14.54357907 |
| 42 | rs12740374 | T | G | 0.2145 | 0.221008 | 0.0288629 | 0.00229106 | 2.20E-36 | 54.6555916 |
| 43 | rs12781812 | T | G | 0.3077 | 0.400915 | 0.0107203 | 0.00194289 | 3.40E-08 | 14.62519861 |
| 44 | rs1281959 | G | C | 0.5522 | 0.525716 | 0.0146574 | 0.00190666 | 1.50E-14 | 29.47247234 |
| 45 | rs12926854 | G | A | 0.2291 | 0.269909 | 0.0122457 | 0.00213341 | 9.50E-09 | 12.98538247 |
| 46 | rs12928099 | A | C | 0.2823 | 0.29612 | 0.0214263 | 0.00206787 | 3.70E-25 | 44.76001565 |
| 47 | rs12986742 | C | T | 0.4173 | 0.475905 | -0.0106002 | 0.00191315 | 3.00E-08 | 15.31456513 |
| 48 | rs12998038 | T | C | 0.2837 | 0.258702 | 0.0131918 | 0.0021847 | 1.60E-09 | 13.98492028 |
| 49 | rs13087167 | C | G | 0.6375 | 0.632997 | 0.0167458 | 0.00198206 | 2.90E-17 | 33.16751816 |
| 50 | rs13107325 | T | C | 0.01403 | 0.074942 | -0.080346 | 0.00362445 | 7.00E-109 | 68.14588612 |
| 51 | rs13111599 | G | A | 0.7518 | 0.737196 | 0.0127921 | 0.00216664 | 3.50E-09 | 13.50726706 |
| 52 | rs13137144 | A | G | 0.4324 | 0.461454 | 0.0164073 | 0.00192431 | 1.50E-17 | 36.13616656 |
| 53 | rs13144151 | G | A | 0.846 | 0.850001 | 0.0178695 | 0.00270341 | 3.80E-11 | 11.14162647 |
| 54 | rs13235365 | T | C | 0.2432 | 0.273616 | 0.0258181 | 0.00214379 | 2.10E-33 | 57.66093355 |
| 55 | rs133015 | G | C | 0.4067 | 0.440063 | 0.0205993 | 0.00192854 | 1.20E-26 | 56.23283042 |
| 56 | rs13379043 | C | T | 0.1988 | 0.28042 | 0.0198764 | 0.00216717 | 4.70E-20 | 33.95016662 |
| 57 | rs13389219 | T | C | 0.3486 | 0.392619 | 0.0277324 | 0.00194608 | 4.50E-46 | 96.87640993 |
| 58 | rs1349852 | C | A | 0.5372 | 0.475293 | 0.0112127 | 0.00192565 | 5.80E-09 | 16.91180651 |
| 59 | rs1395221 | T | G | 0.4298 | 0.397646 | -0.0111888 | 0.00195359 | 1.00E-08 | 15.71423819 |
| 60 | rs141062196 | A | G | 0.136 | 0.194436 | -0.0188553 | 0.00241089 | 5.20E-15 | 19.16184296 |
| 61 | rs1412234 | C | T | 0.3754 | 0.327133 | -0.011989 | 0.00203583 | 3.90E-09 | 15.26795062 |
| 62 | rs1431659 | G | A | 0.7588 | 0.728463 | 0.0127918 | 0.00214104 | 2.30E-09 | 14.12190549 |
| 63 | rs1446585 | G | A | 0.3484 | 0.244295 | 0.0167827 | 0.00217146 | 1.10E-14 | 22.05659293 |
| 64 | rs145947882 | C | A | 0.0301 | 0.026042 | -0.164935 | 0.00609907 | 4.70E-161 | 37.1005809 |
| 65 | rs1471251 | T | A | 0.3965 | 0.398159 | -0.0193662 | 0.00195327 | 3.60E-23 | 47.11737351 |
| 66 | rs147627829 | A | G | 0.02388 | 0.043706 | -0.0535763 | 0.00468184 | 2.50E-30 | 10.94673001 |
| 67 | rs150224153 | T | C | 0.07636 | 0.029665 | -0.0934689 | 0.00575879 | 3.10E-59 | 15.16638164 |
| 68 | rs150844304 | C | A | 0.005039 | 0.025907 | -0.090543 | 0.00599215 | 1.40E-51 | 11.52397188 |
| 69 | rs1534696 | A | C | 0.564 | 0.540992 | 0.0166573 | 0.0019113 | 2.90E-18 | 37.72515186 |
| 70 | rs1601934 | A | G | 0.6504 | 0.687495 | -0.10102 | 0.00207033 | 1.00E-200 | 1025.63124 |
| 71 | rs1601934 | A | G | 0.002598 | 0.687495 | -0.10102 | 0.00207033 | 1.00E-200 | 1025.63124 |
| 72 | rs16928809 | A | G | 0.07427 | 0.093089 | -0.0262633 | 0.00329991 | 1.70E-15 | 10.69539374 |
| 73 | rs17138358 | C | G | 0.298 | 0.398392 | -0.0272272 | 0.00194811 | 2.20E-44 | 93.65516854 |
| 74 | rs17309930 | A | C | 0.2393 | 0.205132 | -0.0218885 | 0.00235533 | 1.50E-20 | 28.16534192 |
| 75 | rs17326656 | T | G | 0.1631 | 0.238666 | -0.0223923 | 0.00223885 | 1.50E-23 | 36.35633735 |
| 76 | rs174566 | G | A | 0.4139 | 0.350061 | -0.0562241 | 0.00199551 | 1.20E-174 | 361.5512984 |
| 77 | rs1760940 | C | A | 0.2169 | 0.24702 | 0.0121791 | 0.0022157 | 3.90E-08 | 11.23993929 |
| 78 | rs17713879 | A | G | 0.3821 | 0.364695 | 0.0138956 | 0.00197564 | 2.00E-12 | 22.92469474 |
| 79 | rs1771582 | G | T | 0.6301 | 0.557402 | 0.0129458 | 0.00197832 | 6.00E-11 | 21.12969536 |
| 80 | rs1862205 | A | G | 0.4125 | 0.404725 | 0.0113186 | 0.00194381 | 5.80E-09 | 16.33807505 |
| 81 | rs1955512 | A | G | 0.5864 | 0.575802 | 0.0109986 | 0.00197003 | 2.40E-08 | 15.22702771 |
| 82 | rs1970811 | C | T | 0.378 | 0.457743 | -0.0116112 | 0.00191759 | 1.40E-09 | 18.20191124 |
| 83 | rs201441 | G | T | 0.4982 | 0.580679 | -0.0109672 | 0.0019342 | 1.40E-08 | 15.65727505 |
| 84 | rs2066714 | C | T | 0.09405 | 0.128622 | 0.0465396 | 0.00284369 | 3.40E-60 | 60.04751517 |
| 85 | rs2068888 | A | G | 0.47 | 0.450441 | 0.0191803 | 0.0019152 | 1.30E-23 | 49.66106155 |
| 86 | rs2098368 | T | C | 0.6425 | 0.54591 | -0.0116029 | 0.00192757 | 1.80E-09 | 17.96482244 |
| 87 | rs2098918 | T | C | 0.3852 | 0.45513 | 0.0118698 | 0.00191595 | 5.80E-10 | 19.03685195 |
| 88 | rs2111216 | G | A | 0.5379 | 0.593934 | 0.0213297 | 0.00194338 | 5.00E-28 | 58.1137864 |
| 89 | rs2155220 | T | C | 0.3926 | 0.437951 | -0.0105204 | 0.00191315 | 3.80E-08 | 14.88709685 |
| 90 | rs2159607 | T | G | 0.9038 | 0.810874 | -0.0239454 | 0.00243213 | 7.20E-23 | 29.73274778 |
| 91 | rs2196808 | C | T | 0.7602 | 0.731961 | 0.0128638 | 0.00215716 | 2.50E-09 | 13.95412535 |
| 92 | rs2236464 | C | T | 0.1481 | 0.212102 | -0.0155836 | 0.00235595 | 3.70E-11 | 14.62385174 |
| 93 | rs2237035 | T | G | 0.3512 | 0.385886 | 0.0139172 | 0.00195824 | 1.20E-12 | 23.940516 |
| 94 | rs2247355 | T | C | 0.2475 | 0.182679 | 0.0206138 | 0.00245873 | 5.10E-17 | 20.99066182 |
| 95 | rs2256609 | G | A | 0.2956 | 0.189238 | -0.0328881 | 0.00244058 | 2.20E-41 | 55.72896344 |
| 96 | rs2268840 | C | T | 0.2426 | 0.22846 | 0.0173197 | 0.00226775 | 2.20E-14 | 20.56406427 |
| 97 | rs2271308 | C | T | 0.7399 | 0.733051 | 0.0278808 | 0.002156 | 3.00E-38 | 65.45964657 |
| 98 | rs2290866 | T | C | 0.3265 | 0.253153 | -0.0119226 | 0.00218189 | 4.60E-08 | 11.29096205 |
| 99 | rs2297409 | A | G | 0.1231 | 0.194635 | -0.0333565 | 0.00240571 | 1.00E-43 | 60.28104132 |
| 100 | rs2298214 | A | C | 0.5401 | 0.577365 | -0.01241 | 0.00194099 | 1.60E-10 | 19.95088545 |
| 101 | rs2298624 | T | C | 0.1721 | 0.132532 | 0.0300361 | 0.00280916 | 1.10E-26 | 26.28841818 |
| 102 | rs2298632 | T | C | 0.552 | 0.498073 | 0.0144252 | 0.00193744 | 9.70E-14 | 27.7190516 |
| 103 | rs2302263 | T | C | 0.128 | 0.088695 | -0.0364373 | 0.00335268 | 1.60E-27 | 19.09498999 |
| 104 | rs2307111 | C | T | 0.4272 | 0.394924 | 0.0190053 | 0.0019521 | 2.10E-22 | 45.30491731 |
| 105 | rs2339234 | A | G | 0.6538 | 0.683004 | -0.0118996 | 0.00205717 | 7.30E-09 | 14.48921356 |
| 106 | rs235314 | T | C | 0.4338 | 0.532212 | -0.0177425 | 0.00191993 | 2.40E-20 | 42.52713672 |
| 107 | rs2362541 | G | T | 0.4175 | 0.507519 | -0.0108666 | 0.00190325 | 1.10E-08 | 16.2960683 |
| 108 | rs2364723 | C | G | 0.333 | 0.319156 | 0.0120956 | 0.00204641 | 3.40E-09 | 15.1832356 |
| 109 | rs2417125 | G | A | 0.2214 | 0.284014 | -0.0131524 | 0.00211658 | 5.20E-10 | 15.70470017 |
| 110 | rs2435307 | T | C | 0.4927 | 0.48634 | 0.0162767 | 0.00190927 | 1.50E-17 | 36.31454822 |
| 111 | rs2498786 | G | C | 0.628 | 0.615979 | -0.0254741 | 0.00196826 | 2.60E-38 | 79.26230567 |
| 112 | rs2516331 | A | C | 0.2701 | 0.375524 | 0.0126835 | 0.00196658 | 1.10E-10 | 19.50999965 |
| 113 | rs2520096 | G | A | 0.3984 | 0.270192 | 0.0147205 | 0.00215114 | 7.70E-12 | 18.4687107 |
| 114 | rs254562 | G | A | 0.4118 | 0.409277 | -0.0114752 | 0.00194192 | 3.40E-09 | 16.88515598 |
| 115 | rs2586116 | G | C | 0.2447 | 0.259435 | -0.0162577 | 0.00217179 | 7.10E-14 | 21.53402922 |
| 116 | rs2642438 | G | A | 0.7157 | 0.702698 | 0.0276562 | 0.00207854 | 2.10E-40 | 73.98474338 |
| 117 | rs2645979 | A | G | 0.4347 | 0.357369 | 0.0114281 | 0.00198606 | 8.70E-09 | 15.20848941 |
| 118 | rs267738 | G | T | 0.1916 | 0.219354 | 0.0214529 | 0.00229757 | 9.90E-21 | 29.86029942 |
| 119 | rs2723065 | G | A | 0.3416 | 0.376773 | 0.0149623 | 0.00196239 | 2.40E-14 | 27.30293307 |
| 120 | rs2726111 | G | A | 0.6363 | 0.667447 | -0.0148127 | 0.00202533 | 2.60E-13 | 23.74695309 |
| 121 | rs2740488 | C | A | 0.1971 | 0.265328 | -0.0686515 | 0.00216181 | 1.00E-200 | 393.5431387 |
| 122 | rs2750411 | G | T | 0.4853 | 0.4919 | -0.0108405 | 0.0019091 | 1.40E-08 | 16.11804871 |
| 123 | rs2792751 | C | T | 0.6824 | 0.725031 | -0.0360998 | 0.0021336 | 3.20E-64 | 114.1759096 |
| 124 | rs2800710 | C | T | 0.5252 | 0.51821 | -0.0202674 | 0.00190382 | 1.80E-26 | 56.59741168 |
| 125 | rs2804894 | A | G | 0.7872 | 0.734831 | 0.0173063 | 0.0021841 | 2.30E-15 | 24.4696519 |
| 126 | rs2814982 | T | C | 0.1633 | 0.102216 | -0.0275938 | 0.00314209 | 1.60E-18 | 14.1553167 |
| 127 | rs28510484 | C | G | 0.17 | 0.170462 | -0.0154472 | 0.00253932 | 1.20E-09 | 10.46568442 |
| 128 | rs286965 | C | T | 0.6274 | 0.632135 | -0.0134141 | 0.00197553 | 1.10E-11 | 21.44396192 |
| 129 | rs2910949 | G | T | 0.2432 | 0.354888 | 0.0133996 | 0.00199892 | 2.00E-11 | 20.57639291 |
| 130 | rs2925979 | C | T | 0.6776 | 0.699999 | 0.037299 | 0.00205659 | 1.60E-73 | 138.1959958 |
| 131 | rs2943645 | T | C | 0.638 | 0.646663 | -0.0434706 | 0.00198833 | 5.90E-106 | 218.5466831 |
| 132 | rs2963468 | G | A | 0.1377 | 0.235297 | -0.0196653 | 0.00226256 | 3.60E-18 | 27.18737021 |
| 133 | rs2965169 | C | A | 0.3556 | 0.388738 | 0.0120831 | 0.00195448 | 6.30E-10 | 18.16460869 |
| 134 | rs3027167 | T | C | 0.7708 | 0.682038 | -0.0124258 | 0.00205083 | 1.40E-09 | 15.92275275 |
| 135 | rs308 | G | T | 0.02987 | 0.020639 | 0.12651 | 0.00669305 | 1.10E-79 | 14.44363162 |
| 136 | rs3184504 | C | T | 0.5912 | 0.517322 | 0.0265387 | 0.00190452 | 3.90E-44 | 96.99264017 |
| 137 | rs32578 | A | G | 0.3834 | 0.309015 | 0.0133235 | 0.00206513 | 1.10E-10 | 17.77612104 |
| 138 | rs34045894 | A | G | 0.1755 | 0.15624 | -0.0172909 | 0.00262706 | 4.60E-11 | 11.42211434 |
| 139 | rs34138141 | T | G | 0.2219 | 0.280655 | -0.0175318 | 0.00212461 | 1.60E-16 | 27.49552344 |
| 140 | rs343 | A | C | 0.06338 | 0.08307 | 0.133778 | 0.00346421 | 1.00E-200 | 227.3073783 |
| 141 | rs34940374 | A | G | 0.247 | 0.182973 | -0.0170378 | 0.00247025 | 5.30E-12 | 14.22367338 |
| 142 | rs35493868 | G | C | 0.18 | 0.202024 | 0.0372262 | 0.00237772 | 3.00E-55 | 79.0463115 |
| 143 | rs35980001 | G | GC | 0.7673 | 0.786507 | -0.118787 | 0.00234184 | 1.00E-200 | 865.899395 |
| 144 | rs36057735 | G | C | 0.17 | 0.199089 | -0.0302136 | 0.00238679 | 1.00E-36 | 51.10823059 |
| 145 | rs367070 | G | A | 0.2991 | 0.225571 | 0.0417949 | 0.00228709 | 1.30E-74 | 116.7073607 |
| 146 | rs3745683 | A | G | 0.09038 | 0.074638 | -0.0546353 | 0.00362928 | 3.20E-51 | 31.30681389 |
| 147 | rs3746915 | G | A | 0.6269 | 0.580939 | 0.0108813 | 0.00194098 | 2.10E-08 | 15.30282187 |
| 148 | rs3747973 | G | A | 0.6135 | 0.592542 | 0.0141665 | 0.00193802 | 2.70E-13 | 25.80276203 |
| 149 | rs3768321 | T | G | 0.1645 | 0.196691 | -0.0452296 | 0.00239482 | 1.50E-79 | 112.7498344 |
| 150 | rs3794752 | C | T | 0.2818 | 0.280634 | 0.0128326 | 0.00213876 | 2.00E-09 | 14.53579788 |
| 151 | rs3814883 | T | C | 0.4115 | 0.482467 | -0.0153035 | 0.00189151 | 5.90E-16 | 32.69135376 |
| 152 | rs3924313 | A | G | 0.262 | 0.322221 | -0.0239168 | 0.00204761 | 1.60E-31 | 59.5998997 |
| 153 | rs407133 | C | G | 0.5817 | 0.558791 | -0.0105618 | 0.00193239 | 4.60E-08 | 14.73071286 |
| 154 | rs4074448 | A | G | 0.4853 | 0.589254 | 0.015131 | 0.00196355 | 1.30E-14 | 28.7465656 |
| 155 | rs41272086 | A | G | 0.1011 | 0.105505 | -0.0567846 | 0.00310319 | 8.50E-75 | 63.21078263 |
| 156 | rs429358 | C | T | 0.1831 | 0.154325 | -0.0756554 | 0.00263871 | 8.71E-181 | 214.6816443 |
| 157 | rs4330777 | A | G | 0.4645 | 0.474907 | -0.0197681 | 0.00189019 | 1.30E-25 | 54.55696895 |
| 158 | rs454968 | C | T | 0.6157 | 0.644812 | 0.0110337 | 0.00199469 | 3.20E-08 | 14.01605628 |
| 159 | rs460428 | C | T | 0.1969 | 0.230673 | -0.0143462 | 0.00226779 | 2.50E-10 | 14.20427086 |
| 160 | rs4614 | G | A | 0.3028 | 0.406222 | -0.0178217 | 0.00193829 | 3.80E-20 | 40.78683299 |
| 161 | rs4650994 | A | G | 0.4665 | 0.531842 | -0.018056 | 0.00190061 | 2.10E-21 | 44.94778764 |
| 162 | rs4691379 | T | C | 0.2447 | 0.318939 | 0.0118717 | 0.00204326 | 6.20E-09 | 14.66613958 |
| 163 | rs4784709 | A | T | 0.9807 | 0.959159 | -0.0747244 | 0.00477284 | 3.00E-55 | 19.20465845 |
| 164 | rs4804101 | T | G | 0.4332 | 0.438994 | -0.0139042 | 0.00192656 | 5.30E-13 | 25.65715243 |
| 165 | rs4855582 | T | C | 0.428 | 0.429621 | 0.0111652 | 0.00192369 | 6.50E-09 | 16.51039502 |
| 166 | rs4871603 | T | C | 0.6625 | 0.653471 | 0.0360061 | 0.00199228 | 5.20E-73 | 147.9804137 |
| 167 | rs4871624 | G | T | 0.2671 | 0.286726 | -0.020416 | 0.00211032 | 3.90E-22 | 38.28575008 |
| 168 | rs4875043 | C | A | 0.2329 | 0.216821 | -0.0149745 | 0.0023285 | 1.30E-10 | 14.04615639 |
| 169 | rs4917675 | C | T | 0.2749 | 0.254595 | 0.014562 | 0.00219637 | 3.40E-11 | 16.68472306 |
| 170 | rs4930352 | T | G | 0.5282 | 0.494128 | 0.0161709 | 0.00193852 | 7.30E-17 | 34.79150105 |
| 171 | rs4969141 | T | C | 0.6192 | 0.489676 | 0.0296373 | 0.00191152 | 3.20E-54 | 120.1797866 |
| 172 | rs532436 | A | G | 0.2006 | 0.184606 | 0.0231547 | 0.00245365 | 3.80E-21 | 26.81162184 |
| 173 | rs557933 | C | A | 0.5604 | 0.520349 | 0.0152472 | 0.00190783 | 1.30E-15 | 31.88475249 |
| 174 | rs55935382 | A | C | 0.3135 | 0.32503 | 0.01764 | 0.00203822 | 4.90E-18 | 32.86746003 |
| 175 | rs559355 | T | A | 0.2298 | 0.1573 | -0.0349311 | 0.00261254 | 9.00E-41 | 47.40008798 |
| 176 | rs564832 | C | T | 0.3163 | 0.315005 | -0.011708 | 0.00205455 | 1.20E-08 | 14.01456943 |
| 177 | rs58123204 | G | A | 0.1318 | 0.153445 | -0.0184574 | 0.00264401 | 2.90E-12 | 12.66089012 |
| 178 | rs59104589 | T | C | 0.3964 | 0.358286 | 0.0150155 | 0.0019816 | 3.50E-14 | 26.40433262 |
| 179 | rs59781045 | T | C | 0.08123 | 0.068117 | 0.074037 | 0.0037879 | 4.50E-85 | 48.50622871 |
| 180 | rs6018652 | A | G | 0.7877 | 0.792888 | 0.0259271 | 0.0023605 | 4.60E-28 | 39.62665658 |
| 181 | rs6066148 | C | G | 0.1994 | 0.259964 | 0.0133124 | 0.00218121 | 1.00E-09 | 14.33266893 |
| 182 | rs6073958 | C | T | 0.176 | 0.198804 | -0.0609299 | 0.00239207 | 4.10E-143 | 206.7888741 |
| 183 | rs6075860 | A | G | 0.6225 | 0.56549 | -0.0128 | 0.00192763 | 3.10E-11 | 21.66947721 |
| 184 | rs6123685 | A | G | 0.2509 | 0.254745 | 0.0159783 | 0.0021911 | 3.00E-13 | 20.192815 |
| 185 | rs61352607 | T | G | 0.2328 | 0.240851 | 0.0306111 | 0.00222442 | 4.40E-43 | 69.26312382 |
| 186 | rs6142206 | A | G | 0.4712 | 0.42072 | -0.0161106 | 0.00193468 | 8.30E-17 | 33.80267147 |
| 187 | rs61805075 | A | G | 0.2873 | 0.328694 | -0.0257183 | 0.00202534 | 6.00E-37 | 71.17136567 |
| 188 | rs62102718 | T | A | 0.2879 | 0.286413 | -0.0236953 | 0.0021125 | 3.40E-29 | 51.43441266 |
| 189 | rs62117487 | G | A | 0.03474 | 0.057038 | 0.0457487 | 0.00411744 | 1.10E-28 | 13.28016774 |
| 190 | rs62271373 | A | T | 0.04392 | 0.059972 | -0.0406667 | 0.00408672 | 2.50E-23 | 11.1649625 |
| 191 | rs62428831 | C | T | 0.1872 | 0.141547 | 0.0178151 | 0.00275536 | 1.00E-10 | 10.15958084 |
| 192 | rs635769 | C | T | 0.5719 | 0.628046 | 0.0197069 | 0.00196961 | 1.40E-23 | 46.77728019 |
| 193 | rs6460894 | C | T | 0.2719 | 0.339247 | -0.0121653 | 0.00201452 | 1.60E-09 | 16.34944679 |
| 194 | rs6469605 | T | C | 0.6938 | 0.568501 | 0.0316197 | 0.00191959 | 5.80E-61 | 133.1620736 |
| 195 | rs6693842 | C | T | 0.4619 | 0.364844 | 0.013664 | 0.00199336 | 7.10E-12 | 21.77825614 |
| 196 | rs6705285 | T | G | 0.6765 | 0.609068 | 0.0115225 | 0.00191933 | 1.90E-09 | 17.16356237 |
| 197 | rs676210 | A | G | 0.2662 | 0.205227 | 0.0592694 | 0.00235237 | 4.50E-140 | 207.1943702 |
| 198 | rs6762415 | G | T | 0.4405 | 0.535292 | -0.0108964 | 0.00191344 | 1.20E-08 | 16.13438707 |
| 199 | rs6765484 | T | C | 0.5608 | 0.472827 | 0.0225207 | 0.00190864 | 3.90E-32 | 69.41824488 |
| 200 | rs680321 | C | T | 0.4292 | 0.456556 | 0.0108517 | 0.00191658 | 1.50E-08 | 15.90872799 |
| 201 | rs6824451 | A | G | 0.5218 | 0.464071 | -0.0200444 | 0.00191167 | 1.00E-25 | 54.69386386 |
| 202 | rs686030 | A | C | 0.8813 | 0.858823 | 0.0498241 | 0.00273951 | 6.50E-74 | 80.22594691 |
| 203 | rs689183 | T | G | 0.6928 | 0.751408 | -0.0158298 | 0.00220903 | 7.70E-13 | 19.1848789 |
| 204 | rs6934962 | T | C | 0.4251 | 0.40151 | 0.0161905 | 0.00194099 | 7.30E-17 | 33.44190351 |
| 205 | rs6939861 | A | G | 0.2603 | 0.261916 | -0.0141972 | 0.00219368 | 9.70E-11 | 16.19466342 |
| 206 | rs7036107 | G | A | 0.5141 | 0.51098 | -0.0122524 | 0.00194377 | 2.90E-10 | 19.85785346 |
| 207 | rs703966 | A | G | 0.3798 | 0.418881 | 0.0156949 | 0.00193455 | 4.90E-16 | 32.04610292 |
| 208 | rs7158166 | C | T | 0.5973 | 0.593856 | 0.0140932 | 0.00195101 | 5.10E-13 | 25.17191972 |
| 209 | rs7170463 | G | A | 0.3476 | 0.311432 | 0.018911 | 0.0020591 | 4.20E-20 | 36.17853055 |
| 210 | rs7186799 | C | A | 0.4538 | 0.437388 | -0.0220239 | 0.00190185 | 5.20E-31 | 66.01013119 |
| 211 | rs7218647 | A | G | 0.6315 | 0.559041 | 0.0109209 | 0.00192614 | 1.40E-08 | 15.84995076 |
| 212 | rs7251640 | C | T | 0.2168 | 0.194019 | 0.0141902 | 0.00242811 | 5.10E-09 | 10.68190506 |
| 213 | rs72647336 | A | G | 0.03847 | 0.056628 | -0.0440494 | 0.00442834 | 2.60E-23 | 10.57186225 |
| 214 | rs7281183 | A | G | 0.7142 | 0.737142 | -0.01272 | 0.00218963 | 6.30E-09 | 13.07817111 |
| 215 | rs72926946 | A | C | 0.2762 | 0.296376 | -0.0206565 | 0.00208041 | 3.10E-23 | 41.12172657 |
| 216 | rs72964564 | C | A | 0.1762 | 0.250244 | -0.0125748 | 0.00220598 | 1.20E-08 | 12.19336599 |
| 217 | rs73243877 | G | A | 0.1429 | 0.168086 | -0.0253693 | 0.00254909 | 2.50E-23 | 27.70219827 |
| 218 | rs7488780 | C | G | 0.255 | 0.204263 | 0.0145459 | 0.00237104 | 8.50E-10 | 12.23499205 |
| 219 | rs7583067 | T | C | 0.245 | 0.240114 | 0.0145946 | 0.00223535 | 6.60E-11 | 15.55621097 |
| 220 | rs7622114 | A | C | 0.496 | 0.581017 | 0.0115687 | 0.00193999 | 2.50E-09 | 17.31417638 |
| 221 | rs76247316 | C | T | 0.4882 | 0.479856 | -0.0113866 | 0.0019083 | 2.40E-09 | 17.77363697 |
| 222 | rs7665587 | C | T | 0.4395 | 0.42208 | 0.0139886 | 0.00193372 | 4.70E-13 | 25.53166388 |
| 223 | rs771481 | A | T | 0.1811 | 0.18382 | 0.0288148 | 0.0024568 | 9.10E-32 | 41.28027176 |
| 224 | rs7725218 | A | G | 0.3616 | 0.338422 | -0.0122469 | 0.00201433 | 1.20E-09 | 16.55298746 |
| 225 | rs77320712 | T | G | 0.2049 | 0.235452 | -0.0127407 | 0.0022658 | 1.90E-08 | 11.38388918 |
| 226 | rs77605964 | A | G | 0.1814 | 0.226924 | 0.0170396 | 0.00227819 | 7.50E-14 | 19.62865214 |
| 227 | rs7794796 | T | C | 0.3556 | 0.332335 | -0.016879 | 0.00203772 | 1.20E-16 | 30.45086862 |
| 228 | rs77960347 | G | A | 0.007282 | 0.013349 | 0.290966 | 0.00830188 | 1.00E-200 | 32.359851 |
| 229 | rs78058190 | A | G | 0.08195 | 0.050415 | -0.0783702 | 0.00490443 | 1.80E-57 | 24.44964616 |
| 230 | rs7817574 | C | T | 0.2473 | 0.185003 | 0.0331985 | 0.00243942 | 3.50E-42 | 55.85813619 |
| 231 | rs7826177 | C | T | 0.637 | 0.635428 | 0.0112399 | 0.00197917 | 1.40E-08 | 14.94349788 |
| 232 | rs7853377 | G | A | 0.2117 | 0.216224 | 0.0152447 | 0.0023168 | 4.70E-11 | 14.67574501 |
| 233 | rs7924036 | T | G | 0.5138 | 0.503377 | 0.0138679 | 0.00190752 | 3.60E-13 | 26.42771761 |
| 234 | rs79600951 | G | C | 0.0948 | 0.092057 | -0.106795 | 0.00325908 | 1.00E-200 | 179.5759087 |
| 235 | rs80005209 | G | T | 0.01897 | 0.029614 | -0.144197 | 0.00563687 | 2.50E-144 | 37.61372023 |
| 236 | rs8007841 | C | T | 0.6334 | 0.656767 | -0.0128714 | 0.0020177 | 1.80E-10 | 18.34789798 |
| 237 | rs8014289 | G | A | 0.664 | 0.562282 | 0.0151048 | 0.00192712 | 4.60E-15 | 30.24275755 |
| 238 | rs8081548 | A | T | 0.6988 | 0.658521 | 0.01845 | 0.00202095 | 6.90E-20 | 37.4872326 |
| 239 | rs8086351 | G | C | 0.8295 | 0.824212 | 0.0839587 | 0.0025068 | 1.00E-200 | 325.3101822 |
| 240 | rs830620 | T | C | 0.4364 | 0.415386 | 0.0149538 | 0.00193085 | 9.60E-15 | 29.13307291 |
| 241 | rs907866 | A | G | 0.5516 | 0.445487 | -0.0183154 | 0.00191879 | 1.40E-21 | 45.0195278 |
| 242 | rs921919 | A | G | 0.5708 | 0.669162 | -0.041665 | 0.00206455 | 1.40E-90 | 180.4099091 |
| 243 | rs9327468 | A | C | 0.8492 | 0.755398 | -0.0148994 | 0.00221907 | 1.90E-11 | 16.66008245 |
| 244 | rs9347737 | G | A | 0.4506 | 0.428295 | -0.0134784 | 0.00194715 | 4.40E-12 | 23.46637102 |
| 245 | rs9465693 | A | C | 0.3296 | 0.304872 | -0.0121004 | 0.00208231 | 6.20E-09 | 14.31311762 |
| 246 | rs9604045 | T | G | 0.1715 | 0.253572 | 0.0175151 | 0.00228914 | 2.00E-14 | 22.16263658 |
| 247 | rs9622830 | G | C | 0.361 | 0.354212 | -0.0163821 | 0.00199965 | 2.60E-16 | 30.70755726 |
| 248 | rs964184 | C | G | 0.854 | 0.866364 | 0.10544 | 0.00279456 | 1.00E-200 | 329.9059741 |
| 249 | rs9647335 | T | A | 0.1193 | 0.191928 | 0.0277595 | 0.00242471 | 2.40E-30 | 40.65964908 |
| 250 | rs968050 | T | C | 0.48 | 0.48238 | 0.0136601 | 0.00190807 | 8.10E-13 | 25.59616236 |
| 251 | rs983663 | G | A | 0.269 | 0.251682 | -0.0143608 | 0.0022075 | 7.70E-11 | 15.94186681 |
| 252 | rs9916613 | A | T | 0.3354 | 0.356493 | -0.0133119 | 0.00199143 | 2.30E-11 | 20.50237498 |
| 253 | rs9933509 | C | T | 0.4121 | 0.413899 | -0.0140121 | 0.00191313 | 2.40E-13 | 26.02796176 |
| 254 | rs998584 | A | C | 0.4822 | 0.482733 | -0.0341901 | 0.00190975 | 1.10E-71 | 160.1287961 |
| 255 | rs9987289 | G | A | 0.8574 | 0.908737 | 0.0872902 | 0.00329366 | 9.10E-155 | 116.5358419 |
| 256 | rs9989419 | G | A | 0.6153 | 0.605917 | 0.143765 | 0.00192633 | 1.00E-200 | 2677.58293 |
| **Exposures** | **SNP** | **Effect**  **allele** | **Other**  **allele** | **Eaf**  **Outcomes** | **Eaf**  **Exposures** | **Beta**  **Exposures** | **Se**  **Exposures** | **P**  **Exposures** | **F statistic** |
| **ApoA-I** |  |  |  |  |  |  |  |  |  |
| 1 | rs10023962 | G | T | 0.8832 | 0.816753 | 0.0176607 | 0.00254251 | 3.80E-12 | 14.44314722 |
| 2 | rs10170658 | T | G | 0.267 | 0.222908 | -0.0132874 | 0.00235794 | 1.70E-08 | 11.00151638 |
| 3 | rs10271082 | C | G | 0.4318 | 0.407793 | -0.0190331 | 0.00199937 | 1.70E-21 | 43.77459672 |
| 4 | rs1037117 | A | G | 0.2854 | 0.254731 | 0.0161057 | 0.00225649 | 9.50E-13 | 19.34355948 |
| 5 | rs10404380 | A | C | 0.5532 | 0.604801 | -0.0257904 | 0.00201461 | 1.60E-37 | 78.35682837 |
| 6 | rs1047891 | A | C | 0.3233 | 0.315663 | -0.0256666 | 0.00210473 | 3.30E-34 | 64.25935485 |
| 7 | rs10489044 | G | A | 0.1512 | 0.199084 | -0.0183462 | 0.00246586 | 1.00E-13 | 17.65330515 |
| 8 | rs10504477 | C | T | 0.4313 | 0.412293 | -0.0121174 | 0.00199318 | 1.20E-09 | 17.91184171 |
| 9 | rs1055582 | T | C | 0.4091 | 0.505137 | 0.0155609 | 0.00196562 | 2.40E-15 | 31.33479507 |
| 10 | rs10745954 | G | A | 0.6192 | 0.480326 | -0.0143687 | 0.0019633 | 2.50E-13 | 26.74151629 |
| 11 | rs10748165 | T | C | 0.4742 | 0.504155 | 0.0110061 | 0.00196411 | 2.10E-08 | 15.69966695 |
| 12 | rs10750766 | A | C | 0.7376 | 0.709869 | -0.0147925 | 0.00216197 | 7.80E-12 | 19.28436139 |
| 13 | rs10752898 | C | T | 0.5814 | 0.55762 | 0.0125396 | 0.00196775 | 1.90E-10 | 20.03601126 |
| 14 | rs10774439 | A | G | 0.7612 | 0.814838 | 0.0164484 | 0.00255731 | 1.30E-10 | 12.48375613 |
| 15 | rs10798615 | G | T | 0.4665 | 0.533322 | -0.0153188 | 0.00196392 | 6.20E-15 | 30.28795651 |
| 16 | rs1086056 | G | T | 0.821 | 0.844669 | -0.0188503 | 0.00271238 | 3.70E-12 | 12.67424054 |
| 17 | rs10876447 | A | G | 0.1315 | 0.154052 | -0.01939 | 0.00271564 | 9.30E-13 | 13.28815535 |
| 18 | rs10883451 | C | T | 0.39 | 0.496282 | 0.022096 | 0.00196056 | 1.80E-29 | 63.51568372 |
| 19 | rs10917383 | G | A | 0.8857 | 0.875246 | 0.0203915 | 0.00296088 | 5.70E-12 | 10.35811136 |
| 20 | rs11021232 | C | T | 0.1711 | 0.181005 | -0.0164557 | 0.00256026 | 1.30E-10 | 12.24835366 |
| 21 | rs11045172 | C | A | 0.2176 | 0.197912 | 0.0222366 | 0.00247597 | 2.70E-19 | 25.60921285 |
| 22 | rs11057390 | G | T | 0.2462 | 0.295431 | 0.0190026 | 0.00215664 | 1.20E-18 | 32.3231609 |
| 23 | rs11065987 | G | A | 0.3829 | 0.416331 | -0.0140735 | 0.00198803 | 1.50E-12 | 24.35669923 |
| 24 | rs11067231 | A | C | 0.474 | 0.523895 | 0.021994 | 0.00196177 | 3.60E-29 | 62.71281702 |
| 25 | rs11089620 | G | C | 0.2956 | 0.188588 | -0.0321639 | 0.00251317 | 1.70E-37 | 50.1339948 |
| 26 | rs11226108 | C | G | 0.1823 | 0.191516 | -0.0146559 | 0.00249387 | 4.20E-09 | 10.69531195 |
| 27 | rs1125873 | T | A | 0.4329 | 0.492029 | 0.0122955 | 0.00197818 | 5.10E-10 | 19.31254516 |
| 28 | rs1132274 | A | C | 0.1362 | 0.154087 | -0.0287721 | 0.0027161 | 3.20E-26 | 29.25522273 |
| 29 | rs11429307 | G | GT | 0.8652 | 0.808887 | 0.0223154 | 0.00250419 | 5.00E-19 | 24.55318235 |
| 30 | rs11481448 | A | AT | 0.4143 | 0.497412 | 0.0114668 | 0.00197074 | 5.90E-09 | 16.92780585 |
| 31 | rs11632618 | A | G | 0.0485 | 0.06962 | 0.108049 | 0.00384587 | 1.10E-173 | 102.2792258 |
| 32 | rs11641548 | C | A | 0.2975 | 0.43959 | -0.0130768 | 0.00197499 | 3.60E-11 | 21.6012303 |
| 33 | rs1168124 | T | C | 0.7359 | 0.65073 | 0.0426112 | 0.00205353 | 1.20E-95 | 195.8175263 |
| 34 | rs11691486 | C | T | 0.245 | 0.240048 | 0.0141328 | 0.00230186 | 8.30E-10 | 13.75389949 |
| 35 | rs11870735 | T | C | 0.1285 | 0.180979 | -0.0152398 | 0.00254853 | 2.20E-09 | 10.60087593 |
| 36 | rs12044156 | C | G | 0.5845 | 0.563837 | -0.0119199 | 0.00197231 | 1.50E-09 | 17.96573103 |
| 37 | rs12229372 | C | T | 0.1548 | 0.10329 | 0.0296946 | 0.003302 | 2.40E-19 | 14.98150507 |
| 38 | rs12273363 | C | T | 0.2393 | 0.205682 | -0.0197428 | 0.00242496 | 3.90E-16 | 21.65961507 |
| 39 | rs12462109 | T | C | 0.3024 | 0.287067 | -0.0134097 | 0.00216974 | 6.40E-10 | 15.6350681 |
| 40 | rs1260326 | C | T | 0.6493 | 0.604673 | -0.0258112 | 0.00200095 | 4.50E-38 | 79.56766908 |
| 41 | rs12740374 | T | G | 0.2145 | 0.220913 | 0.0442974 | 0.00235869 | 1.10E-78 | 121.4461859 |
| 42 | rs12898210 | G | T | 0.1023 | 0.121583 | -0.0491359 | 0.00300967 | 6.40E-60 | 56.94090096 |
| 43 | rs12943517 | A | G | 0.2917 | 0.310724 | -0.0118613 | 0.00212624 | 2.40E-08 | 13.330624 |
| 44 | rs12977604 | G | C | 0.4721 | 0.499196 | 0.0373935 | 0.0020925 | 2.00E-71 | 159.7365762 |
| 45 | rs12987470 | T | A | 0.3092 | 0.294649 | 0.012042 | 0.00215213 | 2.20E-08 | 13.01405571 |
| 46 | rs13059175 | T | C | 0.1916 | 0.299999 | 0.0148346 | 0.00213401 | 3.60E-12 | 20.29679441 |
| 47 | rs13107325 | T | C | 0.01403 | 0.074994 | -0.071753 | 0.00372931 | 1.70E-82 | 51.36635432 |
| 48 | rs13108218 | G | A | 0.6773 | 0.614914 | -0.01333 | 0.00203151 | 5.30E-11 | 20.39129416 |
| 49 | rs1318175 | T | C | 0.1742 | 0.159965 | -0.0780529 | 0.00268206 | 3.40E-186 | 227.7417868 |
| 50 | rs133015 | G | C | 0.4067 | 0.44 | 0.0200344 | 0.00198398 | 5.60E-24 | 50.25764095 |
| 51 | rs13326165 | G | A | 0.8211 | 0.795376 | -0.0195454 | 0.00242685 | 8.00E-16 | 21.11462718 |
| 52 | rs13379043 | C | T | 0.1988 | 0.280373 | 0.0217854 | 0.00222966 | 1.50E-22 | 38.52722851 |
| 53 | rs1395221 | T | G | 0.4298 | 0.397633 | -0.0132838 | 0.00201044 | 3.90E-11 | 20.91495345 |
| 54 | rs1400362 | C | T | 0.7804 | 0.741488 | 0.0125215 | 0.00226712 | 3.30E-08 | 11.69470409 |
| 55 | rs141368429 | T | C | 0.02755 | 0.055929 | -0.0569584 | 0.00458365 | 1.90E-35 | 16.30722921 |
| 56 | rs144311893 | T | C | 0.007773 | 0.021987 | 0.122697 | 0.00690208 | 1.10E-70 | 13.59130984 |
| 57 | rs1446585 | G | A | 0.3484 | 0.244189 | 0.0185954 | 0.00223649 | 9.20E-17 | 25.51951438 |
| 58 | rs145947882 | C | A | 0.0301 | 0.026076 | -0.136427 | 0.00627244 | 6.90E-105 | 24.02968042 |
| 59 | rs150483923 | A | C | 0.1766 | 0.225932 | 0.0197365 | 0.0023555 | 5.30E-17 | 24.55759497 |
| 60 | rs1540687 | T | A | 0.6098 | 0.674487 | 0.0116627 | 0.00209933 | 2.80E-08 | 13.55256384 |
| 61 | rs1601933 | T | C | 0.4554 | 0.466597 | -0.0842338 | 0.00199576 | 1.00E-200 | 888.713652 |
| 62 | rs1661052 | A | G | 0.9271 | 0.908498 | 0.0293691 | 0.00340349 | 6.20E-18 | 12.3802194 |
| 63 | rs17138358 | C | G | 0.298 | 0.398512 | -0.0261653 | 0.00200552 | 6.60E-39 | 81.61758499 |
| 64 | rs17326656 | T | G | 0.1631 | 0.238733 | -0.0181882 | 0.00230557 | 3.10E-15 | 22.62169014 |
| 65 | rs174566 | G | A | 0.4139 | 0.350206 | -0.0349039 | 0.00205457 | 1.00E-64 | 131.3948063 |
| 66 | rs1789896 | A | G | 0.4555 | 0.513472 | 0.0149273 | 0.00195991 | 2.60E-14 | 28.98509116 |
| 67 | rs1800961 | T | C | 0.04508 | 0.031062 | -0.149001 | 0.00566244 | 1.30E-152 | 41.68409428 |
| 68 | rs1852922 | A | G | 0.712 | 0.69011 | 0.0123581 | 0.00212826 | 6.40E-09 | 14.42193864 |
| 69 | rs1862205 | A | G | 0.4125 | 0.404644 | 0.0110691 | 0.00200091 | 3.20E-08 | 14.74563317 |
| 70 | rs1919309 | C | T | 0.61 | 0.508795 | 0.0116908 | 0.00196745 | 2.80E-09 | 17.64956264 |
| 71 | rs1970811 | C | T | 0.378 | 0.457761 | -0.0116143 | 0.00197362 | 4.00E-09 | 17.19235302 |
| 72 | rs2066714 | C | T | 0.09405 | 0.128552 | 0.054303 | 0.00292716 | 7.90E-77 | 77.12372584 |
| 73 | rs2068888 | A | G | 0.47 | 0.450403 | 0.0134211 | 0.00197105 | 9.80E-12 | 22.95512757 |
| 74 | rs2071379 | G | A | 0.5568 | 0.595245 | -0.0214336 | 0.00199927 | 8.10E-27 | 55.38909696 |
| 75 | rs2111705 | A | G | 0.423 | 0.543888 | -0.0149276 | 0.0019717 | 3.70E-14 | 28.44058204 |
| 76 | rs2159935 | A | G | 0.4794 | 0.490312 | 0.0114396 | 0.00196234 | 5.60E-09 | 16.98621608 |
| 77 | rs2247355 | T | C | 0.2475 | 0.182653 | 0.0177172 | 0.00253838 | 3.00E-12 | 14.54635013 |
| 78 | rs2256720 | C | T | 0.3233 | 0.444499 | 0.0121751 | 0.00197748 | 7.40E-10 | 18.72082449 |
| 79 | rs2269434 | C | T | 0.4418 | 0.345387 | 0.0346097 | 0.00205762 | 1.70E-63 | 127.9749752 |
| 80 | rs2281719 | T | C | 0.5606 | 0.612469 | 0.0534464 | 0.00201161 | 1.50E-155 | 335.3806282 |
| 81 | rs2294915 | T | C | 0.2751 | 0.231744 | -0.0199868 | 0.00233124 | 1.00E-17 | 26.17482081 |
| 82 | rs2297409 | A | G | 0.1231 | 0.194714 | -0.0366326 | 0.00247546 | 1.50E-49 | 68.6870139 |
| 83 | rs2298214 | A | C | 0.5401 | 0.577488 | -0.0131486 | 0.00199824 | 4.70E-11 | 21.12987208 |
| 84 | rs2298624 | T | C | 0.1721 | 0.132474 | 0.0319587 | 0.00288956 | 2.00E-28 | 28.11815165 |
| 85 | rs2302263 | T | C | 0.128 | 0.0887 | -0.0285016 | 0.00345206 | 1.50E-16 | 11.02063857 |
| 86 | rs2305696 | T | C | 0.502 | 0.486258 | -0.018423 | 0.00195245 | 3.90E-21 | 44.48868994 |
| 87 | rs235314 | T | C | 0.4338 | 0.532284 | -0.0214751 | 0.00197484 | 1.50E-27 | 58.88775847 |
| 88 | rs2362541 | G | T | 0.4175 | 0.507575 | -0.0112615 | 0.00195989 | 9.10E-09 | 16.50499594 |
| 89 | rs2395158 | G | A | 0.147 | 0.16935 | -0.0344264 | 0.00261038 | 1.00E-39 | 48.93965051 |
| 90 | rs2419605 | G | A | 0.1294 | 0.147796 | -0.0312837 | 0.00277987 | 2.20E-29 | 31.90483666 |
| 91 | rs2424993 | C | G | 0.7014 | 0.697876 | -0.0161362 | 0.00214155 | 4.90E-14 | 23.94218703 |
| 92 | rs2494747 | T | G | 0.6076 | 0.615762 | -0.0322265 | 0.00202366 | 4.30E-57 | 120.0396551 |
| 93 | rs2520096 | G | A | 0.3984 | 0.270128 | 0.0140397 | 0.00221413 | 2.30E-10 | 15.85517458 |
| 94 | rs2540951 | G | A | 0.3414 | 0.376251 | 0.01211 | 0.00201943 | 2.00E-09 | 16.87969654 |
| 95 | rs2544654 | T | G | 0.7774 | 0.715237 | 0.0128434 | 0.00217845 | 3.70E-09 | 14.15929997 |
| 96 | rs254559 | A | C | 0.3998 | 0.40484 | -0.0170773 | 0.0020013 | 1.40E-17 | 35.09115966 |
| 97 | rs2642438 | G | A | 0.7157 | 0.702624 | 0.0291285 | 0.00213949 | 3.30E-42 | 77.47435458 |
| 98 | rs267738 | G | T | 0.1916 | 0.219299 | 0.0343761 | 0.00236534 | 7.50E-48 | 72.33610498 |
| 99 | rs2726112 | G | A | 0.4579 | 0.453816 | -0.0138549 | 0.00196994 | 2.00E-12 | 24.52304772 |
| 100 | rs2740488 | C | A | 0.1971 | 0.265536 | -0.0804019 | 0.00222407 | 1.00E-200 | 510.4119484 |
| 101 | rs2803619 | C | G | 0.6824 | 0.724683 | -0.0374903 | 0.00219505 | 2.10E-65 | 116.4357847 |
| 102 | rs2804894 | A | G | 0.7872 | 0.734838 | 0.0162537 | 0.00224777 | 4.80E-13 | 20.37764182 |
| 103 | rs286965 | C | T | 0.6274 | 0.632207 | -0.0160285 | 0.00203382 | 3.20E-15 | 28.88571925 |
| 104 | rs2925979 | C | T | 0.6776 | 0.69987 | 0.0285287 | 0.00212908 | 6.10E-41 | 75.44281723 |
| 105 | rs2943645 | T | C | 0.638 | 0.646764 | -0.0338732 | 0.00204772 | 1.80E-61 | 125.0685206 |
| 106 | rs2963468 | G | A | 0.1377 | 0.235288 | -0.0173619 | 0.00232896 | 9.00E-14 | 19.99944765 |
| 107 | rs2965169 | C | A | 0.3556 | 0.388716 | 0.0188372 | 0.00201158 | 7.60E-21 | 41.67804549 |
| 108 | rs2972166 | A | G | 0.331 | 0.274658 | 0.0149974 | 0.00220729 | 1.10E-11 | 18.39485382 |
| 109 | rs2999141 | C | A | 0.2481 | 0.275749 | -0.0121341 | 0.00222468 | 4.90E-08 | 11.88294261 |
| 110 | rs326 | G | A | 0.2606 | 0.293968 | 0.0827185 | 0.00214788 | 1.00E-200 | 616.6206289 |
| 111 | rs33042 | A | G | 0.2535 | 0.246125 | 0.0166688 | 0.00227684 | 2.50E-13 | 19.89062698 |
| 112 | rs34138141 | T | G | 0.2219 | 0.280674 | -0.0180909 | 0.00218637 | 1.30E-16 | 27.64771808 |
| 113 | rs34180494 | C | A | 0.3779 | 0.273764 | -0.0128704 | 0.00219833 | 4.80E-09 | 13.62998156 |
| 114 | rs34397747 | C | T | 0.07708 | 0.084314 | -0.0393293 | 0.00355947 | 2.20E-28 | 18.85196549 |
| 115 | rs34642857 | C | T | 0.1781 | 0.250631 | -0.0133464 | 0.00227583 | 4.50E-09 | 12.9187711 |
| 116 | rs34767118 | G | A | 0.3137 | 0.325924 | 0.0169225 | 0.00210261 | 8.40E-16 | 28.46400623 |
| 117 | rs34955778 | C | T | 0.4404 | 0.418701 | -0.0124025 | 0.00197989 | 3.70E-10 | 19.10243834 |
| 118 | rs35135293 | T | C | 0.5883 | 0.518821 | -0.0217004 | 0.00196743 | 2.70E-28 | 60.75135865 |
| 119 | rs35745599 | T | C | 0.2161 | 0.238631 | 0.0284306 | 0.0022991 | 4.00E-35 | 55.57347371 |
| 120 | rs35909200 | G | T | 0.1677 | 0.157388 | 0.0189094 | 0.00267946 | 1.70E-12 | 13.20999262 |
| 121 | rs36024006 | A | G | 0.448 | 0.451989 | -0.0178019 | 0.00196944 | 1.60E-19 | 40.47965875 |
| 122 | rs367070 | G | A | 0.2991 | 0.225382 | 0.0390583 | 0.00235452 | 8.40E-62 | 96.10868794 |
| 123 | rs3732356 | T | G | 0.9314 | 0.93378 | -0.0367324 | 0.00398002 | 2.70E-20 | 10.53419291 |
| 124 | rs3740688 | T | G | 0.531 | 0.544632 | 0.0156042 | 0.00197254 | 2.60E-15 | 31.04267333 |
| 125 | rs3747973 | G | A | 0.6135 | 0.592553 | 0.0148472 | 0.00199494 | 9.90E-14 | 26.747617 |
| 126 | rs3749748 | T | C | 0.1556 | 0.247259 | 0.0220635 | 0.00228295 | 4.30E-22 | 34.77121652 |
| 127 | rs3760230 | G | C | 0.5861 | 0.579267 | -0.013626 | 0.00198867 | 7.30E-12 | 22.88493867 |
| 128 | rs3768321 | T | G | 0.1645 | 0.196773 | -0.0422764 | 0.00246498 | 6.20E-66 | 93.0044018 |
| 129 | rs3798233 | C | A | 0.4256 | 0.402016 | 0.0179824 | 0.00199898 | 2.30E-19 | 38.91186763 |
| 130 | rs3802548 | A | T | 0.2811 | 0.240427 | 0.0340679 | 0.00229726 | 9.40E-50 | 80.34145639 |
| 131 | rs3818716 | T | C | 0.3308 | 0.364549 | -0.0127148 | 0.00204635 | 5.20E-10 | 17.8872986 |
| 132 | rs3828960 | G | A | 0.2207 | 0.206522 | -0.0146086 | 0.00242641 | 1.70E-09 | 11.88039669 |
| 133 | rs3915932 | C | G | 0.3831 | 0.414974 | 0.0158209 | 0.00199475 | 2.20E-15 | 30.54520202 |
| 134 | rs4052908 | AATT | A | 0.5739 | 0.635826 | 0.0154632 | 0.00205027 | 4.60E-14 | 26.34394038 |
| 135 | rs41272086 | A | G | 0.1011 | 0.105579 | -0.037348 | 0.00319457 | 1.40E-31 | 25.81580752 |
| 136 | rs429358 | C | T | 0.1831 | 0.154478 | -0.102539 | 0.00271512 | 1.00E-200 | 372.9332951 |
| 137 | rs4330777 | A | G | 0.4645 | 0.475043 | -0.0165422 | 0.00195706 | 2.80E-17 | 35.63707531 |
| 138 | rs4441609 | C | T | 0.5357 | 0.625576 | 0.0110807 | 0.00202443 | 4.40E-08 | 14.03511291 |
| 139 | rs4632228 | T | G | 0.2243 | 0.209496 | -0.01986 | 0.00240757 | 1.60E-16 | 22.53892146 |
| 140 | rs4660586 | T | C | 0.6998 | 0.73925 | 0.0125868 | 0.00223611 | 1.80E-08 | 12.21521918 |
| 141 | rs4676609 | T | C | 0.2412 | 0.196448 | 0.0149357 | 0.00247368 | 1.60E-09 | 11.50975887 |
| 142 | rs472629 | A | G | 0.2074 | 0.333138 | -0.0182717 | 0.0020755 | 1.30E-18 | 34.43803827 |
| 143 | rs4762756 | C | T | 0.8106 | 0.73563 | 0.0140023 | 0.00224718 | 4.60E-10 | 15.10216131 |
| 144 | rs4784709 | A | T | 0.9807 | 0.959184 | -0.0617963 | 0.00494325 | 7.40E-36 | 12.23695788 |
| 145 | rs4795386 | G | A | 0.7409 | 0.723594 | 0.0255755 | 0.00220821 | 5.10E-31 | 53.66572524 |
| 146 | rs4807462 | T | A | 0.3684 | 0.359573 | -0.0114556 | 0.0020561 | 2.50E-08 | 14.29708503 |
| 147 | rs4820346 | G | C | 0.6009 | 0.692925 | 0.0130171 | 0.00213798 | 1.10E-09 | 15.77600766 |
| 148 | rs4875043 | C | A | 0.2329 | 0.216842 | -0.0151546 | 0.00240369 | 2.90E-10 | 13.50104964 |
| 149 | rs4883201 | G | A | 0.1173 | 0.102363 | -0.025331 | 0.00323925 | 5.30E-15 | 11.23827579 |
| 150 | rs4930352 | T | G | 0.5282 | 0.494116 | 0.0167274 | 0.00199616 | 5.30E-17 | 35.10852722 |
| 151 | rs532436 | A | G | 0.2006 | 0.184527 | 0.0304626 | 0.00252555 | 1.70E-33 | 43.78926464 |
| 152 | rs55710224 | A | G | 0.4377 | 0.498535 | 0.0121553 | 0.00196433 | 6.10E-10 | 19.14642432 |
| 153 | rs557933 | C | A | 0.5604 | 0.520257 | 0.0190421 | 0.00196414 | 3.20E-22 | 46.92356434 |
| 154 | rs55801554 | A | C | 0.2404 | 0.247289 | 0.0137251 | 0.00227815 | 1.70E-09 | 13.51270127 |
| 155 | rs559355 | T | A | 0.2298 | 0.157349 | -0.0446168 | 0.00269005 | 8.80E-62 | 72.96191576 |
| 156 | rs57274629 | G | A | 0.4536 | 0.35757 | 0.0150043 | 0.0020611 | 3.30E-13 | 24.34865179 |
| 157 | rs58473820 | T | C | 0.3754 | 0.377992 | 0.0207534 | 0.00202552 | 1.20E-24 | 49.37036469 |
| 158 | rs59104589 | T | C | 0.3964 | 0.358199 | 0.0170163 | 0.00204114 | 7.60E-17 | 31.95747972 |
| 159 | rs59347135 | G | C | 0.04075 | 0.046207 | -0.0805948 | 0.00478788 | 1.40E-63 | 24.97726073 |
| 160 | rs6062510 | C | G | 0.7408 | 0.674412 | -0.0152419 | 0.00209559 | 3.50E-13 | 23.23339673 |
| 161 | rs61352607 | T | G | 0.2328 | 0.240776 | 0.0277155 | 0.00229129 | 1.10E-33 | 53.50018889 |
| 162 | rs613808 | G | A | 0.6253 | 0.715399 | -0.0849696 | 0.00218306 | 1.00E-200 | 617.8602487 |
| 163 | rs61805076 | C | T | 0.2877 | 0.333426 | -0.015348 | 0.00207699 | 1.50E-13 | 24.27376813 |
| 164 | rs61980899 | C | T | 0.4972 | 0.537847 | -0.0139977 | 0.00197925 | 1.50E-12 | 24.8663381 |
| 165 | rs62114506 | C | G | 0.3203 | 0.262092 | 0.0140502 | 0.00222524 | 2.70E-10 | 15.42098446 |
| 166 | rs62135193 | T | C | 0.461 | 0.541376 | -0.0108289 | 0.00197527 | 4.20E-08 | 14.92505015 |
| 167 | rs6224 | T | G | 0.3991 | 0.473585 | 0.0125532 | 0.0019683 | 1.80E-10 | 20.28162243 |
| 168 | rs62405458 | T | C | 0.2345 | 0.18267 | -0.0147906 | 0.00254902 | 6.50E-09 | 10.05376334 |
| 169 | rs6448429 | T | C | 0.1468 | 0.166112 | -0.0222015 | 0.0026563 | 6.40E-17 | 19.35389076 |
| 170 | rs6469605 | T | C | 0.6938 | 0.568386 | 0.0349728 | 0.00198158 | 1.00E-69 | 152.8878973 |
| 171 | rs6505176 | G | T | 0.3207 | 0.331623 | -0.0174387 | 0.00209523 | 8.60E-17 | 30.71090769 |
| 172 | rs6557267 | T | C | 0.438 | 0.403524 | -0.0118925 | 0.00200039 | 2.80E-09 | 17.0147626 |
| 173 | rs6705285 | T | G | 0.6765 | 0.609051 | 0.0111578 | 0.00197677 | 1.70E-08 | 15.17271061 |
| 174 | rs676210 | A | G | 0.2662 | 0.205092 | 0.0600946 | 0.00242305 | 8.71E-136 | 200.6601352 |
| 175 | rs6765484 | T | C | 0.5608 | 0.472703 | 0.0204373 | 0.00196501 | 2.50E-25 | 53.9322479 |
| 176 | rs6772763 | C | T | 0.5937 | 0.565136 | 0.0128955 | 0.00198642 | 8.50E-11 | 20.71530394 |
| 177 | rs6807935 | G | A | 0.366 | 0.343276 | -0.0126887 | 0.00206633 | 8.20E-10 | 17.00230457 |
| 178 | rs681869 | T | C | 0.7533 | 0.704152 | -0.0165331 | 0.00215035 | 1.50E-14 | 24.63090249 |
| 179 | rs686030 | A | C | 0.8813 | 0.858646 | 0.056386 | 0.00281822 | 4.70E-89 | 97.19639849 |
| 180 | rs6977416 | A | G | 0.3598 | 0.334458 | -0.0157606 | 0.00209756 | 5.70E-14 | 25.1356033 |
| 181 | rs698927 | C | A | 0.1811 | 0.183778 | 0.0245539 | 0.00252916 | 2.80E-22 | 28.27803276 |
| 182 | rs7032795 | C | T | 0.4772 | 0.493507 | 0.0145998 | 0.00197016 | 1.30E-13 | 27.4546362 |
| 183 | rs7036107 | G | A | 0.5141 | 0.511088 | -0.0112609 | 0.00200049 | 1.80E-08 | 15.8359854 |
| 184 | rs7136506 | C | T | 0.2213 | 0.216465 | -0.0318107 | 0.00242195 | 2.10E-39 | 58.52677841 |
| 185 | rs7147511 | T | C | 0.4219 | 0.495499 | -0.0146441 | 0.00198342 | 1.50E-13 | 27.25578532 |
| 186 | rs7170463 | G | A | 0.3476 | 0.311339 | 0.0161797 | 0.00211746 | 2.20E-14 | 25.03830676 |
| 187 | rs7216643 | T | C | 0.2237 | 0.223559 | -0.0223626 | 0.00236278 | 2.90E-21 | 31.10002758 |
| 188 | rs7238484 | T | G | 0.1996 | 0.268307 | -0.0213669 | 0.00221605 | 5.30E-22 | 36.50504889 |
| 189 | rs7251640 | C | T | 0.2168 | 0.194006 | 0.0151586 | 0.00249926 | 1.30E-09 | 11.50489802 |
| 190 | rs72926946 | A | C | 0.2762 | 0.296458 | -0.0154139 | 0.00214215 | 6.20E-13 | 21.5988269 |
| 191 | rs7304603 | C | T | 0.6391 | 0.537686 | -0.0127358 | 0.00197331 | 1.10E-10 | 20.70991241 |
| 192 | rs73052033 | C | T | 0.1619 | 0.185091 | -0.0184412 | 0.00253065 | 3.20E-13 | 16.01968911 |
| 193 | rs7305678 | G | T | 0.8683 | 0.866252 | -0.0219163 | 0.00291351 | 5.40E-14 | 13.11219089 |
| 194 | rs73216701 | G | A | 0.428 | 0.429931 | 0.0164708 | 0.00198166 | 9.40E-17 | 33.8658888 |
| 195 | rs737338 | T | C | 0.06366 | 0.035108 | -0.10808 | 0.00533159 | 2.30E-91 | 27.84328786 |
| 196 | rs7503353 | T | G | 0.5925 | 0.532015 | -0.0135158 | 0.00197236 | 7.30E-12 | 23.38409494 |
| 197 | rs75104038 | A | G | 0.03373 | 0.06109 | -0.0391467 | 0.00409914 | 1.30E-21 | 10.46256683 |
| 198 | rs75265117 | G | C | 0.0996 | 0.11993 | 0.0316195 | 0.00301917 | 1.20E-25 | 23.1543862 |
| 199 | rs75406471 | A | G | 0.1415 | 0.154253 | -0.0246389 | 0.00272053 | 1.30E-19 | 21.40233226 |
| 200 | rs76213248 | T | C | 0.4113 | 0.410374 | 0.021302 | 0.00200107 | 1.80E-26 | 54.84802905 |
| 201 | rs7700617 | A | C | 0.6273 | 0.497345 | -0.0127352 | 0.00196898 | 9.90E-11 | 20.9173943 |
| 202 | rs77960347 | G | A | 0.007282 | 0.01329 | 0.335992 | 0.00855615 | 1.00E-200 | 40.44716296 |
| 203 | rs78058190 | A | G | 0.08195 | 0.050441 | -0.0662337 | 0.00504914 | 2.60E-39 | 16.48446452 |
| 204 | rs7817574 | C | T | 0.2473 | 0.184902 | 0.0362105 | 0.002519 | 7.40E-47 | 62.29608211 |
| 205 | rs78296522 | A | C | 0.04237 | 0.045117 | 0.0682915 | 0.00475519 | 9.00E-47 | 17.7719608 |
| 206 | rs7952521 | A | G | 0.1112 | 0.105535 | 0.0270189 | 0.00330241 | 2.80E-16 | 12.63790126 |
| 207 | rs79984435 | A | G | 0.0948 | 0.092203 | -0.0855866 | 0.00337218 | 4.20E-142 | 107.8625678 |
| 208 | rs8086351 | G | C | 0.8295 | 0.824039 | 0.096182 | 0.00257748 | 1.00E-200 | 404.2362332 |
| 209 | rs8098618 | T | C | 0.5045 | 0.479414 | 0.0115362 | 0.00197395 | 5.10E-09 | 17.04916337 |
| 210 | rs8103728 | G | C | 0.658 | 0.669595 | 0.0228493 | 0.00208825 | 7.30E-28 | 52.98170562 |
| 211 | rs921919 | A | G | 0.5708 | 0.669251 | -0.0371454 | 0.00212658 | 2.50E-68 | 135.1173753 |
| 212 | rs9347737 | G | A | 0.4506 | 0.428349 | -0.0111198 | 0.00200525 | 2.90E-08 | 15.06017602 |
| 213 | rs9426827 | C | T | 0.4605 | 0.478096 | 0.0170728 | 0.00195828 | 2.80E-18 | 37.93458602 |
| 214 | rs9471972 | A | G | 0.4455 | 0.53516 | 0.0268788 | 0.00196789 | 1.80E-42 | 92.84009399 |
| 215 | rs9604045 | T | G | 0.1715 | 0.253532 | 0.0154221 | 0.00235515 | 5.80E-11 | 16.23078689 |
| 216 | rs9608972 | C | T | 0.2031 | 0.241455 | -0.0174956 | 0.00230014 | 2.80E-14 | 21.19424204 |
| 217 | rs9647335 | T | A | 0.1193 | 0.191878 | 0.0316374 | 0.00249696 | 8.60E-37 | 49.79242687 |
| 218 | rs976002 | G | A | 0.2381 | 0.24529 | 0.0230909 | 0.00228117 | 4.40E-24 | 37.93988903 |
| 219 | rs9817452 | T | G | 0.3121 | 0.387699 | 0.0174889 | 0.00202187 | 5.20E-18 | 35.5259096 |
| 220 | rs9977268 | T | C | 0.1638 | 0.19875 | -0.0182291 | 0.00246656 | 1.50E-13 | 17.39679587 |
| 221 | rs9987289 | G | A | 0.8574 | 0.908733 | 0.0852351 | 0.00339983 | 1.00E-138 | 104.2834949 |
| 222 | rs9989419 | G | A | 0.6153 | 0.60551 | 0.114993 | 0.00199407 | 1.00E-200 | 1595.164478 |
| **Exposures** | **SNP** | **Effect**  **allele** | **Other**  **allele** | **Eaf**  **Outcomes** | **Eaf**  **Exposures** | **Beta**  **Exposures** | **Se**  **Exposures** | **P**  **Exposures** | **F statistic** |
| **ApoB** |  |  |  |  |  |  |  |  |  |
| 1 | rs1003533 | T | C | 0.2167 | 0.188327 | -0.0200281 | 0.00266165 | 5.30E-14 | 17.31077184 |
| 2 | rs10087526 | G | T | 0.2257 | 0.198764 | 0.0187619 | 0.00260759 | 6.20E-13 | 16.48988114 |
| 3 | rs10127775 | T | A | 0.5463 | 0.60619 | -0.0233243 | 0.00212188 | 4.20E-28 | 57.69739058 |
| 4 | rs10448340 | G | T | 0.3038 | 0.319766 | -0.0170557 | 0.00223016 | 2.00E-14 | 25.44549187 |
| 5 | rs10794579 | C | T | 0.6243 | 0.575021 | 0.0137267 | 0.00210335 | 6.80E-11 | 20.81656034 |
| 6 | rs10832963 | G | T | 0.6174 | 0.744199 | 0.0219536 | 0.00238777 | 3.80E-20 | 32.18679059 |
| 7 | rs10953298 | T | C | 0.2469 | 0.236254 | -0.0170824 | 0.00245825 | 3.70E-12 | 17.42685617 |
| 8 | rs11047939 | A | G | 0.2945 | 0.224841 | 0.0152893 | 0.00250351 | 1.00E-09 | 13.00120838 |
| 9 | rs11057397 | T | C | 0.3064 | 0.337102 | -0.0183422 | 0.00220085 | 7.80E-17 | 31.04478906 |
| 10 | rs11099097 | T | C | 0.3451 | 0.291413 | -0.0161746 | 0.00229432 | 1.80E-12 | 20.52624616 |
| 11 | rs112758337 | A | G | 0.1543 | 0.185556 | -0.0212571 | 0.00267849 | 2.10E-15 | 19.03754947 |
| 12 | rs11591147 | T | G | 0.03605 | 0.017452 | -0.345954 | 0.00790085 | 1.00E-200 | 65.76292168 |
| 13 | rs11601507 | A | C | 0.07935 | 0.069438 | 0.0414121 | 0.004036 | 1.10E-24 | 13.60617906 |
| 14 | rs11621792 | T | C | 0.3686 | 0.452905 | 0.0210752 | 0.00210348 | 1.30E-23 | 49.75236235 |
| 15 | rs116734477 | T | C | 0.02994 | 0.040814 | -0.0633493 | 0.00526644 | 2.50E-33 | 11.32923463 |
| 16 | rs11709868 | T | G | 0.2613 | 0.297035 | -0.015551 | 0.00228244 | 9.50E-12 | 19.38681289 |
| 17 | rs118039278 | A | G | 0.04597 | 0.078706 | 0.0868026 | 0.00387054 | 2.20E-111 | 72.95055116 |
| 18 | rs11901691 | A | C | 0.5403 | 0.577074 | 0.0142576 | 0.00211213 | 1.50E-11 | 22.24321023 |
| 19 | rs12046278 | C | T | 0.43 | 0.34509 | -0.0156231 | 0.00217266 | 6.40E-13 | 23.37308362 |
| 20 | rs12054451 | G | T | 0.236 | 0.259497 | 0.0159506 | 0.00238162 | 2.10E-11 | 17.23905036 |
| 21 | rs12078100 | G | C | 0.7215 | 0.623091 | 0.0152136 | 0.00213952 | 1.20E-12 | 23.75039155 |
| 22 | rs12208357 | T | C | 0.05701 | 0.07008 | 0.0629791 | 0.0040818 | 1.00E-53 | 31.03043322 |
| 23 | rs12469941 | T | C | 0.452 | 0.400959 | 0.0134202 | 0.00209668 | 1.50E-10 | 19.68147595 |
| 24 | rs12471768 | C | T | 0.7691 | 0.704041 | 0.0139101 | 0.00226938 | 8.80E-10 | 15.65737886 |
| 25 | rs1250258 | T | C | 0.7884 | 0.736741 | 0.0141509 | 0.00235577 | 1.90E-09 | 13.99723568 |
| 26 | rs1260326 | C | T | 0.6493 | 0.604525 | -0.0495555 | 0.00211469 | 1.90E-121 | 262.7310079 |
| 27 | rs12603290 | C | T | 0.5333 | 0.514724 | -0.0288448 | 0.00207989 | 9.80E-44 | 96.10380827 |
| 28 | rs12691088 | A | G | 0.03412 | 0.02091 | 0.246111 | 0.00769582 | 1.00E-200 | 41.87919466 |
| 29 | rs12916 | C | T | 0.4542 | 0.400485 | 0.0549288 | 0.00212005 | 5.20E-148 | 322.5827914 |
| 30 | rs13076933 | G | T | 0.2708 | 0.259424 | -0.0182624 | 0.00238248 | 1.80E-14 | 22.57809533 |
| 31 | rs13108218 | G | A | 0.6773 | 0.614849 | -0.0229367 | 0.00215262 | 1.60E-26 | 53.77839274 |
| 32 | rs13230111 | G | A | 0.4639 | 0.492349 | -0.0138317 | 0.00207813 | 2.80E-11 | 22.14592983 |
| 33 | rs13247874 | T | C | 0.1779 | 0.197154 | -0.0215939 | 0.00261336 | 1.40E-16 | 21.61478518 |
| 34 | rs13379043 | C | T | 0.1988 | 0.280473 | -0.0143675 | 0.00235876 | 1.10E-09 | 14.97531214 |
| 35 | rs13389219 | T | C | 0.3486 | 0.392571 | -0.0161286 | 0.00211805 | 2.60E-14 | 27.65604202 |
| 36 | rs1358980 | T | C | 0.4626 | 0.483147 | 0.0149035 | 0.00209447 | 1.10E-12 | 25.28876421 |
| 37 | rs13702 | C | T | 0.2591 | 0.287911 | -0.0295019 | 0.00229451 | 7.80E-38 | 67.79655855 |
| 38 | rs138354 | C | T | 0.4545 | 0.534598 | -0.0120708 | 0.00208392 | 6.90E-09 | 16.69591114 |
| 39 | rs138692741 | T | C | 0.03301 | 0.035781 | 0.0714986 | 0.00568647 | 3.00E-36 | 10.90878884 |
| 40 | rs143020224 | G | C | 0.1022 | 0.118569 | -0.165274 | 0.0031786 | 1.00E-200 | 565.8273633 |
| 41 | rs144926613 | T | TTGGAGGGCAGACTAGCCCAGGCCC | 0.7128 | 0.671796 | -0.0145634 | 0.00220815 | 4.20E-11 | 19.18212342 |
| 42 | rs148150904 | TTAAAG | T | 0.2587 | 0.189665 | 0.0159241 | 0.00263918 | 1.60E-09 | 11.19083895 |
| 43 | rs148601586 | G | C | 0.002831 | 0.013309 | 0.183504 | 0.00917667 | 5.90E-89 | 10.50233426 |
| 44 | rs148933445 | A | G | 0.0211 | 0.021557 | -0.573249 | 0.00759178 | 1.00E-200 | 240.6518173 |
| 45 | rs150474434 | A | G | 0.0787 | 0.101399 | -0.0343097 | 0.00345194 | 2.80E-23 | 18.00336778 |
| 46 | rs1556562 | T | G | 0.778 | 0.790018 | 0.0159957 | 0.00247804 | 1.10E-10 | 13.82453772 |
| 47 | rs1561139 | T | G | 0.3246 | 0.424334 | -0.0131421 | 0.0021042 | 4.20E-10 | 19.05815154 |
| 48 | rs17050272 | A | G | 0.5353 | 0.40926 | -0.0244302 | 0.00210465 | 3.80E-31 | 65.16019869 |
| 49 | rs174564 | G | A | 0.4151 | 0.348509 | -0.0450573 | 0.00218206 | 1.00E-94 | 193.70413 |
| 50 | rs17569873 | T | C | 0.1377 | 0.201289 | 0.0183445 | 0.00259488 | 1.60E-12 | 16.07055284 |
| 51 | rs183130 | T | C | 0.2796 | 0.323911 | -0.0478017 | 0.00222241 | 1.30E-102 | 202.7198647 |
| 52 | rs1888488 | T | C | 0.6673 | 0.565289 | 0.0173586 | 0.00210255 | 1.50E-16 | 33.50186888 |
| 53 | rs200046586 | C | CA | 0.98386 | 0.981247 | 0.412271 | 0.00778939 | 1.00E-200 | 103.1187742 |
| 54 | rs2043085 | C | T | 0.5792 | 0.612252 | -0.0193562 | 0.00213525 | 1.20E-19 | 39.02016658 |
| 55 | rs2068888 | A | G | 0.47 | 0.4508 | -0.0241729 | 0.00208746 | 5.20E-31 | 66.40936097 |
| 56 | rs2073547 | G | A | 0.3265 | 0.183953 | 0.0312042 | 0.00266416 | 1.10E-31 | 41.19042676 |
| 57 | rs2137234 | C | T | 0.1517 | 0.195281 | 0.0152817 | 0.00262327 | 5.70E-09 | 10.66596525 |
| 58 | rs2160994 | C | T | 0.665 | 0.647429 | 0.0172687 | 0.00217691 | 2.10E-15 | 28.72985951 |
| 59 | rs2199048 | G | A | 0.227 | 0.313756 | -0.0132194 | 0.00224306 | 3.80E-09 | 14.95736577 |
| 60 | rs2238162 | T | C | 0.5627 | 0.522997 | -0.0226439 | 0.00208185 | 1.50E-27 | 59.03509729 |
| 61 | rs224391 | C | G | 0.2193 | 0.227492 | -0.0174615 | 0.00248326 | 2.00E-12 | 17.37932991 |
| 62 | rs2287622 | G | A | 0.5098 | 0.602775 | -0.0203449 | 0.00211842 | 7.70E-22 | 44.17234772 |
| 63 | rs2519093 | T | C | 0.2006 | 0.184543 | 0.0410869 | 0.00267778 | 3.90E-53 | 70.86857367 |
| 64 | rs2618566 | T | G | 0.6771 | 0.659901 | -0.0303008 | 0.00219303 | 2.00E-43 | 85.70693075 |
| 65 | rs2737263 | T | G | 0.3084 | 0.280253 | -0.0185263 | 0.00231348 | 1.20E-15 | 25.87197716 |
| 66 | rs2738447 | C | A | 0.6063 | 0.592706 | 0.0436273 | 0.00209036 | 9.90E-97 | 210.4058712 |
| 67 | rs2761311 | T | C | 0.5268 | 0.571902 | 0.012077 | 0.00211012 | 1.00E-08 | 16.04030115 |
| 68 | rs278981 | C | T | 0.7608 | 0.758151 | 0.0148256 | 0.00239561 | 6.10E-10 | 14.04540403 |
| 69 | rs2807854 | C | T | 0.6304 | 0.671334 | 0.0162948 | 0.00220437 | 1.40E-13 | 24.11428385 |
| 70 | rs28406917 | T | C | 0.4491 | 0.427571 | 0.0134005 | 0.00210735 | 2.00E-10 | 19.79457231 |
| 71 | rs28601761 | G | C | 0.4126 | 0.418842 | -0.07256 | 0.00212854 | 1.00E-200 | 566.4520182 |
| 72 | rs28814720 | G | A | 0.4586 | 0.519754 | 0.0117382 | 0.00214286 | 4.30E-08 | 14.98027688 |
| 73 | rs3127580 | T | C | 0.124 | 0.155127 | 0.0404476 | 0.00286539 | 3.00E-45 | 52.23679527 |
| 74 | rs34042070 | G | C | 0.1947 | 0.187904 | 0.0494204 | 0.00267088 | 1.90E-76 | 104.5149717 |
| 75 | rs35081008 | T | C | 0.1302 | 0.148139 | -0.0311143 | 0.00290126 | 7.80E-27 | 29.02963899 |
| 76 | rs35882350 | G | A | 0.272 | 0.261055 | 0.0135528 | 0.0023664 | 1.00E-08 | 12.65512407 |
| 77 | rs35980001 | G | GC | 0.7673 | 0.786526 | -0.0244043 | 0.00255807 | 1.40E-21 | 30.56498397 |
| 78 | rs3746337 | T | C | 0.513 | 0.489153 | 0.0156368 | 0.00207666 | 5.10E-14 | 28.33717637 |
| 79 | rs3822855 | T | G | 0.4256 | 0.401636 | 0.0148392 | 0.00211668 | 2.40E-12 | 23.62435886 |
| 80 | rs3823376 | T | C | 0.4554 | 0.502185 | 0.0164137 | 0.00207374 | 2.50E-15 | 31.32528851 |
| 81 | rs3860846 | T | C | 0.2504 | 0.275298 | 0.0138403 | 0.00235387 | 4.10E-09 | 13.79527096 |
| 82 | rs4052908 | AATT | A | 0.5739 | 0.636165 | -0.012576 | 0.00217168 | 7.00E-09 | 15.52426361 |
| 83 | rs4307732 | A | G | 0.157 | 0.105893 | 0.0481874 | 0.00338596 | 5.80E-46 | 38.35535717 |
| 84 | rs4470903 | G | C | 0.2147 | 0.214994 | 0.0345326 | 0.00253303 | 2.60E-42 | 62.74328601 |
| 85 | rs454715 | G | T | 0.7159 | 0.582689 | -0.0235286 | 0.0020918 | 2.40E-29 | 61.53714874 |
| 86 | rs45537841 | T | C | 0.1948 | 0.180708 | -0.0158986 | 0.00270418 | 4.10E-09 | 10.23530539 |
| 87 | rs4671050 | T | G | 0.3584 | 0.316138 | -0.0196981 | 0.00222973 | 1.00E-18 | 33.74820528 |
| 88 | rs4689088 | A | G | 0.6274 | 0.618684 | 0.0124572 | 0.00214057 | 5.90E-09 | 15.98010901 |
| 89 | rs472495 | T | G | 0.5767 | 0.649019 | 0.0407634 | 0.00217172 | 1.30E-78 | 160.5687096 |
| 90 | rs4782568 | G | C | 0.4383 | 0.450839 | -0.0182854 | 0.00210073 | 3.20E-18 | 37.51930076 |
| 91 | rs478975 | A | G | 0.5602 | 0.642584 | 0.0141364 | 0.00217403 | 7.90E-11 | 19.42216795 |
| 92 | rs4935356 | A | T | 0.1481 | 0.243914 | 0.0245813 | 0.00255516 | 6.60E-22 | 34.13840229 |
| 93 | rs4935356 | A | T | 0.3147 | 0.243914 | 0.0245813 | 0.00255516 | 6.60E-22 | 34.13840229 |
| 94 | rs546240 | T | C | 0.5634 | 0.619967 | -0.0118734 | 0.00214585 | 3.10E-08 | 14.42725286 |
| 95 | rs556107 | T | C | 0.5675 | 0.523242 | 0.0358479 | 0.00207937 | 1.30E-66 | 148.3334655 |
| 96 | rs55714927 | T | C | 0.2643 | 0.190338 | -0.0321831 | 0.00264686 | 5.10E-34 | 45.57179589 |
| 97 | rs55831924 | T | C | 0.3327 | 0.361347 | 0.0182553 | 0.00217539 | 4.80E-17 | 32.50523134 |
| 98 | rs581080 | C | G | 0.8548 | 0.818992 | 0.0185579 | 0.00270151 | 6.40E-12 | 13.9914841 |
| 99 | rs59328596 | A | G | 0.1777 | 0.148104 | -0.0210021 | 0.00292183 | 6.60E-13 | 13.03796567 |
| 100 | rs597808 | G | A | 0.5853 | 0.515902 | 0.0216847 | 0.00208463 | 2.40E-25 | 54.05437291 |
| 101 | rs6072279 | A | G | 0.5324 | 0.473669 | 0.0292319 | 0.00208189 | 8.70E-45 | 98.3235777 |
| 102 | rs6073958 | C | T | 0.176 | 0.198642 | 0.0423193 | 0.00260555 | 2.50E-59 | 84.00146395 |
| 103 | rs6129620 | A | T | 0.2912 | 0.338295 | -0.0253099 | 0.00223276 | 8.70E-30 | 57.53626605 |
| 104 | rs62119267 | C | A | 0.009432 | 0.022316 | -0.33464 | 0.00708026 | 1.00E-200 | 97.49819276 |
| 105 | rs62122481 | A | C | 0.308 | 0.376906 | 0.0841137 | 0.00214492 | 1.00E-200 | 723.5036557 |
| 106 | rs6426328 | T | G | 0.3857 | 0.489784 | 0.0122017 | 0.00207321 | 4.00E-09 | 17.31242478 |
| 107 | rs6475606 | T | C | 0.4136 | 0.484473 | -0.017332 | 0.00207721 | 7.20E-17 | 34.77922916 |
| 108 | rs6560499 | A | G | 0.6252 | 0.575961 | -0.0121606 | 0.00211507 | 9.00E-09 | 16.14742884 |
| 109 | rs6602909 | C | T | 0.3449 | 0.327566 | 0.021441 | 0.0022203 | 4.60E-22 | 41.08505558 |
| 110 | rs6657811 | T | A | 0.1011 | 0.129938 | -0.127721 | 0.00308568 | 1.00E-200 | 387.7220124 |
| 111 | rs6874202 | C | T | 0.6678 | 0.634225 | 0.0312135 | 0.00215543 | 1.60E-47 | 97.31923922 |
| 112 | rs6940814 | G | A | 0.5045 | 0.587465 | -0.0199476 | 0.00210823 | 3.00E-21 | 43.3969341 |
| 113 | rs7012637 | A | G | 0.5077 | 0.473809 | 0.0200133 | 0.00209504 | 1.30E-21 | 45.5064028 |
| 114 | rs7249565 | A | G | 0.4533 | 0.41494 | 0.0145524 | 0.00208362 | 2.90E-12 | 23.68478021 |
| 115 | rs72631343 | G | C | 0.1426 | 0.128891 | -0.0278921 | 0.00310009 | 2.30E-19 | 18.17836876 |
| 116 | rs72823013 | A | G | 0.09362 | 0.126195 | -0.0228563 | 0.00313773 | 3.20E-13 | 11.70245496 |
| 117 | rs75331444 | A | G | 0.08358 | 0.065607 | -0.0903607 | 0.00418412 | 2.00E-103 | 57.18935904 |
| 118 | rs7569317 | C | T | 0.5179 | 0.530625 | 0.0180783 | 0.0020715 | 2.60E-18 | 37.9418496 |
| 119 | rs7590687 | C | T | 0.9303 | 0.923067 | -0.0430516 | 0.00398578 | 3.40E-27 | 16.57075293 |
| 120 | rs7603427 | T | C | 0.4424 | 0.53344 | 0.0135724 | 0.00207851 | 6.60E-11 | 21.22516866 |
| 121 | rs76186504 | T | C | 0.01763 | 0.025023 | -0.11643 | 0.0066216 | 3.30E-69 | 15.0862254 |
| 122 | rs7707394 | A | G | 0.3732 | 0.357327 | 0.0352503 | 0.00216546 | 1.40E-59 | 121.7389546 |
| 123 | rs7734476 | A | G | 0.5056 | 0.550001 | 0.019942 | 0.00208686 | 1.20E-21 | 45.20625578 |
| 124 | rs7746081 | A | G | 0.3701 | 0.30414 | -0.0227685 | 0.00225861 | 6.70E-24 | 43.01820785 |
| 125 | rs77498041 | A | G | 0.1814 | 0.19111 | -0.0162677 | 0.00265126 | 8.50E-10 | 11.64019021 |
| 126 | rs77542162 | G | A | 0.006795 | 0.022483 | 0.115967 | 0.00701839 | 2.50E-61 | 12.00085845 |
| 127 | rs79220007 | C | T | 0.03725 | 0.076094 | -0.0537922 | 0.00390806 | 4.20E-43 | 26.64088997 |
| 128 | rs8016418 | A | T | 0.4139 | 0.374825 | 0.0197194 | 0.00214984 | 4.60E-20 | 39.43413113 |
| 129 | rs8107974 | T | A | 0.06479 | 0.075757 | -0.0908575 | 0.00388309 | 4.50E-121 | 76.67952663 |
| 130 | rs9297994 | A | G | 0.6225 | 0.664535 | -0.0291083 | 0.00220081 | 6.20E-40 | 78.00793903 |
| 131 | rs9482772 | C | T | 0.4436 | 0.448075 | 0.0177235 | 0.00208937 | 2.20E-17 | 35.59280872 |
| 132 | rs9496567 | A | G | 0.2077 | 0.243555 | -0.0190492 | 0.00242335 | 3.80E-15 | 22.76911418 |
| 133 | rs9616822 | A | G | 0.4326 | 0.351376 | 0.0159299 | 0.00218001 | 2.70E-13 | 24.34032212 |
| 134 | rs964184 | C | G | 0.854 | 0.86677 | -0.0765255 | 0.00305668 | 2.50E-138 | 144.8069747 |
| 135 | rs969075 | C | T | 0.7197 | 0.664927 | 0.0138101 | 0.00221388 | 4.40E-10 | 17.33979899 |
| 136 | rs9834932 | G | A | 0.08202 | 0.088856 | -0.0317059 | 0.00365151 | 3.90E-18 | 12.20812733 |
| 137 | rs9884390 | C | T | 0.2312 | 0.234076 | 0.023248 | 0.00248318 | 7.80E-21 | 31.4308736 |
| **Exposures** | **SNP** | **Effect**  **allele** | **Other**  **allele** | **Eaf**  **Outcomes** | **Eaf**  **Exposures** | **Beta**  **Exposures** | **Se**  **Exposures** | **P**  **Exposures** | **F statistic** |
| **Lp(a)** |  |  |  |  |  |  |  |  |  |
| 1 | rs10455872 | G | A | 0.04595 | 0.071668 | 89.361 | 0.20378 | 1.00E-200 | 28224.2777 |
| 2 | rs1086567 | A | G | 0.6103 | 0.62565 | 2.8444 | 0.13841 | 8.98E-94 | 197.9685969 |
| 3 | rs112110249 | C | T | 0.07096 | 0.043323 | -10.644 | 0.32856 | 1.00E-200 | 87.02198689 |
| 4 | rs117733303 | G | A | 0.01133 | 0.0083134 | 43.396 | 0.73219 | 1.00E-200 | 57.93267888 |
| 5 | rs117857195 | T | G | 0.02004 | 0.023342 | -16.547 | 0.47734 | 1.00E-200 | 54.79957122 |
| 6 | rs117881880 | A | T | 0.009332 | 0.015013 | -13.415 | 0.55714 | 5.64E-128 | 17.14765238 |
| 7 | rs12086910 | T | A | 0.5465 | 0.56506 | -0.75034 | 0.13516 | 2.84E-08 | 15.14937067 |
| 8 | rs12179053 | T | C | 0.2787 | 0.25948 | -9.2946 | 0.15168 | 1.00E-200 | 1450.662614 |
| 9 | rs146534110 | T | G | 0.005616 | 0.012612 | 28.147 | 0.59737 | 1.00E-200 | 55.30491445 |
| 10 | rs184158723 | A | G | 0.01711 | 0.018569 | 18.557 | 0.49506 | 1.00E-200 | 51.22195902 |
| 11 | rs3127580 | T | C | 0.124 | 0.15747 | 27.895 | 0.17692 | 1.00E-200 | 6759.217199 |
| 12 | rs494554 | G | C | 0.01969 | 0.027235 | -9.6137 | 0.42587 | 9.89E-113 | 27.0042421 |
| 13 | rs9355328 | C | T | 0.9569 | 0.974544 | 6.1439 | 0.43167 | 5.96E-46 | 10.05122322 |
